# Facile Oxidative Rearrangements Using Hypervalent Iodine Reagents

**DOI:** 10.1002/open.201200037

**Published:** 2012-11-06

**Authors:** Fateh V Singh, Julia Rehbein, Thomas Wirth

**Affiliations:** [a] School of Chemistry, Cardiff UniversityPark Place, Main Building, Cardiff CF10 3AT (United Kingdom) E-mail: wirth@cf.ac.uk

**Keywords:** calculations, cyclizations, hypervalent iodine, oxidations, rearrangements

## Abstract

Aromatic substituents migrate in a novel oxidative cyclization mediated by iodine(III) reagents. 4-Arylbut-3-enoic acids are cyclized and rearranged to 4-arylfuran-2(5*H*)-ones by hypervalent iodine compounds in good to excellent yields under mild reaction conditions. Other ring sizes are also accessible. The mechanism of the reaction is described in detail, and calculations highlight the cationic nature of the intermediates in the rearrangement. The fast access to heavily substituted furanones is used for the synthesis of biologically active derivatives.

## Introduction

Hypervalent iodine reagents have received particular attention in the area of synthetic chemistry and have found wide applications in synthesis due to their environmentally friendly nature, low toxicity and easy accessibility.^1^ Because of their high electrophilic nature, hypervalent iodine(III) compounds have been frequently used to activate carbon–carbon double bonds. The facile formation of cationic intermediates has been used in different rearrangements for the synthesis of functionalized molecules,^2^ including oxidative ring expansions^3^ and iodine(III)-mediated ring contractions.^4^ We have developed oxidative cyclizations^5^ including the cyclization of unsaturated acids with a 1,2-migration of aryl groups.^6^

## Results and Discussion

**Scheme 1:**
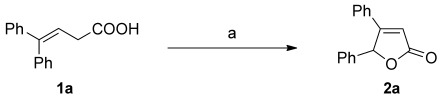
Cyclization and rearrangement of acid 1 a to furanone 2 a. *Reagents and conditions*: a) PhI(OCOCF_3_)_2_ (1 equiv), TMSOTf (2 equiv), MeCN, RT, 30 min, 93 %.

Herein, we report a novel oxidative rearrangement of 4-arylbut-3-enoic acids to 4-arylfuran-2(5*H*)-ones mediated by hypervalent iodine compounds in high yields under mild reaction conditions. Optimal reagents reaction conditions were established using 4,4-diphenylbut-3-enoic acid (**1 a**) as model substrate.^7^ The rearrangement of **1 a** to 4,5-diphenylfuran-2(5*H*)-one (**2 a**) was carried out in acetonitrile using a hypervalent iodine reagent with two equivalents of trimethylsilyl triflate (TMSOTf; see Scheme 1). We have recently used this reagent combination for the generation of the highly reactive [bis(trifluoromethylsulfonyl)iodo]benzene, and it is assumed that this is the active reagent as there is no reaction without addition of TMSOTf.5b

The rearranged product **2 a** was obtained in very good yields with different iodine(III) reagents in combination with TMSOTf (see Supporting Information, Table S1). The combination of TMSOTf and iodine(III) reagents of type PhIRR′ (R=R′=OCOCF_3_; R=R′=OAc; R=OH, R′=OTs) leads to the in situ formation of PhI(OTf)_2_ resulting in yields between 85 % and 93 %, whereas iodosylbenzene (PhIO) or the cyclic hypervalent iodine(V) reagent 2-iodoxybenzoic acid (IBX) with TMSOTf led to product **2 a** in only 32 % and 21 % yield, respectively. A bridged reagent with two iodine(III) moieties [μ-oxobis(trifluoroacetoxyiodo)benzene]^8^ is very reactive but also leads to side-product formation with **2 a** formed in 84 % yield.

The substituents R and R′ in trivalent iodine compounds PhIRR′ can be exchanged easily, their nature has a strong influence on the reactivity. We investigated different Lewis acids to activate [bis(trifluoroacetoxy)iodo]benzene as shown in Table 1, as there is no reaction without activating the hypervalent iodine reagent (Table 1, Entry 1). Initially, TMSOTf and *tert*-butyldimethylsilyl triflate (TBDMSOTf) were used and **2 a** was isolated in similar yields (93 % and 88 %, respectively; Table 1, Entries 2 and 3). The combination of hypervalent iodine reagents and boron trifluoride diethyl etherate, BF_3_⋅Et_2_O, has already been studied,^9^ and, here, a weak activation was observed, and rearranged product **2 a** was isolated in only 18 % yield together with a different rearranged product **3 a**, which was obtained in 67 % yield (Table 1, Entry 3). Rearrangement products such as **3 a** have already been reported using hypervalent iodine reagents.2b, 10 Rearranged cyclic product **2 a** was not observed by using a Brønsted acid such as camphorsulfonic acid and only product **3 a** was formed (Table 1, Entry 5). Koser’s reagent, PhI(OH)OTs, also led mainly to compound **3 a** (Table 1, Entry 6).

**Table 1:** Different activators in the rearrangement of 1 a[a]

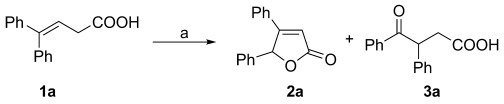			

Entry	Activator	2 aYield [%]	3 aYield [%]
1	–	0	0
2	TMSOTf	93	0
3	TBDMSOTf	88	0
4	BF_3_⋅OEt_2_	18	67
5[b]	camphorsulfonic acid	0	92
6[c]	TsOH⋅H_2_O	8	64

[a] Reagents and conditions: a) PhI(OCOCF_3_)_2_ (1 equiv), activator (2 equiv), MeCN, RT, 30 min;

[b] When the reaction was performed by using substrate (*E*)-**1 f**, rearranged product **3 f** was obtained in 89 % yield.

[c] Koser’s reagent Ph(OH)OTs was used for this reaction. In addition to **2 a** and **3 a**, compound **4 a** was observed in 8 % yield.

**Scheme 2:**
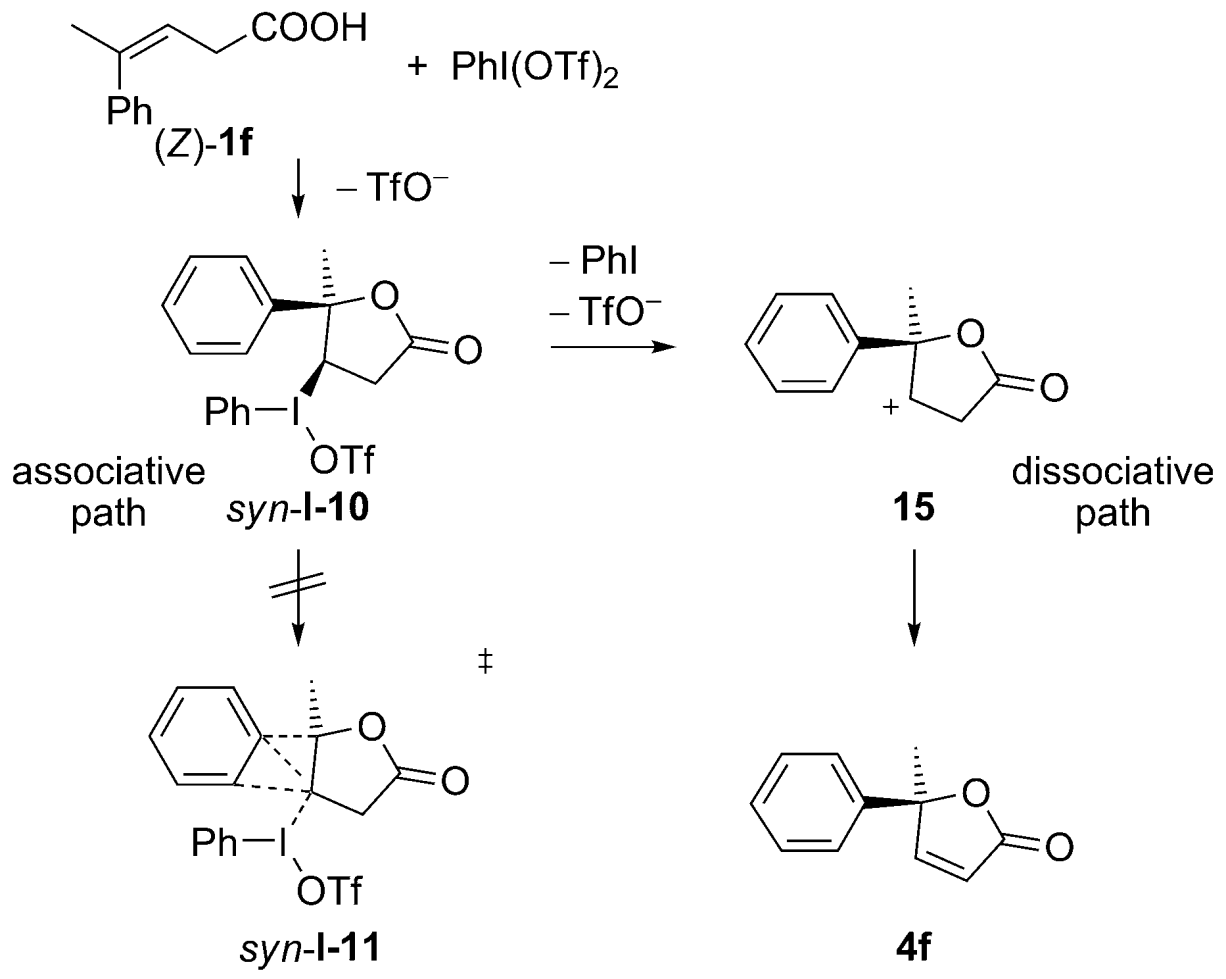
Schematic representation of the dissociative mechanism for the elimination in the formation of 4 f.

With these optimized reaction conditions, a series of 4-arylbut-3-enoic acids **1** were rearranged to yield 4-arylfuranones **2** in 78–95 % yield. The oxidative rearrangement proceeds smoothly with substrates **1** having both mono- and disubstitution at position C-4, as shown in Table 2. The rearranged products **2** were obtained in good yields with substrates **1** bearing two aryl substituents (Table 2, Entries 1–3). Substrate **1 c** containing a 1:1 mixture of *E* and *Z* isomers lead to a mixture of two rearranged products **2 c** in a 1:1 ratio in overall 88 % yield (Table 2, Entry 3). The α-methylated substrates **1 d** and **1 g** reacted similarly well as the unsubstituted substrates to product **2 d** and **2 g** in 81 % and 87 % yield, respectively (Table 2, Entries 4 and 8). (*E*)-4-Phenylbut-3-enoic acid (**1 e**) gave furanone **2 e** in 78 % yield (Table 2, Entry 5). Furthermore, phenyl and methyl group-bearing substrates (*E*)-**1 f** and (*Z*)-**1 f** were prepared. Compound (*E*)-**1 f** was photochemically isomerized to (*Z*)-**1 f**.^11^ Acid (*E*)-**1 f** was rearranged to compound **2 f** in 89 % yield (Table 2, Entry 6), whereas (*Z*)-**1 f** exclusively formed the cyclization product **4 f** in 81 % yield without rearrangement (Table 2, Entry 7). A mechanistic rationalization of the reaction pathway of (*Z*)-**1 f** is shown in Scheme 2. Compounds **4** as products from an addition/elimination sequence can also be obtained through a selenolactonization of acids **1** followed by an elimination induced by hypervalent iodine compounds, as we have shown previously.^10^, ^12^

**Table 2:** Oxidative rearrangements of compounds 1 a–h[a]

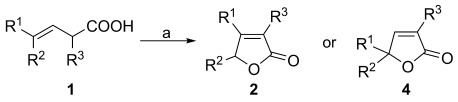			

Entry	Substrate1	Yield of2[%]	Yield of4[%]
1	**1 a**: R^1^=R^2^=Ph, R^3^=H	93	–
2	**1 b**: R^1^=R^2^=4-Cl-C_6_H_4_, R^3^=H	95	–
3	**1 c**: R^1^=Ph, R^2^=4-Br-C_6_H_4_, R^3^=H	88	–
4[b]	**1 d**: R^1^=R^2^=Ph, R^3^=Me	81	–
5	**1 e**: R^1^=Ph, R^2^=R^3^=H	78	–
6	(*E*)-**1 f**: R^1^=Ph, R^2^=Me, R^3^=H	89	–
7	(*Z*)-**1 f**: R^1^=Me, R^2^=Ph, R^3^=H	trace	81
8	**1 g**: R^1^=Ph, R^2^=R^3^=Me	87	–
9[c]	**1 h**: R^1^=Me, R^2^=R^3^=H	–	73

[a] *Reagents and conditions*: a) PhI(OCOCF_3_)_2_ (1 equiv), TMSOTf (2 equiv), MeCN, RT, 30 min;

[b] The use of PhI(OH)OTs instead of PhI(OCOCF_3_)_2_ yielded **2 d** in 76 % yield together with **4 d** in 16 % yield.

[c] Reaction time was 5 h.

Finally, (*E*)-pent-3-enoic acid (**1 h**) with an aliphatic substituent R^1^ was employed as starting material, and product **4 h** was isolated exclusively (Table 2, Entry 9). This result clearly indicates that the presence of an aryl functionality at the C-4 position in substrates **1** is essential to perform these rearrangements.

**Scheme 3:**
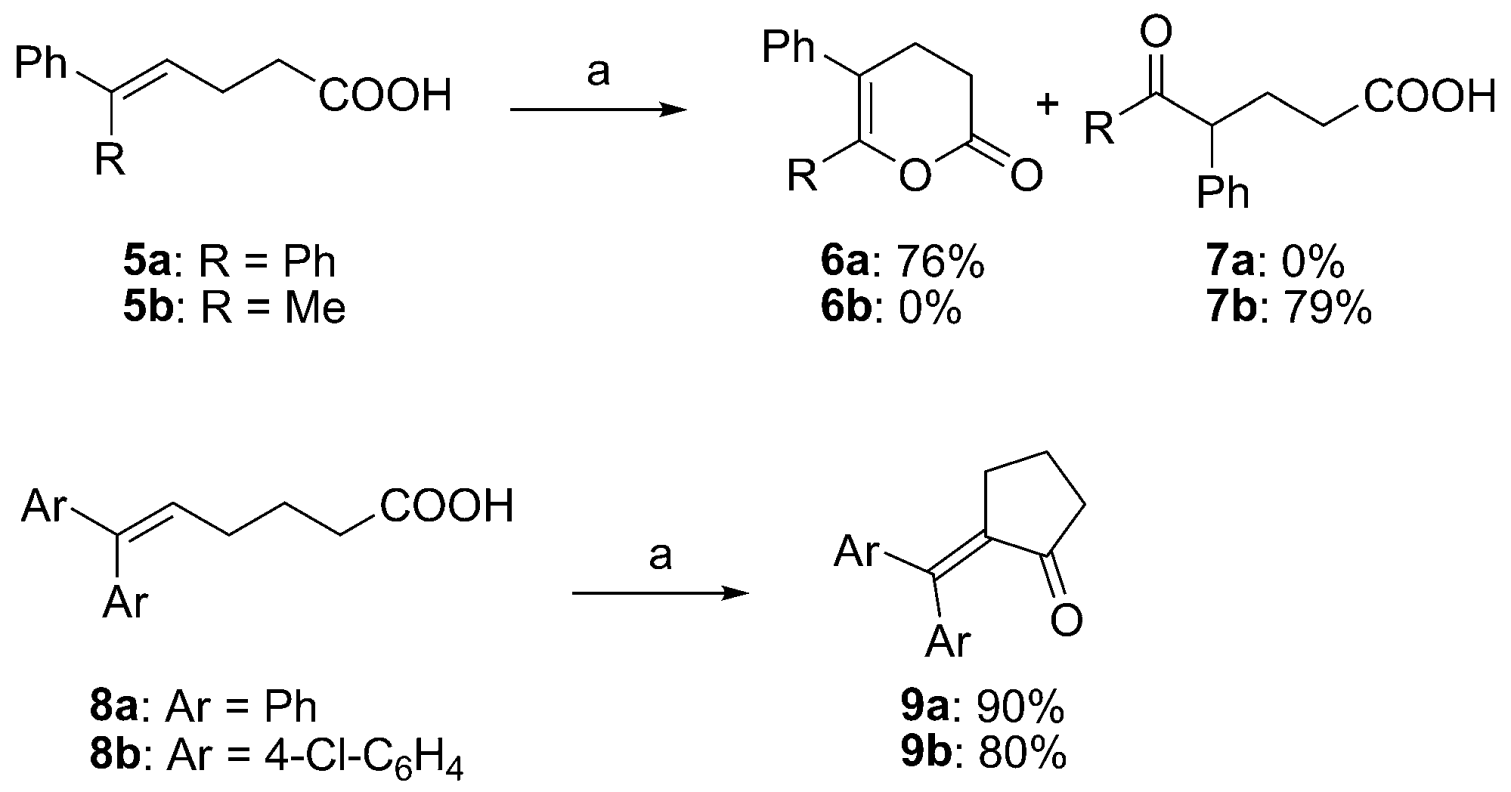
Reactions of unsaturated carboxylic acids 5 and 8. *Reagents and conditions*: a) PhI(OCOCF_3_)_2_ (1 equiv), TMSOTf (2 equiv), MeCN, RT, 30 min.

Even other ring sizes are accessible using substrate **5 a**, which is prepared through a Wittig reaction.^13^ Rearranged product **6 a** was isolated in 76 % yield. (*E*)-5-Phenylhex-4-enoic acid **5 b** was synthesized from cyclopropyl methyl ketone (see Supporting Information). Surprisingly, only the acyclic rearranged product **7 b** was isolated in 79 % yield and no cyclization occurred (Scheme 3). The hexenoic acid **8 a** also cyclized, and compound **9 a** was confirmed by X-ray analysis to be the cyclization product.^14^ The same product **9** is also obtained in a slower reaction by treatment of **8** with triflic acid only, probably through activation of the carboxylic acid and cyclization followed by elimination.

Asymmetric oxidative rearrangements of 4-arylbutenoic acids **1** could probably be achieved by using chiral hypervalent iodine reagents. Lactate-based chiral hypervalent iodine reagents have been synthesized by Fujita et al.^15^ and employed as chiral reagent in highly enantioselective spirolactonizations^16^ and oxyamination reactions.5b, 17 When using **1 a** or (*E*)-**1 f** in such transformations, the products **2 a** and **2 f** were obtained only as racemates in 28 % and 73 % yield, respectively (see Supporting Information).

### Calculations

**Scheme 4:**
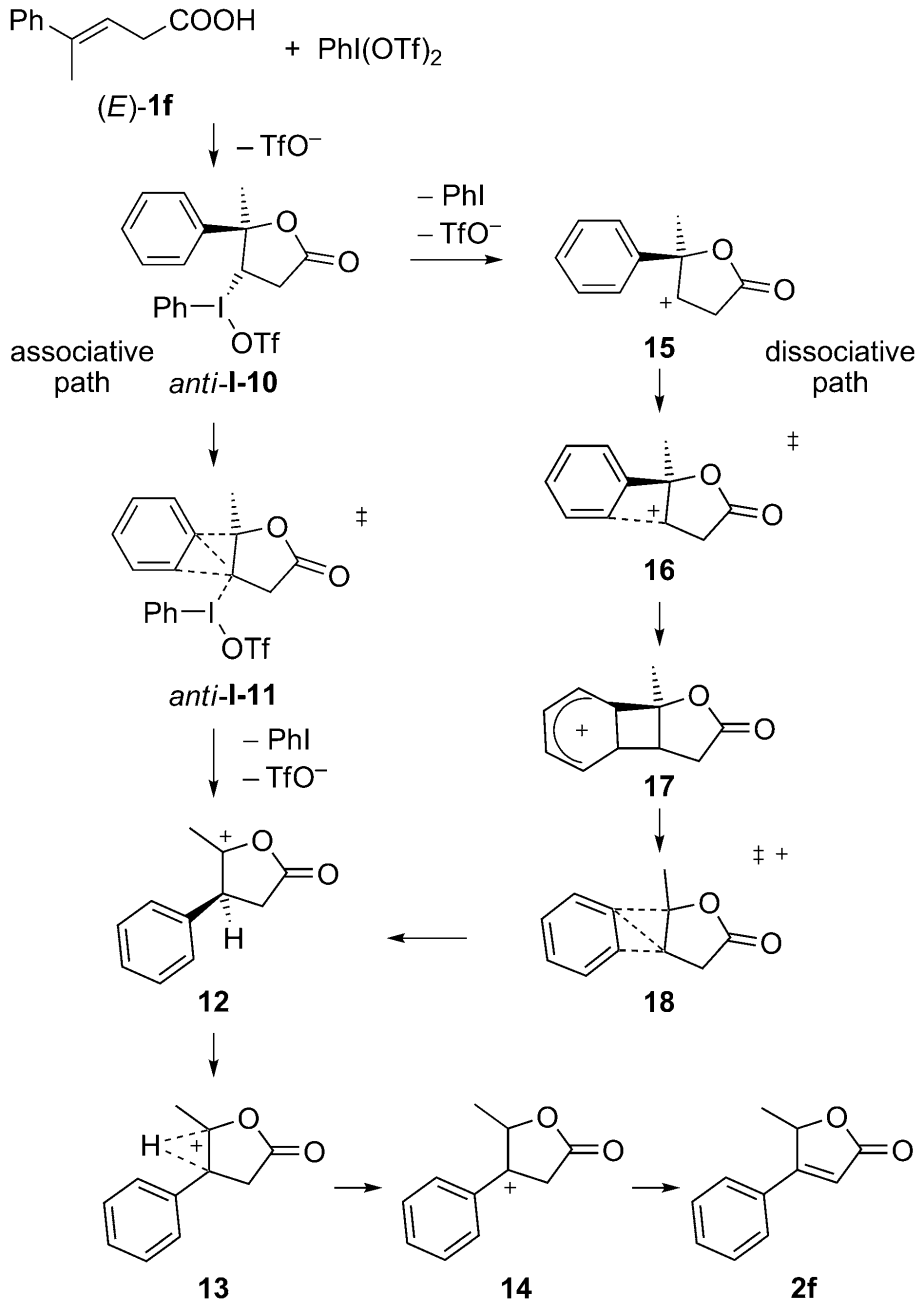
Schematic representation of an associative versus dissociative mechanism for the rearrangement–elimination sequence for the formation of 2 f.

The exclusive formation of product **2 f** when substrate (*E*)-**1 f** is reacted with hypervalent iodine reagents in contrast to the formation of **4 f** upon treating (*E*)-**1 f** with a combination of (SePh)_2_ and PhI(OCOCF_3_)_2_ can be explained by either a dissociative stepwise mechanism or by a concerted one-step mechanism (Scheme 4). In case of a dissociation of the complex *anti*-**I-10**, the relative stability of the ions **15** and **12** might govern the observed product distribution. In a different scenario, the relative stability of the precursor *anti*-**I-10** towards the dissociation or, in other words, the relative barriers of dissociation versus elimination could be an explanation. Calculations were conducted to analyze the first case scenario of the dissociation model and the possible one-step mechanism in more detail.

**Figure 1:**
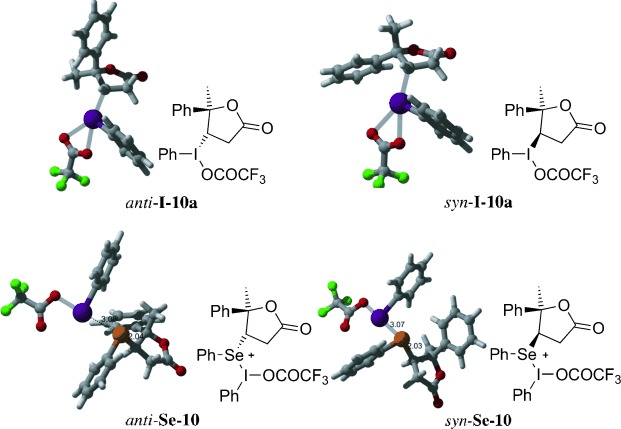
Iodo- and selenocyclization intermediates.

**Figure 2:**
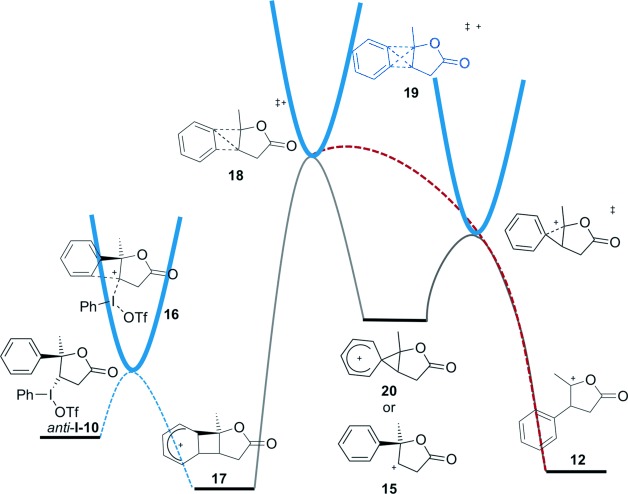
Energy profile at B3LYP/6-31+G** level of theory for phenyl migration.

**Scheme 5:**
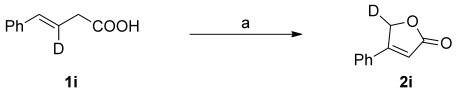
Cyclization of deuterated compound 1 i to 2 i. *Reagents and conditions*: a) PhI(OCOCF_3_)_2_ (1 equiv), TMSOTf (2 equiv), MeCN, RT, 30 min, 58 %.

The dissociation of either **I-10** or the corresponding reaction products with a selenium electrophile, **Se-10** (Figure 1), should in principle result in the formation of the secondary carbenium ion **15**. However, the nature of **15** is fleeting: It is neither a ground state nor a transition state (TS). Instead, optimizations started off from **15**, a classical carbenium ion, or from **20**, a nonclassical carbenium-ion structure, and led either to the resonance-stabilized cation **17** or to the rearranged cation **12**, as shown in Figure 2. The latter is a precursor to the elimination product **2 f** that is observed as the main product of the reaction (*E*)-**1 f** with hypervalent iodine reagents. The transition state **18** that connects **17** and **12** shows interesting features. The reorganization of the bonds would enable the formation of **14** and **15**, the very definition of a “merged transition state”. This bonding characteristic of the TS is thought to be indicative for bifurcating potential energy surface (PES).^18^ The intrinsic reaction coordinate (IRC) was calculated starting from **19** to map out the minimum energy pathway (MEP). The MEP occurs through a shallow plateau that corresponds to the nonclassical carbenium ion **20** and then proceeds to the rearranged ion **12**. Further studies including molecular dynamic simulations are currently conducted to investigate the driving force for the formation of **14** over **15** and to clarify if the potential bifurcation exists. IRC calculations confirm that structure **15** is neither a saddle point of first order (transition state) nor a ground state. A possible reaction path “downhill” from intermediate **17** occurs through a merged transition state **19** that incorporates features necessary for the bond reorganization for **14** and **15**. The hydride shift proposed in the transformation from **12** to **14** was confirmed by the synthesis and reaction of the deuterated compound **1 i** to the cyclized derivative **2 i**, as shown in Scheme 5.

Alternatively, the dissociation of adducts **I-10** or **Se-10** could occur concertedly with the rearrangement of the phenyl group (one-step mechanism). In this case, rearrangement is only expected to occur in case the migrating phenyl group is orientated *anti* to the leaving group (Scheme 4). The steric impact of a *syn* configuration should prevent a migration of the phenyl group (Figure 1). Control experiments employing (*Z*)-**1 f** instead of (*E*)-**1 f** using hypervalent iodine compounds indeed resulted in the exclusive formation of **4 f**, hence no rearrangement occurred.

The different chemoselectivities of PhI(OCOCF_3_)_2_ compared with the combination of (SePh)_2_/PhI(OCOCF_3_)_2_ and the dependence of product formation on the double bond configuration of starting material **1 f** strongly indicate a concerted mechanism of elimination/rearrangement in case of **I-10**. The complete absence of rearrangement in case of the selenium compounds suggests that the barrier for dissociation is higher compared with the barrier for the elimination of the β-proton. This effect can be rationalized by the different bond strengths: Carbon–selenium bonds are shorter than carbon–iodine bonds, and equally the bond-dissociation energy (BDE) for C–Se is higher than the BDE for C–I.

**Figure 3:**
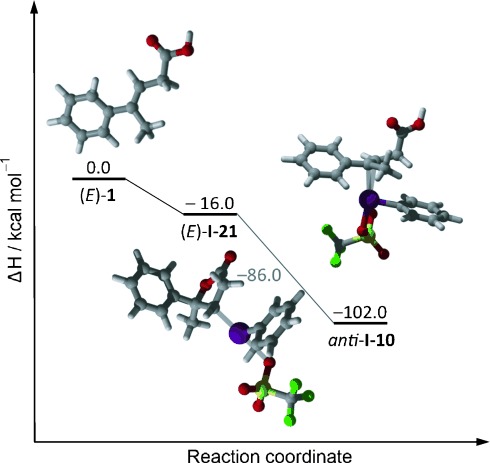
Energy profile at B3LYP/6-31+G** and LANLD2 VZ level of theory with effective core potentials (ECPs) on iodine.

The first reaction steps of PhI(OTf)_2_ with unsaturated acid (*E*)-**1 f** are all exothermic (Figure 3). The formation of iodonium ion (*E*)-**I-21** is favored by 16 kcal mol^−1^ and the subsequent cyclization by 86 kcal mol^−1^. Comparable results are obtained with the double bond isomer (*Z*)-**1 f** (see Supporting Information).

Interestingly, the labile ligands of the iodine compound play a crucial role for its reactivity. The experimental observation that PhI(OCOCF_3_)_2_ alone is not reactive in this reaction is possibly explained by the predicted strongly endothermic first step of the electrophilic addition of the iodine moiety to the double bond.

**Scheme 6:**
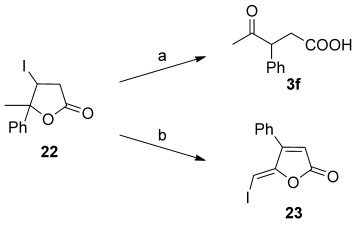
Oxidation of iodolactone 22. *Reagents and conditions*: a) Oxone (1.5 equiv), TFA, CHCl_3_, 2 h, 86 %; b) PhI(OCOCF_3_)_2_ (1 equiv), TMSOTf (2 equiv), MeCN, RT, 12 h, 83 %.

In a further attempt, the reaction was performed in two steps. Acid (*E*)-**1 f** was cyclized with iodine monochloride to give the iodolactone **22** in 78 % yield. Unsuccessful reagents to oxidize the iodine atom in **22** include peracetic acid, sodium perborate and PhI(OCOCF_3_)_2_. Oxidation with oxone led to compound **3 f** in 86 % yield (Scheme 6), whereas treatment with PhI(OTf)_2_ resulted in the formation of **23** probably via similar intermediates as described above.^19^ Compound **2 f** was, however, not detected. No further information on the reaction mechanism could be obtained by these experiments.

**Scheme 7:**
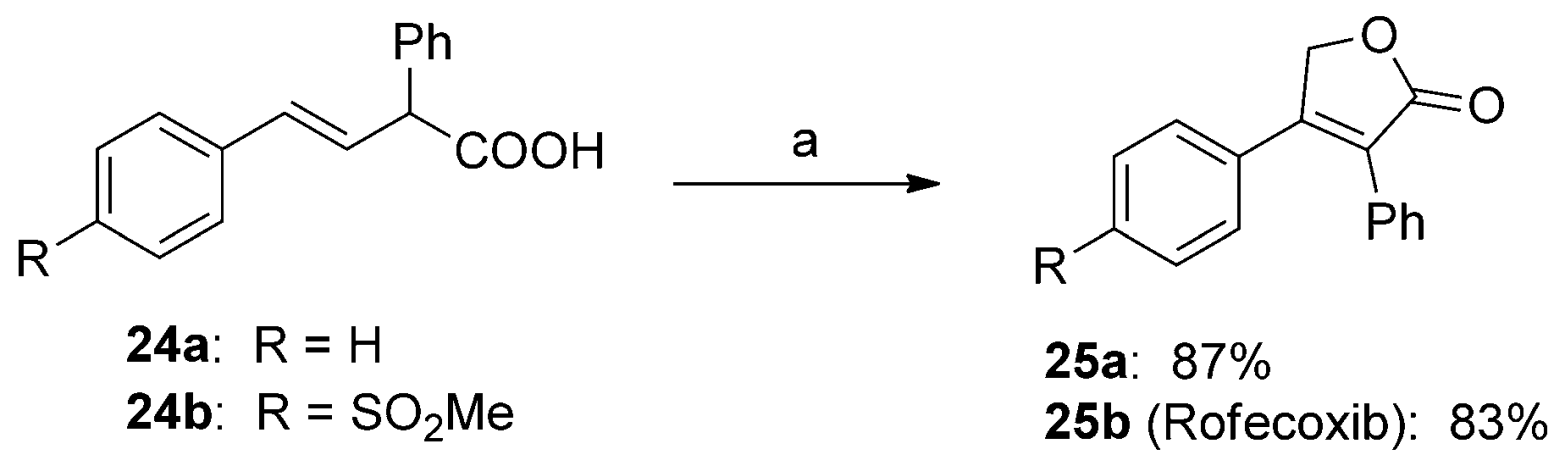
Synthesis of Rofecoxib 25 b. *Reagents and conditions*: a) PhI(OCOCF_3_)_2_ (1 equiv), TMSOTf (2 equiv), MeCN, RT, 30 min.

Furanone scaffolds are basic structural motifs found in various naturally occurring biologically active compounds.^20^ Also, synthetic furanones with interesting pharmacological and pharmacokinetic properties have been reported.^21^ For example, furanone derivative **25 b** is known as a selective cyclooxygenase-2 (COX-2) inhibitor.^22^ Rofecoxib (**25 b**) was synthesized in a straightforward manner from acid **24 b** in 83 % yield, and unsubstituted acid **24 a** led to the cyclized and rearranged product **25 a**, also a bioactive compound, in 87 % yield (Scheme 7).^23^

## Conclusion

In conclusion, we have developed an efficient novel oxidative rearrangement mediated by hypervalent iodine reagents of 4-arylbut-3-enoic acids leading to highly substituted furanone derivatives in good to excellent yields under mild reaction conditions. We have studied mechanistic details for these transformations by calculations, and a deuterium-labelled compound was also synthesized to provide further insight. Our approach to prepare functionalized furanone architectures is very simple, economical and does not require any specialized reagents or catalysts. We have used this methodology for a successful synthesis of the cyclooxygenase-2 (COX-2) inhibitor, Rofecoxib. Further investigations of this approach to the synthesis of furanones are currently in progress.

## Experimental Section

**General procedure for cyclizations**: A solution of the γ,δ-unsaturated acid **1** (0.2 mmol) in acetonitrile (2 mL) was added to a solution of [bis(trifluoroacetoxy)iodo]benzene (86 mg, 0.2 mmol) and TMSOTf (0.072 mL, 0.4 mmol) in acetonitrile (2 mL) at RT. The reaction was monitored by TLC and is usually complete within 30 min. Acetonitrile was removed in vacuo, water (5 mL) was added, and the mixture extracted with CH_2_Cl_2_ (3×5 mL). The combined organic layers were dried over MgSO_4_, filtered and concentrated in vacuo. The product mixture was purified by flash chromatography using CH_2_Cl_2_ as eluent.

**Calculations**: DFT calculations were performed using the Gaussian 03 suite of programs.^24^ Reactants, transition states (TSs), intermediates, and products were fully optimized with the hybrid density functional B3LYP^25^ using the Pople-type 6-31+G(d,p) basis set^26^ for all compounds without iodine and a composite basis set consisting of 6-31+G(d,p) for C, H, Se, O, F and LANL2DZ for I incorporating a relativistic pseudopotential (effective core potential, ECP) that largely accounts for scalar relativistic effects in iodine. Solvent effects of acetonitrile were approximated using the SCRF approach employing PCM as implemented in Gaussian 03.^27^ Energies in solution were obtained as single point calculations on gas-phase geometries (SCRF=PCM//B3LYP/6-31+G**). All TSs have been characterized by one imaginary frequency (first-order saddle points) on the potential energy surface (PES).^28^ To determine minimum energy pathways (MEPs), intrinsic reaction coordinate (IRC) analyses were performed, in order to confirm that a specific TS connects the different local minima.^29^ In addition, the imaginary frequencies were visually analyzed and proven to be the correct eigen vibration by animating it in Gabedit.^30^ Vibrational frequencies and zero-point vibrational energies (ZPE) were determined within the harmonic oscillator approximation, at the same level of theory as that for geometries. All energies reported in this paper are free energies or enthalpies in kcal mol^−1^ at 298 K and 1 atm if not stated otherwise. Frequencies remained unscaled.

## Supplementary material

Detailed facts of importance to specialist readers are published as ”Supporting Information”. Such documents are peer-reviewed, but not copy-edited or typeset. They are made available as submitted by the authors.
