# Supplementary material for: Facile Oxidative Rearrangements Using Hypervalent Iodine Reagents
Source: ChemistryOpen. 2012 Nov 6;1(6):245–50. doi: 10.1002/open.201200037 (PMC3922481; doi:10.1002/open.201200037)
Supplement: Supplementary file 1 [file open0001-0245-SD1.pdf]

ChemistryOPEN

Supporting Information

© 2012 The Authors. Published by Wiley-VCH Verlag GmbH & Co. KGaA, Weinheim

Facile Oxidative Rearrangements Using Hypervalent Iodine Reagents

Fateh V. Singh, Julia Rehbein, and Thomas Wirth*[a]

open_201200037_sm_miscellaneous_information.pdf

General:

Melting points were obtained in open capillary tubes using Gallenkamp apparatus. NMR spectra were recorded on Bruker DPX 250, Bruker DPX 400, Bruker DPX 500, or Oxford 300. ^{1}H NMR spectra were measured at 250, 300, 400 and 500 MHz. ^{13}C NMR spectra were measured at 63, 100 and 126 MHz, using $CDCl_3$, CD_3CN or DMSO-d_6 as a solvent and internal reference. Coupling constants *J* are given in Hz. Multiplicity as follows: s = singulet, d = doublet, t = triplet, q = quartet, m = multiplet, b = broad signal. All starting materials were purchased from commercial suppliers and used without further purification. Mass spectra (*m*/*z*) and HRMS were recorded under the conditions of electron impact (EI) and electrospray (ES) and chemical ionization (CI). Infrared spectra were recorded on a JASCO FT/IR-660 Plus spectrometer. GC-MS measurements were done on a Perkin Elmer Turbomass. Melting points were taken from Gallenkamp heater and are uncorrected. Flash chromatography was carried out using Fisher silica gel (35-70 mesh) or using a Biotage Isolera system. Dry solvents such as THF, acetonitrile and diethyl ether were used from a solvent purification system while anhydrous DMSO was purchased from Sigma Aldrich. All other purchased chemicals were used without further purification. The hypervalent iodine reagents (diacetoxyiodo)benzene,[1] (bis[trifluoroacetoxy]iodo)benzene,[2] Koser reagent,[1] 2-iodoxybenzoic acid (IBX),[3] iodosylbenzene[4] and μ-oxo-bridged phenyliodine trifluoroacetate[5] were synthesized in the laboratory. Lactate-based chiral hypervalent iodine reagents have been synthesized according to a procdure by Fujita *et al*,[6] Reactions requiring the exclusion of air were carried out under an atmosphere of argon or nitrogen in oven-dried glassware using usual Schlenk technique.

Table S1. Different hypervalent iodine reagents for the oxidative rearrangement of compound **1a**.

Entry	Reagent	Reaction Time [h]	**2a** Yield [%][a]
1	$PhI(OCOCF_3)_2$	0.5	93
2	$PhI(OAc)_2$	1	85
3	PhI(OH)OTs	0.5	91
4	PhIO	15	32
5	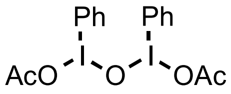	1	84[b]
6	IBX	15	21

[a] Reactions performed in MeCN at r.t. with 2 eq. TMSOTf. [b] Reaction performed at 0 ºC; reaction at r.t. for 0.25 h: 73% yield; reaction at –78 ºC for 24 h: 21% yield.

Syntheses and Reactions:

General procedure for the synthesis of 4-arylbut-4-enoic acids (1a, 1e and 1f):[7]

The mixture of α-substituted phenyl acetaldehydes (1 mmol), malonic acid (104 mg, 1.0 mmol) and pyridine (0.082 mL, 1.0 mmol) was stirred with heating at 95 °C for 12-72 h. The reaction was monitored by TLC. The reaction with α-methyl phenyl acetaldehyde was quite slow and was complete after 72 h while the reaction with phenyl acetaldehyde and 1,1-diphenyl acetaldehyde were complete after 12 h. After completion of the reaction, the reaction mixture was cooled to room temperature and dissolved in Et_2O (8 mL) followed by extraction with 2 M aq. NaOH solution (3 x 5 mL). The organic layer was discarded and the aqueous extract was acidified by dropwise addition of concentrated sulfuric acid up to pH 1-2. The acidified aqueous layer was extracted with Et_2O (3 x 5 mL). The combined organic extracts were washed with saturated brine, dried ($MgSO_4$), filtered and concentrated in vacuum. The crude product mixtures were purified by column chromatography by using methanol / dichloromethane (1:100) as eluent.

4,4-Diphenylbut-3-enoic acid (1a):[8]

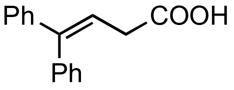

1a

Light yellow solid; m.p.: 110–112 °C; yield: 77% (183 mg, 0.77 mmol); ^{1}H NMR (400 MHz, $CDCl_3$): δ = 3.25 (d, *J* = 7.2 Hz, 2H, CH_2), 6.27 (t, *J* = 7.4 Hz, 1H, CH), 7.21-7.23 (m, 2H, ArH), 7.28-7.30 (m, 5H, ArH), 7.37-7.45 (m, 3H, ArH) ppm; ^{13}C NMR (100 MHz, $CDCl_3$): δ = 35.1, 119.5, 127.4 (2C), 127.6 (2C), 128.2 (2C), 128.5 (2C), 129.7 (2C), 139.1, 141.8, 145.4, 178.0 ppm.

Synthesis of 1b and 1c:

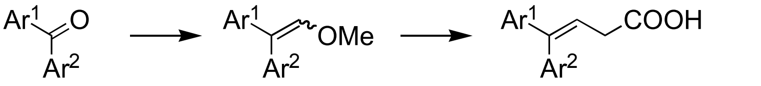

S1b **S2b** **1b** (Ar^1=Ar^2=4-Cl-C_6H_4)
S1c **S2c** **1c** (Ar^1=4-Br-C_6H_4, Ar^2=Ph)

General procedure for the synthesis of 1-methoxy-2,2-diarylethenes (S2b, S2c): (Methoxymethyl)triphenylphosphonium chloride (684 mg, 2.0 mmol) was suspended in THF (10 mL). The colorless suspension was cooled to 0 °C and *n*-BuLi (2.5 M in hexane, 0.8 mL, 2.0 mmol) was added dropwise and the resulting bright orange solution was stirred at 0 °C for 30 min. A solution of benzophenones **S1a** or **S2b** (2.2 mmol) in THF (5 mL) was added dropwise and the reaction mixture was stirred overnight at room temperature. The reaction was monitored by thin layer chromatography. After completion of the reaction, the solvent was removed under vacuum and reaction mixture was poured into ice-cold water (10 mL). After the acidification with 1 M HCl, the reaction mixture was extracted with dichloromethane (3 x 10 mL). The organic layer was dried over $MgSO_4$, filtered and concentrated under reduced pressure. The crude product was purified by column chromatography on silica gel by using dichloromethane / hexane (1:9) as eluent.

4,4'-(2-Methoxyethene-1,1-diyl)bis(chlorobenzene) (S2b):

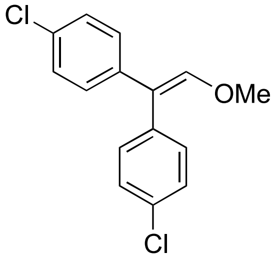

S2b

Colorless solid; m.p.: 128–130 °C; yield: 79% (441 mg, 1.58 mmol); ^{1}H NMR (400 MHz, $CDCl_3$): δ = 3.83 (s, 3H, OMe), 6.50 (s, 1H, CH), 7.17 (td, J_1 = 2.4 & J_2 = 8.4 Hz, 2H, ArH), 7.30 (td, J_1 = 2.0, J_2 = 8.4 Hz, 2H, ArH), 7.35 (d, J = 2.4 Hz, 4H, ArH) ppm; ^{13}C NMR (125 MHz, $CDCl_3$): δ = 61.3, 118.8, 128.7 (2C), 129.0 (2C), 129.9 (2C), 131.6 (2C), 132.8, 132.9, 136.1, 139.0, 147.4 ppm; IR (film): ν = 2931, 2833, 1729, 1660, 1631, 1590, 1399, 1232, 1091, 1013, 830 cm^{-1}; HRMS (APCI): m/z [M+1]$^{+}$ calcd for $C_{15}H_{13}Cl_2O$: 279.0338; found 279.0341.

(*E*/*Z*)-1-Bromo-4-(2-methoxy-1-phenylvinyl)benzene (S2c):

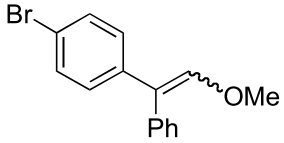

S2c

Colorless oil; yield: 74% (428 mg, 1.48 mmol); 1:1 mixture of isomers: ^{1}H NMR (400 MHz, $CDCl_3$): δ = 3.84 (s, 3H, OMe), 3.85 (s, 3H, OMe), 6.54 (s, 1H, CH), 6.55 (s, 1H, CH), 7.18 (td, J_1 = 2.0, J_2 = 7.2 Hz, 2H, ArH), 7.28-7.31 (m, 2H, ArH), 7.33-7.50 (m, 12H, ArH), 7.53 (td, J_1 = 2.0, J_2 = 7.2 Hz, 2H, ArH) ppm; ^{13}C NMR (125 MHz, $CDCl_3$): δ = 60.7, 119.5, 119.7, 120.3, 120.4, 126.8 (2C), 126.9, 128.2 (2C), 128.4 (2C), 128.5 (2C), 129.87 (2C), 129.91 (2C), 131.2 (2C), 131.5, 131.6 (2C), 136.7, 137.2, 139.6, 140.0, 146.6, 146.9 ppm; IR (film): ν = 2922, 1725, 1654, 1586, 1487, 1447, 1072, 1010, 830, 754 cm^{-1}; HRMS (APCI): m/z [M+1]$^{+}$ calcd for $C_{15}H_{14}BrO$: 289.0223; found 289.0227.

General procedure for the synthesis of 4,4-diarylbut-4-enoic acids (1b, 1c):

To a solution of 1-methoxy-2,2-diarylethene **S2b** or **S2c** (0.5 mmol) in 1,4-dioxane (5 mL), NaI (570 mg, 3.8 mmol) and aq. H_2SO_4 (2 M, 0.5 mL) were added. The mixture was stirred overnight at room temperature. The reaction was monitored by TLC. After the completion of reaction, 1,4-dioxane was removed under vacuum and water (5 mL) was added. A saturated aqueous solution of $Na_2S_2O_3$ was added dropwise until the violet color disappeared completely. The mixture was extracted with dichloromethane (3 x 5 mL). The combined organic layers were washed with aqueous $NaHCO_3$, brine and dried over $MgSO_4$, filtered and concentrated under reduced pressure. The crude product was used in the next step without any further purification. In the next step the crude product was mixed with malonic acid (52 mg, 0.5 mmol) and pyridine (0.041 mL, 0.5 mmol) and heated at 95 °C. The rapid formation of a yellow/orange homogeneous solution was accompanied by the evolution of gas and the mixture was heated overnight before the resultant orange solution was cooled to room temperature. The cooled mixture was dissolved in Et_2O (5 mL) and extracted with 2 M aqueous NaOH solution (3 x 5 mL). The organic layer was discarded and the aqueous extract was acidified by dropwise addition of concentrated sulfuric acid up to pH 1-2. The acidified aqueous layer was extracted with Et_2O (3 x 5 mL). The combined organic extracts were washed with saturated brine, dried ($MgSO_4$), filtered and concentrated under vacuum. Finally the crude products were purified by column chromatography by using methanol / dichloromethane (1:100) as eluent.

4,4-Bis(4-chlorophenyl)but-3-enoic acid (1b):

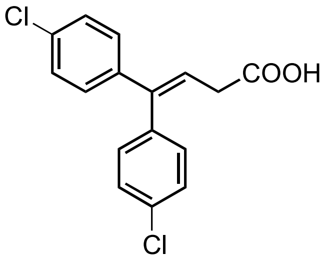

1b

Yellow solid; m.p.: 106–108 °C; yield: 58% (89 mg, 0.29 mmol); ^{1}H NMR (400 MHz, $CDCl_3$): δ = 3.22 (d, J = 7.6 Hz, 2H, CH_2), 6.24 (t, J = 7.4 Hz, 1H, CH), 7.13 (td, J_1 = 2.2 & J_2 = 8.4 Hz, 2H, ArH), 7.18 (td, J_1 = 2.2 & J_2 = 8.4 Hz, 2H, ArH), 7.26-7.29 (m,

2H, ArH), 7.40 (td, J_1 = 2.2 & J_2 = 8.4 Hz, 2H, ArH) ppm; ^{13}C NMR (100 MHz, $CDCl_3$): δ = 35.6, 120.8, 128.9 (2C), 129.1 (2C), 129.3 (2C), 131.5 (2C), 134.2, 134.3, 137.4, 140.2, 143.7, 178.3 ppm; IR (film): ν = 3428, 1702, 1498, 1406, 1043, 1015, 918, 743 cm^{-1}; HRMS (NSI): m/z $[M-COOH]^+$ calcd for $C_{16}H_{11}Cl_2$: 261.0237; found 261.0235.

(*E*/*Z*)-4-(4-bromophenyl)-4-phenylbut-3-enoic acid (1c):

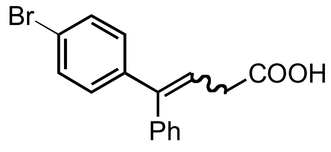

1c

Yellow oil; yield: 75% (118 mg, 0.335 mmol); ^{1}H NMR (400 MHz, $CDCl_3$): δ = 3.22 (d, *J* = 7.2 Hz, 2H, CH_2), 3.23 (d, *J* = 7.2 Hz, 2H, CH_2), 6.25 (t, *J* = 7.2 Hz, 1H, CH), 6.27 (t, *J* = 7.2 Hz, 1H, CH), 7.10 (d, *J* = 7.6 Hz, 2H, ArH), 7.15 (d, *J* = 8.4 Hz, 2H, ArH), 7.17-7.20 (m, 2H, ArH), 7.24-7.32 (m, 4H, ArH), 7.37-7.43 (m, 6H, ArH), 7.56 (d, *J* = 8.4 Hz, 2H, ArH) ppm; ^{13}C NMR (100 MHz, $CDCl_3$): δ = 35.2, 120.02, 120.04, 121.7, 121.8, 127.4 (2C), 127.8 (2C), 128.3 (2C), 128.6 (2C), 129.1 (2C), 129.7 (2C), 131.3 (2C), 131.5 (2C), 131.7 (2C), 137.9, 138.5, 140.7, 141.3, 144.29, 144.31, 178.0, 178.1 ppm (both isomers in 1:1 ratio); IR (film): ν = 3428, 2993, 2943, 1706, 1483, 1448, 1035 cm^{-1}; HRMS (NSI): m/z $[M-COOH]^+$ calcd for $C_{15}H_{12}Br$: 271.0128; found 271.0123.

Synthesis of 1d and 1g:

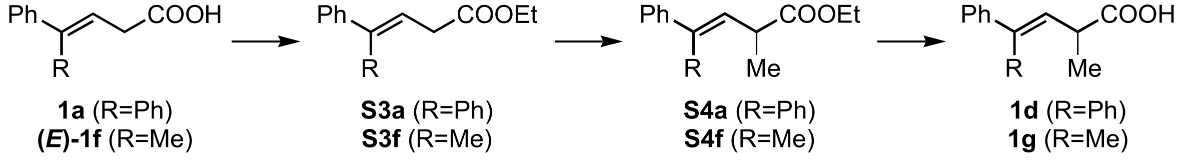

General procedure for the synthesis of ethyl 4,4-diphenylbut-3-enoate (S3a) and (*E*)-ethyl 4-phenylpent-3-enoate (S3f):

A mixture of acids **1a** or (***E***)-**1f** (1.0 mmol), thionyl chloride (0.2 mL, 3.0 mmol) and ethanol (3.50 mL, 60 mmol) was stirred at room temperature for 2-4 h. The reaction was monitored by TLC. After the completion of reaction, ethanol was removed under

vacuum, water (5 mL) was added and the mixture extracted with dichloromethane (3 x 5 mL). The combined organic extracts were washed with brine, dried over $MgSO_4$, filtered and concentrated under vacuum. The crude products were purified by column chromatography by using dichloromethane / hexane (1:1) as eluent.

Ethyl 4,4-diphenylbut-3-enoate (S3a):[9]

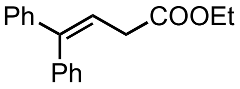

S3a

Colorless oil; yield: 97% (516 mg, 1.94 mmol); ^{1}H NMR (400 MHz, $CDCl_3$): δ = 1.26 (t, J = 7.2 Hz, 3H, Me), 3.15 (d, J = 7.6 Hz, 2H, CH_2), 4.15 (q, J = 7.2 Hz, 1H, CH_2), 6.26 (t, J = 7.2 Hz, 1H, CH), 7.17-7.20 (m, 2H, ArH), 7.25-7.27 (m, 5H, ArH), 7.32-7.40 (m, 3H, ArH) ppm; ^{13}C NMR (100 MHz, $CDCl_3$): δ = 14.7, 36.0, 61.2, 121.0, 127.8 (2C), 127.9 (2C), 128.6 (2C), 128.8 (2C), 130.2 (2C), 139.7, 142.4, 145.0, 172.4 ppm.

(*E*)-Ethyl 4-phenylpent-3-enoate (S3f):[10]

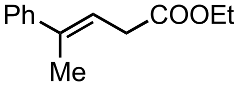

S3f

Colorless oil; yield: 99% (404 mg, 1.98 mmol); ^{1}H NMR (400 MHz, $CDCl_3$): δ = 1.18 (t, J = 7.2 Hz, 3H, Me), 1.96 (d, J = 1.2 Hz, 3H, Me), 3.15 (dd, J_1 = 0.8, J_2 = 7.2 Hz, 2H, CH_2), 4.08 (q, J = 7.2 Hz, 1H, CH_2), 5.87 (qt, J_1 = 1.2, J_2 = 7.2 Hz, 1H, CH), 7.14 (tt, J_1 = 1.2, J_2 = 7.2 Hz, 1H, ArH), 7.20-7.24 (m, 2H, ArH), 7.30-7.33 (m, 2H, ArH) ppm; ^{13}C NMR (100 MHz, $CDCl_3$): δ = 14.7, 16.7, 35.0, 61.2, 119.8, 126.3 (2C), 127.5, 128.7 (2C), 138.4, 143.6, 172.3 ppm.

General procedure for the synthesis of ethyl 2-methyl-4,4-diphenylbut-3-enoate (S4a) and (*E*)-ethyl 2-methyl-4-phenylpent-3-enoate (S4f):

The solution of β,γ–unsaturated esters **S3a** or **S3f** (1.0 mmol) in THF was added to the solution of *t*-BuOK (134 mg, 1.2 mmol) in dry THF (5 mL) at –78°C under argon atmosphere and methyl iodide (0.09 mL, 1.5 mmol) was added after 5 min. The reaction was allowed to warm up to room temperature and stirring was continued for 6 h. The

reaction was monitored by TLC. After completion of the reaction, THF was removed under vacuum and water (5.0 mL) was added. The reaction mixture was extracted with dichloromethane (3 x 5 mL). The combined organic layers were washed with brine, dried over $MgSO_4$ and evaporated to give a crude product. Finally, the crude product was purified by column chromatography using dichloromethane / hexane (1:1) as eluent.

Ethyl 2-methyl-4,4-diphenylbut-3-enoate (S4a):

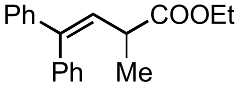

S4a

Colorless oil; yield: 93% (260 mg, 0.93 mmol); ^{1}H NMR (400 MHz, $CDCl_3$): δ = 1.26 (t, *J* = 7.2 Hz, 3H, Me), 1.27 (t, *J* = 6.8 Hz, 3H, Me), 3.27 (dq, J_1 = 6.8 & J_2 = 10.4 Hz, 1H, CH), 4.14 (q, *J* = 7.2 Hz, 1H, CH_2), 6.13 (d, *J* = 10.4 Hz, 1H, CH), 7.20-7.27 (m, 7H, ArH), 7.34-7.34 (m, 3H, ArH) ppm; ^{13}C NMR (100 MHz, $CDCl_3$): δ = 14.7, 19.0, 40.9, 61.1, 127.76, 127.81 (3C), 128.4, 128.6 (2C), 128.8 (2C), 130.2 (2C), 139.8, 142.3, 143.4, 175.4 ppm; IR (film): ν = 2967, 2933, 2875, 1736, 1731, 1258, 1083, 1057, 913, 748 cm^{-1}; HRMS (NSI): m/z $[M+1]^+$ calcd for $C_{19}H_{21}O_2$: 281.1536; found 281.1542.

(*E*)-Ethyl 2-methyl-4-phenylpent-3-enoate (S4f):

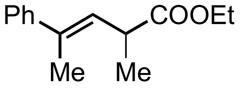

S4f

Colorless oil; yield: 84% (183 mg, 0.84 mmol); ^{1}H NMR (400 MHz, $CDCl_3$): δ = 1.16 (t, *J* = 7.2 Hz, 3H, Me), 1.23 (d, *J* = 7.2 Hz, 3H, Me), 1.99 (d, *J* = 1.2 Hz, 3H, Me), 3.42 (dq, J_1 = 6.8, J_2 = 9.2 Hz, 1H, CH), 4.05 (q, *J* = 7.2 Hz, 1H, CH_2), 5.70 (qd, J_1 = 1.2, J_2 = 9.2 Hz, 1H, CH), 7.14 (tt, J_1 = 1.2, J_2 = 7.2 Hz, 1H, ArH), 7.19-7.23 (m, 2H, ArH), 7.28-7.31 (m, 2H, ArH) ppm; ^{13}C NMR (100 MHz, $CDCl_3$): δ = 13.2, 15.1, 17.0, 38.7, 59.6, 124.8 (2C), 126.0, 126.1, 127.2 (2C), 135.6, 142.1, 173.9 ppm; IR (film): ν = 2977, 2933, 2874, 1737, 1484, 1452, 1406, 1371, 1320, 1257, 1189, 1143, 1126, 1083, 1057, 761 cm^{-1}; HRMS (APCI): m/z $[M+1]^+$ calcd for $C_{14}H_{19}O_2$: 219.1382; found 219.1385.

General procedure for the synthesis of 2-methyl-4,4-diphenylbut-3-enoic acid (1d) and (*E*)-2-methyl-4-phenylpent-3-enoic acid (1g):

The mixture of compound **S4a** or **S4f** (0.5 mmol) and LiOH•H_2O (63 mg, 1.5 mmol) in 6 mL of THF/MeOH/H_2O (4:1:l, v:v:v) was stirred at room temperature for 2 h. The reaction was monitored by TLC. After the completion of reaction, the mixture was acidified by dropwise addition of 1 M HCl solution up to pH 2-3. The reaction mixture was extracted with dichloromethane (3 x 5 mL). The combined organic layers were washed with brine, dried over anhydrous $MgSO_4$ and evaporated under reduced pressure. The crude product was used in cyclization reactions without any further purification.

2-Methyl-4,4-diphenylbut-3-enoic acid (1d):[11]

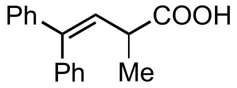

1d

Colorless solid; m.p.: 146 °C; yield: 99% (249 mg, 0.99 mmol); ^{1}H NMR (400 MHz, $CDCl_3$): δ = 1.23 (d, *J* = 6.8, Hz, 3H, Me), 3.25 (dq, J_1 = 6.8 & J_2 = 10.4 Hz, 1H, CH), 6.03 (d, *J* = 10.4 Hz, 1H, CH), 7.13-7.21 (m, 7H, ArH), 7.27-7.34 (m, 3H, ArH), 11.64 (brs, 1H, COOH) ppm; ^{13}C NMR (100 MHz, $CDCl_3$): δ = 18.8, 40.7, 127.4, 127.8 (2C), 127.9, 128.0, 128.6 (2C), 128.9 (2C), 130.2 (2C), 139.6, 142.1, 144.1, 181.9 ppm.

(*E*)-4-Phenylbut-3-enoic acid (1e):[12]

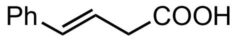

1e

Yellow solid; m.p.: 80–82 °C; yield: 71% (115 mg, 0.71 mmol); ^{1}H NMR (400 MHz, $CDCl_3$): δ = 3.34 (dd, J_1 = 1.4 & J_2 = 6.8 Hz, 2H, CH_2), 6.32 (td, J_1 = 6.8, J_2 = 16.0 Hz, 1H, CH), 6.56 (d, *J* = 16.0 Hz, 1H, CH), 7.25-7.30 (m, 1H, ArH), 7.33-7.37 (m, 2H, ArH), 7.40-7.43 (m, 2H, ArH) ppm; ^{13}C NMR (100 MHz, $CDCl_3$): δ = 38.1, 120.8, 126.4 (2C), 127.8, 128.6 (2C), 134.0, 136.6, 178.3 ppm.

(*E*)-4-Phenylpent-3-enoic acid [(*E*)-1f]:[13]

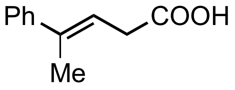

(*E*)-1f

Colorless solid; m.p.: 86–88 °C; yield: 73% (128 mg, 0.73 mmol); ^{1}H NMR (500 MHz, $CDCl_3$): δ = 2.10 (d, *J* = 1.0 Hz, 3H, Me), 3.34 (dd, J_1 = 1.0, J_2 = 7.0 Hz, 2H, CH_2), 5.97 (qt, J_1 = 1.2, J_2 = 7.0 Hz, 1H, CH), 7.28 (tt, J_1 = 1.2, J_2 = 7.2 Hz, 1H, ArH), 7.33-7.37 (m, 2H, ArH), 7.42-7.45 (m, 2H, ArH) ppm; ^{13}C NMR (125 MHz, $CDCl_3$): δ = 16.3, 34.1, 118.4, 125.8 (2C), 127.2, 128.3 (2C), 138.8, 143.0, 178.0 ppm.

(*Z*)-4-Phenylpent-3-enoic acid [(*Z*)-1f]:[14]

The solution of (*E*)-4-phenylpent-3-enoic acid [**(*E*)-1f**] (352 mg, 2.0 mmol) in heptane (60 mL) was placed in a pyrex photochemical reactor and irradiated for 8 h using a mercury medium pressure lamp (400 W). After 5 h, 75% of (*E*)-isomer was converted into the (*Z*)-isomer, but further 3 h irradiation did not change the ratio. The reaction was monitored by ^{1}H NMR. The solvent was removed under vacuum and the crude mixture was purified by a Biotage Isolera system (25 g silica cartridge) using ethyl acetate / hexane (1:9) with a 25 mL/min flow rate.

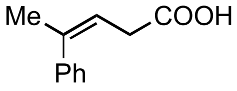

(*Z*)-1f

Colorless oil; yield: 71% (250 mg, 1.42 mmol); ^{1}H NMR (400 MHz, $CDCl_3$): δ = 2.13 (d, *J* = 1.2 Hz, 3H, Me), 3.10 (dd, J_1 = 1.2, J_2 = 7.2 Hz, 2H, CH_2), 5.68 (qt, J_1 = 1.2, J_2 = 7.2 Hz, 1H, CH), 7.21-7.23 (m, 2H, ArH), 7.28-7.33 (m, 1H, ArH), 7.37-7.41 (m, 2H, ArH) ppm; ^{13}C NMR (100 MHz, $CDCl_3$): δ = 26.1, 35.0, 118.0, 127.6, 128.2 (2C), 128.8 (2C), 141.3, 141.5, 179.5 ppm.

(*E*)-2-Methyl-4-phenylpent-3-enoic acid (1g):[15]

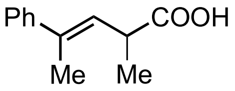

1g

Colorless oil; yield: 99% (94 mg, 0.595 mmol); ^{1}H NMR (400 MHz, $CDCl_3$): δ = 1.26 (d, J = 6.8 Hz, 3H, Me), 2.01 (d, J = 1.6 Hz, 3H, Me), 3.45 (dq, J_1 = 6.8 & J_2 = 9.2 Hz, 1H, CH), 5.68 (dd, J_1 = 1.2 & J_2 = 9.2 Hz, 1H, CH), 7.12-7.16 (m, 1H, ArH), 7.21 (t, J = 7.6 Hz, 2H, ArH), 7.28-7.31 (m, 2H, ArH) ppm; ^{13}C NMR (100 MHz, $CDCl_3$): δ = 15.2, 16.8, 38.5, 124.8 (2C), 125.1, 126.2, 127.2 (2C), 136.4, 141.9, 180.7 ppm.

Synthesis of 1i:

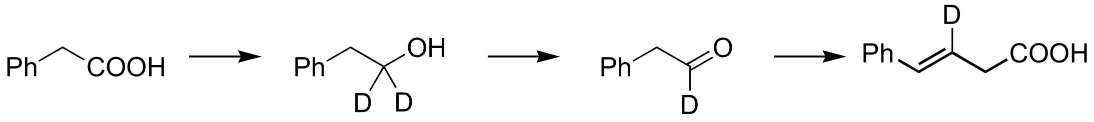

S5 **S6** **1i**

[1,1-$^{2}H_2$]-2-Phenylethanol (S5):[16]

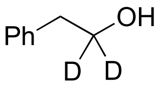

S5

$LiAlD_4$ (126 mg, 3.0 mmol) was suspended in dry diethyl ether (5 mL) and the solution of phenyl acetic acid (272 mg, 2.0 mmol) in diethyl ether (1 mL) was added dropwise at 0 ºC under argon atmosphere and then stirred for 15 min. The reaction was warmed up to room temperature and stirring was continued for 2 h at the same temperature. The reaction was monitored by TLC. After completion of the reaction, the reaction was quenched by dropwise addition water at 0 ºC and extracted with diethyl ether (3 x 5 mL). The combined organic layers were dried over $MgSO_4$, filtered and concentrated under reduced pressure. The crude product was purified by column chromatography by using dichloromethane / hexane (1:1) as eluent.

Colorless oil; yield: 52% (129 mg, 1.04 mmol); ^{1}H NMR (400 MHz, $CDCl_3$): δ = 2.55 (brs, 1H, OH), 2.75 (s, 2H, CH_2), 7.16 (t, J = 7.8 Hz, 3H, ArH), 7.22-7.26 (m, 2H, ArH) ppm; ^{13}C NMR (100 MHz, $CDCl_3$): δ = 37.9, 61.1 ($J_{C\text{-}D}$ = 21.8 Hz), 125.3, 127.5, 128.0, 137.6 ppm.

(*E*)-[3-^{2}H]- 4-Phenylbut-3-enoic acid (1i):

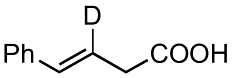

1i

To a solution of IBX (420 mg, 1.5 mmol) in DMSO (5 mL) [1,1-$^{2}H_2$]-2-phenylethanol **S5** (120 mg, 1.0 mmol) was added and stirred at room temperature for 20 h. The reaction was monitored by TLC. After the completion of reaction, water (5 mL) was added at 0 ^{o}C and a white precipitate was obtained. The precipitate was filtered off and discarded while the filtrate was extracted with dichloromethane (3 x 5 mL). The combined organic layers were washed with water, brine, dried over $MgSO_4$, filtered and concentrated under reduced pressure. The crude aldehyde **S6** was used in next step without any further purification. In the next step, the crude product was mixed with malonic acid (104 mg, 1.0 mmol) and pyridine (0.082 mL, 1.0 mmol) and heated at 95 °C for overnight. After the completion of reaction, the reaction mixture was cooled to room temperature, the cooled mixture was dissolved in Et_2O (5 mL) and extracted with 2 M aqueous NaOH solution (3 x 5 mL). The organic layer was discarded and the aqueous extract was acidified by dropwise addition of concentrated sulfuric acid up to pH 1-2. The acidified aqueous layer was extracted with Et_2O (3 x 5 mL). The combined organic extracts were washed with saturated brine, dried ($MgSO_4$), filtered and concentrated under vacuum. Finally the crude products were purified by column chromatography by using methanol / dichloromethane (1:100) as eluent.

Colorless solid; m.p.: 158–160 °C; yield: 30% (49 mg, 0.30 mmol); ^{1}H NMR (400 MHz, $CDCl_3$): δ = 3.23 (s, 2H, CH_2), 6.44 (s, 1H, CH), 7.25-7.30 (m, 1H, ArH), 7.14-7.18 (m, 1H, ArH), 7.22-7.26 (m, 2H, ArH), 7.29-7.32 (m, 2H, ArH) ppm; ^{13}C NMR (100 MHz, $CDCl_3$): δ = 38.4, 120.9 ($J_{C\text{-}D}$ = 23.9 Hz), 126.8 (2C), 128.1, 129.0 (2C), 134.3, 137.0, 178.5 ppm (*E* : *Z* = 84:16); IR (film): ν = 1924, 1733, 1615, 1589, 1484, 1438, 1256, 1060, 1002, 874, 785, 718 cm^{-1}; HRMS (NSI) m/z $[M]^{+}$ calcd for $C_{10}H_9DO_2$: 163.0744; found 163.0742.

General procedure for cyclization / rearrangements of unsaturated acids 1a-1i, 5a,b: A solution of the γ,δ-unsaturated acid **1** (0.2 mmol) in acetonitrile (2 mL) was added to a solution of [bis(trifluoroacetoxy)iodo]benzene (86 mg, 0.2 mmol) and TMSOTf (0.072 mL, 0.4 mmol) in acetonitrile (2 mL) at room temperature. The reaction was monitored by TLC and is usually complete within 30 min. Acetonitrile was removed in vacuuo, water (5 mL) was added and the mixture extracted with dichloromethane (3 x 5 mL). The combined organic layers were dried over $MgSO_4$,

filtered and concentrated in vacuo. The product mixture was purified by column chromatography using dichloromethane as eluent.

General procedure for asymmetric cyclization / rearrangements of unsaturated acids 1a and 1f: A solution of the γ,δ-unsaturated acid **1a** or **1f** (0.2 mmol) in dry dichloromethane (2 mL) was added to a solution of (2*R*,2'*R*)-2,2'-{[2-(diacetoxy)iodo-1,3-phenylene]bis(oxy)}bis(*N*-mesitylpropanamide) (100 mg, 0.2 mmol) and TMSOTf (0.072 mL, 0.4 mmol) in dichloromethane (2 mL) at –78 ºC and stirred for 48 h. The reaction was monitored by TLC. Dichloromethane was removed in vacuuo, water (5 mL) was added and the mixture extracted with CH_2Cl_2 (3 x 5 mL). The combined organic layers were dried over $MgSO_4$, filtered and concentrated in vacuo. The product mixture was purified by column chromatography using dichloromethane as eluent and product **2a** was isolated in 28% yield with 0% *ee* (HPLC conditions: Chiracel OD-H, hexane:2-propanol 9:1, 0.5 mL/min, R_f = 37.6 min, 41.2 min). Furthermore, the same reaction was performed with (*E*)-4-phenylpent-3-enoic acid **(*E*)-1f** using the same combination of (2*R*,2'*R*)-2,2'-{[2-(diacetoxy)iodo-1,3-phenylene]bis(oxy)}bis(*N*-mesitylpropanamide) (100 mg, 0.2 mmol) and TMSOTf (0.072 mL, 0.4 mmol) in acetonitrile for 2 h at room temperature and the rearranged product **2f** was isolated in 73% yield but as an almost racemic mixture (1% *ee*) (HPLC conditions: Chiracel OD-H, hexane:2-propanol 9:1, 0.5 mL/min, R_f = 36.0 min, 38.3 min).

4,5-Diphenylfuran-2(5*H*)-one (2a):[17]

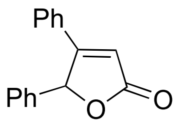

2a

Yellow solid; mp 152-154 °C; yield: 93% (44 mg, 0.186 mmol); ^{1}H NMR (400 MHz, $CDCl_3$): δ = 6.33 (d, *J* = 1.6 Hz, 1H, CH), 6.55 (d, *J* = 1.6 Hz, 1H, CH), 7.30–7.49 (m, 10H, ArH) ppm; ^{13}C NMR (100 MHz, $CDCl_3$): δ = 84.8, 115.1, 128.0 (2C), 128.4 (2C), 129.5 (2C), 129.6 (2C), 130.1, 131.7, 135.3, 166.2, 173.1 ppm.

4,5-Bis(4-chlorophenyl)furan-2(5*H*)-one (2b):[18]

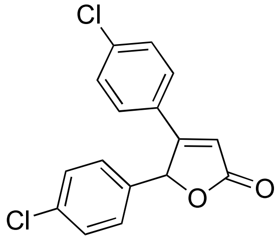

2b

Yellow solid; mp 124-126 °C; yield: 95% (58 mg, 0.19 mmol); ^{1}H NMR (400 MHz, $CDCl_3$): δ = 6.27 (d, *J* = 1.6 Hz, 1H, CH), 6.54 (d, *J* = 1.6 Hz, 1H, CH), 7.23 (d, J_1 = 2.0 & J_2 = 8.8 Hz, 2H, ArH), 7.33–7.35 (m, 6H, ArH) ppm; ^{13}C NMR (100 MHz, $CDCl_3$): δ = 83.3, 115.2, 127.9, 128.8 (2C), 129.2 (2C), 129.5 (2C), 129.6 (2C), 133.1, 135.9, 137.7, 164.1, 172.0 ppm.

5-(4-Bromophenyl)-4-phenylfuran-2(5*H*)-one and 4-(4-Bromophenyl)-5-phenylfuran-2(5*H*)-one (2c):

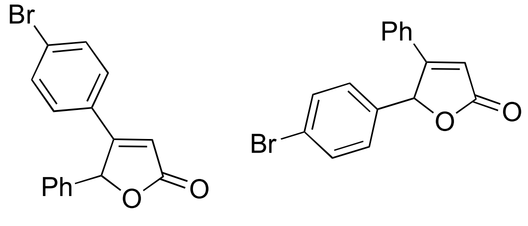

2c

Yellow oil; yield: 88% (55 mg, 0.176 mmol); mixture of two isomers was observed in a 1:1 ratio ^{1}H NMR (400 MHz, $CDCl_3$): δ = 6.07 (d, *J* = 1.6 Hz, 1H, CH), 6.08 (d, *J* = 1.2 Hz, 1H, CH), 6.32 (d, *J* = 1.2 Hz, 1H, CH), 6.33 (d, *J* = 1.6 Hz, 1H, CH), 6.97 (d, *J* = 8.4 Hz, 2H, 2ArH), 7.03–7.08 (m, 4H, ArH), 7.12–7.18 (m, 8H, ArH), 7.23–7.26 (m, 4H, ArH) ppm; ^{13}C NMR (100 MHz, $CDCl_3$): δ = 84.4, 84.2, 114.8, 115.2, 123.9, 125.9, 127.6 (2C), 127.9 (2C), 128.6, 129.0 (2C), 129.2 (2C), 129.3 (2C), 129.4, 129.6 (2C), 129.9, 131.5, 132.37 (2C), 132.42 (2C), 134.0, 134.6, 164.6, 165.6, 172.3, 172.4 ppm; IR (film): ν = 3480, 1747, 1617, 1496, 1449, 1307, 1167, 1036, 984 cm^{-1}; HRMS (API): m/z [M]$^+$ calcd for $C_{15}H_{11}BrO_2$: 313.9942; found 313.9947.

4,5-Diphenylfuran-2(5*H*)-one (2d):[19]

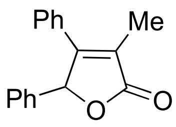

2d

Colorless oil; yield: 81% (40 mg, 0.162 mmol); ^{1}H NMR (400 MHz, $CDCl_3$): δ = 2.16 (d, *J* = 1.6 Hz, 3H, Me), 6.19 (q, *J* = 1.6 Hz, 1H, CH), 7.20–7.23 (m, 2H, ArH), 7.25–7.30 (m, 5H, ArH), 7.33–7.36 (m, 3H, ArH) ppm; ^{13}C NMR (100 MHz, $CDCl_3$): δ = 10.8, 84.2, 124.6, 128.0 (2C), 128.5 (2C), 129.2 (2C), 129.3 (2C), 129.7, 130.1, 131.8, 135.5, 158.8, 175.0 ppm.

4-Phenylfuran-2(5*H*)-one (2e):[20]

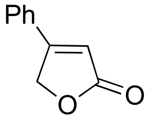

2e

Colorless solid; mp 92-94 °C; yield: 78% (25 mg, 0.156 mmol); ^{1}H NMR (400 MHz, $CDCl_3$): δ = 5.23 (d, *J* = 1.8 Hz, 2H, CH_2), 6.38 (t, *J* = 1.8 Hz, 1H, CH), 7.46–7.52 (m, 5H, ArH) ppm; ^{13}C NMR (125 MHz, $CDCl_3$): δ = 71.0, 113.2, 126.4 (2C), 129.3 (2C), 129.8, 131.8, 163.8, 173.7 ppm.

5-Methyl-4-phenylfuran-2(5*H*)-one (2f):[21]

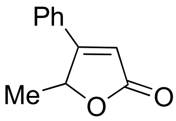

2f

Colorless solid; mp 50-52 °C; yield: 89% (31 mg, 0.178 mmol); ^{1}H NMR (400 MHz, $CDCl_3$): δ = 1.54 (d, *J* = 5.2 Hz, 3H, Me), 5.57 (dq, J_1 = 1.2 Hz, J_2 = 5.6 Hz, 1H, CH), 6.28 (d, *J* = 1.2 Hz, 1H, CH), 7.45–7.50 (m, 5H, ArH) ppm; ^{13}C NMR (125 MHz, $CDCl_3$): δ = 19.8, 78.6, 113.8, 127.2 (2C), 129.2 (2C), 130.0, 131.3, 168.9, 172.5 ppm.

3,5-Dimethyl-4-phenylfuran-2(5*H*)-one (2g):[22]

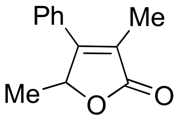

2g

Yellow solid; mp 72–74 °C; yield: 87% (33 mg, 0.174 mmol); ^{1}H NMR (400 MHz, $CDCl_3$): δ = 1.37 (d, *J* = 6.8, 3H, Me), 2.05 (d, *J* = 1.6, 3H, Me), 5.41 (qq, J_1 = 1.6 & J_2 = 6.8 Hz, 1H, CH), 7.33–7.36 (m, 2H, ArH), 7.44–7.51 (m, 3H, ArH) ppm; ^{13}C NMR (100 MHz, $CDCl_3$): δ = 10.5, 19.7, 78.6, 123.6, 128.2 (2C), 129.5 (2C), 130.2, 131.9, 161.0, 174.9 ppm.

5-Methylfuran-2(5*H*)-one (2h):[23]

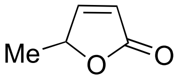

2h

Colorless oil; yield: 81% (28 mg, 0.162 mmol); ^{1}H NMR (500 MHz, $CDCl_3$): δ = 1.37 (d, *J* = 6.8 Hz, 3H, Me), 5.05 (tq, J_1 = 1.6 Hz, J_2 = 6.8 Hz, 1H, CH), 6.01 (dd, J_1 = 2.0 Hz, J_2 = 5.6 Hz, 1H, CH), 7.37 (dd, J_1 = 1.2 Hz, J_2 = 5.6 Hz, 1H, CH) ppm.

5-[^{2}H]-4-Phenylfuran-2(5*H*)-one (2i):

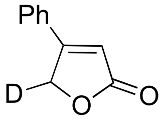

2i

Colorless oil; yield: 58% (9 mg, 0.058 mmol); ^{1}H NMR (500 MHz, $CDCl_3$): δ = 6.02 (d, *J* = 2.0 Hz, 1H, CH), 6.23 (d, *J* = 2.0 Hz, 1H, CH), 7.26–7.28 (m, 2H, ArH), 7.37–7.41 (m, 3H, ArH) ppm; ^{13}C NMR (125 MHz, $CDCl_3$): δ = 29.7, 120.9, 126.5 (2C), 129.1 (2C), 129.3, 144.0, 153.2, 172.4 ppm; HRMS (NSI) m/z $[M]^+$ calcd for $C_{10}H_7DO_2$: 161.0587; found 161.0587.

4-Oxo-3,4-diphenylbutanoic acid (3a):[24]

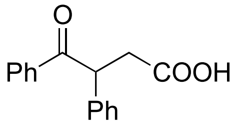

3a

Colorless solid; mp 152–154 °C; yield: 92% (47 mg, 0.184 mmol); ^{1}H NMR (500 MHz, $CDCl_3$): δ = 2.72 (dd, J_1 = 9.5 Hz, J_2 = 34.5 Hz 1H, CH), 3.39 (dd, J_1 = 19.5 Hz, J_2 = 34.5 Hz 1H, CH), 5.03 (dd, J_1 = 9.5 Hz, J_2 = 19.5 Hz 1H, CH), 7.19-7.28 (m, 4H, ArH), 7.32-7.46 (m, 4H, ArH), 7.91-7.96 (m, 2H, ArH) ppm; ^{13}C NMR (125 MHz, $CDCl_3$): δ = 38.3, 49.5, 127.6, 128.1 (2C), 128.5 (2C), 128.9 (2C), 129.3 (2C), 133.0, 136.1, 137.9, 177.3, 198.3 ppm.

4-Oxo-3-phenylpentanoic acid (3f):[13]

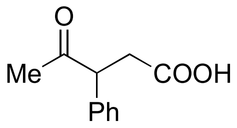

3f

Colorless solid; m.p.: 98–100 ºC; yield: 89% (34 mg, 0.178 mmol); ^{1}H NMR (400 MHz, $CDCl_3$): δ = 2.02 (s, 3H, Me), 2.47 (dd, J_1 = 4.8 Hz, J_2 = 17.2 Hz 1H, CH), 3.18 (dd, J_1 = 10.0 Hz, J_2 = 17.2 Hz 1H, CH), 4.07 (dd, J_1 = 4.8 Hz, J_2 = 9.6 Hz 1H, CH), 7.12 (d, J = 7.2 Hz, 2H, ArH), 7.20-7.29 (m, 3H, ArH), 9.97 (brs, 1H, COOH) ppm; ^{13}C NMR (125 MHz, $CDCl_3$): δ = 28.8, 36.7, 54.7, 127.9, 128.2 (2C), 129.3 (2C), 137.2, 177.6, 206.7 ppm.

3-Methyl-5,5-diphenylfuran-2(5*H*)-one (4d):

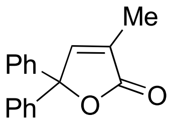

4d

Colorless oil; yield: 16% (8 mg, 0.32 mmol); ^{1}H NMR (400 MHz, $CDCl_3$): δ = 1.90 (s, 3H, Me), 6.64-6.67 (m, 2H, ArH), 7.28–7.38 (m, 6H, 1CH & 5ArH), 7.49 (d, J = 8.0 Hz, 4H, ArH) ppm; ^{13}C NMR (100 MHz, $CDCl_3$): δ = 8.9, 89.7, 125.6, 126.7, 127.2, 127.3, 127.4, 127.6, 128.6, 130.6, 134.2, 160.6, 171.0 ppm; IR (film): ν = 3343, 2966,

2933, 2874, 1736, 1612, 1551, 1488, 1445, 1371, 1258, 1188, 1125, 1083, 1057, 913 cm^{-1}; HRMS (API): m/z $[M]^+$ calcd for $C_{17}H_{14}O_2$: 250.0994; found 250.0994.

5-Methyl-5-phenylfuran-2(5*H*)-one (4f):[13,25]

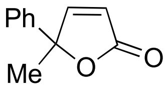

4f

Colorless oil; yield: 81% (28 mg, 0.162 mmol); ^{1}H NMR (500 MHz, $CDCl_3$): δ = 1.83 (s, 3H, Me), 6.05 (d, J = 5.5 Hz, 1H, CH), 7.31–7.38 (m, 5H, ArH), 7.63 (d, J = 5.5 Hz, 1H, CH) ppm; ^{13}C NMR (125 MHz, $CDCl_3$): δ = 26.4, 88.9, 119.4, 124.8 (2C), 128.4, 128.9 (2C), 139.3, 160.3, 172.3 ppm.

5,5-Diphenylpent-4-enoic acid (5a):[26]

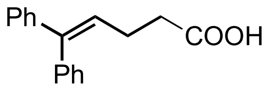

5a

(3-Carboxypropyl)triphenylphosphonium bromide (429 mg, 1.0 mmol) was suspended in THF (15 mL). The colorless suspension was cooled to 0 °C and *n*-BuLi (2.5 M in hexane) (0.8 mL, 2.0 mmol) was added dropwise and the resulting bright orange solution was stirred at 0 °C for 1 h. A solution of benzophenone (218 mg, 1.2 mmol) in THF (5 mL) was added dropwise and the reaction mixture was stirred overnight at room temperature. The reaction was monitored by thin layer chromatography. After completion of the reaction, the solvent was removed under vacuum and reaction mixture was poured into ice-cold water (5 mL). The reaction mixture was acidified by dropwise addition of 1 M HCl up to pH 2-3, after that the reaction mixture was extracted with ethyl acetate (3 x 5 mL). The combined organic layers were dried over $MgSO_4$, filtered and concentrated under reduced pressure. The crude product was purified by column chromatography by using methanol / chloroform (1:99) as eluent.

Colorless solid; m.p.: 74–76 °C; yield: 82% (207 mg, 0.82 mmol); ^{1}H NMR (400 MHz, $CDCl_3$): δ = 2.42-2.49 (m, 4H, 2 x CH_2), 6.06 (t, J = 7.2 Hz, 1H, CH), 7.16-7.37 (m, 10H, ArH) ppm; ^{13}C NMR (125 MHz, $CDCl_3$): δ = 24.9, 34.2, 126.8, 127.26, 127.19, 127.3 (2C), 128.1 (2C), 128.3 (2C), 129.7 (2C), 139.7, 142.3, 143.4, 179.2 ppm; IR (film): ν = 3445, 2918, 1635, 913, 743 cm^{-1}.

Synthesis of 5b:

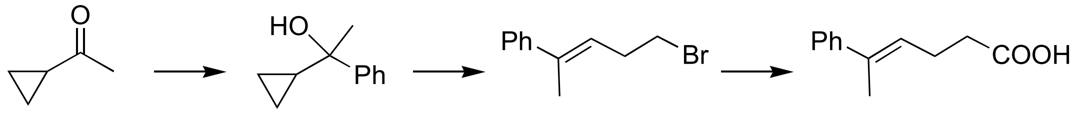

S7 **S8** **5b**

1-Cyclopropyl-1-phenylethanol (S7):[27]

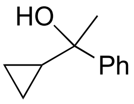

S7

To a magnetically stirred mixture of Mg turnings (6 mmol, 144 mg) and iodine (catalytic amount), a solution of bromobenzene (785 mg, 5 mmol) in dry THF (10 mL) was added dropwise during 10 min and allowed to stir at room temperature for 2 h. Then temperature was decreased to 0 ^{o}C and cyclopropyl methyl ketone (5 mmol, 0.465 mL) was added and allowed to stir at room temperature for 1 h. After completion of the reaction, water (5 mL) was added and the reaction mixture was extracted with ethyl acetate (3 x 5 mL). The combined organic layers were dried over $MgSO_4$, filtered and concentrated under reduced pressure. The crude product was purified by column chromatography using hexane and chloroform (1:1) as eluent.

Colorless oil; yield: 83% (0.67 g, 4.14 mmol); ^{1}H NMR (400 MHz, $CDCl_3$): δ = 0.31-0.47 (m, 4H, 2 x CH_2), 1.15-1.22 (m, 1H, CH), 1.42 (s, 3H, Me), 1.54 (brs, 1H, OH), 7.15-7.19 (m, 1H, ArH), 7.24-7.29 (m, 2H, ArH), 7.45-7.47 (m, 2H, ArH) ppm.

(*E*)-(5-Bromopent-2-en-2-yl)benzene (S8):[28]

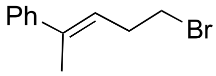

S8

The mixture of 1-cyclopropyl-1-phenylethanol **S7** (324 mg, 2 mmol), aq. HBr solution (48%) (0.34 mL, 2 mmol) and lithium bromide (174 mg, 2 mmol) was stirred at 0 ºC for 2 h. The reaction was monitored by TLC. After completion of the reaction, the reaction mixture was diluted with water (2 mL) and extracted with ethyl acetate (3 x 5 mL). The combined organic layers were dried over $MgSO_4$, filtered and concentrated under

reduced pressure. The crude product was purified by column chromatography by using hexane as eluent.

Colorless oil; yield: 96% (430 mg, 1.92 mmol); ^{1}H NMR (400 MHz, $CDCl_3$): δ = 2.09 (s, 3H, Me), 2.82 (q, *J* = 7.2 Hz, 2H, CH_2), 3.49 (t, *J* = 7.2 Hz, 2H, CH_2), 5.78 (t, *J* = 7.2 Hz, 1H, CH), 7.27-7.29 (m, 1H, ArH), 7.35 (t, *J* = 7.6 Hz, 2H, ArH), 7.42 (d, *J* = 7.6 Hz, 2H, ArH) ppm; ^{13}C NMR (100 MHz, $CDCl_3$): δ = 16.6, 32.7, 32.8, 124.8, 126.2 (2C), 127.5, 128.7 (2C), 138.2, 143.7 ppm; IR (film): ν = 3080, 3056, 3030, 2961, 2921, 2860, 1597, 1494, 1444, 1380, 1304, 1261, 1207, 1061, 1027, 856, 757, 696, 646, 560, 548 cm^{-1}.

(*E*)-5-Phenylhex-4-enoic acid (5b):[26]

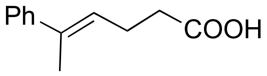

5b

To a magnetically stirred mixture of Mg turnings (36 mg, 1.2 mmol) and iodine (catalytic amount) a solution of (*E*)-(5-bromopent-2-en-2-yl)benzene **S6** (224 mg, 1.0 mmol) in dry THF (5 mL) was added dropwise and allowed to heat at reflux temperature for 2 h under nitrogen atmosphere. After 2 h, the reaction was cooled to room temperature and one pellet of dry ice was added. After 30 min stirring at room temperature, water (2 mL) was added and reaction mixture was acidified by 1 M HCl solution. The reaction mixture was extracted with ethyl acetate (3 x 5 mL). The combined organic layers were dried over $MgSO_4$, filtered and concentrated under reduced pressure. The crude product was purified by column chromatography using chloroform as eluent.

Colorless solid; m.p.: 74 ºC; yield: 74% (128 mg, 0.74 mmol); ^{1}H NMR (400 MHz, $CDCl_3$): δ = 1.98 (s, 3H, Me), 2.41-2.49 (m, 4H, 2 x CH_3), 5.66 (t, *J* = 6.4 Hz, 1H, CH), 7.15 (t, *J* = 7.4 Hz, 1H, ArH), 7.23 (t, *J* = 7.4 Hz, 2H, ArH), 7.29 (d, *J* = 7.2 Hz, 2H, ArH), 11.19 (brs, 1H, COOH) ppm; ^{13}C NMR (100 MHz, $CDCl_3$): δ = 15.9, 24.0, 34.0, 125.6, 125.7 (2C), 126.9, 128.2 (2C), 136.6, 143.5, 179.6 ppm; IR (film): ν = 3465, 3031, 1708, 1449, 1444, 1410, 1381, 1027, 913, 758, 696 cm–1; HRMS calcd for $C_{12}H_{13}O_2$: 189.0921; found 189.0919.

5,6-Diphenyl-3,4-dihydro-2*H*-pyran-2-one (6a):[29]

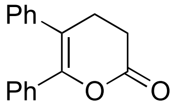

6a

Colorless oil; yield: 76% (38 mg, 0.152 mmol); ^{1}H NMR (500 MHz, $CDCl_3$): δ = 2.83-2.85 (m, 4H, 2 x CH_2), 7.10-7.13 (m, 2H, ArH), 7.16-7.25 (m, 8H, ArH) ppm; ^{13}C NMR (125 MHz, $CDCl_3$): δ = 27.0, 28.9, 116.5, 127.4, 127.9 (2C), 128.57 (2C), 128.59 (4C), 129.0, 130.0, 133.1, 138.5, 147.3, 168.7 ppm; HRMS (API) m/z $[M]^+$ calcd for $C_{17}H_{15}O_2$: 251.1070; found 251.1067.

5-Oxo-4-phenylhexanoic acid (6b):[29]

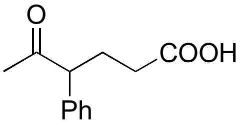

6b

Colorless solid; m.p.: 44–46 ºC; yield: 79% (33 mg, 0.158 mmol); ^{1}H NMR (400 MHz, $CDCl_3$): δ = 1.87-1.93 (m, 1H, CH_2), 1.96 (s, 3H, Me), 2.13-2.28 (m, 3H, CH_2), 3.62 (dd, J_1 = 6.8 Hz & J_2 = 8.0 Hz 1H, CH), 7.08-7.12 (m, 2H, ArH), 7.16-7.20 (m, 1H, ArH), 7.22-7.26 (m, 2H, ArH), 8.89 (brs, 1H, COOH) ppm; ^{13}C NMR (100 MHz, CDC_3): δ = 26.6, 29.0, 31.4, 58.3, 127.6, 128.3 (2C), 129.2 (2C), 138.0, 178.9, 207.6 ppm; MS (EI): 205.0869.

General procedure for the synthesis of 6,6-diarylhex-5-enoic acids 8:

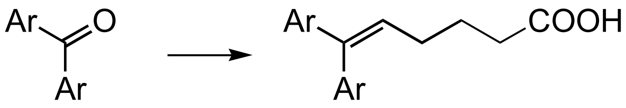

8a (Ar=Ph)
8b (Ar=4-Cl-C_6H_4)

(3-Carboxybutyl)triphenylphosphonium bromide (443 mg, 1.0 mmol) was suspended in THF (15 mL). The colorless suspension was cooled to 0 °C and *n*-BuLi (2.5 M in hexane, 0.8 mL, 2.0 mmol) was added dropwise and the resulting bright orange solution was stirred at 0 °C for 1 h. A solution of the benzophenone (1.2 mmol) in THF (5 mL) was added dropwise and the reaction mixture was stirred overnight at room temperature. The reaction was monitored by thin layer chromatography. After

completion of the reaction, the solvent was removed under vacuum and reaction mixture was poured into ice-cold water. After the acidification with 1 M HCl, the reaction mixture was extracted with ethyl acetate (3 x 5 mL). The combined organic layers were dried over $MgSO_4$, filtered and concentrated under reduced pressure. The crude product was purified by column chromatography by using chloroform as eluent.

6,6-Diphenylhex-5-enoic acid (8a):[26a]

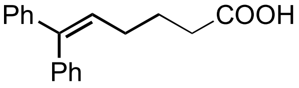

8a

White solid; mp 94 °C; yield: 69% (183 mg, 0.69 mmol); ^{1}H NMR (400 MHz, $CDCl_3$): δ = 1.71 (q, J = 7.4 Hz, 2H, CH_2), 2.10 (q, J = 7.4 Hz, 2H, CH_2), 2.26 (t, J = 7.4 Hz, 2H, CH_2), 5.98 (t, J = 7.4 Hz, 1H, CH), 7.09 (d, J = 7.2 Hz, 2H, ArH), 7.12-7.25 (m, 6H, ArH), 7.29 (t, J = 7.4 Hz, 2H, ArH) ppm; ^{13}C NMR (100 MHz, $CDCl_3$): δ = 24.8, 29.1, 33.5, 127.0, 127.2 (2C), 128.1 (2C), 128.2 (2C), 128.4, 129.9 (2C), 140.0, 142.5, 142.7, 179.4 ppm; IR (film): ν = 3497, 1707, 1494, 1443, 759, 700 cm^{-1}; HRMS calcd for $C_{18}H_{17}O_2$: 265.1234; found 265.1232.

6,6-Bis(4-chlorophenyl)hex-5-enoic acid (8b):

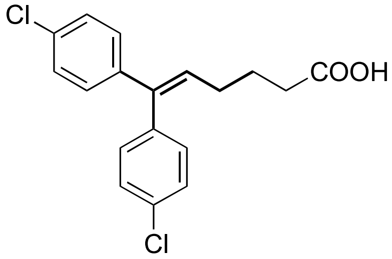

8b

Yellow solid; mp 78 °C; yield: (261 mg, 0.78 mmol) 78%; ^{1}H NMR (400 MHz, $CDCl_3$): δ = 1.65 (q, J = 7.4 Hz, 2H, CH_2), 2.03 (q, J = 7.4 Hz, 2H, CH_2), 2.21 (t, J = 7.4 Hz, 2H, CH_2), 5.91 (t, J = 7.4 Hz, 1H, CH), 6.96 (dd, J_1 = 3.2 Hz & J_2 = 8.4 Hz, 4H, ArH), 7.09 (d, J = 8.4 Hz, 2H, ArH), 7.21 (d, J = 7.6 Hz, 2H, ArH) ppm; ^{13}C NMR (100 MHz, $CDCl_3$): δ = 24.6, 29.1, 33.5, 128.4 (2C), 128.5 (2C), 128.6 (2C), 129.5, 131.2 (2C), 133.1, 133.2, 137.9, 140.5, 140.6, 179.9 ppm; IR (film): ν = 3478, 2952, 1707, 1492,

1403, 1091, 1014, 913, 830, 743 cm^{-1}; HRMS calcd for $C_{18}H_{15}O_2Cl_2$: 3330455.1234; found 333.0454.

2-(Diphenylmethylene)cyclopentanone (9a):[30]

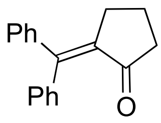

9a

Yellow solid; mp 110–112 °C; yield: 90% (45 mg, 0.180 mmol); ^{1}H NMR (400 MHz, $CDCl_3$): δ = 1.93 (p, *J* = 7.4 Hz, 2H, CH_2), 2.38 (t, *J* = 7.8 Hz, 2H, CH_2), 2.82 (t, *J* = 7.0 Hz, 2H, CH_2), 7.10-7.13 (m, 2H, ArH), 7.17-7.20 (m, 2H, ArH), 7.28-7.36 (m, 6H, ArH) ppm; ^{13}C NMR (100 MHz, $CDCl_3$): δ = 20.6, 33.0, 39.8, 127.9 (3C), 128.0 (2C), 128.4, 129.5 (2C), 129.7 (2C), 134.4, 140.2, 141.8, 148.3, 206.6 ppm.

X-Ray structure of **9a**:

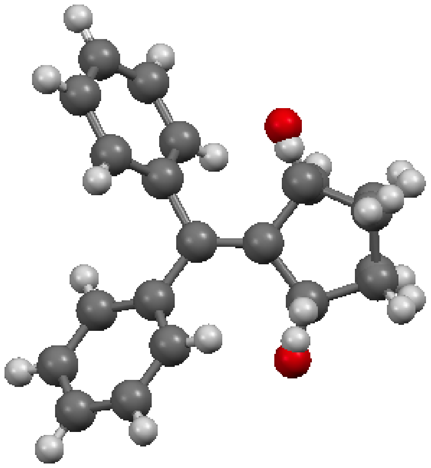

CCDC-883284 [**9a**] contains the supplementary crystallographic data for this paper. These data can be obtained free of charge from The Cambridge Crystallographic Data Centre via www.ccdc.cam.ac.uk/data_request/cif.

2-(Bis(4-chlorophenyl)methylene)cyclopentanone (9b):

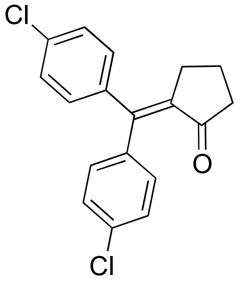

9b

Yellow solid; mp 132–134 ºC; yield: 80% (51 mg, 0.160 mmol); ^{1}H NMR (400 MHz, $CDCl_3$): δ = 1.94 (p, J = 7.4 Hz, 2H, CH_2), 2.38 (t, J = 7.6 Hz, 2H, CH_2), 2.79 (t, J = 7.0 Hz, 2H, CH_2), 7.03 (d, J = 8.4 Hz, 2H, ArH), 7.10 (d, J = 8.4 Hz, 2H, ArH), 7.29 (d, J = 8.4 Hz, 2H, ArH), 7.31 (d, J = 8.4 Hz, 2H, ArH) ppm; ^{13}C NMR (100 MHz, $CDCl_3$): δ = 20.5, 33.0, 39.8, 128.3 (2C), 128.4 (2C), 130.9 (2C), 131.0 (2C), 134.1, 134.7, 135.1, 138.0, 139.8, 145.6, 206.5 ppm; IR (KBr): 3434, 2953, 2928, 1712, 1582, 1495, 1210, 1091, 804 cm^{-1}.

4-Iodo-5-methyl-5-phenyldihydrofuran-2(3*H*)-one (22):[14]

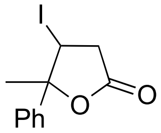

22

A solution of iodine monochloride (324 mg, 2.0 mmol) in THF (5 mL) was added to a solution of (*E*)-4-phenylpent-3-enoic acid **1f** (176 mg, 1.0 mmol) in THF (5 mL) and stirred at room temperature for 1 h. The reaction was monitored by thin layer chromatography. After completion of the reaction, the solvent was removed under vacuum and reaction mixture was poured into ice-cold water (5 mL). After that the saturated solution of $Na_2S_2O_3$ was added dropwise until the violet color was completely disappeared and the reaction mixture was extracted with dichloromethane (3 x 5 mL). The combined organic layers were dried over $MgSO_4$, filtered and concentrated under reduced pressure. The crude product was purified by column chromatography using hexane as eluent.

Light yellow solid; mp 88–90 ºC; yield: 76% (229 mg, 0.76 mmol); ^{1}H NMR (400 MHz, $CDCl_3$): δ = 1.94 (s, 3H, Me), 3.02 (dd, J_1 = 6.2 Hz, J_2 = 18.2 Hz 1H, CH), 3.22

(dd, J_1 = 7.4 Hz, J_2 = 18.2 Hz 1H, CH), 4.65 (dd, J_1 = 6.4 Hz, J_2 = 7.2 Hz 1H, CH), 7.34-7.43 (m, 3H, ArH), 7.43-7.46 (m, 2H, ArH) ppm; ^{13}C NMR (100 MHz, $CDCl_3$): δ = 18.2, 31.0, 42.6, 88.5, 124.7 (2C), 128.9, 129.4 (2C), 141.2, 174.0 ppm;

4-Oxo-3-phenylpentanoic acid (3f):[13]

Oxone (potassium peroxomonosulfate, 184 mg, 0.3 mmol) was added to solution of 4-iodo-5-methyl-5-phenyldihydrofuran-2(3*H*)-one **22** (60 mg, 0.2 mmol) in chloroform (0.2 mL) and trifluoroacetic acid (0.6 mL) and the reaction mixture was stirred at room temperature for 2 h. The reaction was monitored by thin layer chromatography. After the completion of the reaction, the inorganic material was removed by filtration and the filtrate was concentrated under vacuum. After that the saturated solution of $Na_2S_2O_3$ was added dropwise until the violet color was completely disappeared and the reaction mixture was extracted with dichloromethane (3 x 5 mL). The combined organic layers were dried over $MgSO_4$, filtered and concentrated under reduced pressure. The crude product was purified by column chromatography using dichloromethane / hexane (4:1) as eluent. The isolated product was characterized as 4-oxo-3-phenylpentanoic acid **3f** which was obtained in 86% yield.

(*Z*)-5-(Iodomethylene)-4-phenylfuran-2(5*H*)-one (23):[31]

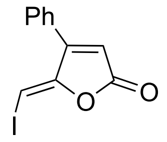

23

A solution of 4-iodo-5-methyl-5-phenyldihydrofuran-2(3*H*)-one **22** (60 mg, 0.2 mmol) in acetonitrile (2 mL) was added to a solution of [bis(trifluoroacetoxy)iodo]benzene (86 mg, 0.2 mmol) and TMSOTf (0.072 mL, 0.4 mmol) in acetonitrile (2 mL) at room temperature and stirred overnight. The reaction was monitored by TLC. Acetonitrile was removed in vacuuo, water (2 mL) was added and the mixture extracted with CH_2Cl_2 (3 x 5 mL). The combined organic layers were dried over $MgSO_4$, filtered and concentrated in vacuo. The product mixture was purified by column chromatography using chloroform as eluent.

Colorless solid; mp 78–80 °C; yield: 83% (49 mg, 0.166 mmol); ^{1}H NMR (400 MHz, $CDCl_3$): δ = 6.35 (s, 1H, CH), 6.44 (s, 1H, CH), 7.43–7.46 (m, 2H, ArH), 7.47-7.53 (m,

3H, ArH) ppm; ^{13}C NMR (125 MHz, $CDCl_3$): δ = 65.0, 117.1, 128.8 (2C), 129.7 (2C), 130.0, 131.4, 155.4, 156.5, 167.6 ppm.

1-(Methylsulfonyl)-4-vinylbenzene:[32]

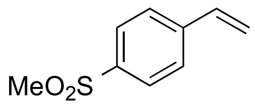

A solution of (methyl)triphenylphosphonium bromide (357 mg, 1.0 mmol) in dimethyl sulfoxide (10 mL) was added dropwise to a stirred suspension of potassium *tert*-butoxide (134 mg, 1.2 mmol) in dimethyl sulfoxide (5 mL) and the resulting bright orange solution was stirred at room temperature for 30 min. A solution of 4-(methylsulfonyl)benzaldehyde (184 mg, 1.0 mmol) in dimethyl sulfoxide (5 mL) was added dropwise and the reaction mixture was stirred overnight at room temperature. The reaction was monitored by thin layer chromatography. After completion of the reaction, the reaction mixture was poured into ice-cold water (10 mL). After the acidification with 1 M HCl, the reaction mixture was extracted with dichloromethane (3 x 10 mL). The organic layer was dried over $MgSO_4$, filtered and concentrated under reduced pressure. The crude product was purified by column chromatography on silica gel by using ethyl acetate / hexane (3:7) as eluent.

Colorless solid; m.p.: 40-42 °C; yield: 67% (122 mg, 0.67 mmol); ^{1}H NMR (500 MHz, $CDCl_3$): δ = 3.05 (s, 3H, SO_2Me), 5.47 (d, J = 11.0 Hz, 1H, CH), 5.91 (d, J = 17.5 Hz, 1H, CH), 6.77 (dd, J_1 = 11.0, J_2 = 17.5 Hz, 1H, CH), 7.57 (d, J = 8.0 Hz, 2H, ArH), 7.90 (d, J = 8.5 Hz, 2H, ArH) ppm; ^{13}C NMR (125 MHz, $CDCl_3$): δ = 44.6, 118.0, 127.0 (2C), 127.8 (2C), 135.2, 139.5, 143.0 ppm.

General procedure for the synthesis of (*E*)-2,4-diarylbut-3-enoic acids 24a and 24b:

A mixture of 2-bromo-2-phenylacetic acid (107 mg, 0.5 mmol), styrene (0.07 mL, 0.6 mmol) [or 1-(methylsulfonyl)-4-vinylbenzene (109 mg, 0.6 mmol)], tributylamine (0.237 mL, 1.0 mmol), palladium acetate (5.6 mg, 0.025 mmol) and triphenylphosphine (13.1 mg, 0.05 mmol) was heated at 100 °C for 24 h. Full conversion was not observed. The reaction mixture was poured into ice-cold water (5 mL), acidified with 1 M HCl and extracted with dichloromethane (3 x 5 mL). The organic layer was dried over $MgSO_4$,

filtered and concentrated under reduced pressure. The crude product (**24a**) was purified by column chromatography on silica gel by using ethyl acetate / hexane (3:7) while the crude product **24b** was purified by same method using ethyl acetate / hexane (2:3) as eluent.

(*E*)-2,4-Diphenylbut-3-enoic acid (24a):

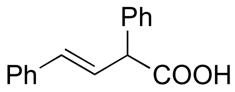

24a

Colorless solid; m.p.: 138-140 °C; yield: 49% (58 mg, 0.245 mmol); ^{1}H NMR (400 MHz, DMSO-d$_6$): δ = 5.26 (d, J = 6.0 Hz, 1H, CH), 6.40 (dd, J_1 = 6.4, J_2 = 16.0 Hz, 1H, CH), 6.65 (d, J = 16.0 Hz, 1H, CH), 7.19-7.26 (m, 2H, ArH), 7.28-7.36 (m, 4H, ArH), 7.40-7.42 (m, 4H, ArH) ppm; ^{13}C NMR (100 MHz, CDCl$_3$): δ = 76.7, 129.7 (2C), 129.8 (2C), 130.4, 130.9, 131.6, 131.7 (2C), 132.1 (2C), 137.2, 140.1, 147.9, 164.5 ppm; IR (KBr): ν = 2966, 2933, 2875, 1735, 1258, 1083, 1057, 1000, 913, 748 cm^{-1}; HRMS (APCI): m/z [M+1]$^+$ calcd for $C_{16}H_{15}O_2$: 239.1072; found 239.1079.

(*E*)-4-(4-(Methylsulfonyl)phenyl)-2-phenylbut-3-enoic acid (24b):

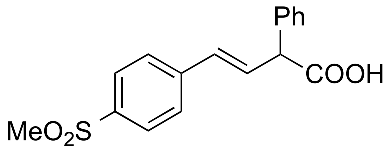

24b

Colorless solid; m.p.: 160-162 °C; yield: 38% (59 mg, 0.190 mmol); ^{1}H NMR (400 MHz, DMSO-d$_6$): δ = 3.18 (s, 3H, SO$_2$Me), 5.31 (d, J = 5.6 Hz, 1H, CH), 6.64 (dd, J_1 = 5.6, J_2 = 16.0 Hz, 1H, CH), 6.78 (d, J = 16.0 Hz, 1H, CH), 7.25 (t, J = 8.0 Hz, 1H, ArH), 7.32-7.37 (m, 2H, ArH), 7.42 (d, J = 7.6 Hz, 2H, ArH), 7.69 (d, J = 7.6 Hz, 2H, ArH), 7.85 (d, J = 8.4 Hz, 2H, ArH) ppm; ^{13}C NMR (100 MHz, CDCl$_3$): δ = 47.9, 77.3, 130.7 (2C), 130.8, 131.4 (2C), 131.5, 131.8 (2C), 132.6 (2C), 142.0, 143.5, 146.2, 148.2, 165.3 ppm; IR (KBr): ν = 2961, 2923, 2870, 1739, 1639, 1443, 1133, 968, 770, 701 cm^{-1}; HRMS (APCI): m/z [M+1]$^+$ calcd for $C_{17}H_{17}O_4S$: 317.0847; found 317.0844.

3,4-Diphenylfuran-2(5*H*)-one (25a):[33]

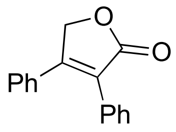

25a

Colorless solid; m.p.: 96-98 °C; yield: 87% (41 mg, 0.174 mmol); ^{1}H NMR (400 MHz, $CDCl_3$): δ = 5.18 (s, 2H, CH_2), 7.31-7.44 (m, 10H, ArH) ppm; ^{13}C NMR (100 MHz, $CDCl_3$): δ = 70.6, 126.2, 127.5 (2C), 128.7 (2C), 128.8, 129.0 (2C), 129.3 (2C), 130.2, 130.6, 130.9, 156.1, 173.5 ppm; HRMS (APCI): m/z $[M+1]^+$ calcd for $C_{16}H_{13}O_2$: 237.0916; found 237.0921.

4-(4-(Methylsulfonyl)phenyl)-3-phenylfuran-2(5*H*)-one (25b):[34]

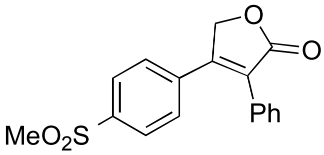

25b

Colorless solid; m.p.: 202-204 °C; yield: 83% (26 mg, 0.083 mmol); ^{1}H NMR (400 MHz, $CDCl_3$): δ = 3.18 (s, 3H, SO_2Me), 5.19 (s, 2H, CH_2), 7.37-7.41 (m, 5H, ArH), 7.51 (d, J = 8.4 Hz, 2H, ArH), 7.91 (d, J = 8.4 Hz, 2H, ArH) ppm; ^{13}C NMR (100 MHz, $CDCl_3$): δ = 44.7, 70.8, 128.5 (2C), 128.9 (2C), 129.4 (3C), 129.5, 129.6 (2C), 129.9, 136.7, 142.4, 153.9, 173.0 ppm; HRMS (APCI): m/z $[M+1]^+$ calcd for $C_{17}H_{15}O_4S$: 315.0682; found 315.0691.

Calculations:

DFT calculations were performed using the GAUSSIAN03 suit of programs.[35] Reactants, transition states (TSs), intermediates, and products were fully optimized with the hybrid density functional B3LYP[36] using the Pople-type 6-31+G(d,p) basis set[37] for all compounds without iodine and a composite basis set consisting of 6-31+G(d,p) for C, H, Se, O, F and LANL2DZ for I incorporating a relativistic pseudopotential (effective core potential, ECP) that largely accounts for scalar relativistic effects in iodine.[38] Solvent effects of acetonitrile were approximated using the SCRF approach employing PCM as implemented in Gaussian03.[39] Energies in solution were either obtained as single point calculations on gas phase geometries (SCRF=PCM//B3LYP/6-31+G**). All transition states have been characterized by one imaginary frequency (first-order saddle points) on the Potential Energy Surface (PES).[40] To determine Minimum Energy Pathways (MEPs) Intrinsic Reaction Coordinate analyses (IRC) were performed, in order to confirm that a specific TS connects the different local minima.[41] In addition, the imaginary frequencies were visually analysed and proven to be the correct eigen vibration by animating it in Gabedit.[42] Vibrational frequencies and Zero-Point vibrational Energies (ZPE) were determined within the harmonic oscillator approximation, at the same level of theory as that for geometries. All energies reported in this paper are free energies or enthalpies in kcal mol^{-1} at 298 K and 1 atm if not stated otherwise. Frequencies remained unscaled. Gabedit was used to generate XYZ-matrices of Gaussian log-files and to obtain geometric data. Rendered graphics were generated with CYLview v1.0.301 BETA by C. Y. Legault.

The following data are compiled on the following pages:

**Figure / Data**		**Page**
Figure S1. IRC forward		S31
Figure S2. IRC reverse		S31
Figure S3. Reaction coordinates (*E*)- and (*Z*)-isomers triflate counterion		S32
Figure S4. Reaction coordinates (*E*)- and (*Z*)-isomers trifluoro acetate counterion		S33
Figure S5. Reaction coordinate of rearrangement		S34
Figure S6. Comparison of iodine and selenium cyclization intermediates		S35
Matrices & thermodynamic data of optimized structures with triflates	B3LYP/6-31+G**/ LANLV2DZ	S36
Matrices & thermodynamic data of optimized structures with trifluoro acetic acid	B3LYP/6-31+G**	
Matrices & thermodynamic data of optimized structures with trifluoro acetic acid	B3LYP/6-31+G**/ LANLV2DZ	
Thermodynamic data of optimized structures with trifluoro acetic acid	SCRF=(PCM, CH_3CN)// B3LYP/6-31+G**	

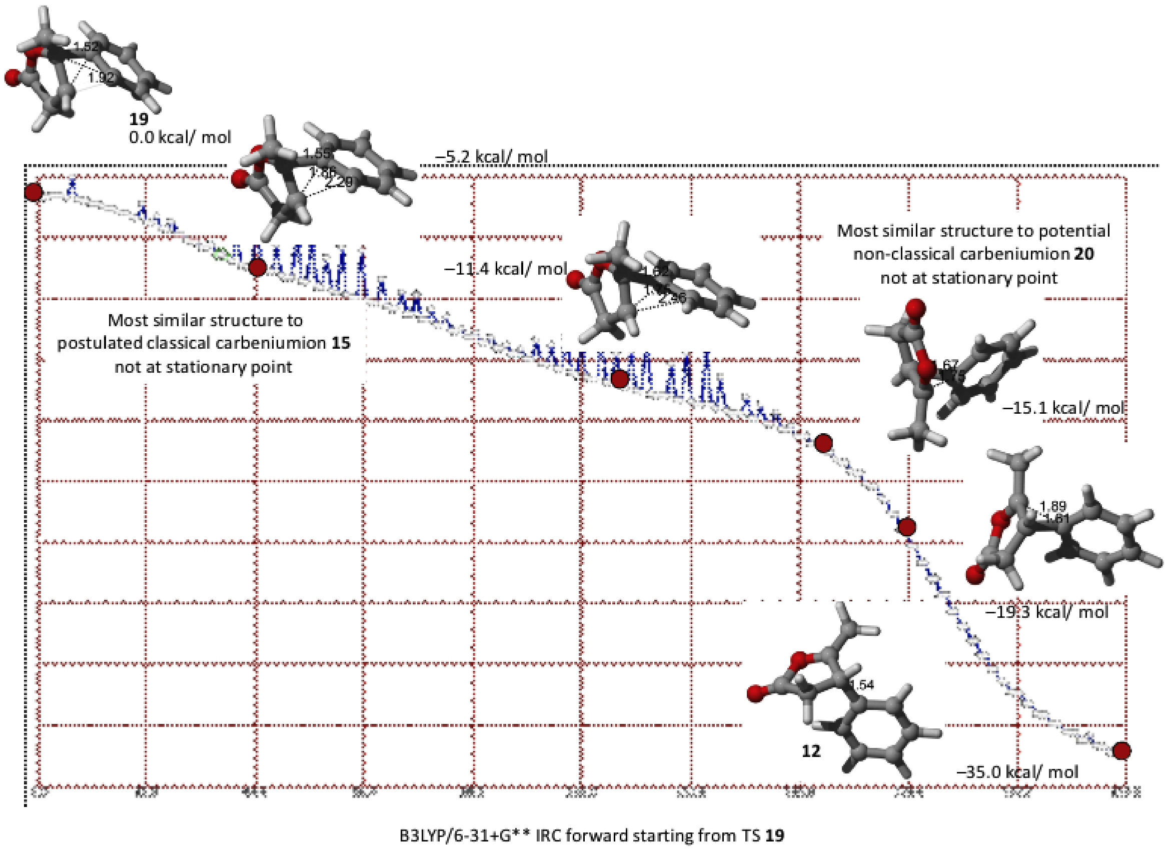

Figure S1: IRC calculation, forward

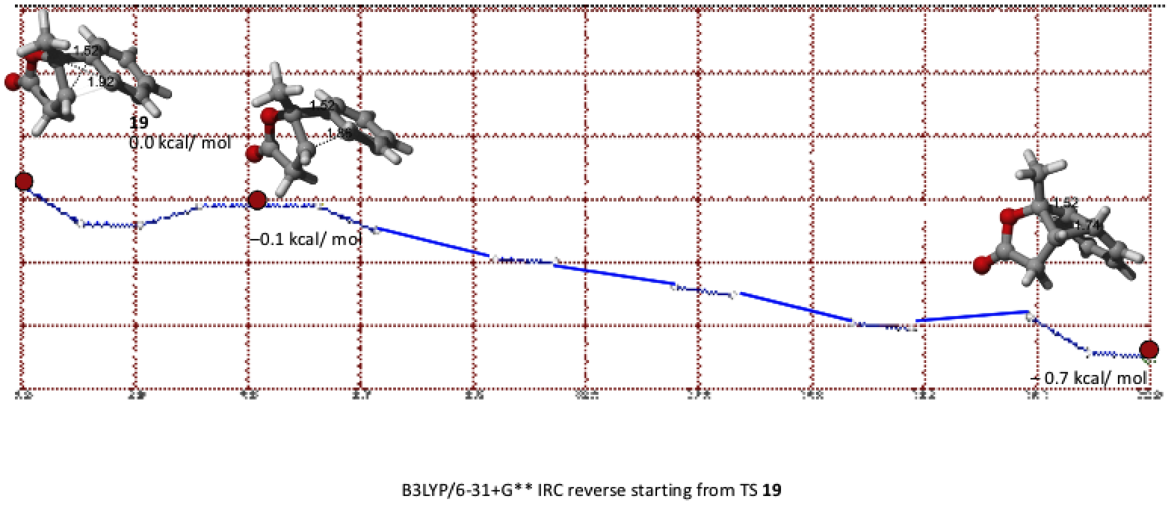

Figure S2: IRC calculation, reverse

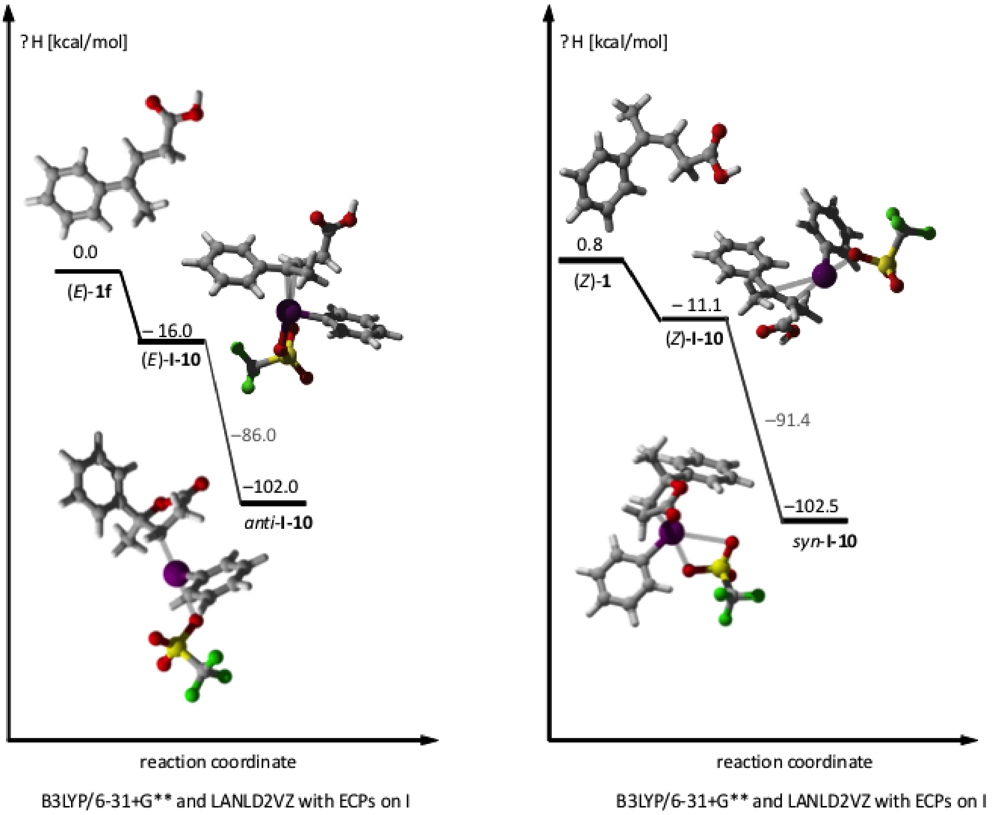

Figure S3: Reaction coordinates (*E*)- and (*Z*)-isomers triflate counterion

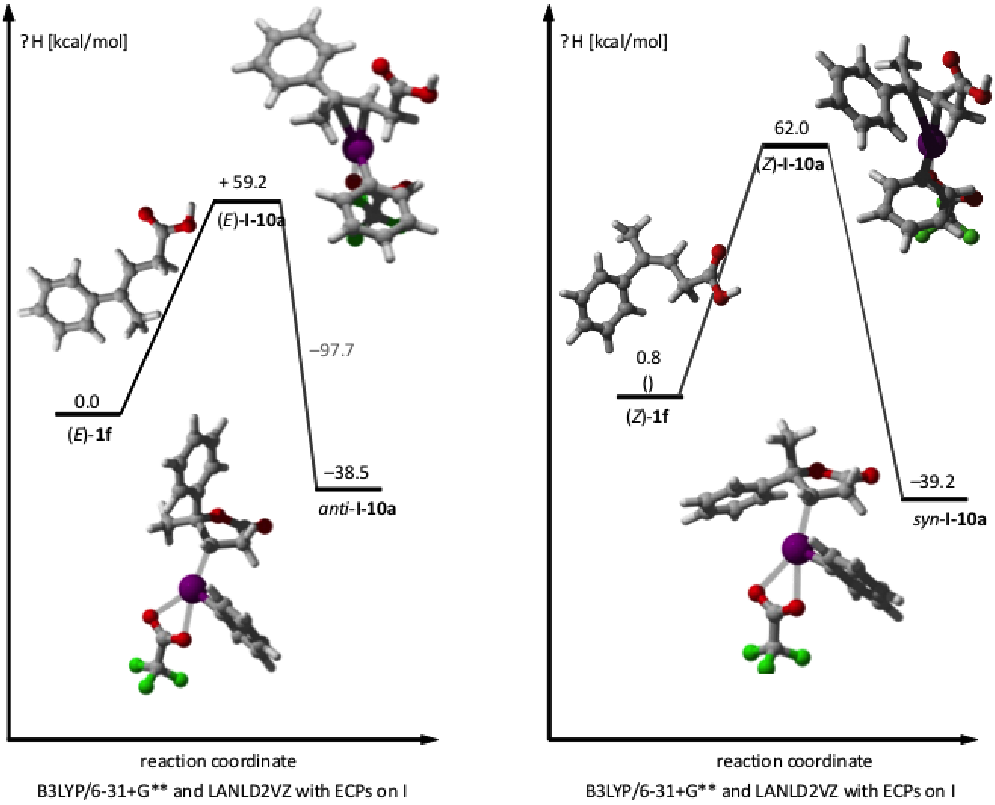

Figure S4: Reaction coordinates (*E*)- and (*Z*)-isomers trifluoro acetate counterion

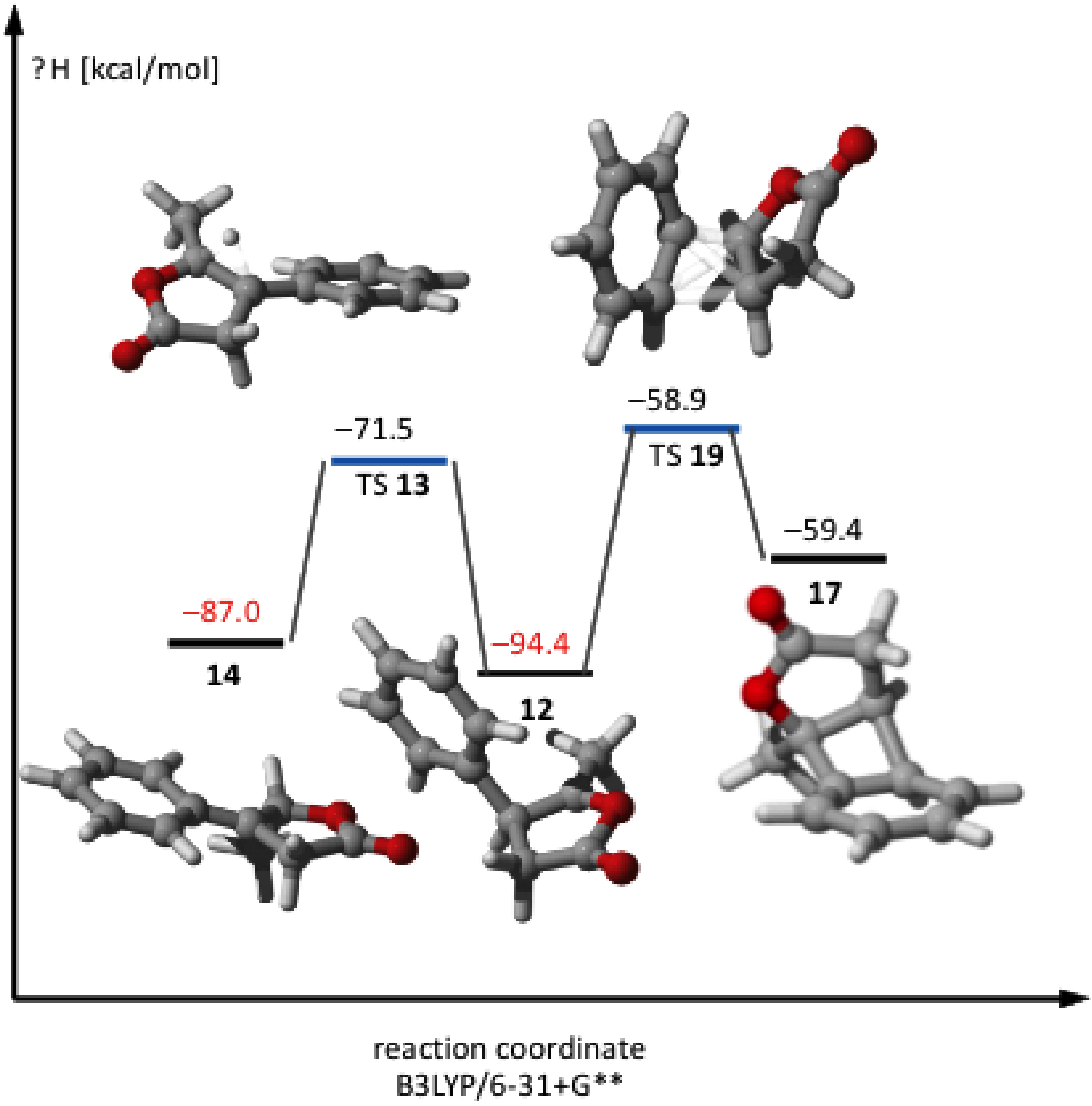

Figure S5: Reaction coordinate of rearrangement

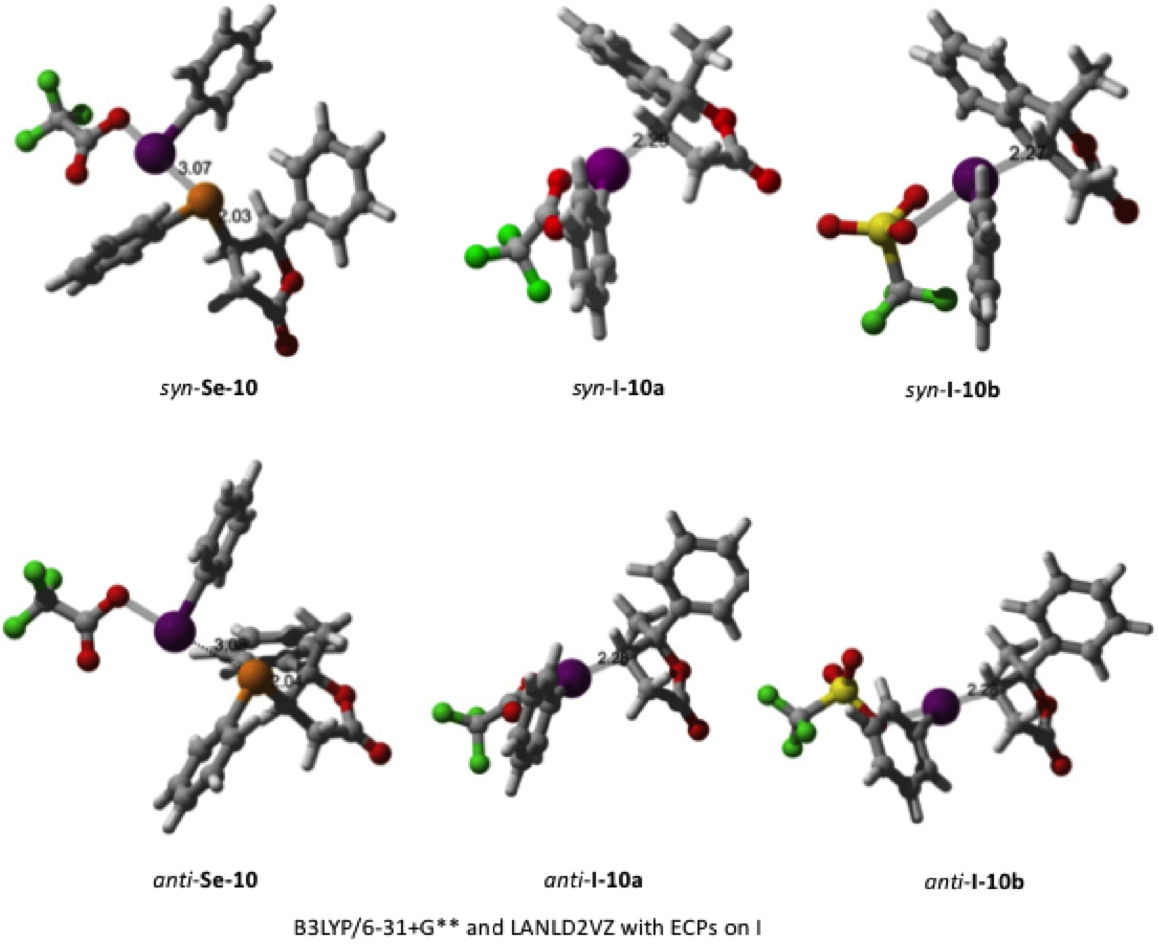

Figure S6: Comparison of iodine and selenium cyclization intermediates

B3LYP/6-31+G**/LANL2DVZ (for I)

	**PhI(OTf)$_2$**	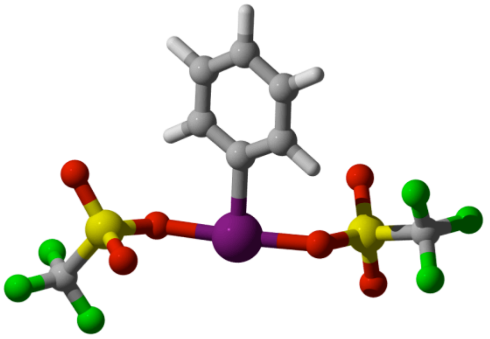

```
 Zero-point correction=                           0.139466
(Hartree/Particle)
 Thermal correction to Energy=                    0.165237
 Thermal correction to Enthalpy=                  0.166182
 Thermal correction to Gibbs Free Energy=         0.075775
 Sum of electronic and zero-point Energies=          -2164.909361
 Sum of electronic and thermal Energies=             -2164.883589
 Sum of electronic and thermal Enthalpies=           -2164.882645
 Sum of electronic and thermal Free Energies=        -2164.973051

                     E (Thermal)             CV                S
                      KCal/Mol        Cal/Mol-Kelvin    Cal/Mol-
Kelvin
 Total                  103.688              87.012
190.276

28
XYZ file generated by gabedit : coordinates in Angstrom
F         -5.9617020000       -2.0090510000        0.6647910000
O         -4.2177580000        0.7684630000        0.1169870000
F         -5.1216670000       -1.8809060000       -1.4093050000
C         -4.8309990000       -2.0878540000       -0.0960000000
S         -3.5810110000       -0.6699730000        0.5088800000
O         -3.1295860000       -0.9980820000        2.0353240000
F         -4.2179250000       -3.2929600000        0.0836310000
O         -2.2873390000       -1.0982420000       -0.6206070000
H         -1.7118030000        1.0944460000       -1.2939770000
I         -0.2215980000       -1.3908880000       -0.0160360000
O          3.0998010000       -0.7128430000       -1.8171850000
C         -0.8680560000        1.5484690000       -0.7920980000
C          0.0611810000        0.8002010000       -0.0839760000
H         -1.3907440000        3.5547870000       -1.3532160000
S          3.1300330000       -1.8735000000       -0.6815880000
O          1.8746260000       -1.5946700000        0.5336880000
C         -0.6802890000        2.9393870000       -0.8128260000
F          4.4210860000       -0.2372860000        1.1016840000
C          1.1524660000        1.3264380000        0.5929760000
C          4.6113500000       -1.4877890000        0.5828300000
H          1.8582960000        0.6973780000        1.1180570000
```

O	3.2055270000	-3.4359400000	-1.1065480000
F	4.6044550000	-2.4265960000	1.5667680000
C	0.4047550000	3.5197790000	-0.1482180000
C	1.3160440000	2.7189030000	0.5490630000
F	5.7826900000	-1.5323540000	-0.1169670000
H	0.5399950000	4.5954940000	-0.1725200000
H	2.1581610000	3.1651870000	1.0663970000

	**(*E*)-I-10**	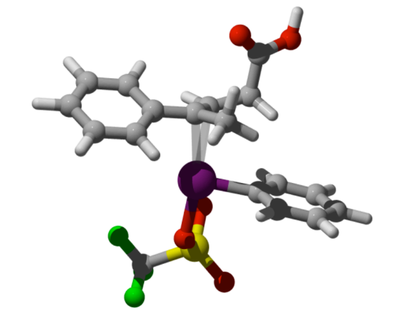

Zero-point correction= 0.323447
(Hartree/Particle)
Thermal correction to Energy= 0.353360
Thermal correction to Enthalpy= 0.354305
Thermal correction to Gibbs Free Energy= 0.254164
Sum of electronic and zero-point Energies= -1780.094483
Sum of electronic and thermal Energies= -1780.064570
Sum of electronic and thermal Enthalpies= -1780.063625
Sum of electronic and thermal Free Energies= -1780.163766

E (Thermal) CV S
KCal/Mol Cal/Mol-Kelvin Cal/Mol-
Kelvin
Total 221.737 105.178
210.763

45
XYZ file generated by gabedit : coordinates in Angstrom

F	4.4044450000	-2.9809680000	-1.1774350000
O	3.9044140000	0.0884850000	-1.8688070000
O	5.5292900000	0.6068940000	0.3831520000
F	5.7594820000	-2.5963300000	0.5712070000
C	5.3581350000	-2.1611760000	-0.6502830000
S	4.5236200000	-0.3653460000	-0.4287180000
H	-2.8588440000	-4.5995590000	-1.8969810000
H	-2.4836380000	-2.1705770000	-1.9178500000
H	-1.1745370000	-0.5902610000	-1.7372100000
H	-0.7695810000	1.7978420000	-2.0117060000
H	2.2879770000	2.0971690000	-1.5015110000
O	3.1701250000	-0.8853370000	0.6105620000
C	-2.6789810000	-4.0764860000	-0.9647760000
F	6.4107530000	-2.0322940000	-1.5006400000
O	-2.9067640000	3.0430650000	-2.3270470000

C	-2.4406010000	-2.7051450000	-0.9761720000
H	-3.8149900000	3.2872480000	-2.6128300000
I	1.1130980000	-0.4037900000	-0.0221120000
C	-1.4626360000	1.5182370000	-1.2135450000
C	-1.3431360000	0.0502510000	-0.8736530000
C	-2.8776490000	1.8476990000	-1.6725650000
C	2.0155060000	2.4832420000	-0.5267520000
H	2.7414930000	4.4661480000	-0.9231620000
O	-3.8727050000	1.1491260000	-1.4633080000
H	-2.8893630000	-5.8451070000	0.2564220000
C	-2.7072560000	-4.7760770000	0.2506890000
H	-1.2336810000	2.1778150000	-0.3673690000
C	2.2862390000	3.8153220000	-0.1854860000
C	1.4451580000	1.6698700000	0.4559770000
C	-2.2404000000	-1.9919510000	0.2381010000
C	-2.0070610000	-0.5498180000	0.2216940000
C	-2.5124120000	-4.0921360000	1.4596810000
C	-2.2955140000	-2.7167990000	1.4580540000
C	1.9908530000	4.2939370000	1.0969460000
C	1.1532580000	2.1086310000	1.7508310000
C	-2.4977890000	0.2953710000	1.3613120000
H	-2.5313120000	-4.6333790000	2.3986380000
H	-1.9166520000	1.2112480000	1.4925000000
H	-3.5298230000	0.5929370000	1.1248600000
H	0.7511320000	1.4431180000	2.5047530000
H	2.2111400000	5.3246850000	1.3504950000
H	-2.1299110000	-2.2130280000	2.4026720000
C	1.4303530000	3.4459780000	2.0612970000
H	-2.5337430000	-0.2415960000	2.3099460000
H	1.2225200000	3.8128590000	3.0601220000

	(*Z*)-I-10	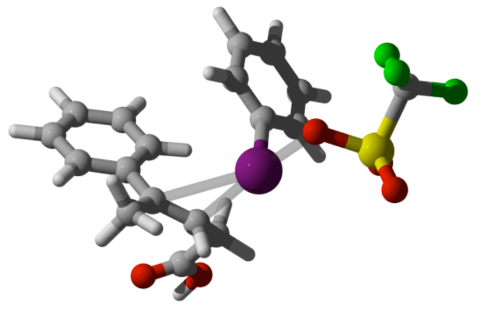

Zero-point correction= 0.323683 (Hartree/Particle)
Thermal correction to Energy= 0.353534
Thermal correction to Enthalpy= 0.354478
Thermal correction to Gibbs Free Energy= 0.254468
Sum of electronic and zero-point Energies= -1780.086448
Sum of electronic and thermal Energies= -1780.056596
Sum of electronic and thermal Enthalpies= -1780.055652
Sum of electronic and thermal Free Energies= -1780.155662

	E (Thermal)	CV	S
	KCal/Mol	Cal/Mol-Kelvin	Cal/Mol-Kelvin

Total 221.846 105.115
210.489

45
XYZ file generated by gabedit : coordinates in Angstrom

H	5.1428450000	1.0893320000	-2.5999950000
H	4.7955690000	2.8511650000	-0.8759370000
C	4.3327810000	0.9984190000	-1.8857140000
H	3.6686540000	-0.8631610000	-2.6872190000
C	4.1384810000	1.9891310000	-0.9121420000
H	1.5983350000	-1.6595140000	-3.2261710000
C	3.4880960000	-0.1051590000	-1.9363780000
H	-0.8017520000	1.6076110000	-1.6748350000
H	-0.7080030000	3.8990460000	-0.7329670000
C	3.0912920000	1.8646670000	0.0127980000
C	1.6245350000	-2.2790130000	-2.3264700000
C	2.4350570000	-0.2704750000	-0.9915870000
H	2.5466770000	-2.8767640000	-2.3583020000
H	2.9294100000	2.6369510000	0.7560880000
H	0.7941630000	-2.9877460000	-2.3630010000
C	2.2578210000	0.7511310000	-0.0176950000
C	-1.0348860000	1.7764680000	-0.6310000000
C	-0.9857610000	3.0689600000	-0.0931800000
C	1.6096360000	-1.4656920000	-1.0527240000
F	-5.6525730000	1.3595390000	-0.1962430000
O	-3.7272960000	-0.7993350000	-1.1120710000
H	1.4433250000	0.6976940000	0.6887160000
F	-6.6125990000	-0.0248450000	-1.6811800000
I	-1.6113030000	-1.2375250000	-0.6203690000
C	-1.4105550000	0.7331120000	0.2204570000
C	-6.2600780000	0.1551860000	-0.3832440000
C	-1.3092550000	3.2870250000	1.2522620000
H	-1.2778960000	4.2924520000	1.6570040000
C	0.8027310000	-1.9903190000	-0.0058760000
S	-4.9421930000	-1.2620300000	0.1072410000
H	0.4927500000	-3.0146010000	-0.2200030000
O	3.2820350000	-2.6345810000	1.1252870000
F	-7.3315530000	0.0185180000	0.4432350000
C	-1.7576010000	0.9160220000	1.5614150000
H	0.8510670000	-0.7594870000	1.8401320000
C	-1.6938130000	2.2191490000	2.0735570000
C	1.0104590000	-1.7787810000	1.4817720000
O	-5.5685910000	-2.7210970000	-0.2021820000
O	-4.3308740000	-0.9310860000	1.5821150000
C	2.4140070000	-2.2107790000	1.8900860000
H	-2.1197690000	0.1002170000	2.1736620000
H	-1.9679320000	2.3928330000	3.1079080000
H	0.3021060000	-2.3989840000	2.0435920000
O	2.5818200000	-2.0682810000	3.2368610000
H	3.4706460000	-2.3653750000	3.5322760000

	anti-**I-10**	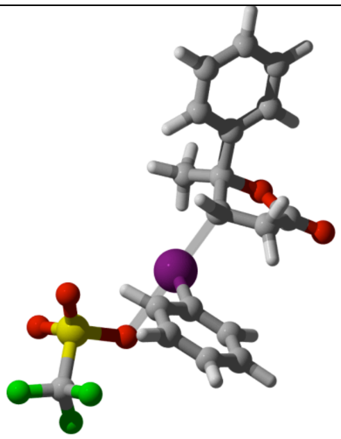

Zero-point correction= 0.313696
(Hartree/Particle)
Thermal correction to Energy= 0.341622
Thermal correction to Enthalpy= 0.342566
Thermal correction to Gibbs Free Energy= 0.249562
Sum of electronic and zero-point Energies= -1779.755136
Sum of electronic and thermal Energies= -1779.727210
Sum of electronic and thermal Enthalpies= -1779.726266
Sum of electronic and thermal Free Energies= -1779.819269

	E (Thermal)	CV	S
	KCal/Mol	Cal/Mol-Kelvin	Cal/Mol-Kelvin
Total	214.371	101.312	195.743

44
XYZ file generated by gabedit : coordinates in Angstrom

H	-6.2956070000	-1.4068930000	2.4382500000
H	-4.0263630000	-2.1233820000	3.1727000000
C	-5.4497840000	-1.3105580000	1.7656570000
C	-4.1737000000	-1.7119380000	2.1795520000
H	-6.6148550000	-0.4856840000	0.1477840000
C	-5.6279630000	-0.7910380000	0.4797580000
C	-3.0840310000	-1.5862640000	1.3134810000
H	-2.1024830000	-1.9117280000	1.6475860000
C	-4.5388330000	-0.6655360000	-0.3914790000
C	-3.2561990000	-1.0555280000	0.0222050000
H	-1.1617930000	-2.9034500000	-0.5147690000
H	-4.6846990000	-0.2804040000	-1.3931350000
H	-2.4269410000	-2.9076580000	-1.7549080000
H	-0.8964520000	-0.0916670000	0.7661380000
C	-1.5764430000	-2.3465920000	-1.3605270000
C	-2.0497070000	-0.9591110000	-0.9239370000
H	1.4856830000	2.5923270000	-1.6930400000
C	-0.9976350000	0.0170340000	-0.3116740000
H	-2.2138190000	1.7863510000	-0.0615720000
H	-0.8304910000	-2.2813230000	-2.1591070000
H	2.4300290000	4.5728950000	-0.5396990000
O	-2.4547580000	-0.2509350000	-2.1731350000
C	-1.4735800000	1.3904010000	-0.7720630000

C	1.8998360000	2.4937660000	-0.6966710000
C	2.4480480000	3.6074630000	-0.0457710000
C	-2.1716950000	1.1156560000	-2.0948130000
I	1.1150280000	-0.4662710000	-1.0286810000
H	-0.7006030000	2.1509660000	-0.8799590000
C	1.9420930000	1.2639600000	-0.0366810000
O	-2.4737640000	1.8991350000	-2.9810390000
O	3.5724600000	-0.8708410000	-1.5400070000
C	3.0309210000	3.4675290000	1.2189220000
H	3.4606360000	4.3322970000	1.7129060000
O	3.6371980000	-1.8569560000	1.0301100000
O	4.8125520000	-3.3269980000	-1.0470150000
S	4.3635900000	-1.8822340000	-0.4468090000
C	2.5417540000	1.0835760000	1.2078640000
C	3.0856540000	2.2129040000	1.8364110000
F	6.8540510000	-1.5434520000	0.6636260000
H	2.6527560000	0.0945860000	1.6387670000
C	6.0228060000	-0.8581280000	-0.1846870000
F	6.6494520000	-0.6626050000	-1.3854680000
H	3.5678170000	2.0969630000	2.8007680000
F	5.7098300000	0.3593690000	0.3741450000

	anti-**Se-10**	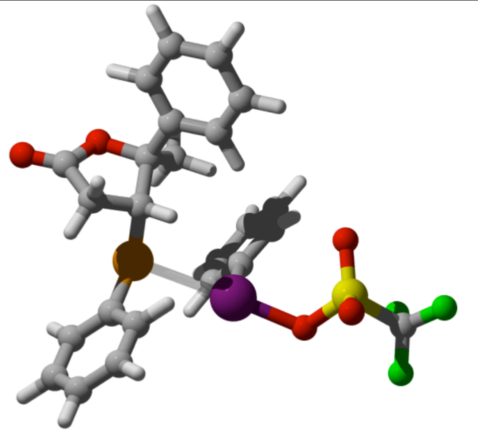

Zero-point correction= 0.406458
(Hartree/Particle)
Thermal correction to Energy= 0.441297
Thermal correction to Enthalpy= 0.442241
Thermal correction to Gibbs Free Energy= 0.330607
Sum of electronic and zero-point Energies= -4409.912683
Sum of electronic and thermal Energies= -4409.877844
Sum of electronic and thermal Enthalpies= -4409.876900
Sum of electronic and thermal Free Energies= -4409.988534

	E (Thermal)	CV	S
	KCal/Mol	Cal/Mol-Kelvin	Cal/Mol-Kelvin
Total	276.918	126.141	234.954

56
XYZ file generated by gabedit : coordinates in Angstrom

F	6.6060830000	-0.3726350000	0.2005340000
O	3.8420800000	-1.6361180000	-0.8103680000
F	6.8686570000	1.0230920000	-1.5405930000
C	6.4380440000	-0.1954000000	-1.1349520000
F	7.0257340000	-1.1940570000	-1.8434730000
S	4.4677070000	-0.3001180000	-1.5013540000
O	4.1533790000	0.0143070000	-3.0572780000
H	-0.3351320000	-4.1496910000	-2.8096970000
H	-2.4155700000	-5.5150350000	-2.8869460000
O	4.0465340000	1.1129810000	-0.4728500000
C	-1.1827270000	-3.8879530000	-2.1854290000
C	-2.3523670000	-4.6568680000	-2.2268380000
H	2.8474040000	-1.5242700000	1.2455690000
H	-0.7993470000	4.0208330000	-3.7967200000
I	1.9395550000	1.2627020000	0.0856080000
H	-0.1796720000	-2.2072400000	-1.3071260000
C	-1.1028120000	-2.7815450000	-1.3353030000
C	-3.4346480000	-4.3171790000	-1.4090690000
H	-4.3400850000	-4.9140460000	-1.4286990000
H	-0.4825630000	2.0679900000	-2.3207090000
C	2.7104820000	-0.9109590000	2.1289410000
H	3.2918090000	-2.4541010000	3.5056950000
C	2.3182020000	0.4250030000	2.0489980000
C	-1.0837560000	4.0878800000	-2.7524820000
C	2.9767830000	-1.4214050000	3.4075350000
C	-0.8997870000	2.9836980000	-1.9135380000
C	-2.1929620000	-2.4295580000	-0.5181120000
C	-3.3584090000	-3.2099620000	-0.5551480000
C	2.1968380000	1.2755650000	3.1492080000
H	-1.7709570000	6.1271820000	-2.8979440000
C	2.8461950000	-0.6083730000	4.5404180000
H	-1.2243210000	-0.0904400000	-1.2795940000
H	1.9166250000	2.3165260000	3.0465690000
C	-1.6311510000	5.2735830000	-2.2443330000
C	2.4605680000	0.7325780000	4.4132170000
H	3.0541420000	-1.0166740000	5.5229410000
C	-1.2720540000	3.0774230000	-0.5655480000
H	-4.1927560000	-2.9643540000	0.0901780000
H	2.3737780000	1.3652150000	5.2893150000
C	-1.8813050000	0.0635170000	-0.4258350000
Se	-1.0219380000	1.5856290000	
0.6811290000			
C	-2.0724320000	-1.2297330000	0.4319600000
C	-2.0002820000	5.3571370000	-0.8969380000
H	-0.0680600000	-1.6614150000	1.1571880000
C	-1.8228090000	4.2573690000	-0.0488700000
O	-3.3751920000	-0.9785980000	1.1005450000
H	-3.6307870000	-0.0603230000	-1.6889480000
C	-1.0730090000	-1.4959720000	1.5559370000
H	-2.4305090000	6.2711350000	-0.5033890000
C	-3.3141880000	0.4688550000	-0.7801370000
H	-1.3770210000	-2.4005530000	2.0877840000
H	-2.1181240000	4.3194960000	0.9926540000
C	-4.1376960000	-0.0375470000	0.3967460000
H	-1.0577420000	-0.6746530000	2.2790930000
H	-3.4814300000	1.5333510000	-0.9481850000

O	-5.2670170000	0.2737530000	0.7286550000

	syn-**I-10**	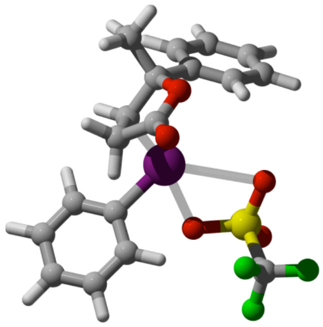

Zero-point correction= 0.313327
(Hartree/Particle)
Thermal correction to Energy= 0.341455
Thermal correction to Enthalpy= 0.342399
Thermal correction to Gibbs Free Energy= 0.248698
Sum of electronic and zero-point Energies= -1779.755975
Sum of electronic and thermal Energies= -1779.727846
Sum of electronic and thermal Enthalpies= -1779.726902
Sum of electronic and thermal Free Energies= -1779.820603

	E (Thermal)	CV	S
	KCal/Mol	Cal/Mol-Kelvin	Cal/Mol-Kelvin
Total	214.266	101.515	197.210

44
XYZ file generated by gabedit : coordinates in Angstrom

H	2.2701620000	-3.5316020000	-2.9648200000
H	-0.7764270000	4.8345350000	-2.4147580000
H	2.9911720000	-1.3773770000	-2.0130750000
H	0.1190710000	2.5324390000	-2.2355370000
C	2.0370510000	-3.2987030000	-1.9310230000
C	-1.1835170000	4.1581140000	-1.6707850000
H	4.2726920000	-0.0518950000	-0.7838780000
C	2.4464430000	-2.0783370000	-1.3877700000
C	-0.6689440000	2.8588150000	-1.5664570000
H	1.9761740000	1.2360620000	-0.8825330000
H	0.9865990000	-5.1514710000	-1.5768640000
C	1.3196890000	-4.2123360000	-1.1488890000
H	-2.6246080000	5.5760390000	-0.9226340000
C	-2.2256620000	4.5714200000	-0.8330880000
F	-5.6902320000	-0.7564670000	0.7915890000
C	4.0297740000	-0.0691650000	0.2819820000
C	-1.2102510000	2.0053250000	-0.6042380000
H	4.2865480000	0.9036180000	0.7131620000
C	1.6370210000	0.7882100000	0.0508920000
C	2.1465550000	-1.7583990000	-0.0487130000
I	-0.4419740000	-0.0023870000	-0.4125820000
H	4.6411400000	-0.8338360000	0.7675170000
C	2.5434520000	-0.3981590000	0.5097560000

O	-2.9414900000	-0.3570840000	-0.8282370000
O	-4.4537800000	-2.6471030000	-1.3723540000
S	-3.4449780000	-1.8920910000	-0.3442480000
O	-2.1885850000	-2.7375650000	0.2942570000
C	-2.7647400000	3.6876600000	0.1085560000
H	2.3961650000	2.4817900000	1.1901930000
C	1.0217010000	-3.9031000000	0.1809920000
C	-4.5476370000	-1.3852670000	1.2103960000
C	-2.2612010000	2.3855490000	0.2308640000
F	-4.8719890000	-2.4948960000	1.9441420000
C	1.5973160000	1.7295780000	1.2504970000
C	1.4401360000	-2.6875570000	0.7353840000
H	0.6619080000	2.2770230000	1.3843060000
H	-3.5841650000	3.9998610000	0.7465200000
H	0.4481500000	-4.5945180000	0.7868800000
H	-2.7041890000	1.6841010000	0.9256130000
O	2.3333140000	-0.4086120000	1.9871640000
F	-3.8214060000	-0.5161930000	2.0036680000
H	1.2208870000	-2.4557860000	1.7694990000
C	1.8626770000	0.8214950000	2.4458860000
O	1.7199750000	1.0682730000	3.6327550000

	**LG-1**	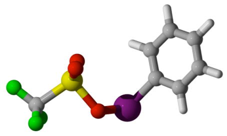

Zero-point correction= 0.114816
(Hartree/Particle)
Thermal correction to Energy= 0.130907
Thermal correction to Enthalpy= 0.131851
Thermal correction to Gibbs Free Energy= 0.066495
Sum of electronic and zero-point Energies= -1203.575913
Sum of electronic and thermal Energies= -1203.559822
Sum of electronic and thermal Enthalpies= -1203.558878
Sum of electronic and thermal Free Energies= -1203.624234

	E (Thermal)	CV	S
	KCal/Mol	Cal/Mol-Kelvin	Cal/Mol-Kelvin
Total	82.145	54.724	137.554

20
XYZ file generated by gabedit : coordinates in Angstrom

H	2.6625030000	1.8908040000	-1.7615860000
O	-2.4114910000	1.6172170000	0.9013980000
H	0.7509780000	0.3142290000	-1.9123770000
C	2.5380350000	1.1246200000	-1.0052730000
O	-1.7529110000	-1.1379440000	0.7904850000
C	1.4653090000	0.2356840000	-1.1016260000
S	-2.6012780000	0.3491590000	-0.0702510000
O	-2.0427820000	0.3233090000	-1.5932600000

H	4.2703700000	1.7269930000	0.1286560000
C	3.4421420000	1.0302520000	0.0649380000
F	-4.7794640000	-0.5435520000	1.3522360000
C	1.3406650000	-0.7402880000	-0.0982280000
C	-4.4837790000	-0.4375020000	0.0416570000
F	-5.3016160000	0.4171570000	-0.6093270000
I	-0.2657260000	-2.1096890000	-0.2446330000
C	3.2844200000	0.0494540000	1.0571370000
C	2.2194440000	-0.8514940000	0.9926810000
F	-4.4069150000	-1.6408570000	-0.5743700000
H	3.9841310000	-0.0122270000	1.8824500000
H	2.0879680000	-1.6053240000	1.7592970000

	**Triflate-anion**	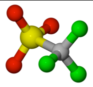

Zero-point correction= 0.026469
(Hartree/Particle)
Thermal correction to Energy= 0.033740
Thermal correction to Enthalpy= 0.034685
Thermal correction to Gibbs Free Energy= -0.006092
Sum of electronic and zero-point Energies= -961.526913
Sum of electronic and thermal Energies= -961.519642
Sum of electronic and thermal Enthalpies= -961.518697
Sum of electronic and thermal Free Energies= -961.559474

E (Thermal) CV S
KCal/Mol Cal/Mol-Kelvin Cal/Mol-
Kelvin
Total 21.172 24.775
85.822

8
XYZ file generated by gabedit : coordinates in Angstrom

O	-1.3340470000	-0.8345460000	-1.1832820000
S	-1.0184880000	-0.0000190000	0.0000490000
O	-1.3339050000	1.4420530000	-0.1313810000
O	-1.3341570000	-0.6073770000	1.3145470000
F	1.3821760000	0.5356250000	-1.1373800000
C	0.8740090000	0.0000370000	0.0000890000
F	1.3821670000	-1.2531480000	0.1047280000
F	1.3822450000	0.7173760000	1.0326330000

	**$HOSO_2CF_3$**	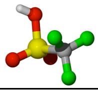

Zero-point correction= 0.038212
(Hartree/Particle)
Thermal correction to Energy= 0.046062

```
 Thermal correction to Enthalpy=                 0.047006
 Thermal correction to Gibbs Free Energy=        0.005157
 Sum of electronic and zero-point Energies=          -962.001835
 Sum of electronic and thermal Energies=             -961.993985
 Sum of electronic and thermal Enthalpies=           -961.993041
 Sum of electronic and thermal Free Energies=        -962.034890

                     E (Thermal)             CV                S
                      KCal/Mol        Cal/Mol-Kelvin    Cal/Mol-
Kelvin
 Total                   28.904             27.025
88.079


9
XYZ file generated by gabedit : coordinates in Angstrom
O         -1.1857660000         -0.0423340000          1.4838850000
S         -0.7910810000         -0.3454860000          0.1160850000
H         -1.3488890000          1.6957440000         -0.2896730000
O         -1.1871580000          0.9101870000         -0.8432930000
O         -1.1579570000         -1.5557580000         -0.5865890000
F          1.6242680000         -1.1303100000          0.8198020000
C          1.0866130000         -0.1940620000          0.0371680000
F          1.4490370000          1.0181070000          0.4774890000
F          1.5109370000         -0.3560920000         -1.2148700000
```

B3LYP/6-31+G**	(*Z*)-**1f**	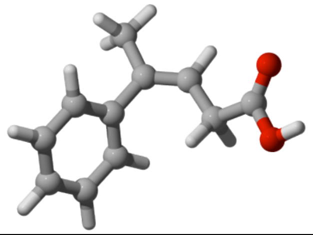

```
25
XYZ file generated by gabedit : coordinates in Angstrom
C           0.2023520000         1.2400410000        -0.2130650000
C           1.4763810000         0.8574670000        -0.4122560000
C           1.9980020000        -0.5525580000        -0.4410040000
C           3.3399580000        -0.6997900000         0.2492350000
O           3.6892600000        -2.0079330000         0.3662890000
C          -0.1641920000         2.7079000000        -0.1642910000
C          -0.9259030000         0.2757030000        -0.0374250000
C          -1.7409070000         0.3229780000         1.1077780000
C          -2.8024950000        -0.5687890000         1.2710560000
C          -3.0820200000        -1.5193850000         0.2847240000
C          -2.2895450000        -1.5708060000        -0.8643140000
C          -1.2232840000        -0.6815800000        -1.0224310000
O           4.0554620000         0.1969100000         0.6413630000
H           2.2330890000         1.6284750000        -0.5378200000
H           2.1560180000        -0.9023710000        -1.4732070000
H           1.2991990000        -1.2665550000         0.0047940000
H           4.5625890000        -2.0322000000         0.7931120000
H          -0.8967470000         2.9543380000        -0.9427870000
H           0.7163210000         3.3415910000        -0.3015840000
H          -0.6250380000         2.9723080000         0.7950800000
H          -1.5314860000         1.0520020000         1.8855090000
H          -3.4111240000        -0.5216480000         2.1697420000
H          -3.9110660000        -2.2098050000         0.4097040000
H          -2.5026160000        -2.2993510000        -1.6415940000
H          -0.6222080000        -0.7169360000        -1.9266000000

Zero-point correction=                           0.204470
(Hartree/Particle)
 Thermal correction to Energy=                    0.216928
 Thermal correction to Enthalpy=                  0.217872
 Thermal correction to Gibbs Free Energy=         0.163386
 Sum of electronic and zero-point Energies=           -576.687386
 Sum of electronic and thermal Energies=              -576.674928
 Sum of electronic and thermal Enthalpies=            -576.673984
 Sum of electronic and thermal Free Energies=         -576.728470

                     E (Thermal)             CV                S
                      KCal/Mol        Cal/Mol-Kelvin    Cal/Mol-
Kelvin
 Total                  136.124             46.310
114.675
```

B3LYP/6-31+G**	(*E*)-**1f**	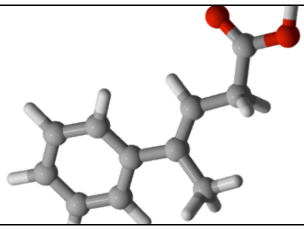

```
25
XYZ file generated by gabedit : coordinates in Angstrom
C        -0.1650010000         0.6360770000         0.0619430000
C        -1.2516640000        -0.0809900000        -0.2858570000
C        -2.6786370000         0.3936320000        -0.2860670000
C        -3.6784040000        -0.7311470000        -0.1029230000
O        -4.9368740000        -0.2449200000         0.0571780000
C        -0.2282640000         2.0886520000         0.4830280000
C         1.1881170000         0.0097500000         0.0170000000
C         1.3737270000        -1.3508730000         0.3288150000
C         2.6381670000        -1.9373530000         0.2748450000
C         3.7554180000        -1.1765740000        -0.0823500000
C         3.5923110000         0.1782260000        -0.3795890000
C         2.3257700000         0.7643060000        -0.3245840000
O        -3.4406810000        -1.9196780000        -0.1102370000
H        -1.1197650000        -1.1033700000        -0.6277030000
H        -2.9413490000         0.8763560000        -1.2408540000
H        -2.8834950000         1.1478430000         0.4805570000
H        -5.5297420000        -1.0104320000         0.1467810000
H         0.1335580000         2.7501000000        -0.3145680000
H         0.4018480000         2.2707500000         1.3601940000
H        -1.2450320000         2.4072440000         0.7221620000
H         0.5216260000        -1.9478520000         0.6387970000
H         2.7525310000        -2.9880950000         0.5263520000
H         4.7407470000        -1.6319630000        -0.1193360000
H         4.4514580000         0.7828960000        -0.6565650000
H         2.2236280000         1.8174120000        -0.5670260000
```

```
 Zero-point correction=                           0.204479
(Hartree/Particle)
 Thermal correction to Energy=                    0.217002
 Thermal correction to Enthalpy=                  0.217946
 Thermal correction to Gibbs Free Energy=         0.162983
 Sum of electronic and zero-point Energies=           -576.687676
 Sum of electronic and thermal Energies=              -576.675153
 Sum of electronic and thermal Enthalpies=            -576.674209
 Sum of electronic and thermal Free Energies=         -576.729171

                     E (Thermal)            CV                S
                      KCal/Mol        Cal/Mol-Kelvin    Cal/Mol-
Kelvin
 Total                  136.171            46.363
115.679
```

B3LYP/6-31+G**	$(PHSe)_2$	

24

```
XYZ file generated by gabedit : coordinates in Angstrom
H        -0.9312330000        0.4716400000        -1.5612010000
H        -2.4767510000        2.3200110000        -0.9658620000
C        -1.7572990000        0.2917650000        -0.8809070000
C        -2.6293810000        1.3305400000        -0.5442620000
Se        -0.7933600000       -2.4262820000        -
0.8819250000
C        -1.9430140000       -0.9851170000        -0.3371650000
C        -3.6978460000        1.0944120000         0.3246940000
H        -4.3787160000        1.9006150000         0.5820900000
C        -3.0222630000       -1.2225640000         0.5233670000
C        -3.8932300000       -0.1840930000         0.8572200000
H        -3.1768550000       -2.2167020000         0.9304080000
H        -4.7273130000       -0.3743000000         1.5266620000
H         4.7272000000       -0.3742930000        -1.5267830000
H         3.1767430000       -2.2166750000        -0.9304440000
C         3.8931510000       -0.1840710000        -0.8573020000
C         3.0221800000       -1.2225350000        -0.5234000000
H         4.3787070000        1.9006170000        -0.5821550000
C         3.6978170000        1.0944280000        -0.3247660000
C         1.9429390000       -0.9850550000         0.3371280000
Se         0.7934210000       -2.4263260000
0.8819060000
C         2.6294390000        1.3305500000         0.5443050000
C         1.7573470000        0.2917990000         0.8809730000
H         2.4769000000        2.3200010000         0.9659850000
H         0.9314170000        0.4716420000         1.5614380000
```

```
 Zero-point correction=                           0.182788
(Hartree/Particle)
 Thermal correction to Energy=                    0.195831
 Thermal correction to Enthalpy=                  0.196775
 Thermal correction to Gibbs Free Energy=         0.139334
 Sum of electronic and zero-point Energies=          -5262.001084
 Sum of electronic and thermal Energies=             -5261.988041
 Sum of electronic and thermal Enthalpies=           -5261.987097
 Sum of electronic and thermal Free Energies=        -5262.044538

                     E (Thermal)             CV                S
                      KCal/Mol        Cal/Mol-Kelvin    Cal/Mol-
Kelvin
 Total                  122.886            47.874
120.894
```

B3LYP/6-31+G**	$PhSe^{-}$	

```
12
XYZ file generated by gabedit : coordinates in Angstrom
H         2.1524830000        1.3015590000        -0.0000230000
H        -2.1524830000        1.3015590000        -0.0000230000
H         2.1434720000       -1.1629400000        -0.0000360000
H        -2.1434720000       -1.1629400000        -0.0000360000
C        -1.2008510000        0.7704540000        -0.0000110000
C         1.2008510000        0.7704540000        -0.0000110000
```

```
Se          0.0000000000         -3.2629860000
0.0000280000
H          0.0000000000          2.5811640000          0.0000320000
C         -1.1995760000         -0.6245900000         -0.0000110000
C          1.1995760000         -0.6245900000         -0.0000110000
C          0.0000000000          1.4935710000          0.0000250000
C          0.0000000000         -1.3807180000          0.0000720000
```

```
 Zero-point correction=                           0.089675
(Hartree/Particle)
 Thermal correction to Energy=                    0.095321
 Thermal correction to Enthalpy=                  0.096265
 Thermal correction to Gibbs Free Energy=         0.058831
 Sum of electronic and zero-point Energies=          -2631.044422
 Sum of electronic and thermal Energies=             -2631.038776
 Sum of electronic and thermal Enthalpies=           -2631.037832
 Sum of electronic and thermal Free Energies=        -2631.075266

                     E (Thermal)             CV                S
                      KCal/Mol        Cal/Mol-Kelvin    Cal/Mol-
Kelvin
 Total                   59.815            21.314
78.786
```

B3LYP/6-31+G**	$^{-}OCOCF_3$	

```
7
XYZ file generated by gabedit : coordinates in Angstrom
O         -1.4689130000         -1.1447960000         -0.0001070000
C         -0.9952810000          0.0101490000         -0.0001340000
O         -1.5186530000          1.1419420000         -0.0001120000
C          0.5924880000          0.0109590000         -0.0000680000
F          1.1669820000          1.2469990000         -0.0034070000
F          1.1119750000         -0.6356820000         -1.0910290000
F          1.1114030000         -0.6295740000          1.0948570000
```

```
 Zero-point correction=                           0.025317
(Hartree/Particle)
 Thermal correction to Energy=                    0.031456
 Thermal correction to Enthalpy=                  0.032400
 Thermal correction to Gibbs Free Energy=        -0.006848
 Sum of electronic and zero-point Energies=           -526.275918
 Sum of electronic and thermal Energies=              -526.269779
 Sum of electronic and thermal Enthalpies=            -526.268835
 Sum of electronic and thermal Free Energies=         -526.308083

                     E (Thermal)             CV                S
                      KCal/Mol        Cal/Mol-Kelvin    Cal/Mol-
Kelvin
 Total                   19.739            19.949
82.604
```

B3LYP/6-31+G**	*anti*-**Se-10a**	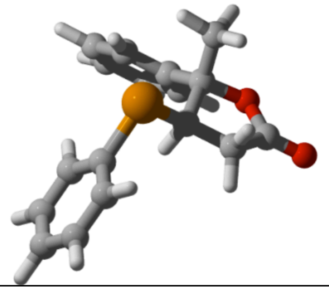

```
36
XYZ file generated by gabedit : coordinates in Angstrom
H          -4.4806700000        -3.3843970000        -0.6294690000
H          -2.3350940000        -3.6359570000         0.6124090000
C          -3.8782230000        -2.5115940000        -0.3950630000
C          -2.6737270000        -2.6523300000         0.2992620000
H          -5.2272410000        -1.1169270000        -1.3367560000
C          -4.2950670000        -1.2397620000        -0.7927190000
C          -1.9030730000        -1.5298450000         0.6066160000
H          -0.9716370000        -1.6545070000         1.1501780000
H           4.0528530000         1.0121410000         1.4895200000
C          -3.5227940000        -0.1137980000        -0.4909040000
C          -2.3129030000        -0.2491300000         0.2032520000
Se          1.2184130000         0.0883780000
1.5440250000
H          -3.8509270000         0.8692360000        -0.8077720000
H           6.0238570000         0.4940170000         0.0775010000
C           3.9590140000         0.3163240000         0.6617860000
H           0.7813420000         2.8670520000         0.5202760000
C           5.0676320000         0.0269080000        -0.1400300000
C           2.7232540000        -0.2796550000         0.3877530000
C          -1.4732790000         0.9701340000         0.5743100000
C           0.0236470000         0.8448100000         0.1452530000
C           0.3979830000         2.2534920000        -0.3035670000
C          -0.9283810000         2.8474720000        -0.7432300000
O          -1.9451570000         2.1012160000        -0.2299180000
O          -1.1279070000         3.8281160000        -1.4153890000
H          -1.4289830000         0.5274910000         2.7084070000
C           4.9446320000        -0.8501520000        -1.2199170000
H          -1.0439380000         2.2184190000         2.3147630000
C          -1.6718250000         1.3631800000         2.0457060000
H           0.0850160000         0.1522820000        -0.6966360000
C           2.6033670000        -1.1704690000        -0.6857890000
H           1.1322040000         2.2841480000        -1.1100450000
H           5.8050910000        -1.0699410000        -1.8448020000
C           3.7090840000        -1.4460480000        -1.4922520000
H           1.6540750000        -1.6570650000        -0.8884470000
H          -2.7188070000         1.6324140000         2.2071250000
H           3.6081820000        -2.1356690000        -2.3254490000
```

B3LYP/6-31+G**	*syn*-**Se-10a**	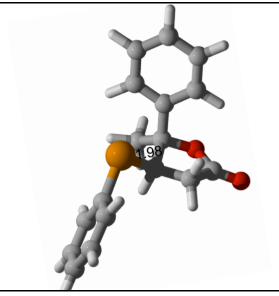

```
36
XYZ file generated by gabedit : coordinates in Angstrom
H          4.7773550000        -2.9862180000         -1.1531050000
H          3.2090970000        -3.5342560000          0.7020390000
C          4.0964440000        -2.2313840000         -0.7709340000
C          3.2156590000        -2.5384650000          0.2683220000
H          4.7939340000        -0.6812040000         -2.0995590000
C          4.1018420000        -0.9412580000         -1.3039280000
C          2.3443830000        -1.5665040000          0.7642640000
H          1.6726650000        -1.8310580000          1.5730770000
H         -2.9556720000        -1.1292380000         -2.2913770000
C          3.2305070000         0.0313220000         -0.8068860000
C          2.3233430000        -0.2715190000          0.2188860000
Se        -0.7422360000         -1.0109950000         -
0.3018500000
H          3.2830020000         1.0364390000         -1.2089310000
H         -5.3959060000        -0.6943260000         -2.2134180000
C         -3.4142810000        -0.7764710000         -1.3728660000
H          0.5392300000         1.1786280000         -1.9907370000
C         -4.7894660000        -0.5261320000         -1.3280930000
C         -2.6235100000        -0.5628540000         -0.2386190000
C          1.3676960000         0.7905660000          0.7766320000
C         -0.0313100000         0.7939880000          0.0853790000
C          0.1588780000         1.7142680000         -1.1132840000
C          1.2416410000         2.6686140000         -0.6362210000
O          1.8873330000         2.1165310000          0.4319960000
O          1.5479200000         3.7452580000         -1.0840800000
H          0.7190090000        -0.1375640000          2.6287200000
C         -5.3782210000        -0.0552560000         -0.1523680000
H          2.2519930000         0.7536930000          2.7600250000
C          1.2603350000         0.7539940000          2.3011830000
H         -0.7515230000         1.2414560000          0.7749520000
C         -3.2179640000        -0.0994010000          0.9417770000
H         -0.7413040000         2.2541970000         -1.4107360000
H         -6.4458960000         0.1402750000         -0.1179040000
C         -4.5893020000         0.1595040000          0.9824400000
H         -2.6174500000         0.0422440000          1.8356090000
H          0.7154530000         1.6367060000          2.6472650000
H         -5.0436660000         0.5164240000          1.9023390000

 Zero-point correction=                           0.287684
(Hartree/Particle)
 Thermal correction to Energy=                    0.305469
 Thermal correction to Enthalpy=                  0.306413
 Thermal correction to Gibbs Free Energy=         0.239461
```

Sum of electronic and zero-point Energies= -3207.095095
Sum of electronic and thermal Energies= -3207.077310
Sum of electronic and thermal Enthalpies= -3207.076366
Sum of electronic and thermal Free Energies= -3207.143318

	E (Thermal)	CV	S
	KCal/Mol	Cal/Mol-Kelvin	Cal/Mol-Kelvin
Total	191.685	68.707	140.913

B3LYP/6-31+G**	**12**	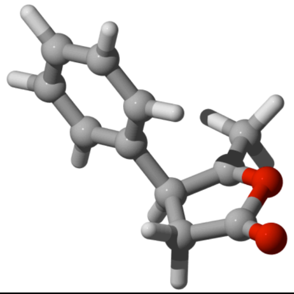

```
24
XYZ file generated by gabedit : coordinates in Angstrom
H          4.0996090000        -1.0839050000        -0.8779390000
H          3.8082740000         0.5282340000         0.9927730000
C          3.1032640000        -0.8193920000        -0.5388530000
C          2.9404450000         0.0907330000         0.5098460000
H          2.1057170000        -2.0991560000        -1.9622270000
C          1.9837070000        -1.3924180000        -1.1478830000
C          1.6601100000         0.4342500000         0.9481520000
H          1.5417240000         1.1334520000         1.7716840000
C          0.7011150000        -1.0549070000        -0.7112100000
C          0.5357400000        -0.1466990000         0.3467700000
H         -0.1552190000        -1.5159070000        -1.1990930000
H         -0.1820660000         2.5076050000        -0.6360470000
H         -0.7622290000         0.9434850000         1.6654360000
C         -0.8681820000         0.2337920000         0.8381090000
H         -1.2930050000        -1.8978090000         1.2869460000
C         -1.2025560000         2.2223760000        -0.8919030000
H         -1.3546830000         2.2169890000        -1.9746540000
C         -1.5669850000         0.9203470000        -0.3027580000
C         -1.8046450000        -0.9366580000         1.1926080000
H         -1.8940620000         2.9715700000        -0.4751050000
H         -2.3592510000        -0.7653020000         2.1230330000
O         -2.5555720000         0.2761030000        -0.7785350000
C         -2.8127440000        -1.0291290000         0.0866390000
O         -3.6685080000        -1.7376460000        -0.2657990000
```

Zero-point correction= 0.193478 (Hartree/Particle)
Thermal correction to Energy= 0.205148
Thermal correction to Enthalpy= 0.206092
Thermal correction to Gibbs Free Energy= 0.154144

Sum of electronic and zero-point Energies= -575.818208
Sum of electronic and thermal Energies= -575.806538
Sum of electronic and thermal Enthalpies= -575.805594
Sum of electronic and thermal Free Energies= -575.857542

	E (Thermal)	CV	S
	KCal/Mol	Cal/Mol-Kelvin	Cal/Mol-Kelvin
Total	128.732	43.941	109.334

B3LYP/6-31+G**	**14**	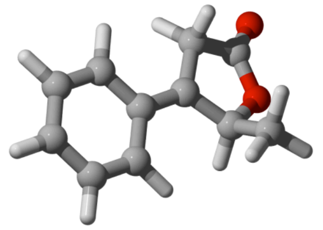

24
XYZ file generated by gabedit : coordinates in Angstrom

O	-4.0399680000	-1.5117640000	-0.4702280000
C	-3.0432100000	-0.8724300000	-0.3258500000
O	-2.9506770000	0.4697300000	-0.5645200000
H	-1.3961640000	-2.3235130000	-0.2900090000
C	-1.6757980000	-1.3693570000	0.1642510000
H	-1.7631050000	-1.5479350000	1.2490690000
C	-1.6223090000	0.9742530000	-0.3509450000
H	-1.3252220000	1.5037590000	-1.2633680000
H	-2.3806110000	2.7184990000	0.6421430000
C	-1.6316520000	1.9499000000	0.8459010000
H	-1.9048980000	1.4349380000	1.7706040000
C	-0.7594490000	-0.2387050000	-0.1107240000
H	-0.6562860000	2.4246470000	0.9742400000
H	0.7919570000	-2.3554730000	0.4962290000
C	0.6404280000	-0.2765490000	-0.1642860000
C	1.3448610000	-1.4736510000	0.1914520000
H	0.8921990000	1.7833890000	-0.8636450000
C	1.3990720000	0.8728490000	-0.5647800000
C	2.7253920000	-1.5076960000	0.1653090000
H	3.2563730000	-2.4121690000	0.4415390000
C	2.7789120000	0.8237230000	-0.5920390000
C	3.4416000000	-0.3623280000	-0.2264490000
H	3.3514880000	1.6915270000	-0.9009690000
H	4.5270570000	-0.3956430000	-0.2529360000

Zero-point correction= 0.194296 (Hartree/Particle)
Thermal correction to Energy= 0.205546
Thermal correction to Enthalpy= 0.206491
Thermal correction to Gibbs Free Energy= 0.156614
Sum of electronic and zero-point Energies= -575.806011
Sum of electronic and thermal Energies= -575.794761
Sum of electronic and thermal Enthalpies= -575.793817

```
 Sum of electronic and thermal Free Energies=         -575.843693

                     E (Thermal)             CV                S
                      KCal/Mol        Cal/Mol-Kelvin    Cal/Mol-
Kelvin
 Total                  128.982              43.363
104.974
```

B3LYP/6-31+G**	**2f**	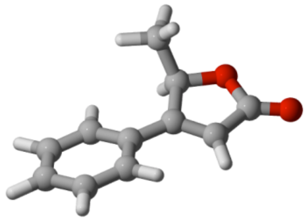

```
23
XYZ file generated by gabedit : coordinates in Angstrom
C          1.7493010000         0.9165530000         -0.2613120000
C          0.9136870000        -0.3525410000         -0.2117460000
C          1.7566060000        -1.4034560000         -0.2075430000
C          3.1481550000        -0.9239860000         -0.2772060000
O          3.1123800000         0.4528130000         -0.3195970000
C         -0.5552670000        -0.3592450000         -0.1769410000
O          4.1833520000        -1.5477260000         -0.3051410000
C         -1.2997230000         0.7104990000         -0.7084920000
C         -2.6948520000         0.6865030000         -0.6946110000
C         -3.3731820000        -0.4027830000         -0.1427280000
C         -2.6463150000        -1.4701450000          0.3951840000
C         -1.2534280000        -1.4487890000          0.3790020000
C          1.5867240000         1.8431380000          0.9430140000
H          1.5642520000         1.4706710000         -1.1911370000
H          1.5202500000        -2.4586850000         -0.1944440000
H         -0.7896320000         1.5584070000         -1.1550100000
H         -3.2509560000         1.5179640000         -1.1173120000
H         -4.4588280000        -0.4196370000         -0.1285310000
H         -3.1660020000        -2.3167170000          0.8339630000
H         -0.6979050000        -2.2720990000          0.8171700000
H          2.2852770000         2.6799180000          0.8609950000
H          1.7978780000         1.3008780000          1.8693330000
H          0.5682240000         2.2384720000          0.9930910000

Zero-point correction=                           0.183617
(Hartree/Particle)
 Thermal correction to Energy=                    0.194330
 Thermal correction to Enthalpy=                  0.195274
 Thermal correction to Gibbs Free Energy=         0.146519
 Sum of electronic and zero-point Energies=           -575.499435
 Sum of electronic and thermal Energies=              -575.488722
 Sum of electronic and thermal Enthalpies=            -575.487778
 Sum of electronic and thermal Free Energies=         -575.536532

                     E (Thermal)             CV                S
                      KCal/Mol        Cal/Mol-Kelvin    Cal/Mol-
Kelvin
 Total                  121.944              41.633
102.613
```

B3LYP/6-31+G**	**4f**	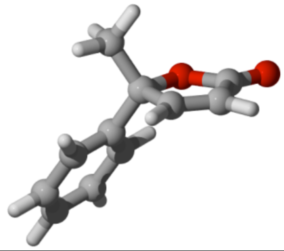

```
23
XYZ file generated by gabedit : coordinates in Angstrom
C        -0.9619440000        0.3972900000        0.3161240000
C        -1.5882730000        0.8508640000       -0.9879410000
C        -2.6467580000        0.0901560000       -1.2766390000
C        -2.8060340000       -0.9208010000       -0.2055710000
O        -1.8022300000       -0.7130830000        0.7139700000
O        -3.6293750000       -1.7949720000       -0.0797720000
C         0.4763130000       -0.0880280000        0.1086160000
C        -1.0567040000        1.4646580000        1.4189930000
C         1.4558900000        0.8125430000       -0.3376410000
C         2.7730660000        0.3934970000       -0.5312560000
C         3.1307740000       -0.9340140000       -0.2789130000
C         2.1611910000       -1.8338780000        0.1670110000
C         0.8406800000       -1.4162850000        0.3599320000
H        -1.1897850000        1.6768570000       -1.5656000000
H        -3.3116070000        0.1402300000       -2.1281800000
H        -0.6802540000        1.0530830000        2.3590550000
H        -2.0971990000        1.7724830000        1.5551160000
H        -0.4589000000        2.3429320000        1.1603960000
H         1.1975140000        1.8491560000       -0.5382670000
H         3.5177690000        1.1035780000       -0.8790780000
H         4.1553060000       -1.2616800000       -0.4283690000
H         2.4285850000       -2.8671990000        0.3684650000
H         0.0919700000       -2.1173820000        0.7095540000

 Zero-point correction=                           0.182974
(Hartree/Particle)
 Thermal correction to Energy=                    0.193592
 Thermal correction to Enthalpy=                  0.194537
 Thermal correction to Gibbs Free Energy=         0.146092
 Sum of electronic and zero-point Energies=          -575.489850
 Sum of electronic and thermal Energies=             -575.479231
 Sum of electronic and thermal Enthalpies=           -575.478287
 Sum of electronic and thermal Free Energies=        -575.526732

                     E (Thermal)            CV                 S
                      KCal/Mol       Cal/Mol-Kelvin    Cal/Mol-
Kelvin
 Total                  121.481            42.009
101.961
```

B3LYP/6-31+G**	**TS 19**	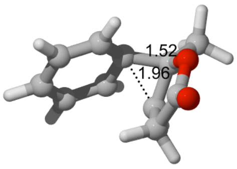

```
24
XYZ file generated by gabedit : coordinates in Angstrom
H          3.6516200000        -1.4437120000          0.1824440000
H          3.0028770000         0.5759120000          1.4709850000
C          2.7209030000        -0.9286950000         -0.0325430000
C          2.3530780000         0.1769930000          0.6986660000
H          2.2343720000        -2.2364450000         -1.6925870000
C          1.9075350000        -1.3803360000         -1.1090710000
C          1.1309710000         0.8595280000          0.3866440000
H          1.0518680000         1.8963800000          0.6997790000
C          0.6935590000        -0.7785170000         -1.4038060000
C          0.3098630000         0.3734250000         -0.6975390000
H          0.0290150000        -1.1885600000         -2.1574480000
H         -0.9803830000         2.9071580000         -0.4411610000
H         -0.6425730000         1.1686620000          1.8091950000
C         -0.6235340000         0.4154330000          1.0273130000
H         -0.2559210000        -1.7262650000          1.3931780000
C         -1.6822980000         2.1150100000         -0.7158940000
H         -1.8740880000         2.1806120000         -1.7896820000
C         -1.1260880000         0.7513120000         -0.3815910000
C         -1.0606830000        -0.9875680000          1.3202350000
H         -2.6238380000         2.2729970000         -0.1836470000
H         -1.6186000000        -1.0348640000          2.2616310000
O         -2.0203560000        -0.2879600000         -0.7569200000
C         -1.9837770000        -1.3456650000          0.1420970000
O         -2.5935230000        -2.3548270000         -0.0302800000


Zero-point correction=                           0.193662
(Hartree/Particle)
 Thermal correction to Energy=                    0.203840
 Thermal correction to Enthalpy=                  0.204784
 Thermal correction to Gibbs Free Energy=         0.158140
 Sum of electronic and zero-point Energies=           -575.758682
 Sum of electronic and thermal Energies=              -575.748505
 Sum of electronic and thermal Enthalpies=            -575.747561
 Sum of electronic and thermal Free Energies=         -575.794204

                     E (Thermal)             CV                S
                      KCal/Mol        Cal/Mol-Kelvin    Cal/Mol-
Kelvin
 Total                  127.911             41.415
98.170
```

NImag = 1, v(imag) = –196 cm^{-1}

B3LYP/6-31+G**	**TS 13**	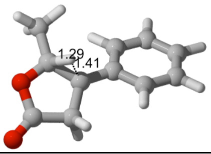

```
24
XYZ file generated by gabedit : coordinates in Angstrom
H          0.8322170000        1.7072610000        -0.9004040000
H          3.2866280000        1.6035740000        -1.0329630000
H         -2.2836800000        2.7536710000         0.9507080000
H         -1.5402490000        2.7642990000        -0.6551700000
C          1.3483480000        0.8295120000        -0.5295230000
C          2.7352190000        0.7664600000        -0.6180430000
C         -1.4902420000        2.3139730000         0.3425830000
H         -1.4456090000       -1.8972810000        -1.1975510000
H         -0.5188050000        2.5322880000         0.7867360000
O         -3.0278240000        0.4863880000         0.0125140000
C         -1.7284800000        0.8418750000         0.2133860000
O         -4.1655890000       -1.4356750000        -0.4556890000
C         -3.1228510000       -0.9096950000        -0.2629100000
C         -0.8321070000       -0.2621530000         0.0414650000
C          0.6239330000       -0.2687380000        -0.0139080000
C          3.4152630000       -0.3769200000        -0.1830170000
H          4.4979390000       -0.4180380000        -0.2487290000
C         -1.7071160000       -1.4662300000        -0.2237640000
C          1.3198490000       -1.4262590000         0.4025780000
C          2.7070190000       -1.4722070000         0.3264270000
H         -1.2878590000        0.2442450000         1.2734520000
H         -1.6322480000       -2.2729370000         0.5122890000
H          0.7789750000       -2.2792470000         0.8003750000
H          3.2372580000       -2.3581670000         0.6591600000

 Zero-point correction=                          0.190750
(Hartree/Particle)
 Thermal correction to Energy=                   0.201572
 Thermal correction to Enthalpy=                 0.202516
 Thermal correction to Gibbs Free Energy=        0.153676
 Sum of electronic and zero-point Energies=          -575.780850
 Sum of electronic and thermal Energies=             -575.770029
 Sum of electronic and thermal Enthalpies=           -575.769084
 Sum of electronic and thermal Free Energies=        -575.817924

                     E (Thermal)            CV                S
                      KCal/Mol        Cal/Mol-Kelvin    Cal/Mol-
Kelvin
 Total                  126.488            42.314
102.791
```

NImag = 1, v(imag) = –885cm^{-1}

B3LYP/6-31+G**	**CF_3COOH**	

```
8
XYZ file generated by gabedit : coordinates in Angstrom
```

O	-1.1947590000	-0.9518150000	-0.0000360000
C	-0.6097890000	0.2522850000	-0.0000470000
O	-1.1673170000	1.3179630000	-0.0000350000
C	0.9328350000	0.0918270000	-0.0000200000
F	1.5275980000	1.2882110000	-0.0005160000
F	1.3353620000	-0.5887940000	-1.0926200000
F	1.3352690000	-0.5878350000	1.0932320000
H	-2.1591980000	-0.8218450000	0.0000450000

Zero-point correction= 0.038708
(Hartree/Particle)
Thermal correction to Energy= 0.044984
Thermal correction to Enthalpy= 0.045929
Thermal correction to Gibbs Free Energy= 0.007195
Sum of electronic and zero-point Energies= -526.783769
Sum of electronic and thermal Energies= -526.777493
Sum of electronic and thermal Enthalpies= -526.776549
Sum of electronic and thermal Free Energies= -526.815282

	E (Thermal)	CV	S
	KCal/Mol	Cal/Mol-Kelvin	Cal/Mol-Kelvin
Total	28.228	21.059	81.521

B3LYP/6-31+G/LANL2DZ (for I)**

	**PhI(OCOCF$_3$)$_2$**	

26
XYZ file generated by gabedit : coordinates in Angstrom

C	-0.1395130000	1.1069950000	-0.1771270000
C	0.9067670000	1.6016210000	0.5937750000
C	0.9234330000	2.9839150000	0.8343090000
C	-0.0791040000	3.8099680000	0.3148150000
C	-1.1119860000	3.2645510000	-0.4548930000
C	-1.1556040000	1.8866290000	-0.7164050000
I	-0.2121170000	-1.0571440000	-0.5746340000
O	1.9623770000	-0.9274630000	-0.2425530000
C	2.6013920000	-2.0946730000	-0.3029390000
O	2.0953440000	-3.1922330000	-0.5589960000
C	4.0979340000	-1.9127750000	-0.0270810000
F	4.7291910000	-3.1269640000	0.1027870000
F	4.3009540000	-1.1909740000	1.1394090000
F	4.7005600000	-1.2179940000	-1.0643460000
O	-2.3630620000	-0.7339250000	-0.9171470000
C	-3.4718030000	-1.3266200000	-0.4540440000
O	-4.6072670000	-0.9132120000	-0.6763950000
C	-3.2481430000	-2.5842120000	0.3972810000
F	-2.3441460000	-3.4472700000	-0.2304840000
F	-4.4078260000	-3.2726750000	0.6268120000
F	-2.6875850000	-2.2446400000	1.6270680000
H	1.6914420000	0.9615390000	0.9717250000
H	1.7266670000	3.4016590000	1.4313870000
H	-0.0551490000	4.8766320000	0.5095980000
H	-1.8917660000	3.8997460000	-0.8605070000
H	-1.9609860000	1.4495110000	-1.2914190000

Zero-point correction= 0.145519 (Hartree/Particle)
Thermal correction to Energy= 0.166664
Thermal correction to Enthalpy= 0.167608
Thermal correction to Gibbs Free Energy= 0.087872
Sum of electronic and zero-point Energies= -1294.813756
Sum of electronic and thermal Energies= -1294.792611
Sum of electronic and thermal Enthalpies= -1294.791667
Sum of electronic and thermal Free Energies= -1294.871404

	E (Thermal)	CV	S
	KCal/Mol	Cal/Mol-Kelvin	Cal/Mol-Kelvin
Total	104.583	71.892	167.820

	(*E*)-**I-10a**	

44
XYZ file generated by gabedit : coordinates in Angstrom

C	-1.9360970000	-0.2892930000	0.1520150000
C	-1.2331350000	0.1867150000	-0.9835570000
C	-1.0862900000	1.6584750000	-1.3025770000
C	-2.4374800000	2.2621710000	-1.6635410000
O	-2.2790830000	3.4600330000	-2.2935250000
O	-3.5322720000	1.7558760000	-1.4036400000
C	-2.4861150000	-1.6406740000	0.1645560000
C	-2.6255460000	-2.3700040000	1.3758090000
C	-3.1495610000	-3.6595190000	1.3740400000
C	-3.5743510000	-4.2454320000	0.1722660000
C	-3.4674380000	-3.5350360000	-1.0326660000
C	-2.9234760000	-2.2541000000	-1.0424080000
C	-2.1435470000	0.6098470000	1.3355140000
I	1.1296480000	-0.7406700000	-0.4190810000
O	3.1703320000	-1.5931110000	-0.0993630000
C	4.1250030000	-1.1612400000	-0.9328920000
O	4.0115380000	-0.3011420000	-1.8099170000
C	5.4521560000	-1.8890720000	-0.6511620000
F	5.8605790000	-1.6545240000	0.6498180000
F	6.4340660000	-1.4490460000	-1.5036460000
F	5.2986470000	-3.2523940000	-0.8196400000
C	1.7930240000	1.1233570000	0.4418220000
C	2.4237190000	2.0463050000	-0.3955260000
C	2.8908670000	3.2330650000	0.1857190000
C	2.7292020000	3.4638140000	1.5576530000
C	2.1070880000	2.5076110000	2.3706350000
C	1.6309300000	1.3114760000	1.8174220000
H	-1.2850510000	-0.4467040000	-1.8675410000
H	-0.4064860000	1.8149470000	-2.1448800000
H	-0.6795610000	2.2430680000	-0.4685230000
H	-3.1396050000	3.8787540000	-2.5179230000
H	-2.2871230000	-1.9451240000	2.3131850000
H	-3.2292170000	-4.2102170000	2.3042380000
H	-3.9946890000	-5.2450890000	0.1762810000
H	-3.8237790000	-3.9760900000	-1.9564520000
H	-2.9080100000	-1.6956420000	-1.9709120000
H	-1.3440680000	1.3445670000	1.4579840000
H	-2.2746910000	0.0655110000	2.2710290000
H	-3.0765490000	1.1658250000	1.1601340000
H	2.5933730000	1.8404460000	-1.4441570000
H	3.3927430000	3.9635390000	-0.4385660000
H	3.1010340000	4.3828220000	1.9963650000
H	2.0021580000	2.6777870000	3.4362310000
H	1.1771200000	0.5581160000	2.4493780000

```
 Zero-point correction=                           0.326626
(Hartree/Particle)
 Thermal correction to Energy=                    0.354069
 Thermal correction to Enthalpy=                  0.355013
 Thermal correction to Gibbs Free Energy=         0.261584
 Sum of electronic and zero-point Energies=          -1345.049962
 Sum of electronic and thermal Energies=             -1345.022519
 Sum of electronic and thermal Enthalpies=           -1345.021575
 Sum of electronic and thermal Free Energies=        -1345.115004

                     E (Thermal)             CV                S
                      KCal/Mol        Cal/Mol-Kelvin    Cal/Mol-
Kelvin
 Total                  222.182             97.387
196.638
```

	(*Z*)-**I-10a**	

44
XYZ file generated by gabedit : coordinates in Angstrom

C	1.4787790000	-1.6978850000	-0.6770610000
C	0.6988180000	-1.8452160000	0.5057290000
C	0.9724070000	-1.1694570000	1.8354830000
C	2.3808370000	-1.5027020000	2.3136150000
O	2.5986870000	-0.9803510000	3.5548830000
O	3.2147190000	-2.1517380000	1.6793790000
C	2.2929240000	-0.5490100000	-1.0372780000
C	3.3318820000	-0.7088180000	-1.9981640000
C	4.1613350000	0.3530940000	-2.3426610000
C	3.9636910000	1.6159180000	-1.7659370000
C	2.9293700000	1.8068750000	-0.8374210000
C	2.1127600000	0.7426150000	-0.4694020000
C	1.4689680000	-2.8856550000	-1.6102710000
I	-1.7070420000	-1.3037320000	-0.2711320000
O	-3.8349320000	-0.9498050000	-0.8715430000
C	-4.7055730000	-0.7845950000	0.1320130000
O	-4.4526080000	-0.7634390000	1.3393080000
C	-6.1297700000	-0.5898200000	-0.4193840000
F	-6.1839410000	0.5510270000	-1.2020880000
F	-7.0314990000	-0.4568020000	0.6073320000
F	-6.5015710000	-1.6692430000	-1.1975410000
C	-1.5860440000	0.7806670000	0.2661600000
C	-1.9144050000	1.1406630000	1.5754510000
C	-1.9060710000	2.5058810000	1.8925950000
C	-1.5922320000	3.4594530000	0.9155770000
C	-1.2871840000	3.0627600000	-0.3927120000
C	-1.2825560000	1.7044420000	-0.7375130000
H	0.3919850000	-2.8825270000	0.6493320000
H	0.2718220000	-1.5416160000	2.5917720000
H	0.8599150000	-0.0836850000	1.8417810000

H	3.4945820000	-1.1970020000	3.8955670000
H	3.5134550000	-1.6770490000	-2.4459190000
H	4.9612320000	0.2011640000	-3.0579850000
H	4.6074940000	2.4445720000	-2.0397080000
H	2.7645280000	2.7864550000	-0.4033330000
H	1.3057080000	0.9280120000	0.2237740000
H	0.6394930000	-3.5633790000	-1.3950170000
H	2.3905580000	-3.4654670000	-1.4566410000
H	1.4268240000	-2.5986110000	-2.6635640000
H	-2.2157850000	0.4060180000	2.3106410000
H	-2.1664100000	2.8152120000	2.8984380000
H	-1.6021050000	4.5133290000	1.1699700000
H	-1.0667490000	3.8028680000	-1.1537650000
H	-1.0662890000	1.3965630000	-1.7527450000

Zero-point correction= 0.326772 (Hartree/Particle)
Thermal correction to Energy= 0.353269
Thermal correction to Enthalpy= 0.354214
Thermal correction to Gibbs Free Energy= 0.263854
Sum of electronic and zero-point Energies= -1345.042135
Sum of electronic and thermal Energies= -1345.015638
Sum of electronic and thermal Enthalpies= -1345.014694
Sum of electronic and thermal Free Energies= -1345.105054

	E (Thermal)	CV	S
	KCal/Mol	Cal/Mol-Kelvin	Cal/Mol-Kelvin
Total	221.680	95.363	190.179

	anti-**I-10a**	

43
XYZ file generated by gabedit : coordinates in Angstrom

C	1.5560070000	-1.3561030000	0.3471020000
C	0.7301520000	-0.0514490000	0.1183030000
C	1.0309830000	0.7951130000	1.3540960000
C	1.3499010000	-0.2242320000	2.4374300000
O	1.5988830000	-1.4594050000	1.8335100000
O	1.4099100000	-0.0642170000	3.6463490000
C	2.9832880000	-1.1610480000	-0.1865350000
C	3.1852390000	-0.9124030000	-1.5562400000
C	4.4768380000	-0.7651210000	-2.0694230000
C	5.5855320000	-0.8663710000	-1.2201110000
C	5.3917300000	-1.1236040000	0.1404800000
C	4.0986510000	-1.2740050000	0.6569310000
C	0.9572220000	-2.6748050000	-0.1498160000
I	-1.5068660000	-0.4885080000	-0.0424300000
O	-4.0054360000	-0.4971830000	-0.0499560000
C	-4.3666130000	-1.7472300000	-0.0933500000

O	-3.6183850000	-2.7508670000	-0.1779750000
C	-5.8911340000	-1.9341390000	-0.0349700000
F	-6.4269830000	-1.3422170000	1.1053820000
F	-6.2596170000	-3.2648000000	-0.0211990000
F	-6.5115240000	-1.3418470000	-1.1311110000
C	-1.9346400000	1.6335640000	-0.0994310000
C	-1.2422890000	2.4462070000	-0.9972320000
C	-1.5371700000	3.8163200000	-1.0191850000
C	-2.5156990000	4.3392730000	-0.1666510000
C	-3.2108700000	3.4956560000	0.7069410000
C	-2.9273880000	2.1235590000	0.7473640000
H	0.9776430000	0.4269250000	-0.8266930000
H	0.2293100000	1.4631870000	1.6740110000
H	1.9231530000	1.4157630000	1.1882200000
H	2.3365920000	-0.8426340000	-2.2317180000
H	4.6168180000	-0.5748170000	-3.1286310000
H	6.5885120000	-0.7509800000	-1.6175380000
H	6.2452410000	-1.2126430000	0.8046510000
H	3.9536770000	-1.4914310000	1.7078670000
H	1.6473130000	-3.4911510000	0.0765510000
H	0.8032190000	-2.6493790000	-1.2330970000
H	0.0090050000	-2.8998450000	0.3488420000
H	-0.5009700000	2.0425260000	-1.6766760000
H	-1.0087860000	4.4633840000	-1.7111750000
H	-2.7437120000	5.3993890000	-0.1915920000
H	-3.9824640000	3.8946230000	1.3561270000
H	-3.4942690000	1.4569540000	1.3825910000

```
Zero-point correction=                           0.316317
(Hartree/Particle)
 Thermal correction to Energy=                    0.342124
 Thermal correction to Enthalpy=                  0.343068
 Thermal correction to Gibbs Free Energy=         0.254050
 Sum of electronic and zero-point Energies=          -1344.705937
 Sum of electronic and thermal Energies=             -1344.680130
 Sum of electronic and thermal Enthalpies=           -1344.679186
 Sum of electronic and thermal Free Energies=        -1344.768203

                     E (Thermal)             CV                S
                      KCal/Mol        Cal/Mol-Kelvin    Cal/Mol-
Kelvin
 Total                  214.686             93.934
187.353
```

	anti-**Se-10a**	

55

XYZ file generated by gabedit : coordinates in Angstrom

C	-2.6125530000	-0.8681530000	-0.1348370000
C	-2.2912230000	0.6579130000	-0.2586130000

C	-3.6147100000	1.3850710000	0.0147730000
C	-4.6781210000	0.3542380000	-0.3534040000
O	-4.0856140000	-0.9004830000	-0.4349330000
O	-5.8685460000	0.5360340000	-0.5333470000
C	-1.9430030000	-1.7575920000	-1.1747980000
C	-2.5380760000	-2.9937650000	-1.4854880000
C	-1.9411190000	-3.8557700000	-2.4089630000
C	-0.7386210000	-3.5013040000	-3.0337900000
C	-0.1415790000	-2.2726420000	-2.7354090000
C	-0.7432570000	-1.4059880000	-1.8123140000
C	-2.4663010000	-1.4299690000	1.2869320000
Se	-0.8818910000	1.3311560000	1.0574850000
I	2.0201770000	0.5469760000	0.3694850000
C	-0.7025380000	3.1684430000	0.3869070000
C	-0.3512050000	3.4284520000	-0.9438680000
C	-0.2229840000	4.7549940000	-1.3698320000
C	-0.4393830000	5.8062610000	-0.4693610000
C	-0.7858630000	5.5350010000	0.8593560000
C	-0.9191800000	4.2114190000	1.2959590000
C	1.5975110000	-2.4122510000	1.0592570000
C	1.9693590000	-1.1913700000	1.6287190000
C	2.3692400000	-1.0487450000	2.9601540000
C	2.3888000000	-2.1981130000	3.7590930000
C	2.0178760000	-3.4382820000	3.2227870000
C	1.6242700000	-3.5452070000	1.8825260000
O	4.1544920000	-0.0033570000	0.0226120000
C	4.6837480000	0.8505100000	-0.8559700000
O	4.0790030000	1.7916550000	-1.3850520000
C	6.1601620000	0.5299160000	-1.1435160000
F	6.9006010000	0.6300210000	0.0182670000
F	6.6674470000	1.4034520000	-2.0699750000
F	6.2796440000	-0.7571870000	-1.6314150000
H	-1.9140900000	0.8926090000	-1.2539260000
H	-3.7393940000	2.2957660000	-0.5731410000
H	-3.7522550000	1.6486400000	1.0707500000
H	-3.4837710000	-3.2607050000	-1.0285530000
H	-2.4203650000	-4.7986600000	-2.6487520000
H	-0.2805060000	-4.1689060000	-3.7553150000
H	0.7774940000	-1.9786970000	-3.2322390000
H	1.3002530000	-2.4946380000	0.0207820000
H	-2.9239140000	-2.4208770000	1.3261900000
H	-2.9676480000	-0.7941090000	2.0239430000
H	-1.4111490000	-1.5363820000	1.5613200000
H	-0.1785300000	2.6212370000	-1.6483770000
H	0.0495950000	4.9633930000	-2.3983400000
H	-0.3366200000	6.8326240000	-0.8031130000
H	-0.9562280000	6.3474920000	1.5567270000
H	-1.1924880000	4.0013590000	2.3239950000

H	-0.2704090000	-0.4509710000	-1.6113840000
H	2.6671820000	-0.0913260000	3.3690230000
H	2.7006280000	-2.1190380000	4.7942990000
H	2.0403320000	-4.3230000000	3.8490070000
H	1.3453000000	-4.5071360000	1.4676960000

```
 Zero-point correction=                           0.409678
(Hartree/Particle)
 Thermal correction to Energy=                    0.443124
 Thermal correction to Enthalpy=                  0.444068
 Thermal correction to Gibbs Free Energy=         0.335121
 Sum of electronic and zero-point Energies=          -3974.863981
 Sum of electronic and thermal Energies=             -3974.830535
 Sum of electronic and thermal Enthalpies=           -3974.829591
 Sum of electronic and thermal Free Energies=        -3974.938538

                    E (Thermal)            CV                S
                     KCal/Mol        Cal/Mol-Kelvin    Cal/Mol-
Kelvin
 Total                 278.065           120.396
229.298
```

	syn-**I-10a**	

43

XYZ file generated by gabedit : coordinates in Angstrom

C	2.5295390000	-0.4248680000	0.1876320000
C	1.5568050000	0.7421870000	-0.1711970000
C	1.8213480000	1.8000820000	0.8970300000
C	2.3396790000	1.0056700000	2.0887810000
O	2.6771130000	-0.2792570000	1.6662980000
O	2.4751990000	1.3704930000	3.2465550000
C	2.0025290000	-1.8228730000	-0.1092520000
C	1.9026580000	-2.2589780000	-1.4450690000
C	1.3934960000	-3.5261890000	-1.7395390000
C	0.9782240000	-4.3746020000	-0.7062890000
C	1.0811300000	-3.9507900000	0.6214200000
C	1.5954690000	-2.6845390000	0.9235090000
C	3.9253410000	-0.1861420000	-0.4168170000
I	-0.6011160000	-0.0046170000	-0.0544310000
O	-3.0609910000	-0.2739490000	0.2193500000
C	-3.3157190000	-1.5423890000	0.3695730000
O	-2.5003710000	-2.4936140000	0.3323260000
C	-4.8059390000	-1.8246410000	0.6234080000
F	-5.2508860000	-1.1755050000	1.7725630000
F	-5.0676000000	-3.1716450000	0.7850550000
F	-5.5948360000	-1.3724730000	-0.4297170000
C	-1.3181590000	2.0225080000	-0.2758550000
C	-0.9086140000	2.7744400000	-1.3778430000
C	-1.3867390000	4.0852870000	-1.5100090000

C	-2.2671750000	4.6121420000	-0.5587330000
C	-2.6809180000	3.8291560000	0.5248550000
C	-2.2098460000	2.5178780000	0.6749230000
H	1.6824890000	1.0827630000	-1.1986470000
H	0.9549560000	2.3996660000	1.1841680000
H	2.6002800000	2.5066550000	0.5772120000
H	2.2148770000	-1.6113840000	-2.2593530000
H	1.3146430000	-3.8470580000	-2.7730070000
H	0.5694680000	-5.3524730000	-0.9359980000
H	0.7535380000	-4.5993550000	1.4262750000
H	1.6874660000	-2.3602320000	1.9520080000
H	4.6248630000	-0.9153250000	-0.0001700000
H	4.2896750000	0.8185920000	-0.1796830000
H	3.9065210000	-0.3023100000	-1.5040550000
H	-0.2484770000	2.3624330000	-2.1324340000
H	-1.0787060000	4.6815580000	-2.3623870000
H	-2.6391120000	5.6251590000	-0.6683540000
H	-3.3779310000	4.2274860000	1.2539860000
H	-2.5641700000	1.8910740000	1.4819040000

```
Zero-point correction=                           0.316019
(Hartree/Particle)
 Thermal correction to Energy=                    0.341971
 Thermal correction to Enthalpy=                  0.342915
 Thermal correction to Gibbs Free Energy=         0.253272
 Sum of electronic and zero-point Energies=          -1344.707291
 Sum of electronic and thermal Energies=             -1344.681338
 Sum of electronic and thermal Enthalpies=           -1344.680394
 Sum of electronic and thermal Free Energies=        -1344.770037

                     E (Thermal)             CV                S
                      KCal/Mol        Cal/Mol-Kelvin    Cal/Mol-
Kelvin
 Total                  214.590            94.097
188.670
```

	syn-**Se-10a**	

55
XYZ file generated by gabedit : coordinates in Angstrom

C	2.1791560000	-0.1432470000	1.6777850000
C	1.5875230000	1.1546400000	1.0165700000
C	2.7830370000	2.0993980000	0.8714230000
C	3.6412820000	1.7215320000	2.0748720000
O	3.2476010000	0.4597400000	2.5307700000
O	4.5399170000	2.3554500000	2.5960620000
C	2.8208820000	-1.1196230000	0.6895720000
C	4.2085000000	-1.1327320000	0.4721780000
C	4.7748110000	-2.0246500000	-0.4462070000
C	3.9679380000	-2.9191640000	-1.1561150000
C	2.5866210000	-2.9261030000	-0.9337650000

C	2.0186470000	-2.0378020000	-0.0150820000
C	1.2134000000	-0.8127820000	2.6558650000
Se	0.6595020000	0.8767860000	-0.7658830000
I	-2.2479790000	0.1504970000	-0.0972400000
C	0.1570370000	2.7466750000	-1.1146570000
C	-0.5139930000	3.5258450000	-0.1630980000
C	-0.8591790000	4.8447190000	-0.4785280000
C	-0.5373690000	5.3744520000	-1.7349780000
C	0.1313700000	4.5873180000	-2.6794440000
C	0.4823940000	3.2669110000	-2.3735220000
C	-1.9907030000	-2.8467330000	0.5027510000
C	-2.0473220000	-1.9386600000	-0.5581620000
C	-2.0458580000	-2.3233060000	-1.9009010000
C	-1.9655660000	-3.6921000000	-2.1863340000
C	-1.9013780000	-4.6289350000	-1.1466380000
C	-1.9170580000	-4.2099930000	0.1902170000
O	-4.3897510000	-0.3644070000	0.3168100000
C	-5.0159100000	0.7394690000	0.7247320000
O	-4.4872570000	1.8528420000	0.8469810000
C	-6.4964800000	0.4736510000	1.0450350000
F	-7.1393220000	-0.0435610000	-0.0619220000
F	-7.1217290000	1.6346780000	1.4193190000
F	-6.5961270000	-0.4400970000	2.0776340000
H	0.8337690000	1.5839550000	1.6815260000
H	2.5223640000	3.1578910000	0.8960370000
H	3.3616260000	1.9070120000	-0.0404680000
H	4.8554490000	-0.4793560000	1.0435600000
H	5.8492390000	-2.0258190000	-0.5943650000
H	4.4110030000	-3.6118460000	-1.8632250000
H	1.9524340000	-3.6295780000	-1.4630170000
H	-2.0242590000	-2.5226240000	1.5356030000
H	1.7296630000	-1.6137920000	3.1895380000
H	0.3542190000	-1.2459500000	2.1341960000
H	0.8548520000	-0.0847810000	3.3891180000
H	-0.7678180000	3.1304040000	0.8153980000
H	-1.3805210000	5.4510050000	0.2537050000
H	-0.8080040000	6.3963530000	-1.9753840000
H	0.3848960000	4.9963220000	-3.6510020000
H	1.0067740000	2.6602150000	-3.1036100000
H	0.9490870000	-2.0877960000	0.1642700000
H	-2.1150340000	-1.5996270000	-2.7035670000
H	-1.9669400000	-4.0189960000	-3.2199870000
H	-1.8484130000	-5.6867530000	-1.3780190000
H	-1.8810150000	-4.9369670000	0.9935710000

```
 Zero-point correction=                           0.409478
(Hartree/Particle)
 Thermal correction to Energy=                    0.443050
 Thermal correction to Enthalpy=                  0.443994
```

Thermal correction to Gibbs Free Energy= 0.332221
Sum of electronic and zero-point Energies= -3974.864227
Sum of electronic and thermal Energies= -3974.830654
Sum of electronic and thermal Enthalpies= -3974.829710
Sum of electronic and thermal Free Energies= -3974.941484

	E (Thermal)	CV	S
	KCal/Mol	Cal/Mol-Kelvin	Cal/Mol-Kelvin
Total	278.018	120.457	235.248

	**Lg-IIa**	PhSeIph(OCOCF$_3$)

31
XYZ file generated by gabedit : coordinates in Angstrom

Se	-1.7907120000	-1.0294510000	-2.2211010000
I	0.8818120000	-0.7605800000	-1.3405960000
C	-2.6793230000	-1.4226470000	-0.5343570000
C	-3.2237090000	-0.3848030000	0.2386610000
C	-3.9046560000	-0.6796390000	1.4237370000
C	-4.0454950000	-2.0094340000	1.8406290000
C	-3.5049320000	-3.0460170000	1.0693670000
C	-2.8219880000	-2.7550920000	-0.1156100000
C	0.7082510000	1.3589400000	-0.7467280000
C	-0.3023000000	2.1251090000	-1.3176780000
C	-0.4036050000	3.4706330000	-0.9349700000
C	0.4931760000	4.0112380000	-0.0072360000
C	1.4994820000	3.2099030000	0.5419280000
C	1.6224110000	1.8607570000	0.1755180000
O	3.1605180000	-0.4449920000	-0.5424760000
C	3.7919220000	-1.5893110000	-0.6347970000
O	3.3579180000	-2.6628820000	-1.0965450000
C	5.2323370000	-1.4963460000	-0.1007110000
F	6.0061950000	-0.6686200000	-0.9053630000
F	5.8459920000	-2.7299960000	-0.0553410000
F	5.2580610000	-0.9652340000	1.1834940000
H	-3.1128810000	0.6432010000	-0.0867080000
H	-4.3233700000	0.1255560000	2.0185660000
H	-4.5738970000	-2.2364840000	2.7607880000
H	-3.6127160000	-4.0773340000	1.3887410000
H	-2.4017270000	-3.5540600000	-0.7157580000
H	-1.0043710000	1.6989650000	-2.0244590000
H	-1.1830300000	4.0867510000	-1.3709580000
H	0.4100430000	5.0530770000	0.2834340000
H	2.2015320000	3.6239870000	1.2577940000
H	2.4190580000	1.2447910000	0.5687270000

Zero-point correction= 0.211012 (Hartree/Particle)
Thermal correction to Energy= 0.232297
Thermal correction to Enthalpy= 0.233242
Thermal correction to Gibbs Free Energy= 0.152160
Sum of electronic and zero-point Energies= -3399.235329

```
 Sum of electronic and thermal Energies=           -3399.214043
 Sum of electronic and thermal Enthalpies=         -3399.213098
 Sum of electronic and thermal Free Energies=      -3399.294180

                     E (Thermal)             CV                S
                      KCal/Mol        Cal/Mol-Kelvin    Cal/Mol-
Kelvin
 Total                  145.769            73.215
170.651
```

	LG-Ia	

19
XYZ file generated by gabedit : coordinates in Angstrom

I	0.5589340000	-0.2431720000	0.0273950000
O	3.2498260000	-0.5799630000	0.0613380000
C	4.1024500000	0.3752240000	-0.0291900000
O	3.9546410000	1.6213680000	-0.1319840000
C	5.5642010000	-0.1561650000	0.0085820000
F	5.8664680000	-0.7977850000	1.2177660000
F	6.5310360000	0.8368030000	-0.1452230000
F	5.8212770000	-1.0963460000	-0.9974050000
C	-1.6046380000	-0.1050940000	0.0115370000
C	-2.3964800000	-1.2623330000	-0.0462320000
C	-3.7945360000	-1.1697950000	-0.0570520000
C	-4.4197980000	0.0819690000	-0.0099790000
C	-3.6348590000	1.2399700000	0.0478970000
C	-2.2369530000	1.1471810000	0.0584970000
H	-1.9204730000	-2.2370740000	-0.0829680000
H	-4.3929700000	-2.0762850000	-0.1023790000
H	-5.5038720000	0.1541750000	-0.0184480000
H	-4.1085340000	2.2177220000	0.0848130000
H	-1.6357160000	2.0496060000	0.1030370000

```
 Zero-point correction=                          0.116735
(Hartree/Particle)
 Thermal correction to Energy=                   0.130838
 Thermal correction to Enthalpy=                 0.131782
 Thermal correction to Gibbs Free Energy=        0.068794
 Sum of electronic and zero-point Energies=        -768.978908
 Sum of electronic and thermal Energies=           -768.964805
 Sum of electronic and thermal Enthalpies=         -768.963861
 Sum of electronic and thermal Free Energies=      -769.026848

                     E (Thermal)             CV                S
                      KCal/Mol        Cal/Mol-Kelvin    Cal/Mol-
Kelvin
 Total                   82.102            47.125
132.569
```

B3LYP/6-31+G** SCRF=PCM(CH_3CN)	(*Z*)-**1f**	

```
 Variational PCM results
 =======================
 <psi(f)|   H    |psi(f)>                          (a.u.) =    -
576.889235
 <psi(f)|H+V(f)/2|psi(f)>                          (a.u.) =    -
576.911642
 Total free energy in solution:
  with all non electrostatic terms                 (a.u.) =    -
576.894504
 --------------------------------------------------------------------
--
 (Polarized solute)-Solvent                    (kcal/mol) =     -14.06
 --------------------------------------------------------------------
--
 Cavitation energy                             (kcal/mol) =      23.32
 Dispersion energy                             (kcal/mol) =     -13.52
 Repulsion energy                              (kcal/mol) =       0.95
 Total non electrostatic                       (kcal/mol) =      10.75
 --------------------------------------------------------------------
--
 Partition over spheres:
 Sphere   on Atom   Surface  Charge   GEl     GCav    GDR
    1      C1        0.00   0.000    0.00    0.06    0.00
    2      C2        4.77  -0.010   -0.15    1.30   -0.45
    3      C3       15.74  -0.074   -0.49    2.43   -1.20
    4      C4        2.18  -0.013   -0.03    0.46   -0.16
    5      O5       21.85  -0.089   -6.15    2.58   -1.69
    6      C6       44.38  -0.026   -0.13    4.87   -2.34
    7      C7        0.00   0.000    0.00    0.08    0.00
    8      C8        9.63  -0.010   -0.35    1.71   -0.78
    9      C9       17.31  -0.018   -0.64    2.00   -1.32
   10      C10      17.43  -0.017   -0.64    2.00   -1.30
   11      C11      17.32  -0.018   -0.65    2.00   -1.32
   12      C12       8.20  -0.018   -0.37    1.62   -0.68
   13      O13      17.47   0.203   -3.11    2.21   -1.34
 Added spheres:    57.72   0.062   -1.34    0.00    0.00
 --------------------------------------------------------------------
--
 After PCM corrections, the SCF energy is  -576.911641700     a.u.
 --------------------------------------------------------------------
--
```

B3LYP/6-31+G** SCRF=PCM(CH_3CN)	(*E*)-**1f**	

```
Variational PCM results
 =======================
 <psi(f)|   H    |psi(f)>                          (a.u.) =    -
576.889463
 <psi(f)|H+V(f)/2|psi(f)>                          (a.u.) =    -
576.911799
 Total free energy in solution:
  with all non electrostatic terms                 (a.u.) =    -
576.895195
```

```
 ------------------------------------------------------------------
--
 (Polarized solute)-Solvent                (kcal/mol) =     -14.02
 ------------------------------------------------------------------
--
 Cavitation energy                         (kcal/mol) =      23.22
 Dispersion energy                         (kcal/mol) =     -13.74
 Repulsion energy                          (kcal/mol) =       0.94
 Total non electrostatic                   (kcal/mol) =      10.42
 ------------------------------------------------------------------
--
 Partition over spheres:
 Sphere   on Atom   Surface   Charge    GEl     GCav    GDR
    1       C1        0.01    0.000     0.00    0.10    0.00
    2       C2        5.51    0.007    -0.11    1.36   -0.50
    3       C3       16.48   -0.082    -0.66    2.39   -1.27
    4       C4        2.18   -0.013    -0.05    0.46   -0.15
    5       O5       21.84   -0.092    -6.23    2.58   -1.67
    6       C6       38.77   -0.036    -0.22    4.46   -2.15
    7       C7        0.27    0.001    -0.01    0.21   -0.01
    8       C8       12.29    0.001    -0.34    1.85   -1.00
    9       C9       17.29   -0.016    -0.60    1.99   -1.32
   10       C10      17.45   -0.015    -0.63    2.01   -1.29
   11       C11      17.30   -0.016    -0.64    1.99   -1.32
   12       C12       9.48    0.007    -0.28    1.60   -0.81
   13       O13      16.09    0.184    -2.57    2.21   -1.31
 Added spheres:      58.90    0.043    -1.68    0.00    0.00
 ------------------------------------------------------------------
--
 After PCM corrections, the SCF energy is  -576.911799331     a.u.
 ------------------------------------------------------------------
--
```

B3LYP/6-31+G** SCRF=PCM(CH_3CN)	**($(PhSe)_2$)**	

```
Variational PCM results
 =======================
 <psi(f)|   H    |psi(f)>                       (a.u.) =   -
5262.182348
 <psi(f)|H+V(f)/2|psi(f)>                       (a.u.) =   -
5262.195444
 Total free energy in solution:
  with all non electrostatic terms              (a.u.) =   -
5262.172492
 ------------------------------------------------------------------
--
 (Polarized solute)-Solvent                (kcal/mol) =      -8.22
 ------------------------------------------------------------------
--
 Cavitation energy                         (kcal/mol) =      25.80
 Dispersion energy                         (kcal/mol) =     -12.38
 Repulsion energy                          (kcal/mol) =       0.98
 Total non electrostatic                   (kcal/mol) =      14.40
 ------------------------------------------------------------------
--
 Partition over spheres:
```

```
 Sphere   on Atom  Surface  Charge   GEl     GCav    GDR
    1       C3      10.06  -0.022   -0.41    1.86   -0.63
    2       C4      16.45  -0.031   -0.71    2.00   -1.15
    3      Se5      21.72   0.045   -0.64    2.73   -0.49
    4       C6       0.67   0.002   -0.01    0.24   -0.01
    5       C7      17.24  -0.028   -0.73    2.00   -1.26
    6       C9      13.90  -0.025   -0.49    2.07   -0.91
    7       C10     17.16  -0.027   -0.70    2.00   -1.25
    8       C15     17.16  -0.027   -0.70    2.00   -1.25
    9       C16     14.01  -0.025   -0.49    2.07   -0.91
   10       C18     17.24  -0.028   -0.73    2.00   -1.26
   11       C19      0.71   0.002   -0.01    0.24   -0.01
   12      Se20     21.82   0.045   -0.65    2.73   -0.49
   13       C21     16.67  -0.031   -0.71    2.00   -1.15
   14       C22     10.11  -0.021   -0.40    1.87   -0.63
 Added spheres:    55.33   0.128   -0.87    0.00    0.00
 -----------------------------------------------------------------
--
 After PCM corrections, the SCF energy is  -5262.19544421     a.u.
 -----------------------------------------------------------------
--
```

B3LYP/6-31+G** SCRF=PCM(CH_3CN)	PhSe$^-$	

```
Variational PCM results
 =======================
 <psi(f)|   H    |psi(f)>                    (a.u.) =   -
2631.124435
 <psi(f)|H+V(f)/2|psi(f)>                    (a.u.) =   -
2631.224236
 Total free energy in solution:
  with all non electrostatic terms           (a.u.) =   -
2631.212073
 -----------------------------------------------------------------
--
 (Polarized solute)-Solvent              (kcal/mol) =     -62.63
 -----------------------------------------------------------------
--
 Cavitation energy                       (kcal/mol) =      14.39
 Dispersion energy                       (kcal/mol) =      -7.34
 Repulsion energy                        (kcal/mol) =       0.58
 Total non electrostatic                 (kcal/mol) =       7.63
 -----------------------------------------------------------------
--
 Partition over spheres:
 Sphere   on Atom  Surface  Charge   GEl     GCav    GDR
    1       C5      17.22   0.007   -0.99    1.99   -1.23
    2       C6      17.22   0.007   -0.99    1.99   -1.24
    3      Se7      32.86   0.559  -40.29    3.92   -0.71
    4       C9      15.26   0.030   -1.84    2.08   -1.23
    5       C10     15.26   0.030   -1.84    2.07   -1.05
    6       C11     17.68   0.012   -1.14    2.03   -1.26
    7       C12      0.88   0.008   -0.51    0.32   -0.04
 Added spheres:    29.12   0.253  -15.03    0.00    0.00
 -----------------------------------------------------------------
--
```

```
 After PCM corrections, the SCF energy is  -2631.22423635     a.u.
 -------------------------------------------------------------------
--
```

B3LYP/6-31+G** SCRF=PCM(CH_3CN)	**$^-OCOCF_3$**	

```
Variational PCM results
 =======================
 <psi(f)|   H    |psi(f)>                         (a.u.) =    -
526.299669
 <psi(f)|H+V(f)/2|psi(f)>                         (a.u.) =    -
526.393784
 Total free energy in solution:
  with all non electrostatic terms                (a.u.) =    -
526.384587
 -------------------------------------------------------------------
--
 (Polarized solute)-Solvent                   (kcal/mol) =     -59.06
 -------------------------------------------------------------------
--
 Cavitation energy                            (kcal/mol) =      12.26
 Dispersion energy                            (kcal/mol) =      -7.07
 Repulsion energy                             (kcal/mol) =       0.58
 Total non electrostatic                      (kcal/mol) =       5.77
 -------------------------------------------------------------------
--
 Partition over spheres:
 Sphere   on Atom  Surface  Charge   GEl     GCav    GDR
    1      O1      17.14   0.284  -19.46    2.34   -1.27
    2      C2       4.40  -0.001   -0.07    0.88   -0.28
    3      O3      17.30   0.281  -19.00    2.32   -1.32
    4      C4       0.08  -0.001    0.06    0.23    0.00
    5      F5      14.53   0.077   -3.53    2.13   -1.17
    6      F6      15.27   0.078   -3.59    2.18   -1.23
    7      F7      15.28   0.078   -3.55    2.18   -1.23
 Added spheres:    27.37   0.154   -9.93    0.00    0.00
 -------------------------------------------------------------------
--
 After PCM corrections, the SCF energy is  -526.393783856     a.u.
 -------------------------------------------------------------------
--
```

B3LYP/6-31+G** SCRF=PCM(CH_3CN)	**12**	

```
Variational PCM results
 =======================
 <psi(f)|   H    |psi(f)>                         (a.u.) =    -
576.007956
 <psi(f)|H+V(f)/2|psi(f)>                         (a.u.) =    -
576.094131
 Total free energy in solution:
  with all non electrostatic terms                (a.u.) =    -
576.077899
 -------------------------------------------------------------------
--
 (Polarized solute)-Solvent                   (kcal/mol) =     -54.08
 -------------------------------------------------------------------
--
```

```
 Cavitation energy                               (kcal/mol) =      22.14
 Dispersion energy                               (kcal/mol) =     -12.78
 Repulsion energy                                (kcal/mol) =       0.83
 Total non electrostatic                         (kcal/mol) =      10.19
 -----------------------------------------------------------------------
--
 Partition over spheres:
 Sphere   on Atom  Surface  Charge   GEl     GCav    GDR
    1      C3      17.31  -0.044   -1.88    2.00   -1.29
    2      C4      17.05  -0.046   -2.06    2.00   -1.30
    3      C6      17.90  -0.049   -2.20    2.00   -1.32
    4      C7      11.99  -0.058   -3.23    1.84   -0.97
    5      C9       9.22  -0.043   -2.32    1.56   -0.62
    6      C10      0.00   0.000    0.00    0.08    0.00
    7      C14      4.68  -0.087   -5.93    0.68   -0.30
    8      C16     45.95  -0.261  -12.28    5.00   -2.33
    9      C18      0.00   0.000    0.00    0.08    0.00
   10      C19     27.46  -0.250  -13.41    2.91   -1.78
   11      O22      5.55   0.003    0.02    1.11   -0.50
   12      C23      1.40  -0.021   -1.10    0.54   -0.11
   13      O24     19.96   0.068    1.74    2.35   -1.43
 Added spheres:    43.66  -0.205  -11.43    0.00    0.00
 -----------------------------------------------------------------------
--
 After PCM corrections, the SCF energy is  -576.094130560     a.u.
 -----------------------------------------------------------------------
--
```

B3LYP/6-31+G**<br>SCRF=PCM(CH_3CN)	**14**	

```
Variational PCM results
 =======================
 <psi(f)|   H    |psi(f)>                            (a.u.) =    -
575.994764
 <psi(f)|H+V(f)/2|psi(f)>                            (a.u.) =    -
576.091414
 Total free energy in solution:
  with all non electrostatic terms                   (a.u.) =    -
576.075999
 -----------------------------------------------------------------------
--
 (Polarized solute)-Solvent                      (kcal/mol) =     -60.65
 -----------------------------------------------------------------------
--
 Cavitation energy                               (kcal/mol) =      21.87
 Dispersion energy                               (kcal/mol) =     -13.02
 Repulsion energy                                (kcal/mol) =       0.83
 Total non electrostatic                         (kcal/mol) =       9.67
 -----------------------------------------------------------------------
--
 Partition over spheres:
 Sphere   on Atom  Surface  Charge   GEl     GCav    GDR
    1      O1      18.81   0.174    1.76    2.31   -1.40
    2      C2       2.31  -0.019   -0.68    0.54   -0.16
    3      O3       6.20   0.044    0.91    0.98   -0.55
    4      C5      21.15  -0.212  -10.92    2.78   -1.60
```

```
    5      C7       5.30  -0.075   -4.36    0.85   -0.54
    6      C10     42.59  -0.146   -5.95    4.67   -2.26
    7      C12      0.28  -0.004   -0.25    0.14   -0.02
    8      C15      0.49  -0.003   -0.16    0.27   -0.04
    9      C16     10.74  -0.065   -3.67    1.74   -0.95
   10      C18      8.43  -0.052   -2.96    1.60   -0.74
   11      C19     17.38  -0.098   -5.14    2.00   -1.32
   12      C21     17.37  -0.098   -5.20    2.00   -1.34
   13      C22     17.23  -0.121   -6.27    1.99   -1.28
 Added spheres:    52.20  -0.317  -17.75    0.00    0.00
 -----------------------------------------------------------------
--
 After PCM corrections, the SCF energy is  -576.091414269     a.u.
 -----------------------------------------------------------------
--
```

B3LYP/6-31+G**<br>SCRF=PCM(CH_3CN)	**2f**	

```
Variational PCM results
 =======================
 <psi(f)|   H    |psi(f)>                       (a.u.) =    -
575.678648
 <psi(f)|H+V(f)/2|psi(f)>                       (a.u.) =    -
575.702411
 Total free energy in solution:
  with all non electrostatic terms              (a.u.) =    -
575.686962
 -----------------------------------------------------------------
--
 (Polarized solute)-Solvent                 (kcal/mol) =     -14.91
 -----------------------------------------------------------------
--
 Cavitation energy                          (kcal/mol) =      21.77
 Dispersion energy                          (kcal/mol) =     -12.95
 Repulsion energy                           (kcal/mol) =       0.88
 Total non electrostatic                    (kcal/mol) =       9.69
 -----------------------------------------------------------------
--
 Partition over spheres:
 Sphere   on Atom   Surface  Charge   GEl     GCav    GDR
    1      C1       5.43  -0.053   -0.89    0.85   -0.54
    2      C2       0.29  -0.001   -0.01    0.20   -0.02
    3      C3      16.08  -0.055   -0.73    2.23   -1.32
    4      C4       3.14  -0.012    0.07    0.66   -0.22
    5      O5       6.27   0.064   -1.11    0.99   -0.53
    6      C6       0.44   0.001    0.00    0.21   -0.02
    7      O7      20.91   0.257   -6.47    2.40   -1.46
    8      C8       8.63  -0.017   -0.49    1.62   -0.77
    9      C9      17.32  -0.034   -0.88    2.00   -1.33
   10      C10     17.40  -0.035   -0.87    2.00   -1.29
   11      C11     17.30  -0.033   -0.83    1.99   -1.32
   12      C12     12.32  -0.028   -0.69    1.89   -1.00
   13      C13     42.44  -0.063   -0.38    4.74   -2.26
 Added spheres:    49.99  -0.017   -1.63    0.00    0.00
 -----------------------------------------------------------------
--
```

After PCM corrections, the SCF energy is -575.702411411   a.u.

--

B3LYP/6-31+G** SCRF=PCM(CH_3CN)	**4f**	

Variational PCM results
=======================
<psi(f)| H |psi(f)>   (a.u.) =   -575.668755
<psi(f)|H+V(f)/2|psi(f)>   (a.u.) =   -575.690484
Total free energy in solution:
with all non electrostatic terms   (a.u.) =   -575.674963

--
(Polarized solute)-Solvent   (kcal/mol) =   -13.63

--

Cavitation energy	(kcal/mol) =	21.61
Dispersion energy	(kcal/mol) =	-12.73
Repulsion energy	(kcal/mol) =	0.87
Total non electrostatic	(kcal/mol) =	9.74

--
Partition over spheres:

Sphere	on Atom	Surface	Charge	GEl	GCav	GDR
1	C1	0.00	0.000	0.00	0.00	0.00
2	C2	11.35	-0.077	-1.73	1.54	-0.82
3	C3	20.56	-0.078	-1.02	2.33	-1.47
4	C4	2.22	-0.010	0.04	0.66	-0.10
5	O5	4.23	0.043	-0.72	0.74	-0.25
6	O6	20.91	0.246	-5.62	2.40	-1.48
7	C7	0.00	0.000	0.00	0.09	0.00
8	C8	42.65	-0.070	-0.53	4.45	-2.17
9	C9	9.33	-0.024	-0.73	1.55	-0.70
10	C10	17.33	-0.025	-0.76	2.00	-1.34
11	C11	17.42	-0.023	-0.69	2.00	-1.30
12	C12	17.28	-0.023	-0.62	1.99	-1.33
13	C13	11.44	-0.017	-0.24	1.85	-0.90
Added spheres:		42.12	0.030	-1.01	0.00	0.00

--
After PCM corrections, the SCF energy is -575.690483702   a.u.

--

B3LYP/6-31+G** SCRF=PCM(CH_3CN)	**TS 19**	

Variational PCM results
=======================
<psi(f)| H |psi(f)>   (a.u.) =   -575.946953
<psi(f)|H+V(f)/2|psi(f)>   (a.u.) =   -576.043233

```
 Total free energy in solution:
  with all non electrostatic terms           (a.u.) =    -
576.028425
 ------------------------------------------------------------------
--
 (Polarized solute)-Solvent              (kcal/mol) =     -60.42
 ------------------------------------------------------------------
--
 Cavitation energy                       (kcal/mol) =      21.03
 Dispersion energy                       (kcal/mol) =     -12.54
 Repulsion energy                        (kcal/mol) =       0.80
 Total non electrostatic                 (kcal/mol) =       9.29
 ------------------------------------------------------------------
--
 Partition over spheres:
 Sphere  on Atom  Surface  Charge   GEl     GCav    GDR
    1      C3      18.19  -0.113   -5.96    1.97   -1.25
    2      C4      17.60  -0.123   -6.98    1.92   -1.26
    3      C6      16.83  -0.125   -6.45    1.95   -1.27
    4      C7       8.94  -0.100   -6.45    1.17   -0.68
    5      C9      11.71  -0.092   -4.55    1.67   -0.93
    6      C10      0.99  -0.007   -0.33    0.20   -0.03
    7      C14      5.27  -0.098   -6.69    0.85   -0.47
    8      C16     47.01  -0.155   -6.43    4.90   -2.45
    9      C18      0.00   0.000    0.00    0.00    0.00
   10      C19     22.50  -0.174   -8.42    2.77   -1.56
   11      O22      4.95   0.033    0.74    0.88   -0.37
   12      C23      1.66  -0.015   -0.55    0.44   -0.10
   13      O24     18.75   0.166    2.09    2.31   -1.39
 Added spheres:    37.13  -0.187  -10.43    0.00    0.00
 ------------------------------------------------------------------
--
 After PCM corrections, the SCF energy is  -576.043233174     a.u.
 ------------------------------------------------------------------
--
```

B3LYP/6-31+G** SCRF=PCM(CH_3CN)	**TS 13**	

```
  Variational PCM results
 =======================
 <psi(f)|   H    |psi(f)>                    (a.u.) =    -
575.966852
 <psi(f)|H+V(f)/2|psi(f)>                    (a.u.) =    -
576.058756
 Total free energy in solution:
  with all non electrostatic terms           (a.u.) =    -
576.042792
 ------------------------------------------------------------------
--
 (Polarized solute)-Solvent              (kcal/mol) =     -57.67
 ------------------------------------------------------------------
--
 Cavitation energy                       (kcal/mol) =      22.18
 Dispersion energy                       (kcal/mol) =     -12.95
 Repulsion energy                        (kcal/mol) =       0.79
 Total non electrostatic                 (kcal/mol) =      10.02
```

```
---------------------------------------------------------------------
--
 Partition over spheres:
 Sphere  on Atom  Surface  Charge   GEl     GCav    GDR
    1      C5      11.31  -0.030   -1.55    1.51   -0.62
    2      C6      20.37  -0.066   -3.12    2.00   -1.35
    3      C7      47.68  -0.250  -11.61    4.84   -2.39
    4      O10      7.52   0.016    0.34    1.07   -0.51
    5      C11      0.13  -0.004   -0.28    0.08    0.00
    6      O12     21.91   0.140    2.55    2.33   -1.40
    7      C13      5.28  -0.057   -2.69    0.55   -0.21
    8      C14      0.44  -0.008   -0.49    0.12    0.00
    9      C15      1.63  -0.009   -0.54    0.24    0.00
   10      C16     20.37  -0.071   -3.14    2.00   -1.29
   11      C18     28.18  -0.288  -15.84    2.96   -1.77
   12      C19     14.76  -0.057   -3.25    1.77   -0.95
   13      C20     20.39  -0.064   -3.05    2.00   -1.33
   14      H21      5.80  -0.100   -6.60    0.73   -0.35
 Added spheres:    19.42  -0.144   -8.40    0.00    0.00
---------------------------------------------------------------------
--
 After PCM corrections, the SCF energy is  -576.058755590     a.u.
---------------------------------------------------------------------
--
```


[1] M. Justik, G. Koser, *Molecules* **2005**, *10*, 217–225.

[2] A. A. Zagulyaeva, M. S. Yusubov, V. V. Zhdankin, *J. Org. Chem.* **2010**, *75*, 2119–2122.

[3] R. D. Richardson, J. M. Zayed, S. Altermann, D. Smith, T. Wirth, *Angew. Chem.* **2007**, *119*, 6649–6652; Angew. *Chem. Int. Ed.* **2007**, *46*, 6529 –6532.

[4] J.-M. Chen, X.-M. Zeng, K. Middleton, M. S. Yusubov, V. V. Zhdankin, *Synlett* **2011**, 1613–1617.

[5] J. Gallos, A. Varvoglis, *J. Chem. Soc., Perkin Trans. 1* **1985**, 757–763.

[6] a) M. Fujita, S. Okuno, H. J. Lee, T. Sugimura, T. Okuyama, *Tetrahedron Lett.* **2007**, *48*, 8691–8694; b) M. Fujita, Y. Yoshida, K. Miyata, A. Wakisaka, T. Sugimura, *Angew. Chem.* **2010**, *122*, 7222–7225; *Angew. Chem. Int. Ed.* **2010**, *49*, 7068–7071; c) M. Fujita, M. Wakita, T. Sugimura, *Chem. Commun.* **2011**, *47*, 3983–3985.

[7] S. E. Denmark, M. G. Edwards, *J. Org. Chem.* **2006**, *71*, 7293–7306.

[8] D. P. Hudgens, C. Taylor, T. W. Batts, M. K. Patel, M. L. Brown, *Bio. Med. Chem.* **2006**, *14*, 8366–8378.

[9] F. Gualtieri, E. Teodori, C. Bellucci, E. Pesce, G. Piacenza, *J. Med. Chem.* **1985**, *28*, 1621–1628.

[10] K. Takami, H. Yorimitsu, K. Oshima, *Org. Lett.* **2004**, *6*, 4555–4558.

[11] E. Grovenstein, K. W. Black, S. C. Goel, R. L. Hughes, J. H. Northrop, D. L. Streeter, D. VanDerveer, *J. Org. Chem.* **1989**, *54*, 1671–1679.
[12] a) D. M. Browne, O. Niyomura, T. Wirth, *Org. Lett.* **2007**, *9*, 3169–3171; b) J. Izquierdo, S. Rodriguez, F. V. Gonzalez, *Org. Lett.* **2011**, *13*, 3856–3859.
[13] D. M. Browne, O. Niyomura, T. Wirth, *Phosphorus Sulfur* **2008**, *183*, 1026–1035.
[14] J.-M. Garnier, S. Robin, G. Rousseau, *Eur. J. Org. Chem.* **2007**, 3281–3291.
[15] J.-M. Garnier, S. Robin, R. Guillot, G. Rousseau, *Tetrahedron: Asymm.* **2007**, *18*, 1434–1442.
[16] A. A. Scholte, John C. Vederas, *Org. Biomol. Chem.* **2006**, *4*, 730–742.
[17] A. R. Katritzky, D. Feng, H. Lang, *J. Org. Chem.* **1997**, *62*, 715–720.
[18] Y. Lei, Z.-Q. Wang, Y.-X. Xie, S.-C. Yu, B.-X. Tang, J.-H. Li, *Adv. Synth. Cat.* **2011**, *353*, 31–35.
[19] L. Artok, M. Kuş, Ö. Aksın-Artok, F. N. Dege, F. Y. Özkılınç, *Tetrahedron* **2009**, *65*, 9125–9133.
[20] S. Lamandé-Langle, S. I. Ngi, E. Anselmi, H. Allouchi, A. Duchêne, M. Abarbri, J. Thibonnet, *Synthesis* **2011**, 154–160.
[21] Y. Kita, J. Sekihachi, Y. Hayashi, Y.-Z. Da, M. Yamamoto, S. Akai, *J.* Org. *Chem.* **1990**, *55*, 1108–1112.
[22] J. Delaunay, A. Orliac-Lemoing, J. Simonet, *Tetrahedron* **1988**, *44*, 7089–7094.
[23] L. Zhou, L. Lin, J. Ji, M. Xie, X. Liu, X. Feng, *Org. Lett.* **2011**, *13*, 3056–3059.
[24] K. Oishi, K.Kurosawa, *Bull. Chem. Soc. Japan* **1980**, *53*, 179–184.
[25] Y.-Z. Chen, L.-Z. Wu, M.-L. Peng, D. Zhang, L.-P. Zhang, C.-H. Tung, *Tetrahedron* **2006**, *62*, 10688–10693.
[26] S. Nicolai, S. Erard, D. F. González, J. Waser, *Org. Lett.* **2010**, *12*, 384–387; b) F. V. Singh, T. Wirth, *Org. Lett.* **2011**, *13*, 6504–6507.
[27] C. Singh, C. S. Pandey, G. Saxena, N. Srivastava, M. Sharma, *J. Org. Chem*. **2006**, *71*, 9057–9061.
[28] R. M. Bullock, B. J. Rappoli, *J. Am. Chem. Soc.* **1991**, *113*, 1659–1669.
[29] E. J. Cragoe, E. A. M. Pietruszkiewicz, C. M. Robb, *J.* Org. *Chem.* **1958**, *23*, 971–980.
[30] R. C. Larock, C. K. Reddy, *J.* Org. *Chem.* **2002**, *67*, 2027–2033.
[31] M. J. Sofia, J. A. Katzenellenbogen, *J. Med. Chem*. **1986**, *29*, 230–238.
[32] A. Kar, I. A. Sayyed, W. F. Lo, H. M. Kaiser, M. Beller, M. K. Tse, *Org. Lett*. **2007**, *9*, 3405–3408.
[33] F. Bellina, C. Anselmi, F. Martina, R. Rossi, *Eur. J. Org. Chem*. **2003**, 2290–2302.
[34] N. R. Sharma, G. S. Rawat, *Asian J. Chem.* **2004**, *16*, 978–982.
[35] Gaussian 03, Revision E.01, Gaussian, Inc., Wallingford CT, 2004, see main text for full citation.
[36] a) W. Kohn, A. D. Becke, R. G. Parr, *J. Phys. Chem.* **1996**, *100*, 12974–12980; b) A. D. Becke *J. Chem. Phys.* **1993**, *98*, 5648–5652; c) C. Lee, W. Yang, R. G. Parr *Phys. Rev. B* **1988**, *37*, 785–789; d) P. J. Stephens, F. J. Devlin, C. F. Chabalowski, M. J. Frisch *J. Phys. Chem.* **1994**, *98*, 11623–11627; e) S. H. Vosko, L. Wilk, M. Nusair *Can. J. Phys.* **1980**, *58*, 1200–1211.
[37] a) W. J. Hehre, R. F. Stewart, J. A. Pople, *J. Chem. Phys.* **1969**, *51*, 2657–2667; b) J. S. Binkley, J. A. Pople, *J. Am. Chem. Soc.* **1980**, *102*, 939–947; c) W. J. Hehre, R. Ditchfield, J. A. Pople, *J. Chem. Phys.* **1972**, *56*, 2257–2261; d) R. Krishnan, J.

S. Binkley, R. Seeger, J. A. Pople, *J. Chem. Phys.* **1980**, *72*, 650–654; e) M. J. Frisch, J. A. Pople, J. S. Binkley, *J. Chem. Phys.* **1984**, *80*, 3265–3269; f) M. M. Francl, W. J. Pietro, W. J. Hehre, J. S. Binkley, M. S. Gordon, D. J. DeFrees, J. A. Pople, *J. Chem. Phys.* **1982**, *77*, 3654–3665.

[38] a) P. J. Hay, W. R. Wadt, *J. Chem. Phys.* **1985**, *82*, 270–283; b) W. R. Wadt, P. J. Hay, *J. Chem. Phys.* **1985**, *82*, 284–298; c) P. J. Hay, W. R. Wadt, *J. Chem. Phys.* **1985**, *82*, 299–310.

[39] a) E. Cancès, B. Mennucci, J. Tomasi, *J. Chem. Phys.* **1997**, *107*, 3032–3041; b) J. Tomasi, B. Mennucci, R. Cammi, *Chem. Rev.* **2005**, *105*, 2999–3093; c) A. Klamt, B. Mennucci, J. Tomasi, V. Barone, C. Curutchet, M. Orozco, F. J. Luque, *Acc. Chem. Res.* **2009**, *42*, 489–492.

[40] J. W. McIver, A. Komornicki, *J. Am. Chem. Soc.* **1972**, *94*, 2625–2633.

[41] a) C. Gonzales, H. B. Schlegel, *J. Phys. Chem.* **1989**, *90*, 2154–2161; b) C. Gonzales, H. B. Schlegel, *J. Chem. Phys.* **1990**, *94*, 5523–5527.

[42] Free visualization software Gabedit 2.2.9 by A.-R. Allouche: http://gabedit.sourceforge.net/. See also: A.-R. Allouche, *J. Comp. Chem.* **2011**, *32*, 174–182.

NMR spectra (solvents and frequencies are found in the data above)

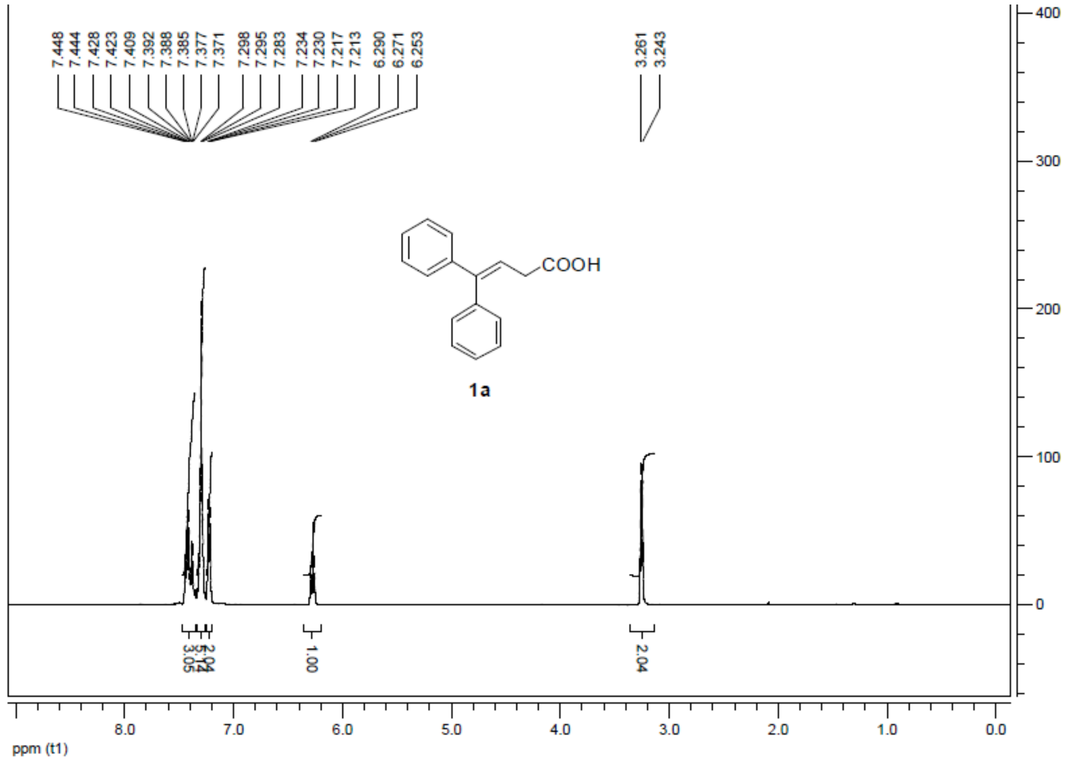

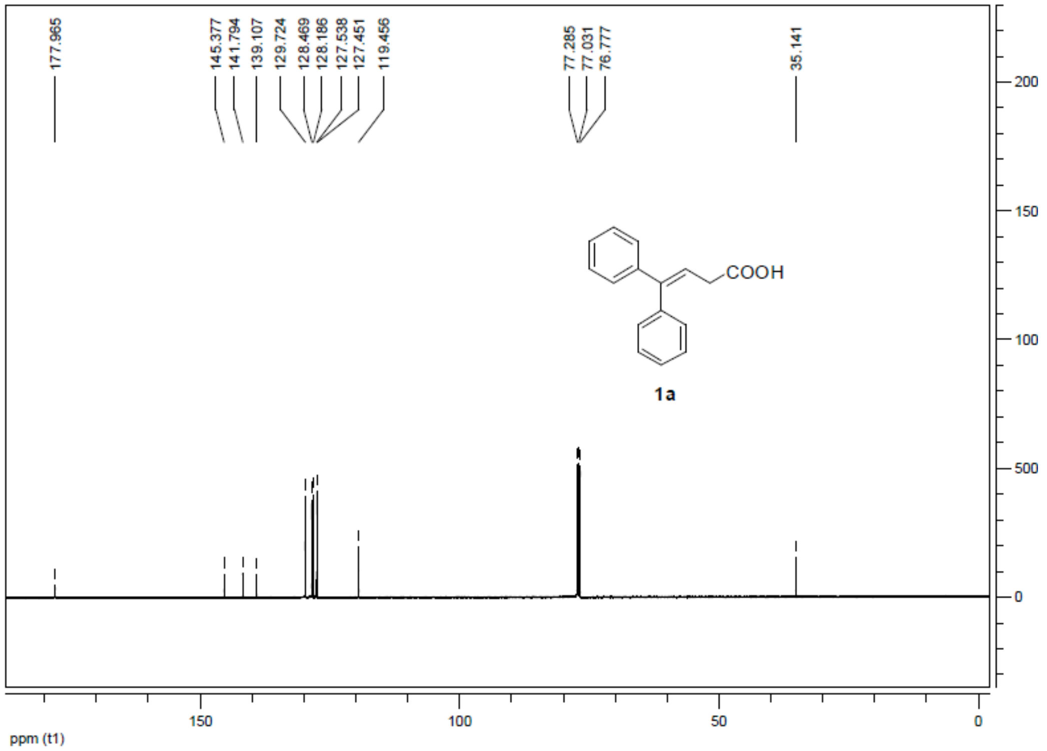

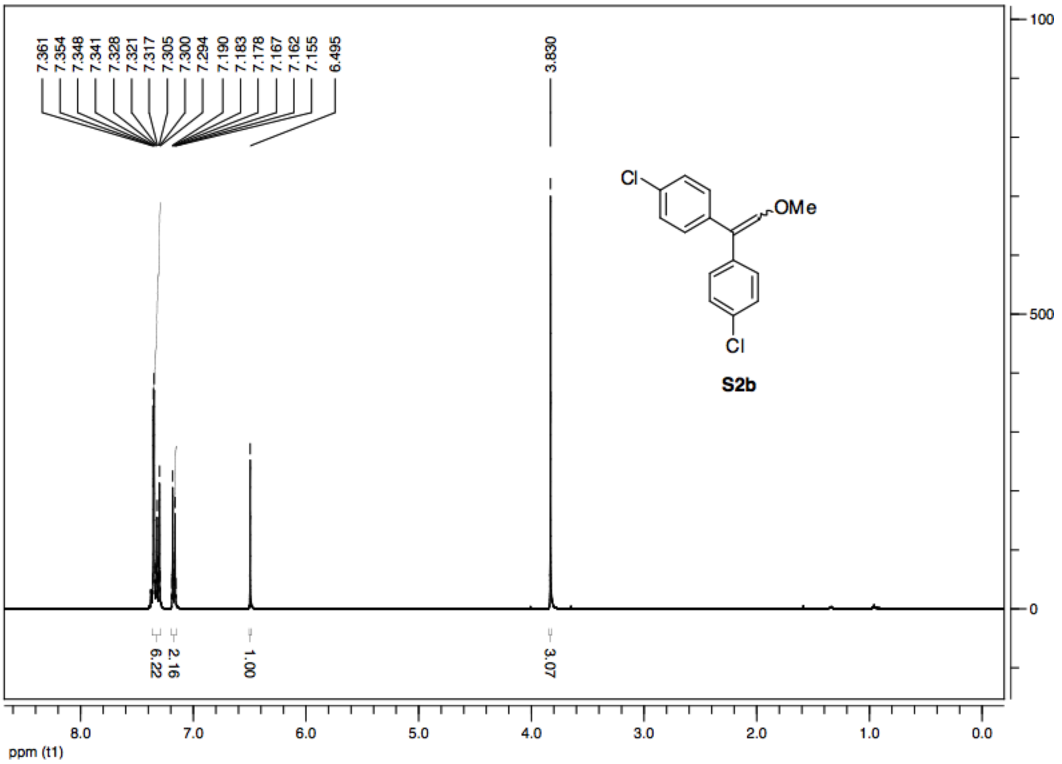
7.361
7.354
7.348
7.341
7.328
7.321
7.317
7.305
7.300
7.294
7.190
7.183
7.178
7.167
7.162
7.155
6.495
3.830
Cl
OMe
Cl
S2b
100
500
0
6.22
2.16
1.00
3.07
8.0
7.0
6.0
5.0
4.0
3.0
2.0
1.0
0.0
ppm (t1)

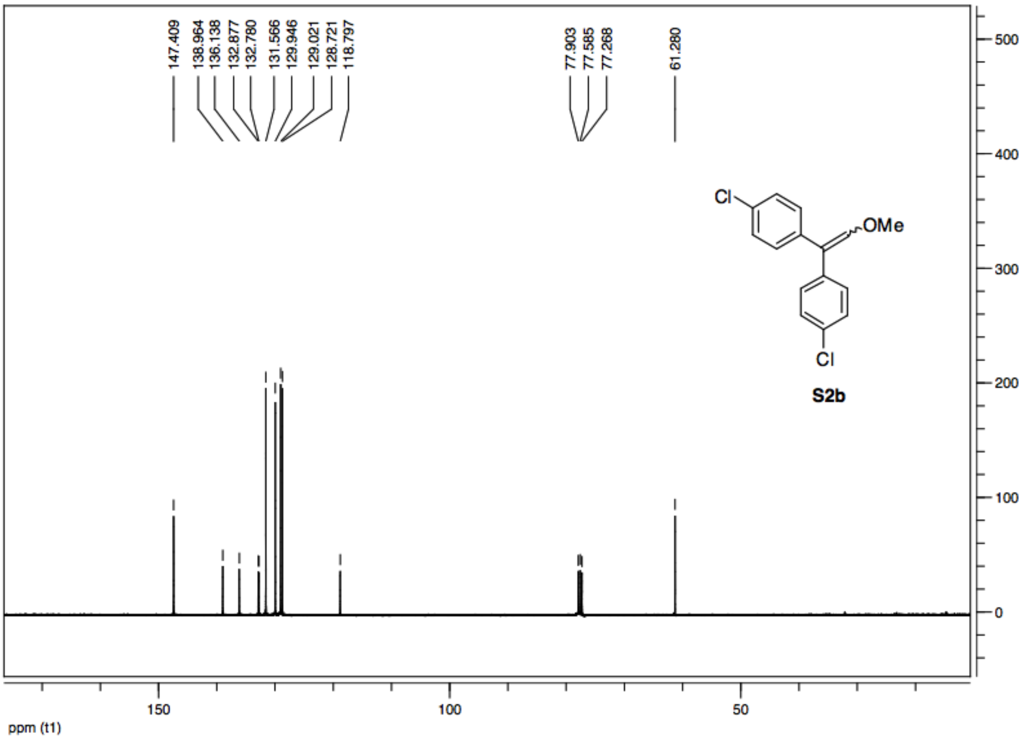
147.409
138.964
136.138
132.877
132.780
131.566
129.946
129.021
128.721
118.797
77.903
77.585
77.268
61.280
Cl
OMe
Cl
S2b
500
400
300
200
100
0
150
100
50
ppm (t1)

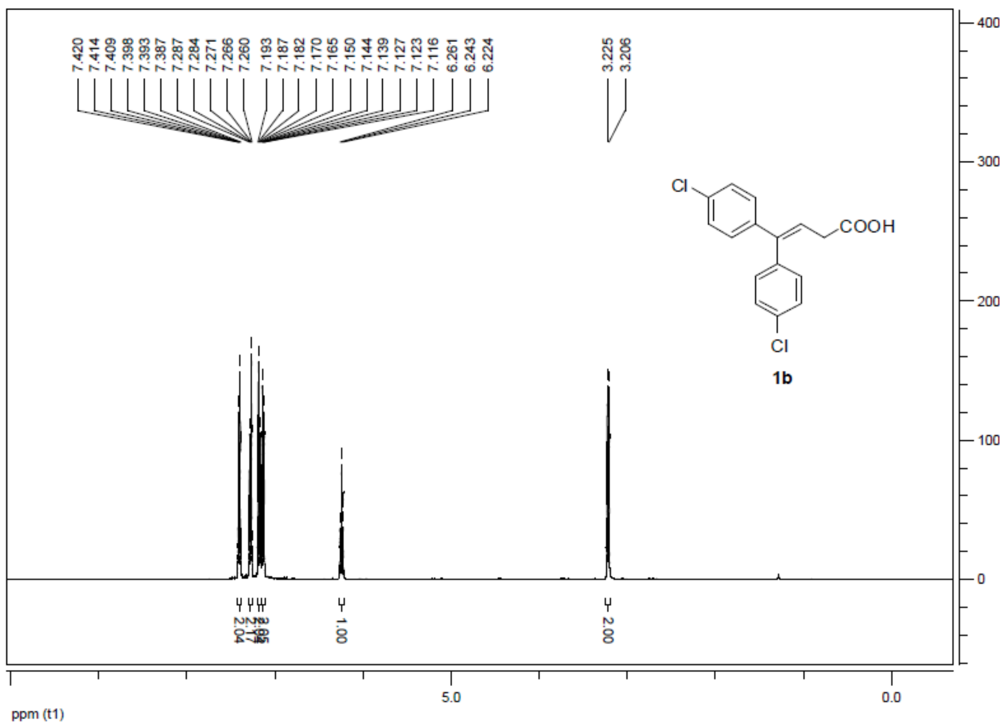
7.420
7.414
7.409
7.398
7.393
7.387
7.287
7.284
7.271
7.266
7.260
7.193
7.187
7.182
7.170
7.165
7.150
7.144
7.139
7.127
7.123
7.116
6.261
6.243
6.224
3.225
3.206
Cl
COOH
Cl
1b
2.04
2.17
2.05
1.00
2.00
5.0
0.0
ppm (t1)
400
300
200
100
0

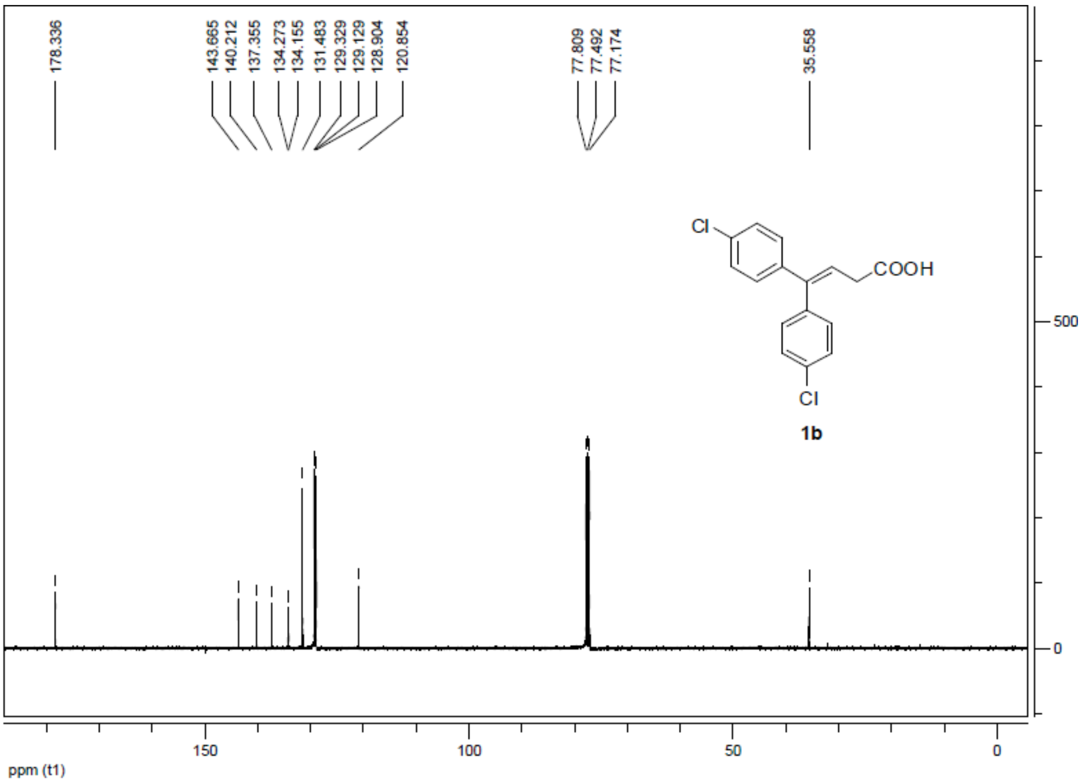
178.336
143.665
140.212
137.355
134.273
134.155
131.483
129.329
129.129
128.904
120.854
77.809
77.492
77.174
35.558
Cl
COOH
Cl
1b
150
100
50
0
ppm (t1)
500
0

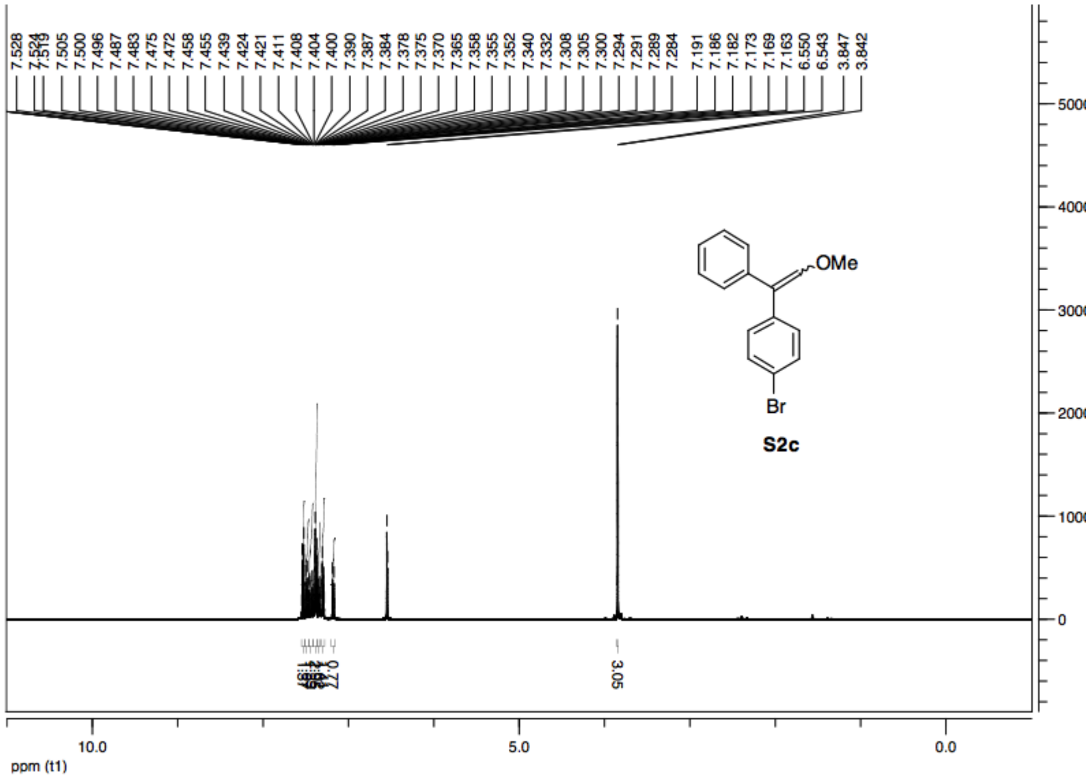
7.528
7.524
7.519
7.505
7.500
7.496
7.487
7.483
7.475
7.472
7.458
7.455
7.439
7.424
7.421
7.411
7.408
7.404
7.400
7.390
7.387
7.384
7.378
7.375
7.370
7.365
7.358
7.355
7.352
7.340
7.332
7.308
7.305
7.300
7.294
7.291
7.289
7.284
7.191
7.186
7.182
7.173
7.169
7.163
6.550
6.543
3.847
3.842
OMe
Br
S2c
0.77
3.05
10.0
5.0
0.0
ppm (t1)
5000
4000
3000
2000
1000
0

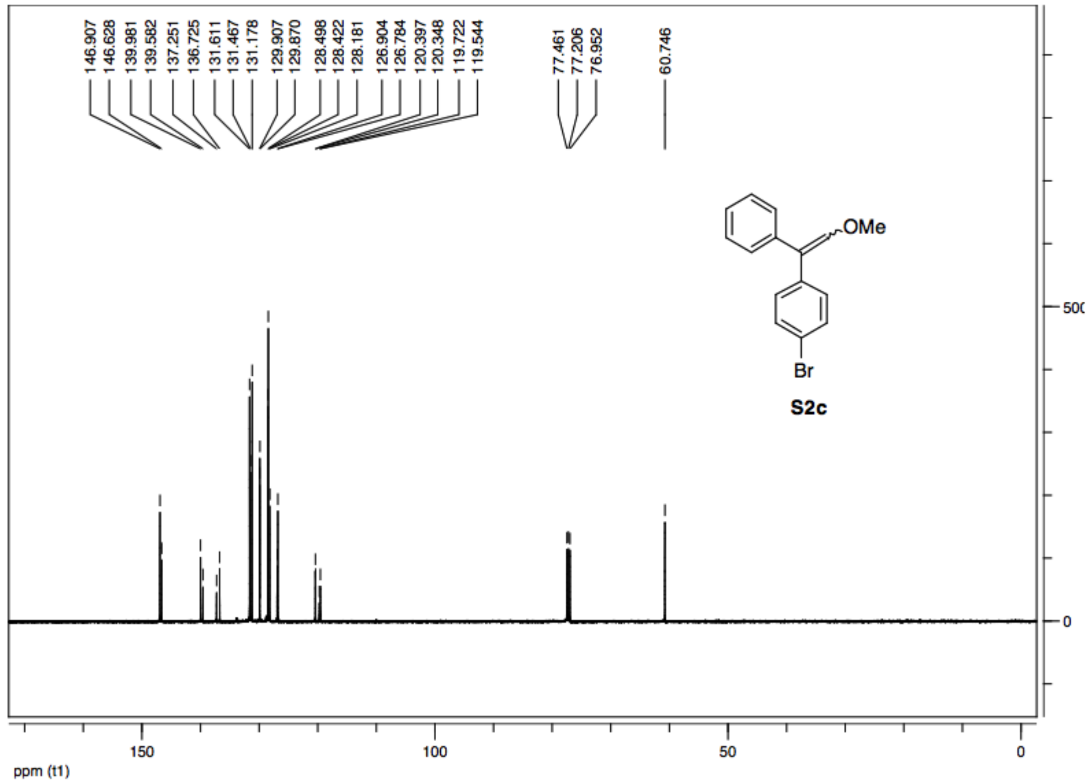
146.907
146.628
139.981
139.582
137.251
136.725
131.611
131.467
131.178
129.907
129.870
128.498
128.422
128.181
126.904
126.784
120.397
120.348
119.722
119.544
77.461
77.206
76.952
60.746
OMe
Br
S2c
150
100
50
0
ppm (t1)
500
0

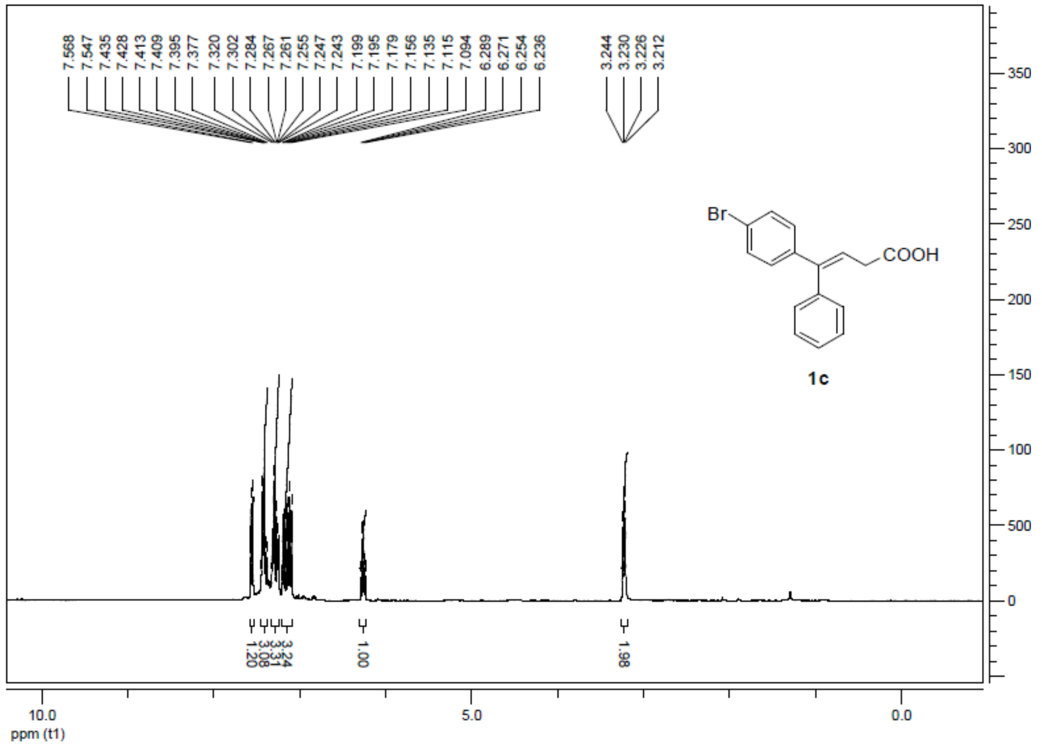
7.568
7.547
7.435
7.428
7.413
7.409
7.395
7.377
7.320
7.302
7.284
7.267
7.261
7.255
7.247
7.243
7.199
7.195
7.179
7.156
7.135
7.115
7.094
6.289
6.271
6.254
6.236
3.244
3.230
3.226
3.212
Br
COOH
1c
1.20
3.08
3.31
3.24
1.00
1.98
10.0
5.0
0.0
ppm (t1)

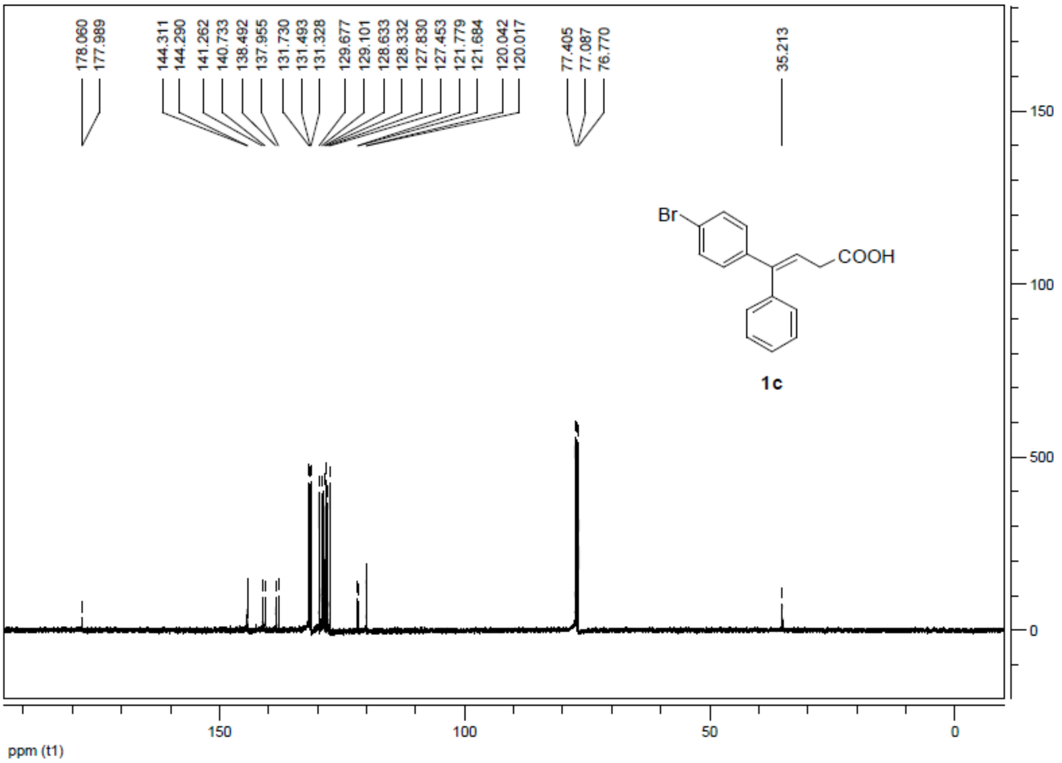
178.060
177.989
144.311
144.290
141.262
140.733
138.492
137.955
131.730
131.493
131.328
129.677
129.101
128.633
128.332
127.830
127.453
121.779
121.684
120.042
120.017
77.405
77.087
76.770
35.213
Br
COOH
1c
150
100
50
0
ppm (t1)

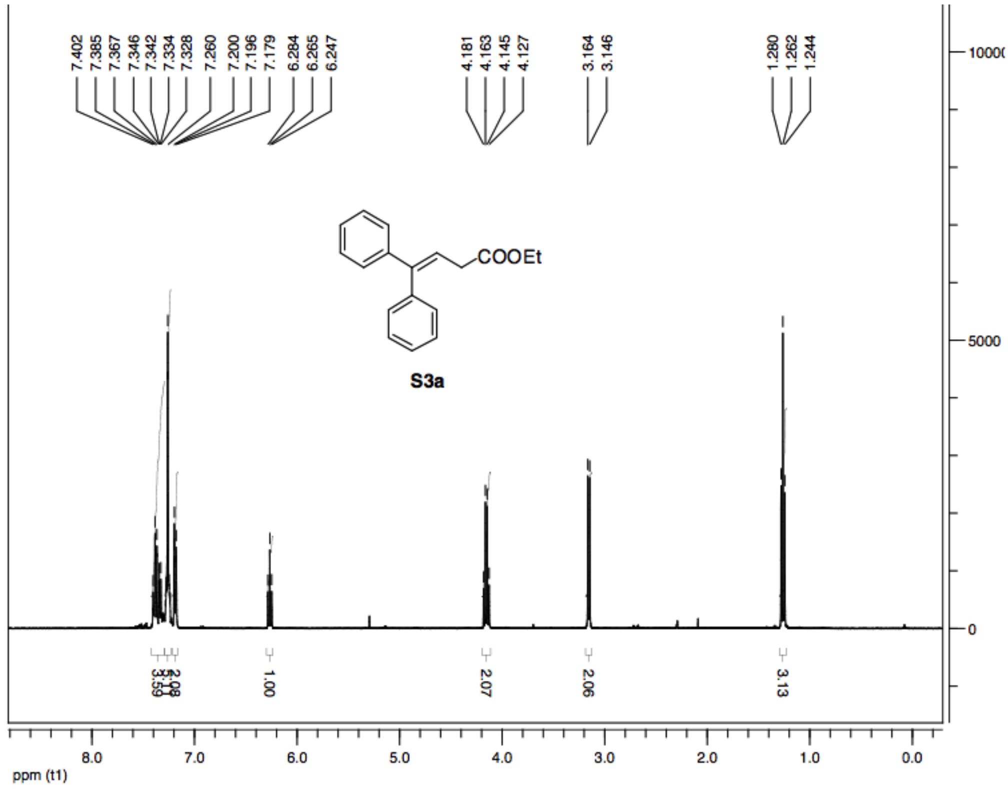
7.402
7.385
7.367
7.346
7.342
7.334
7.328
7.260
7.200
7.196
7.179
6.284
6.265
6.247
4.181
4.163
4.145
4.127
3.164
3.146
1.280
1.262
1.244
COOEt
S3a
3.59
5.13
2.08
1.00
2.07
2.06
3.13
ppm (t1)

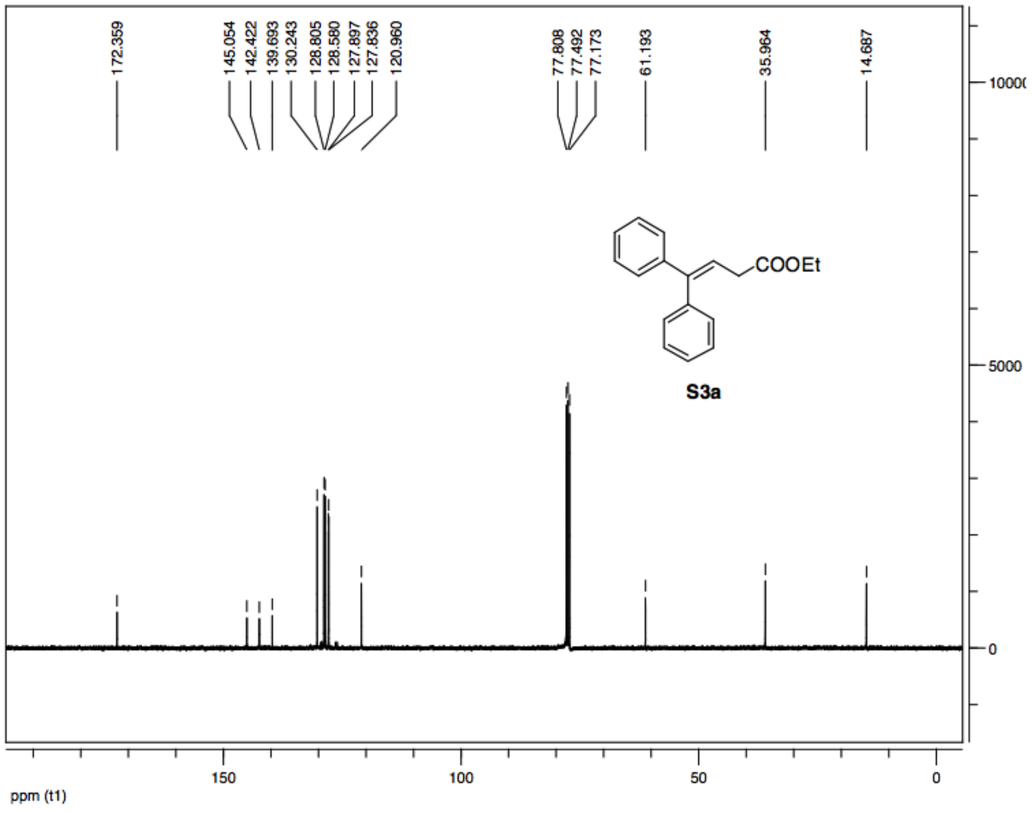
172.359
145.054
142.422
139.693
130.243
128.805
128.580
127.897
127.836
120.960
77.808
77.492
77.173
61.193
35.964
14.687
COOEt
S3a
ppm (t1)

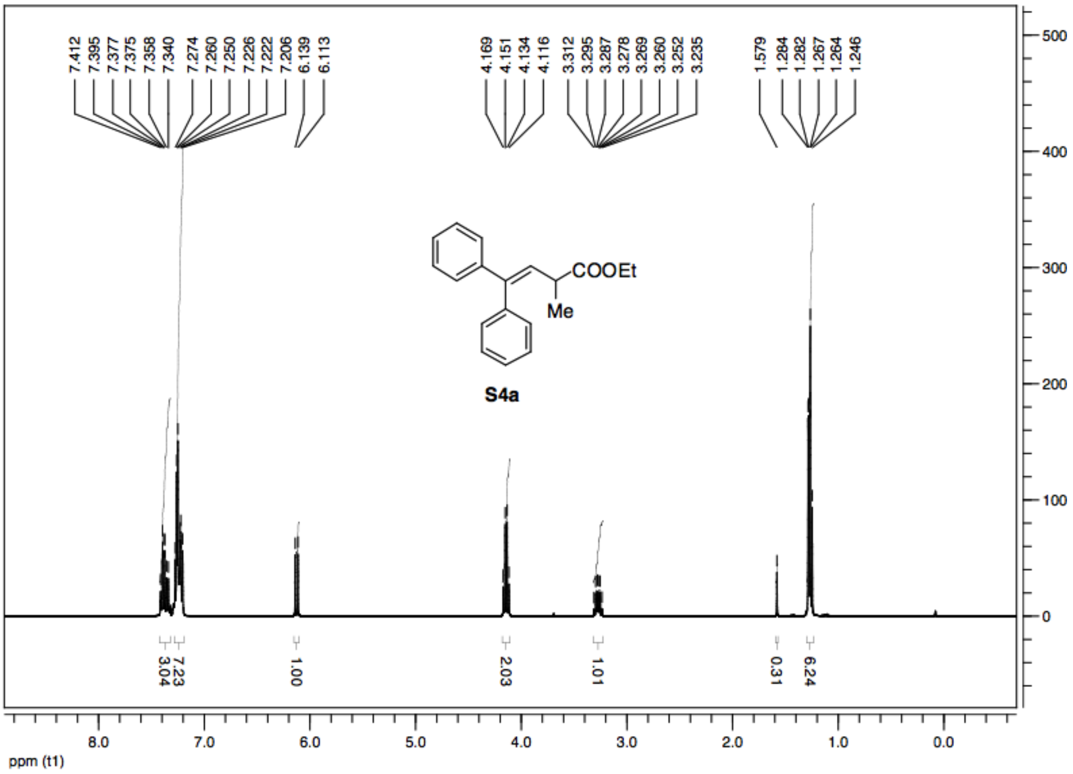
S4a
COOEt
Me
ppm (t1)

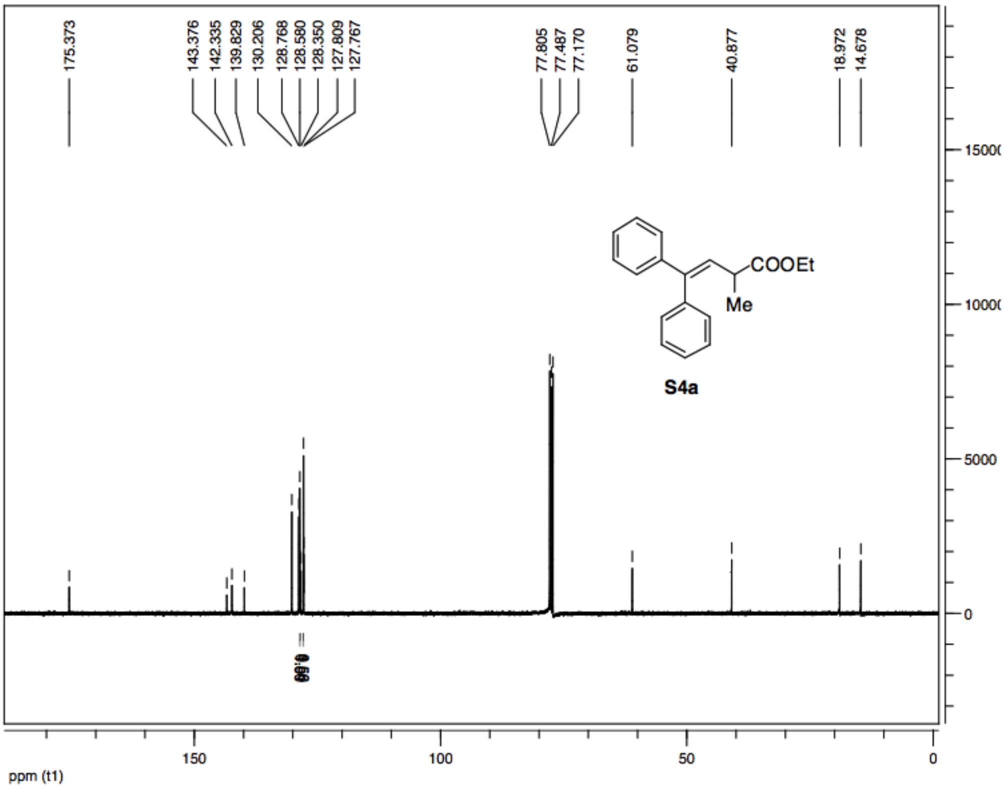
S4a
COOEt
Me
ppm (t1)

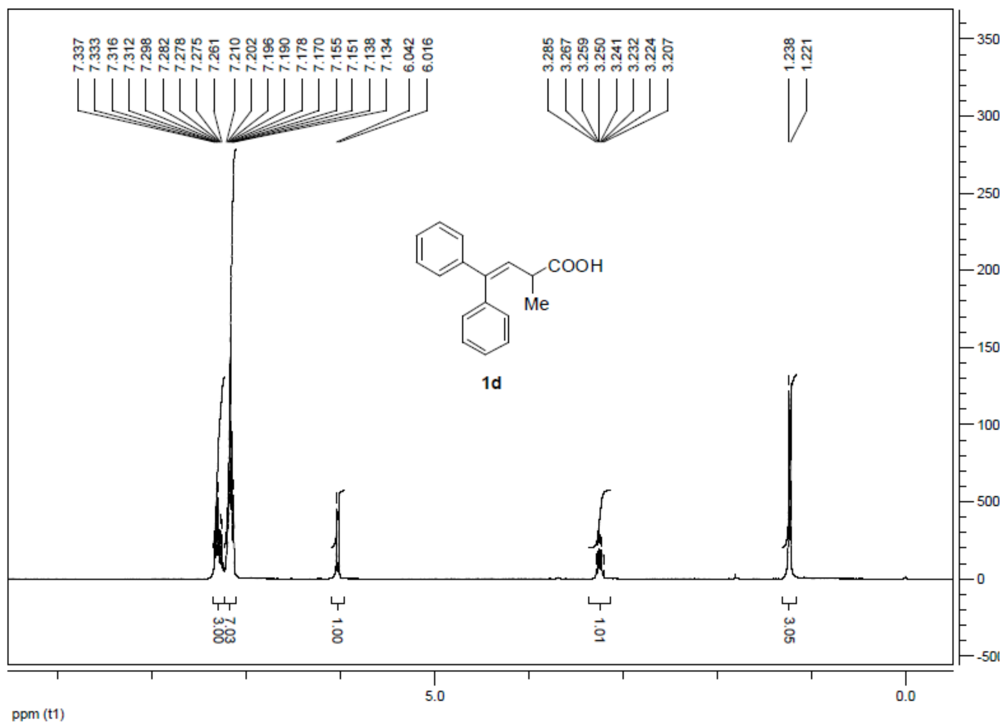
7.337
7.333
7.316
7.312
7.298
7.282
7.278
7.275
7.261
7.210
7.202
7.196
7.190
7.178
7.170
7.155
7.151
7.138
7.134
6.042
6.016
3.285
3.267
3.259
3.250
3.241
3.232
3.224
3.207
1.238
1.221
COOH
Me
1d
3.00
7.03
1.00
1.01
3.05
5.0
0.0
ppm (t1)

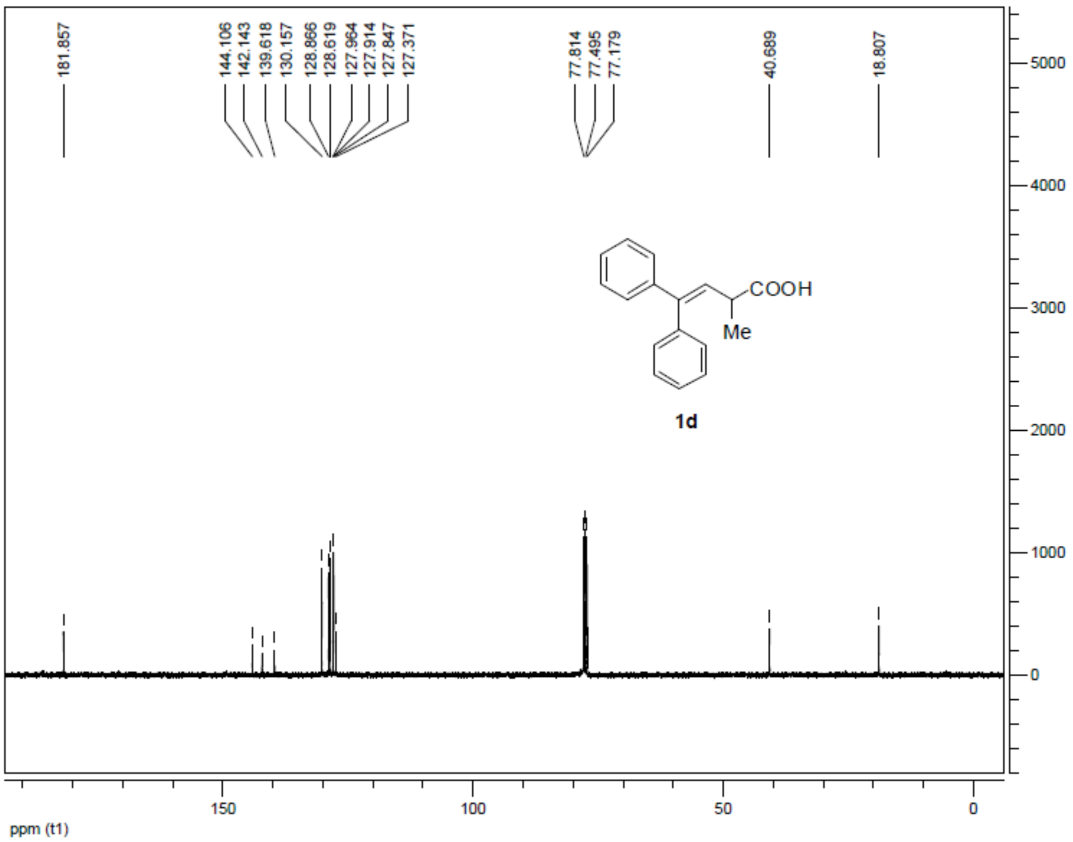
181.857
144.106
142.143
139.618
130.157
128.866
128.619
127.964
127.914
127.847
127.371
77.814
77.495
77.179
40.689
18.807
COOH
Me
1d
150
100
50
0
ppm (t1)

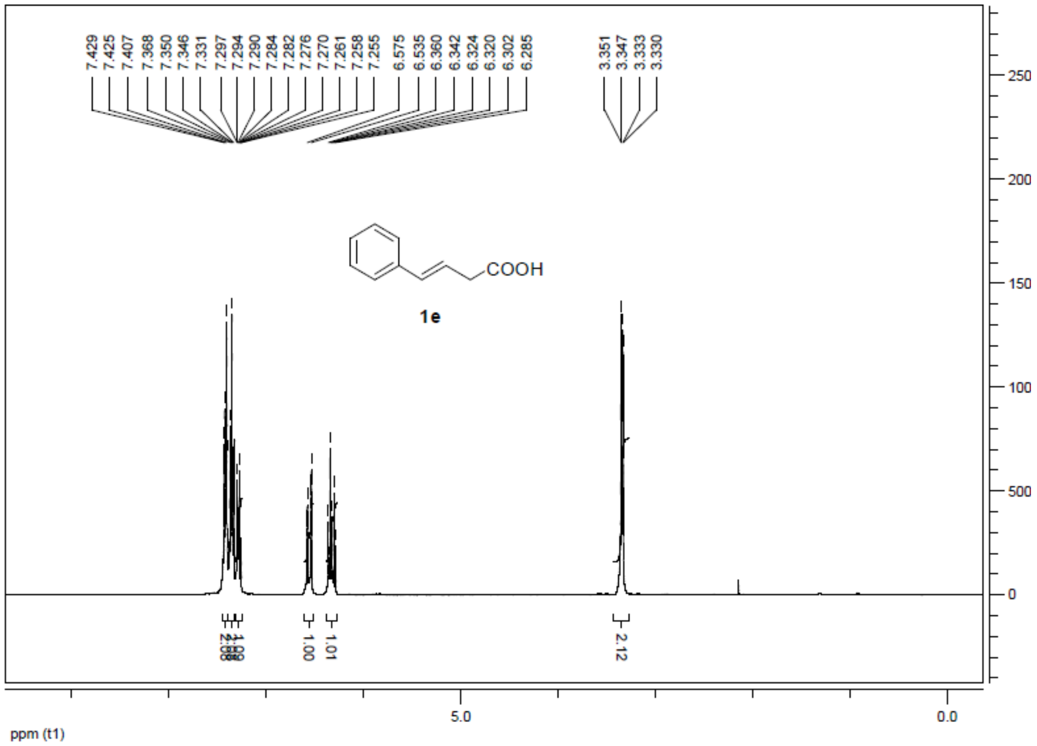

7.429
7.425
7.407
7.368
7.350
7.346
7.331
7.297
7.294
7.290
7.284
7.282
7.276
7.270
7.261
7.258
7.255
6.575
6.535
6.360
6.342
6.324
6.320
6.302
6.285
3.351
3.347
3.333
3.330
COOH
1e
1.00
1.01
2.12
5.0
0.0
ppm (t1)

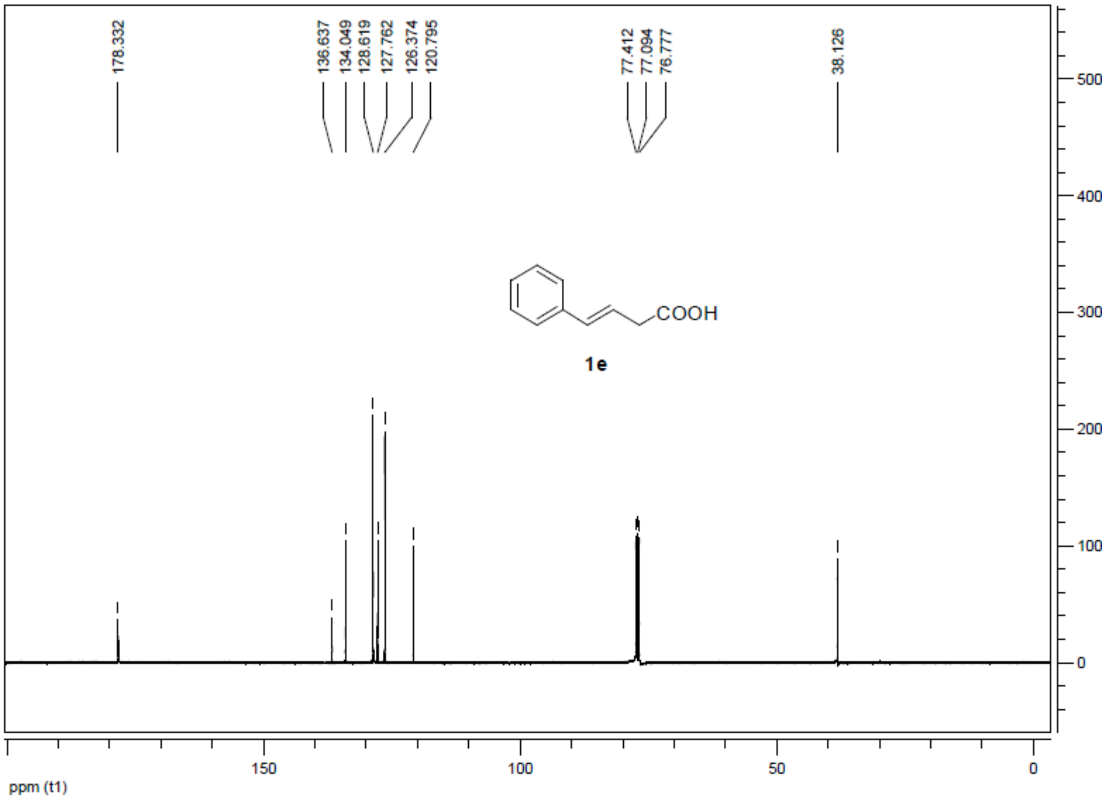

178.332
136.637
134.049
128.619
127.762
126.374
120.795
77.412
77.094
76.777
38.126
COOH
1e
150
100
50
0
ppm (t1)

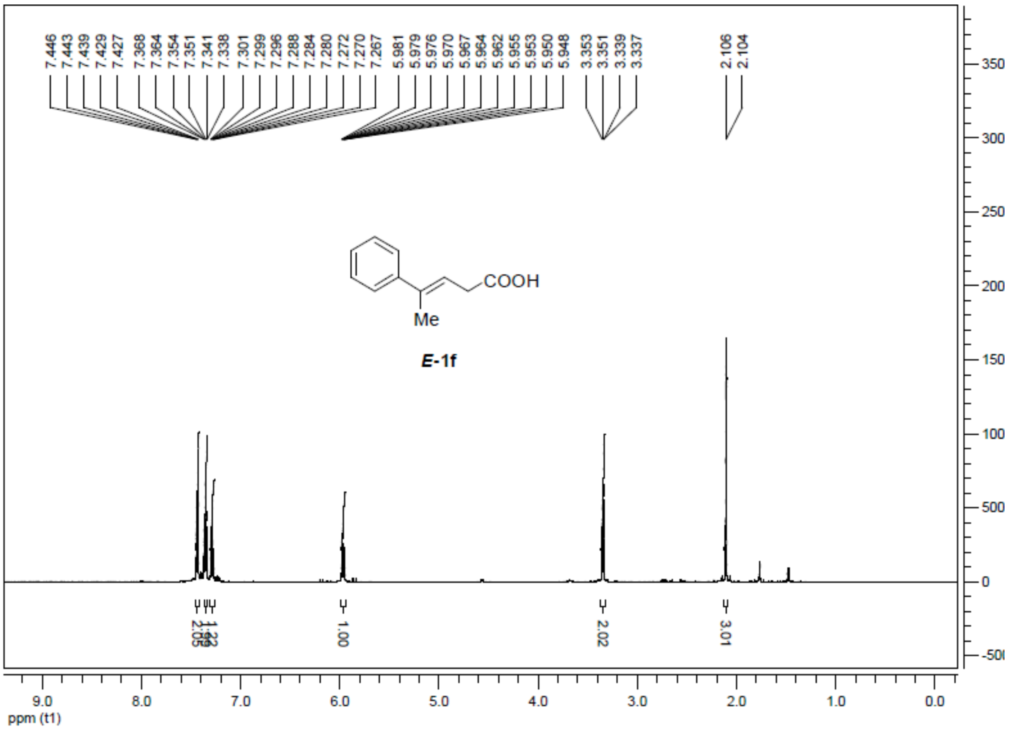
7.446
7.443
7.439
7.429
7.427
7.368
7.364
7.354
7.351
7.341
7.338
7.301
7.299
7.296
7.288
7.284
7.280
7.272
7.270
7.267
5.981
5.979
5.976
5.970
5.967
5.964
5.962
5.955
5.953
5.950
5.948
3.353
3.351
3.339
3.337
2.106
2.104
COOH
Me
E-1f
2.05
1.00
2.02
3.01
ppm (t1)

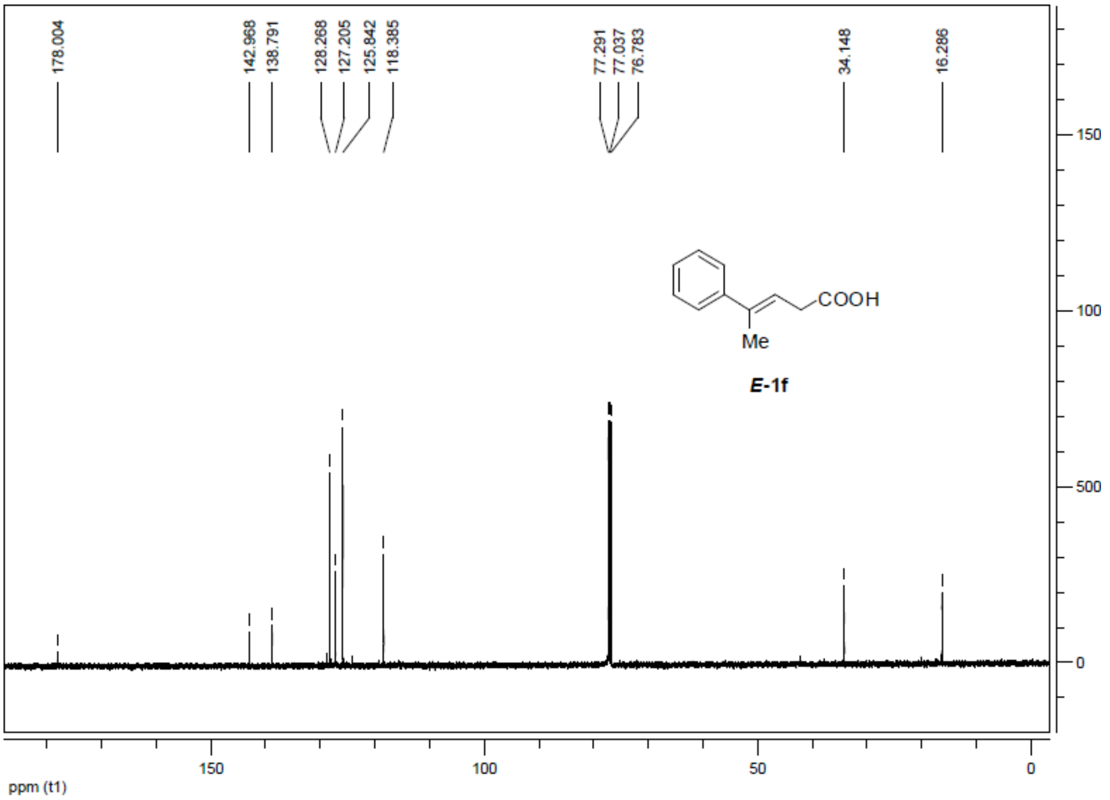
178.004
142.968
138.791
128.268
127.205
125.842
118.385
77.291
77.037
76.783
34.148
16.286
COOH
Me
E-1f
ppm (t1)

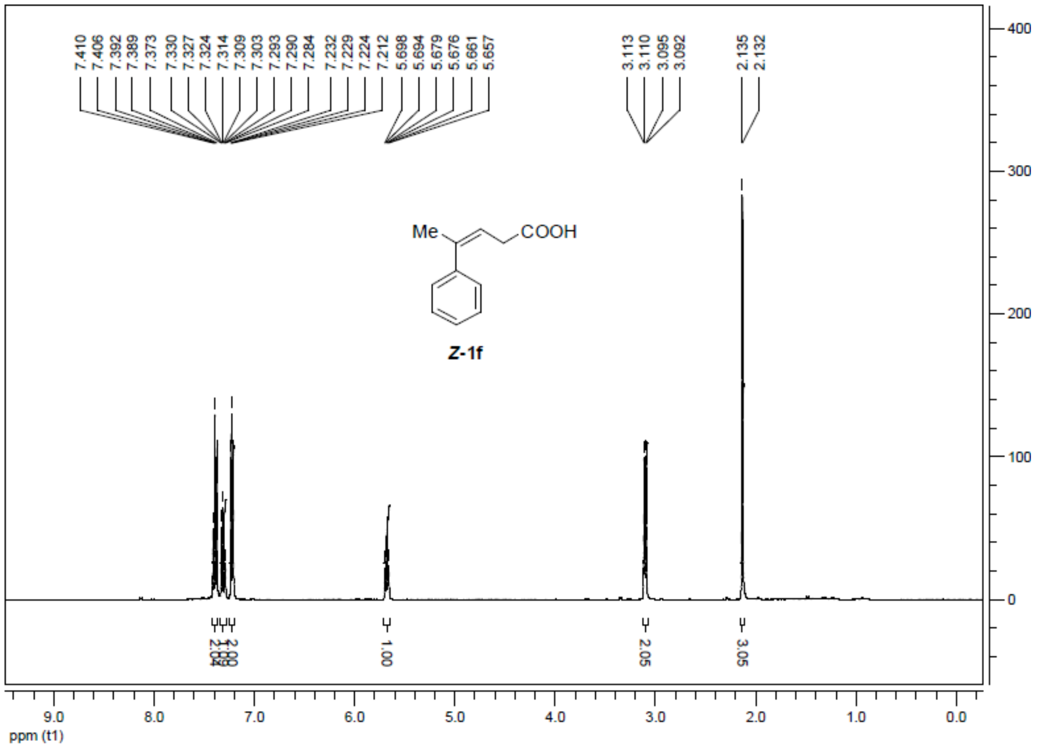
7.410
7.406
7.392
7.389
7.373
7.330
7.327
7.324
7.314
7.309
7.303
7.293
7.290
7.284
7.232
7.229
7.224
7.212
5.698
5.694
5.679
5.676
5.661
5.657
3.113
3.110
3.095
3.092
2.135
2.132
Me
COOH
Z-1f
1.00
2.05
3.05
ppm (t1)

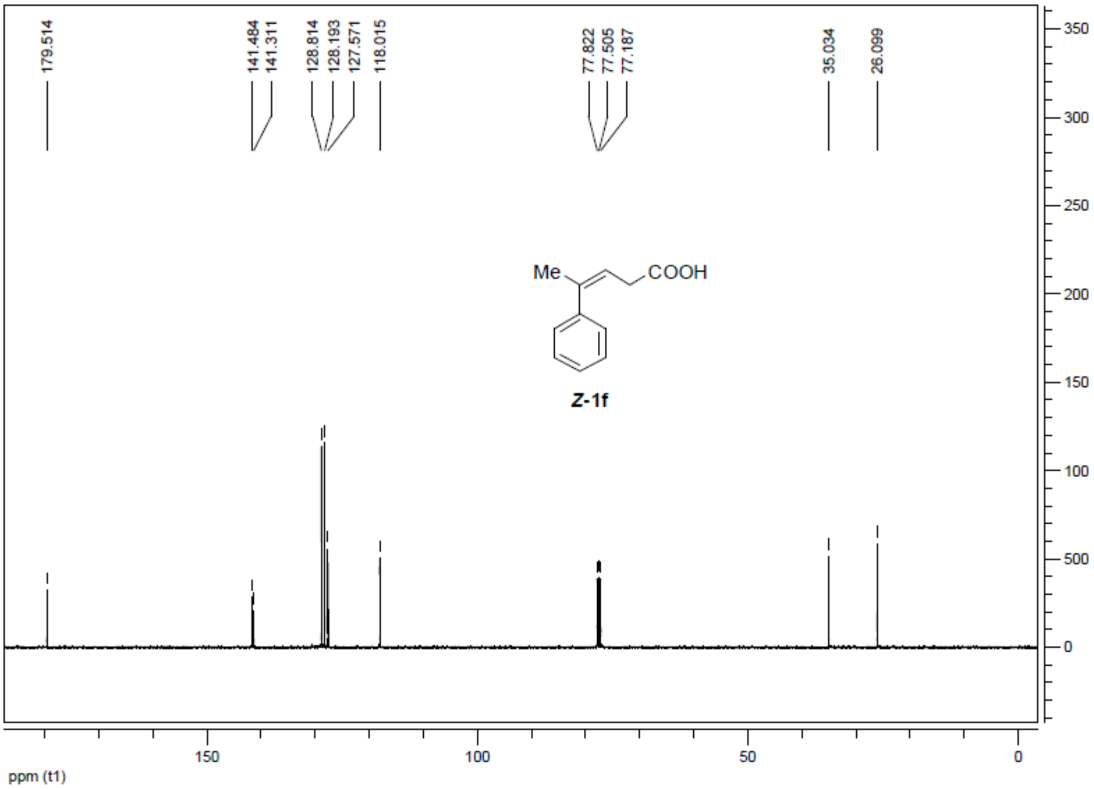
179.514
141.484
141.311
128.814
128.193
127.571
118.015
77.822
77.505
77.187
35.034
26.099
Me
COOH
Z-1f
ppm (t1)

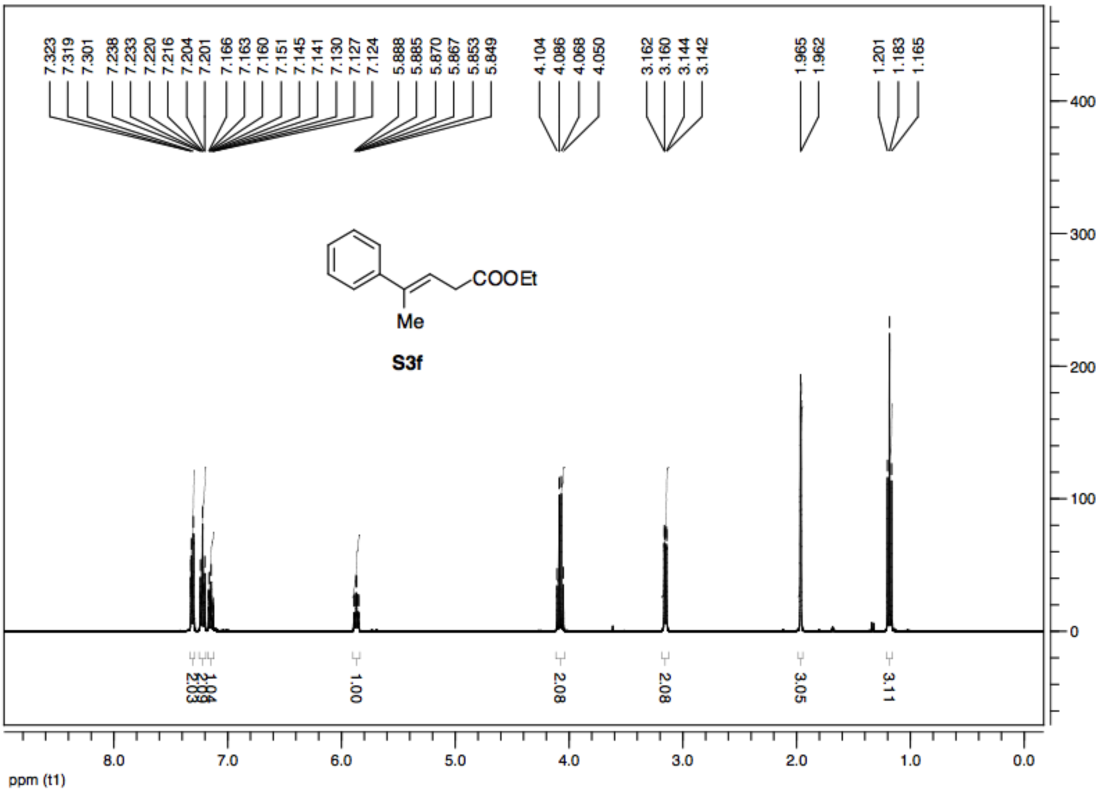

7.323
7.319
7.301
7.238
7.233
7.220
7.216
7.204
7.201
7.166
7.163
7.160
7.151
7.145
7.141
7.130
7.127
7.124
5.888
5.885
5.870
5.867
5.853
5.849
4.104
4.086
4.068
4.050
3.162
3.160
3.144
3.142
1.965
1.962
1.201
1.183
1.165
COOEt
Me
S3f
1.00
2.08
2.08
3.05
3.11
ppm (t1)

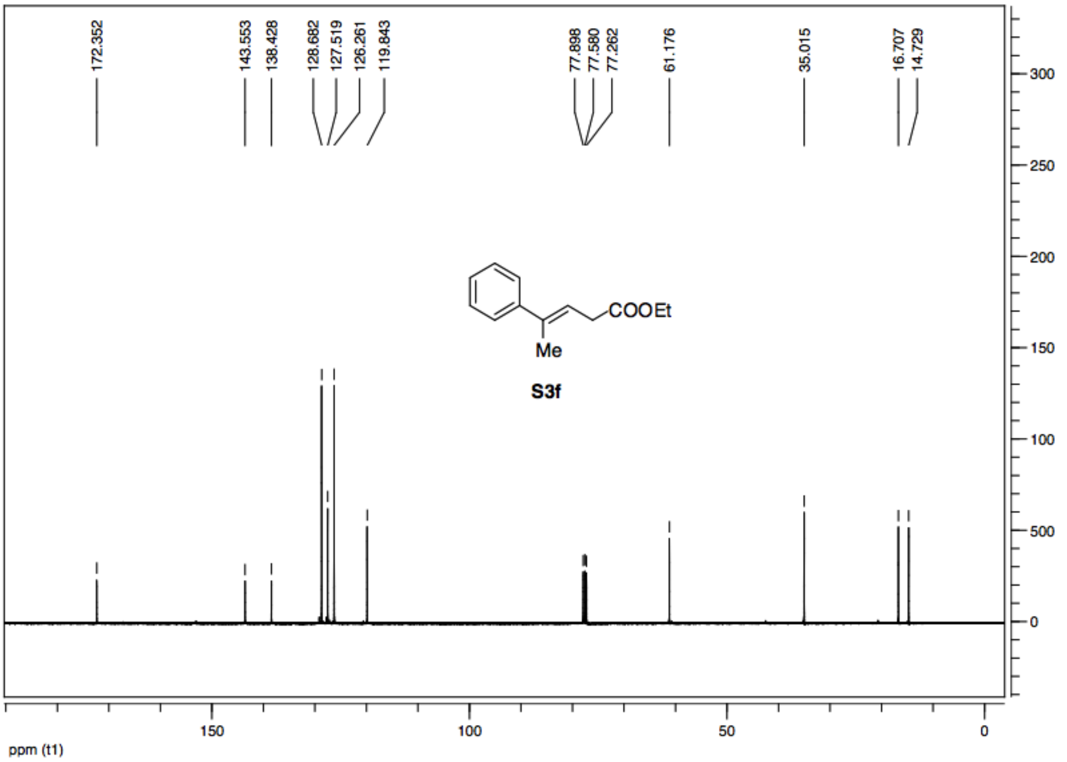

172.352
143.553
138.428
128.682
127.519
126.261
119.843
77.898
77.580
77.262
61.176
35.015
16.707
14.729
COOEt
Me
S3f
ppm (t1)

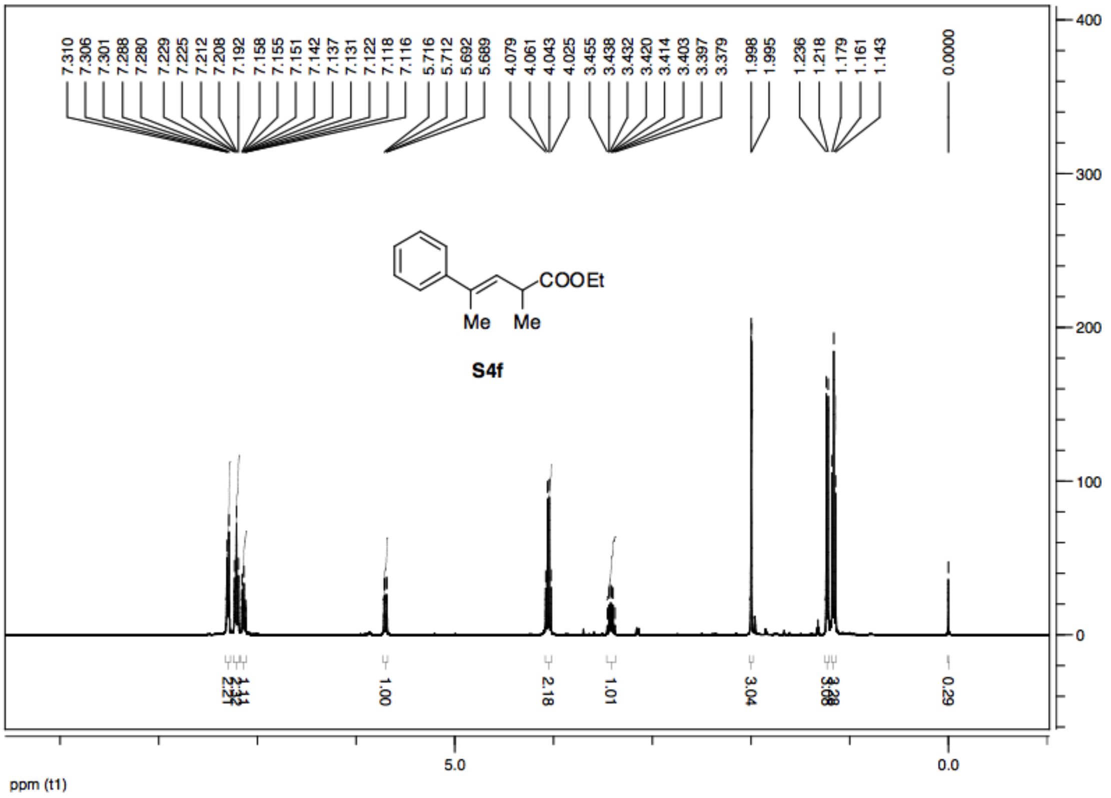
COOEt
Me
Me
S4f
ppm (t1)
5.0
0.0

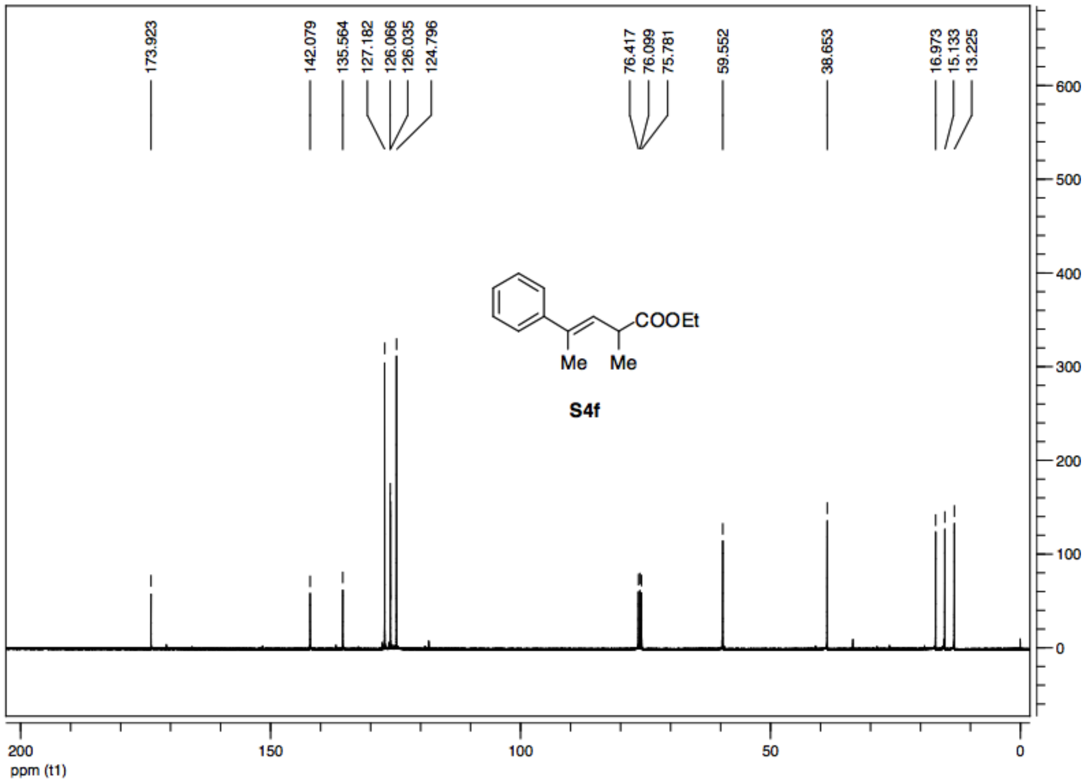
173.923
142.079
135.564
127.182
126.066
126.035
124.796
76.417
76.099
75.781
59.552
38.653
16.973
15.133
13.225
COOEt
Me
Me
S4f
200
150
100
50
0
ppm (t1)

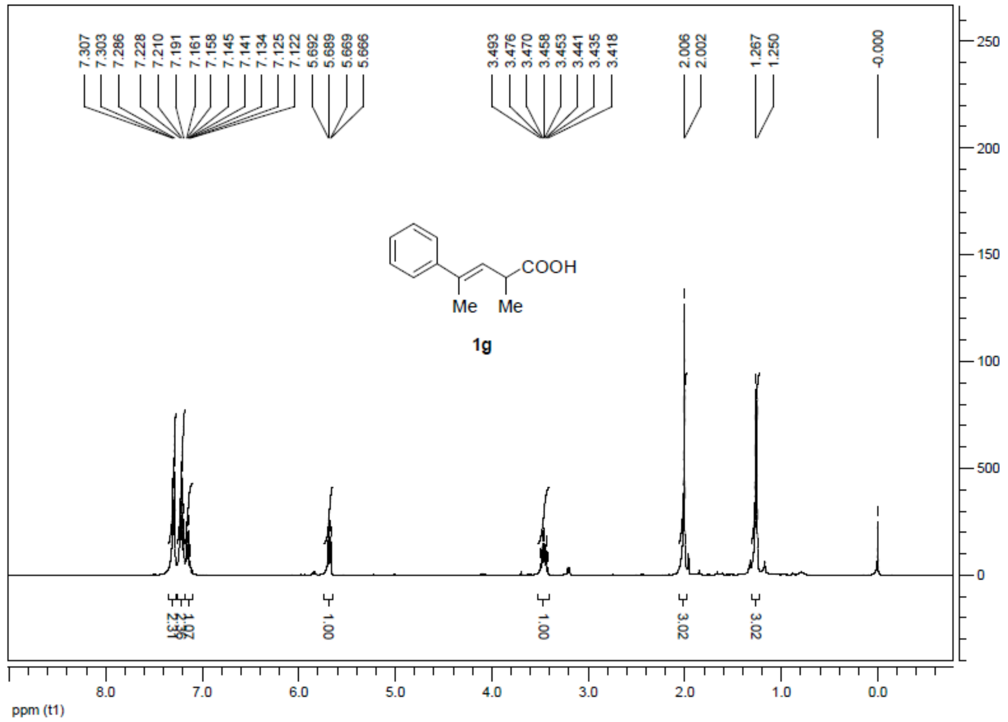
7.307
7.303
7.286
7.228
7.210
7.191
7.161
7.158
7.145
7.141
7.134
7.125
7.122
5.692
5.689
5.669
5.666
3.493
3.476
3.470
3.458
3.453
3.441
3.435
3.418
2.006
2.002
1.267
1.250
-0.000
COOH
Me
Me
1g
1.00
1.00
3.02
3.02
8.0
7.0
6.0
5.0
4.0
3.0
2.0
1.0
0.0
ppm (t1)

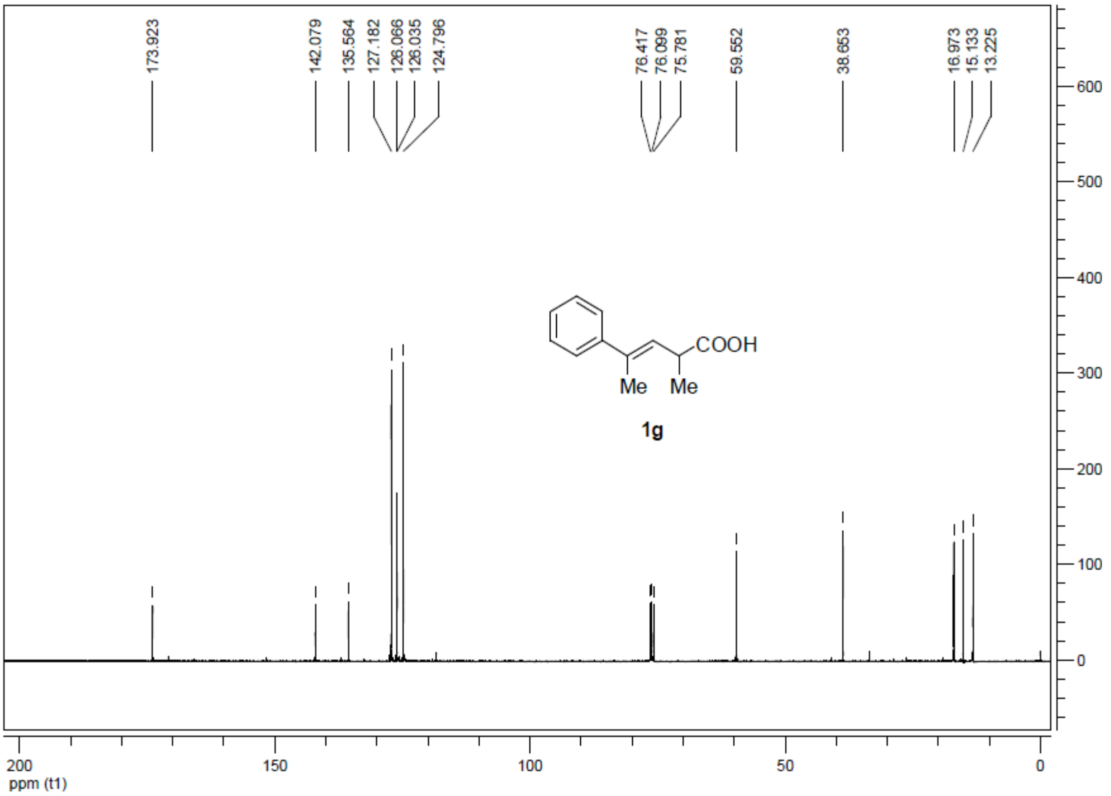
173.923
142.079
135.564
127.182
126.066
126.035
124.796
76.417
76.099
75.781
59.552
38.653
16.973
15.133
13.225
COOH
Me
Me
1g
200
150
100
50
0
ppm (t1)

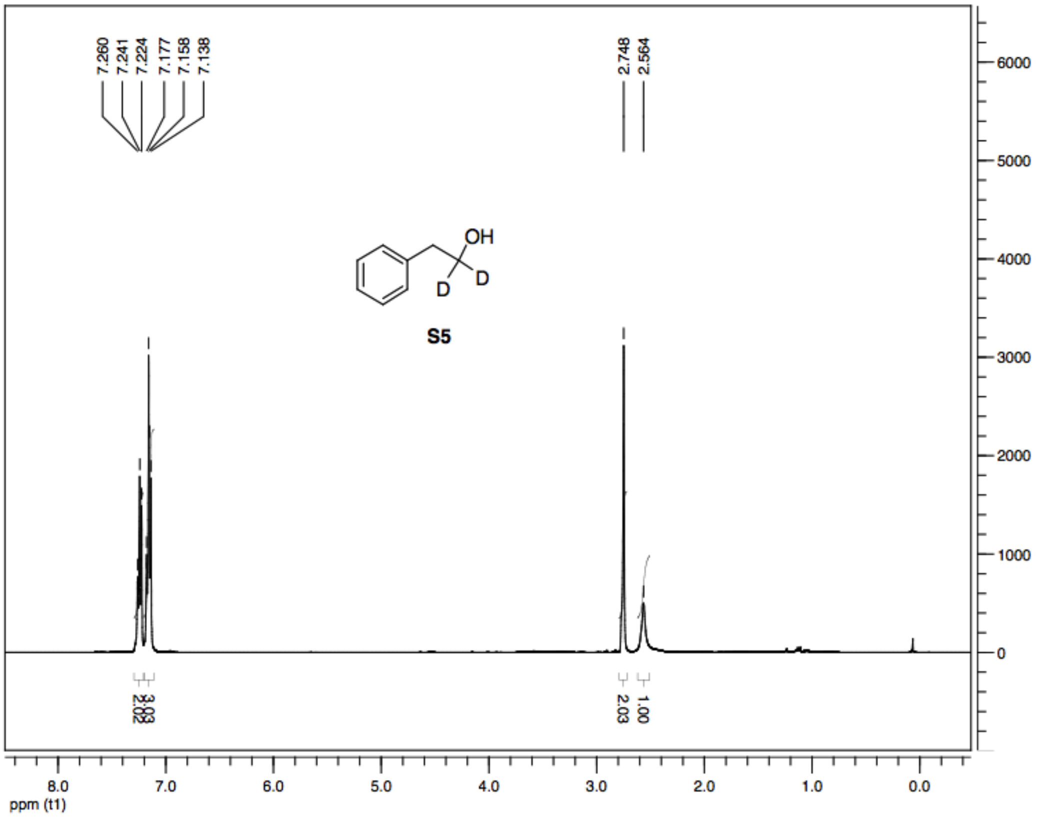
7.260
7.241
7.224
7.177
7.158
7.138
2.748
2.564
OH
D
D
S5
2.02
3.03
2.03
1.00
6000
5000
4000
3000
2000
1000
0
8.0
7.0
6.0
5.0
4.0
3.0
2.0
1.0
0.0
ppm (t1)

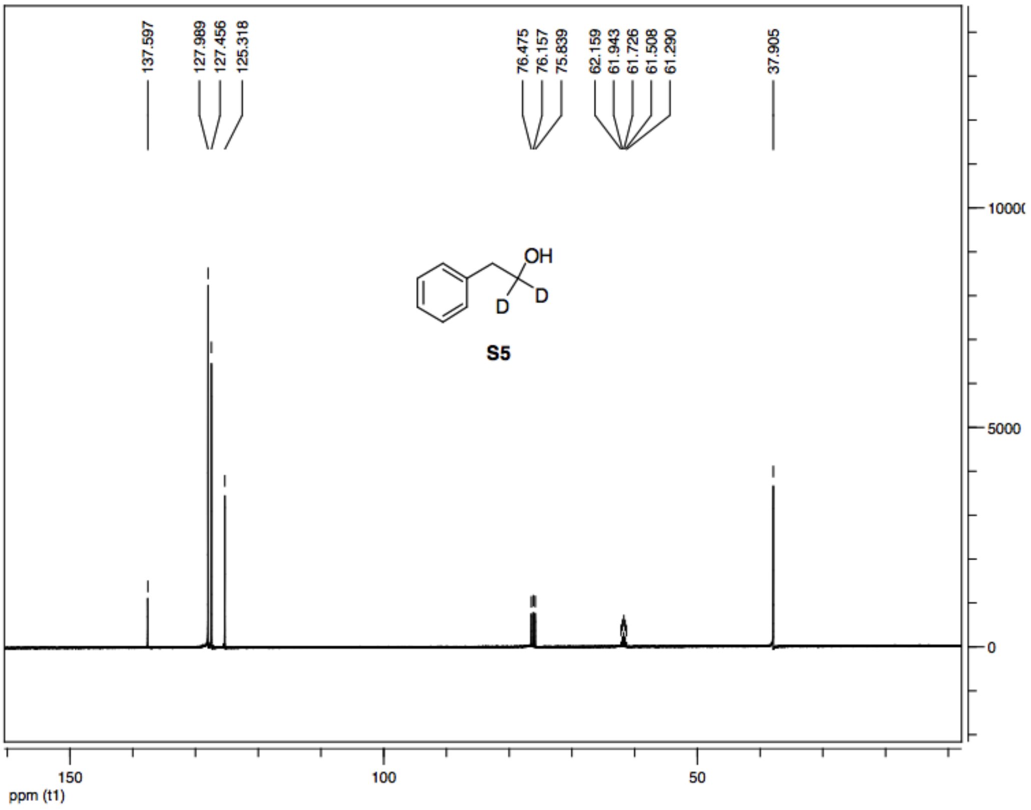
137.597
127.989
127.456
125.318
76.475
76.157
75.839
62.159
61.943
61.726
61.508
61.290
37.905
OH
D
D
S5
5000
0
150
100
50
ppm (t1)

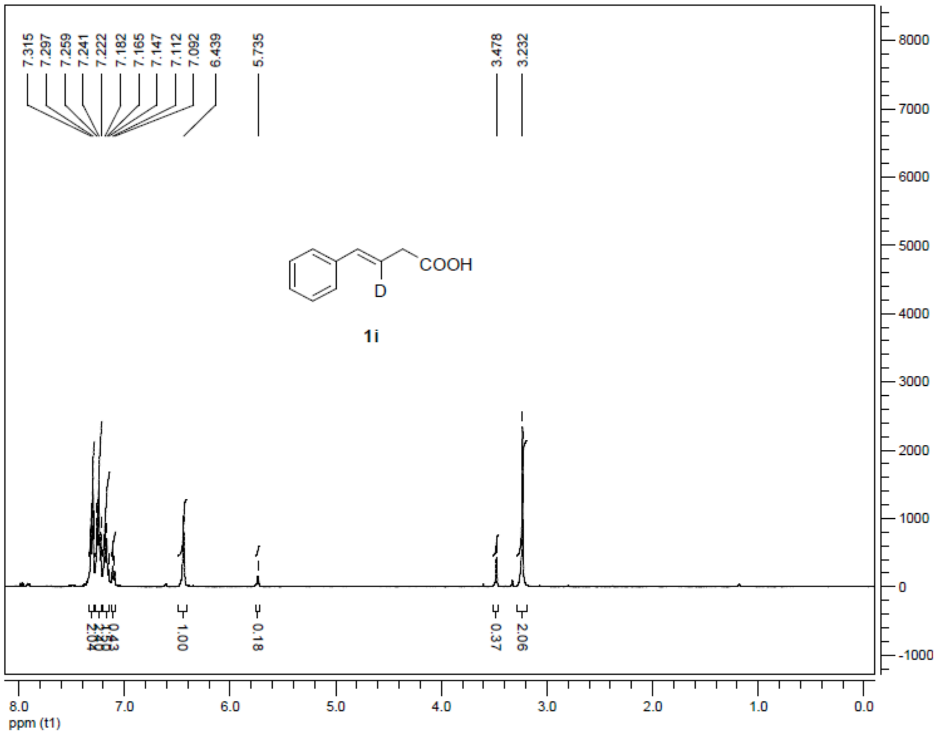
7.315
7.297
7.259
7.241
7.222
7.182
7.165
7.147
7.112
7.092
6.439
5.735
3.478
3.232
COOH
D
1i
1.00
0.18
0.37
2.06
8.0
7.0
6.0
5.0
4.0
3.0
2.0
1.0
0.0
ppm (t1)
8000
7000
6000
5000
4000
3000
2000
1000
0
-1000

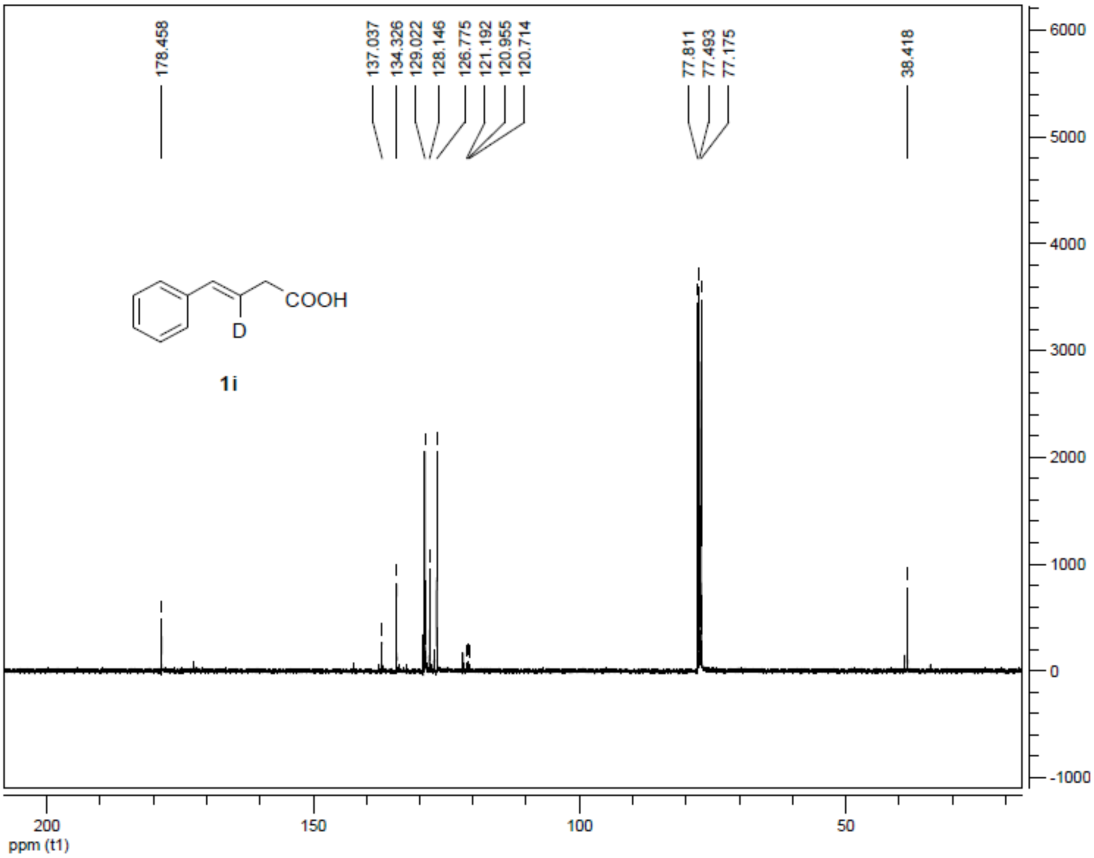
178.458
137.037
134.326
129.022
128.146
126.775
121.192
120.955
120.714
77.811
77.493
77.175
38.418
COOH
D
1i
200
150
100
50
ppm (t1)
6000
5000
4000
3000
2000
1000
0
-1000

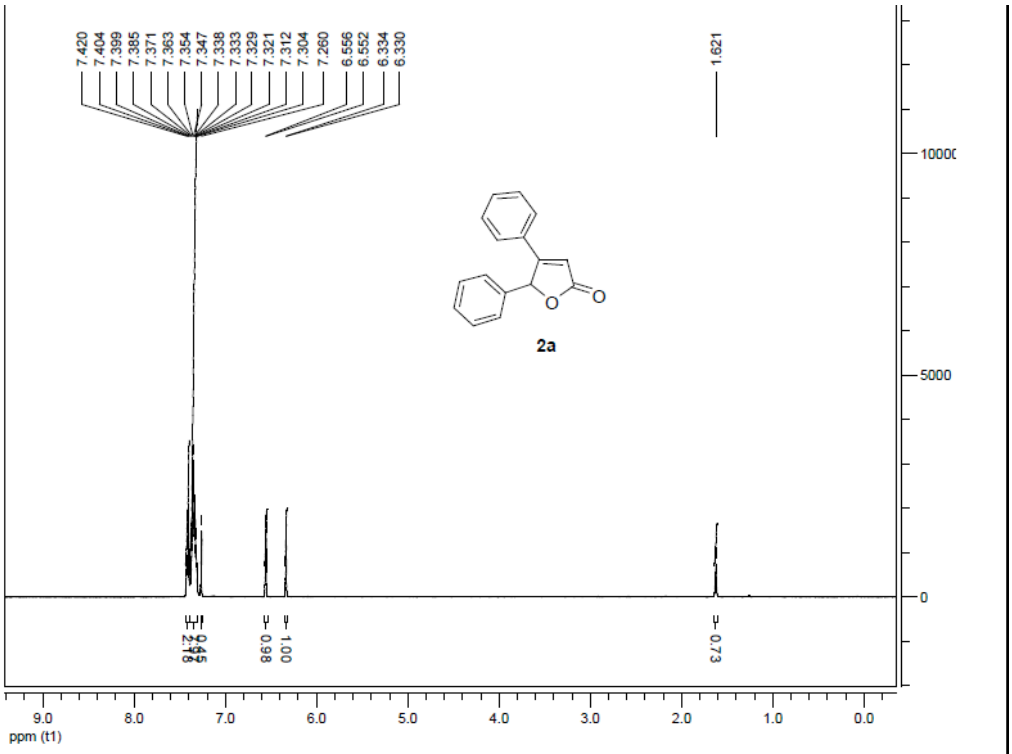
7.420
7.404
7.399
7.385
7.371
7.363
7.354
7.347
7.338
7.333
7.329
7.321
7.312
7.304
7.260
6.556
6.552
6.334
6.330
1.621
2a
2.18
0.98
1.00
0.73
ppm (t1)

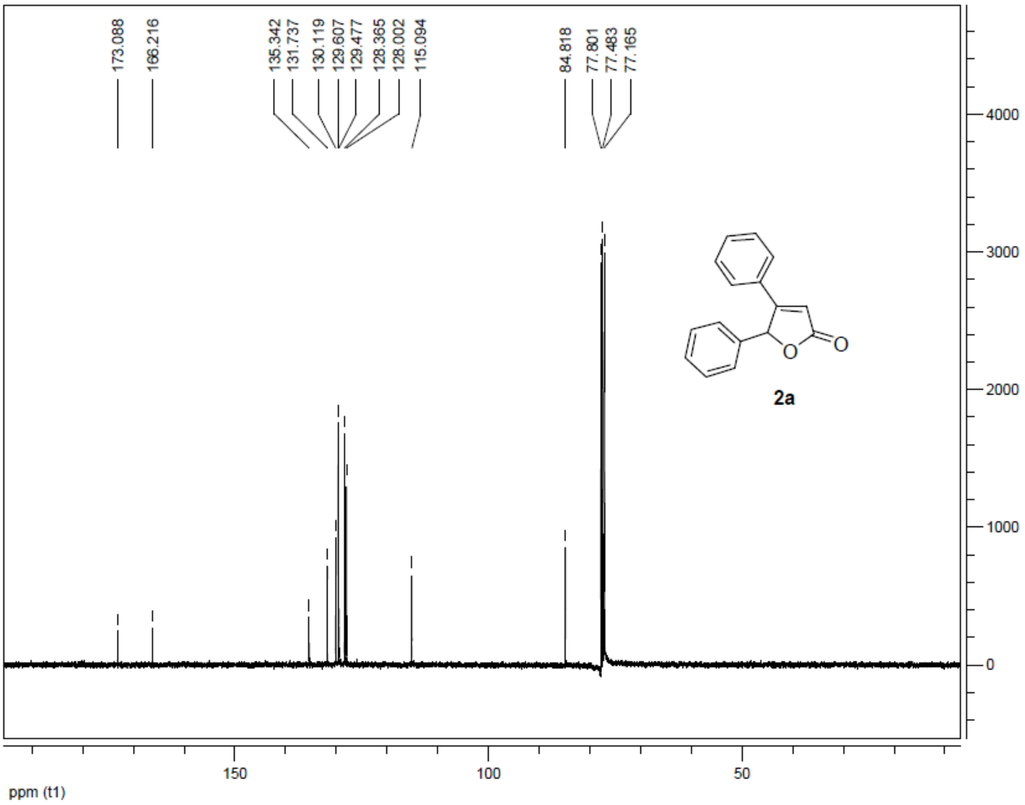
173.088
166.216
135.342
131.737
130.119
129.607
129.477
128.365
128.002
115.094
84.818
77.801
77.483
77.165
2a
ppm (t1)

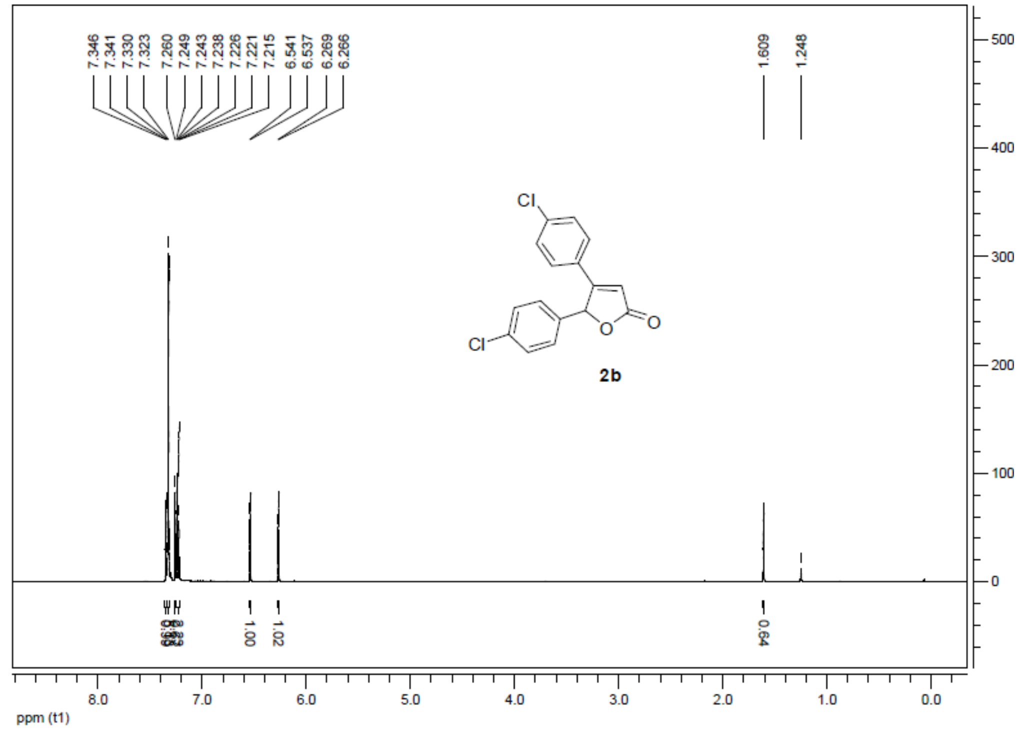

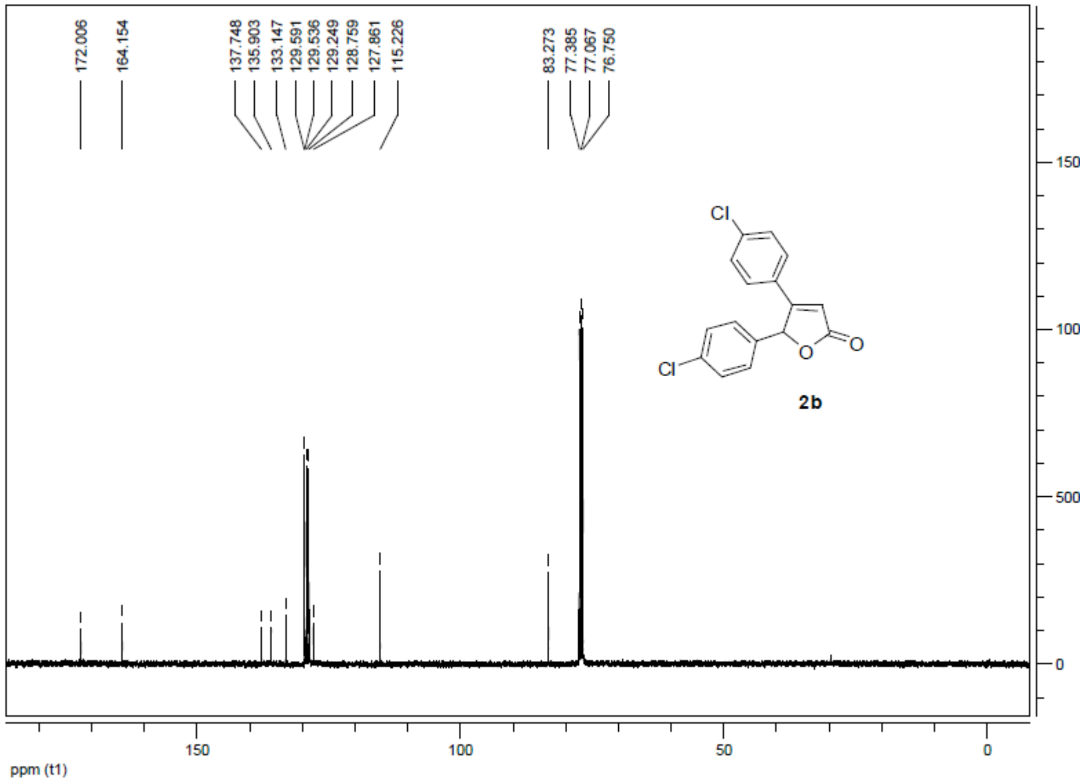

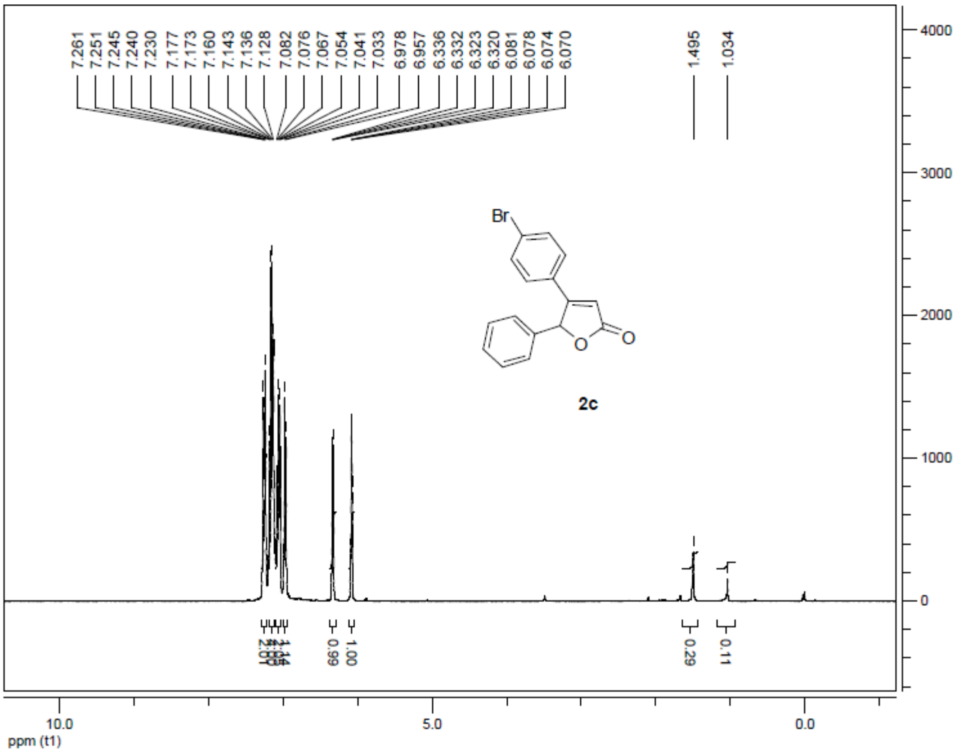
7.261
7.251
7.245
7.240
7.230
7.177
7.173
7.160
7.143
7.136
7.128
7.082
7.076
7.067
7.054
7.041
7.033
6.978
6.957
6.336
6.332
6.323
6.320
6.081
6.078
6.074
6.070
1.495
1.034
Br
O
O
2c
0.99
1.00
0.29
0.11
4000
3000
2000
1000
0
10.0
5.0
0.0
ppm (t1)

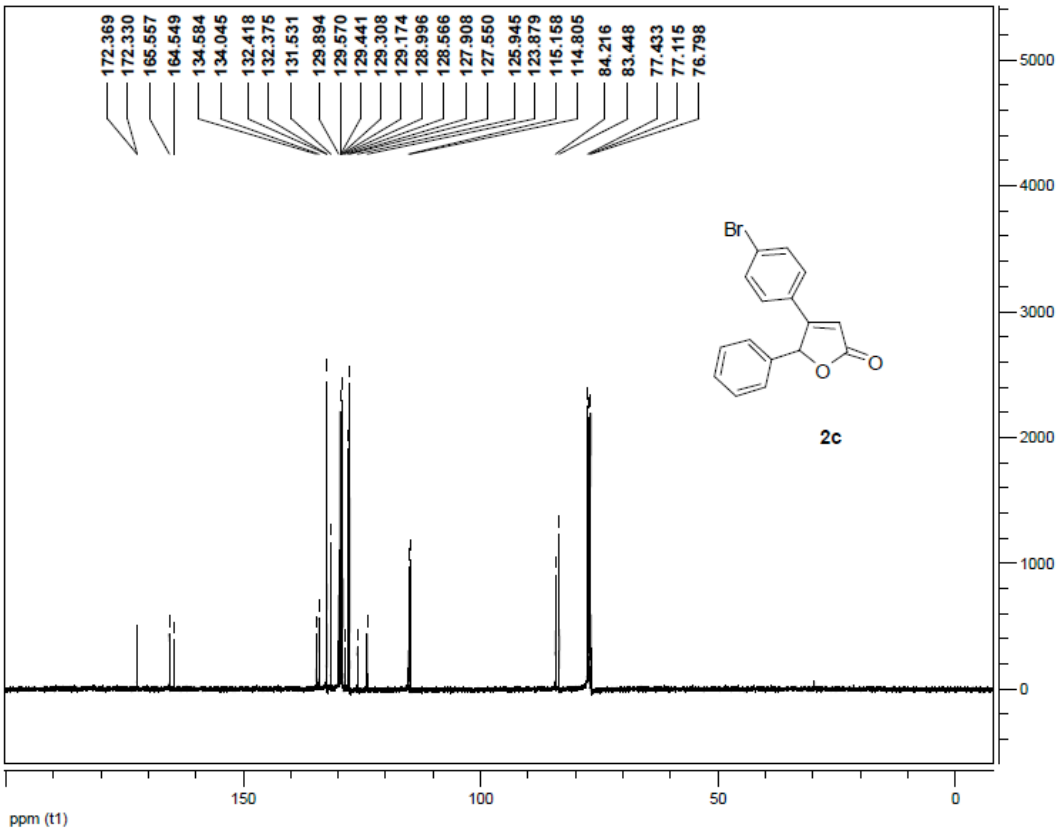
172.369
172.330
165.557
164.549
134.584
134.045
132.418
132.375
131.531
129.894
129.570
129.441
129.308
129.174
128.996
128.566
127.908
127.550
125.945
123.879
115.158
114.805
84.216
83.448
77.433
77.115
76.798
Br
O
O
2c
5000
4000
3000
2000
1000
0
150
100
50
0
ppm (t1)

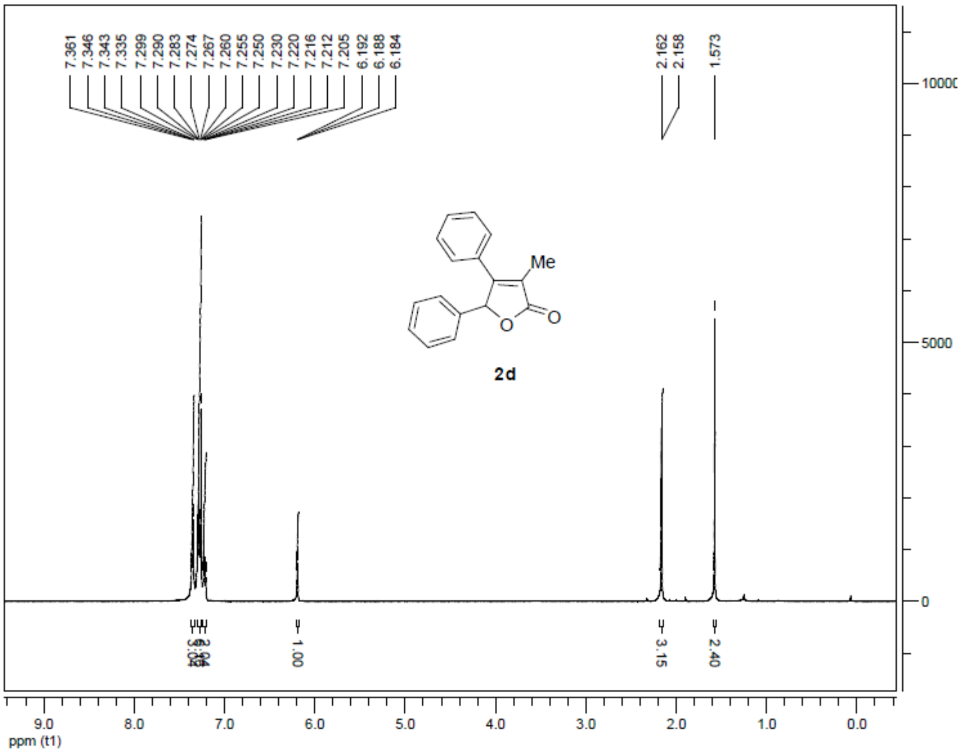

7.361
7.346
7.343
7.335
7.299
7.290
7.283
7.274
7.267
7.260
7.255
7.250
7.230
7.220
7.216
7.212
7.205
6.192
6.188
6.184
2.162
2.158
1.573
Me
O
O
2d
1.00
3.15
2.40
10000
5000
0
9.0
8.0
7.0
6.0
5.0
4.0
3.0
2.0
1.0
0.0
ppm (t1)

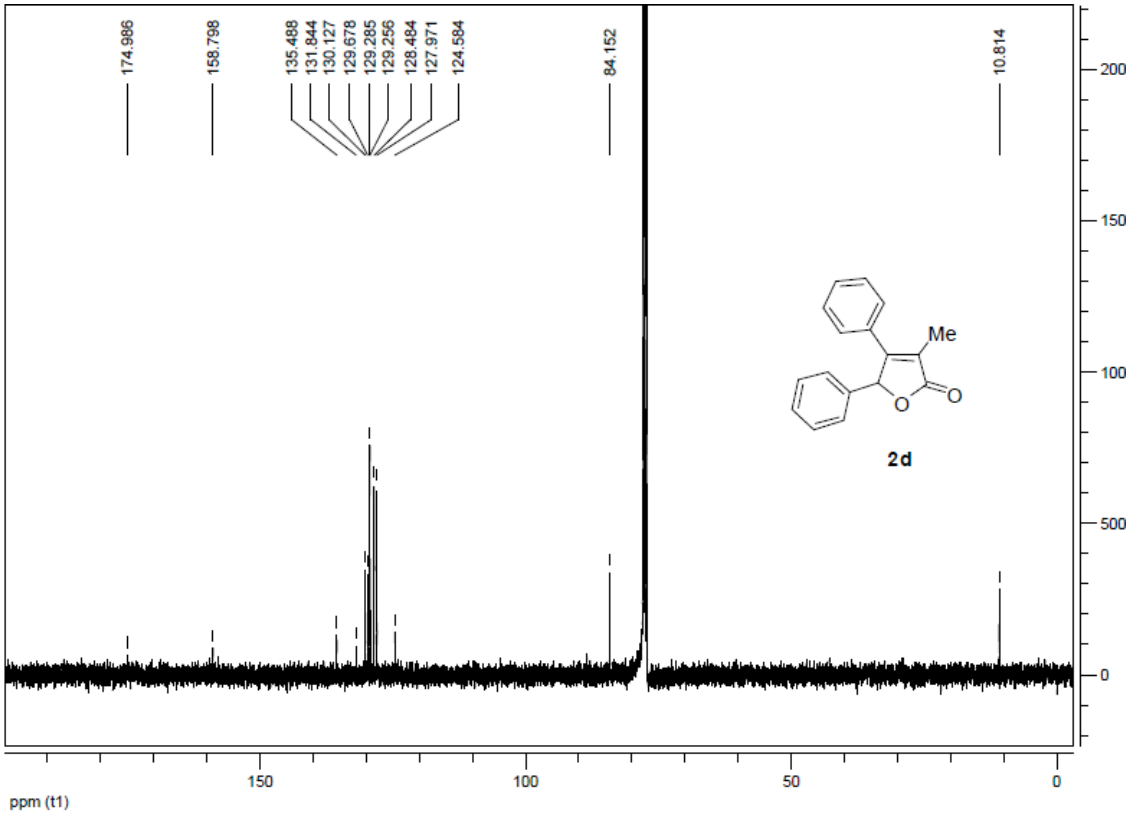

174.986
158.798
135.488
131.844
130.127
129.678
129.285
129.256
128.484
127.971
124.584
84.152
10.814
Me
O
O
2d
200
150
100
500
0
150
100
50
0
ppm (t1)

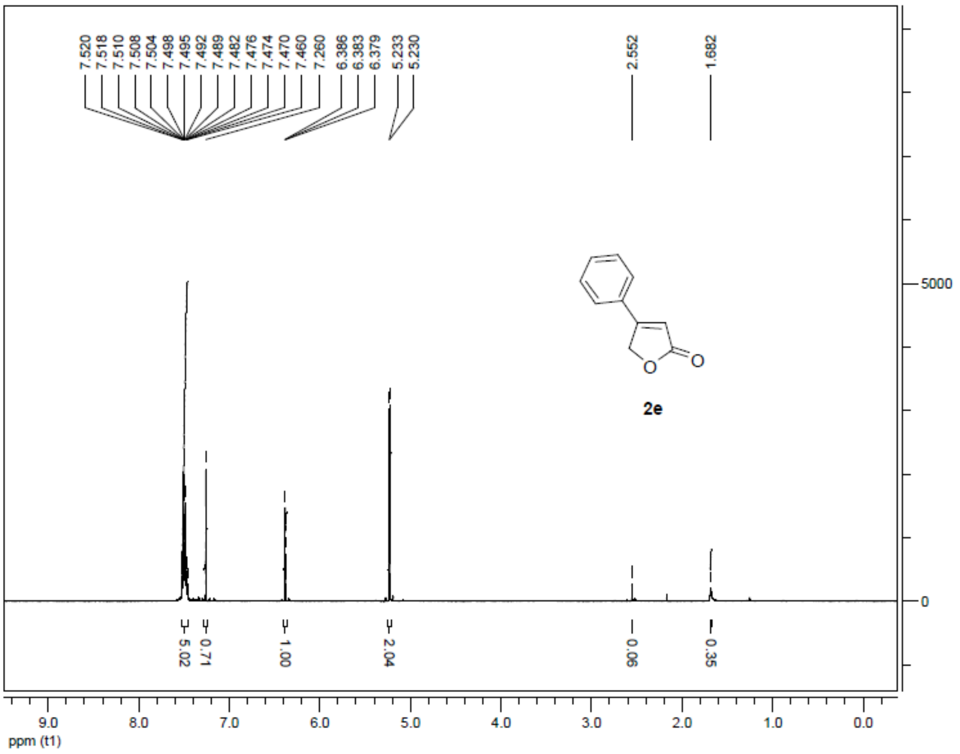

7.520
7.518
7.510
7.508
7.504
7.498
7.495
7.492
7.489
7.482
7.476
7.474
7.470
7.460
7.260
6.386
6.383
6.379
5.233
5.230
2.552
1.682
2e
5.02
0.71
1.00
2.04
0.06
0.35
5000
0
9.0
8.0
7.0
6.0
5.0
4.0
3.0
2.0
1.0
0.0
ppm (t1)

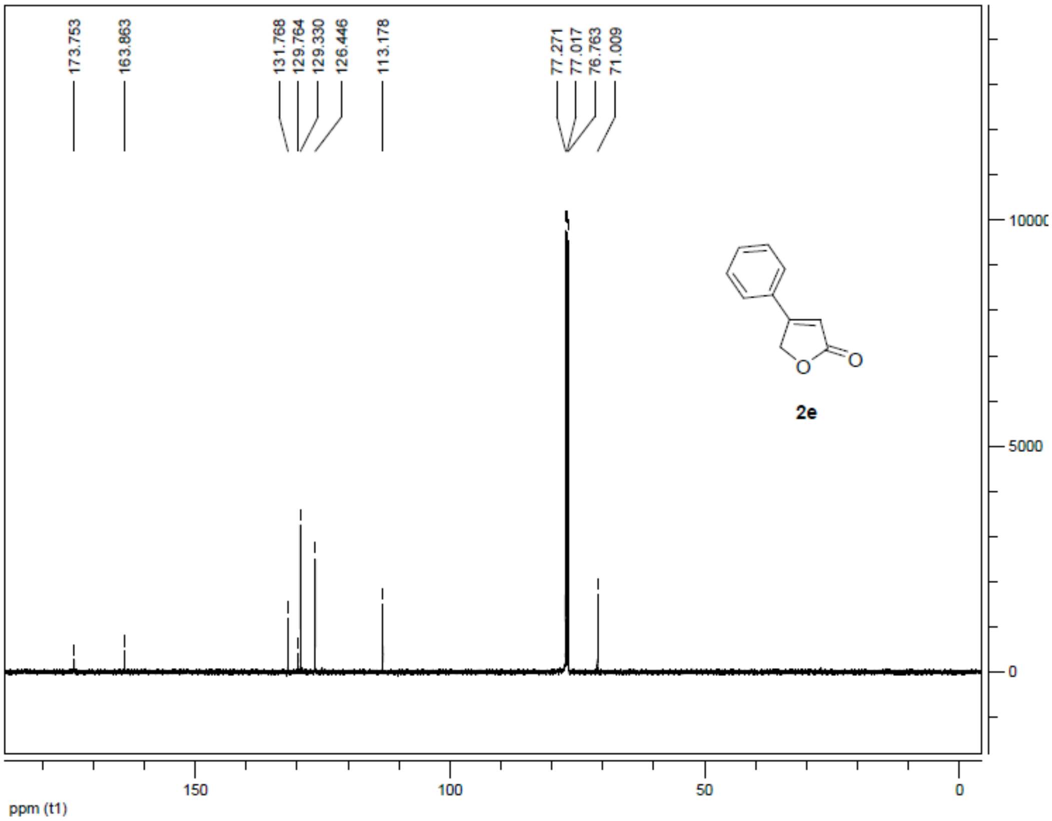

173.753
163.863
131.768
129.764
129.330
126.446
113.178
77.271
77.017
76.763
71.009
2e
10000
5000
0
150
100
50
0
ppm (t1)

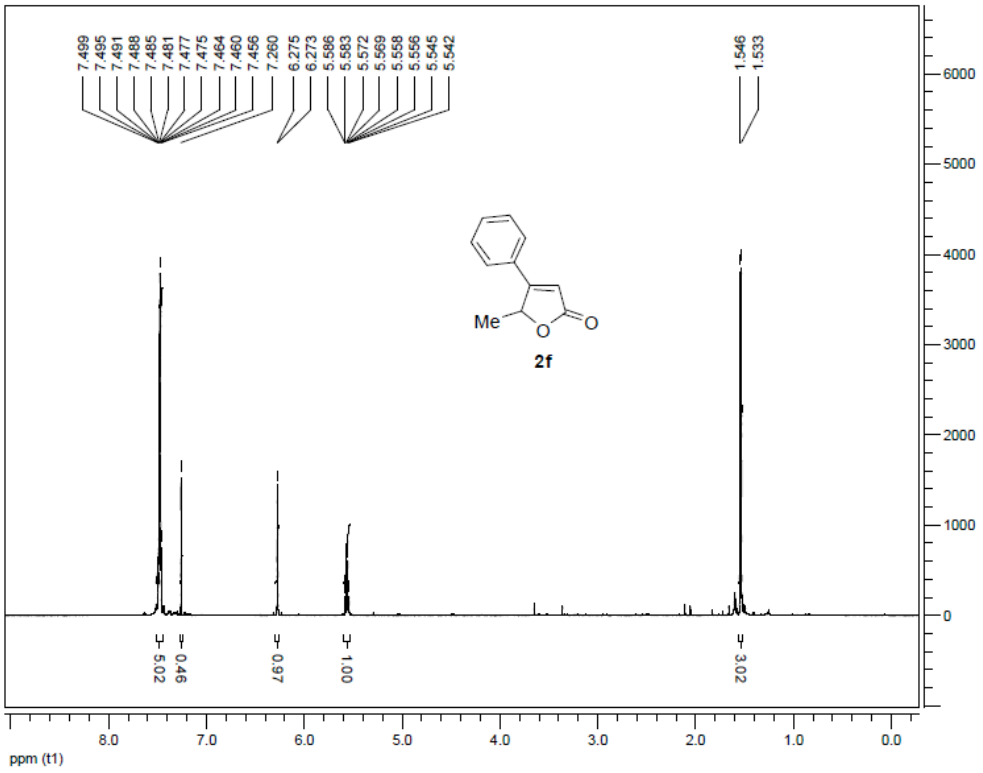

7.499
7.495
7.491
7.488
7.485
7.481
7.477
7.475
7.464
7.460
7.456
7.260
6.275
6.273
5.586
5.583
5.572
5.569
5.558
5.556
5.545
5.542
1.546
1.533
Me
O
O
2f
5.02
0.46
0.97
1.00
3.02
ppm (t1)

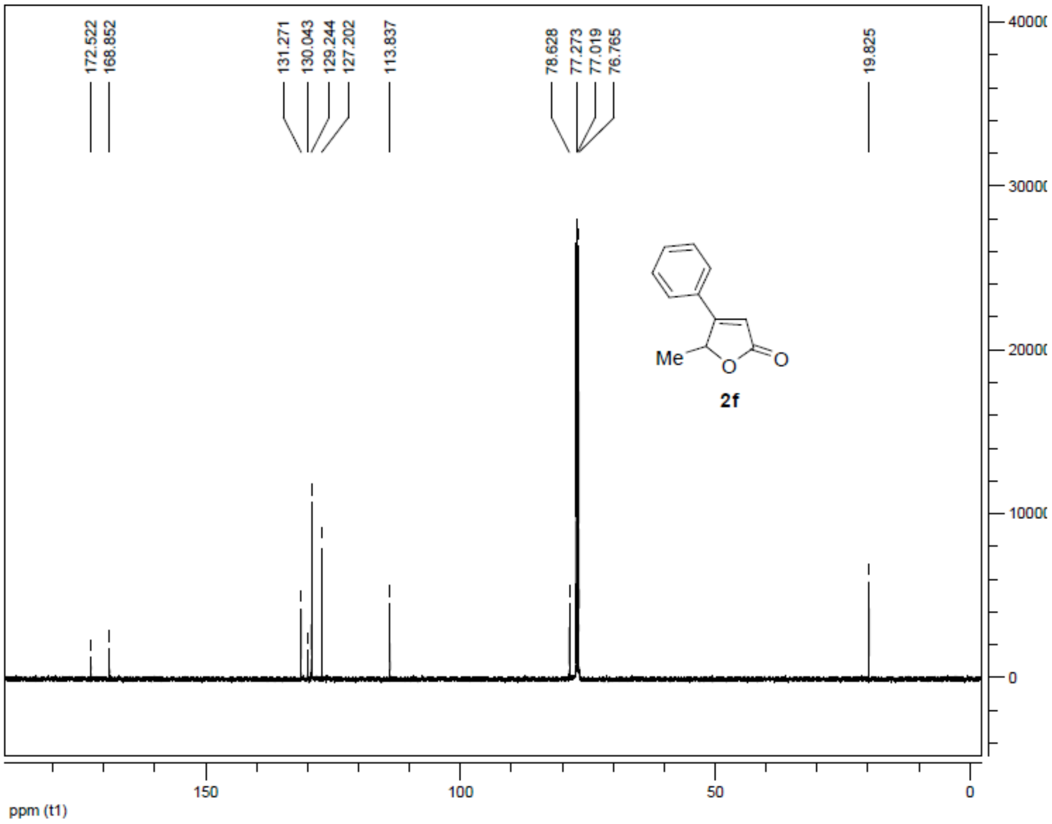

172.522
168.852
131.271
130.043
129.244
127.202
113.837
78.628
77.273
77.019
76.765
19.825
Me
O
O
2f
ppm (t1)

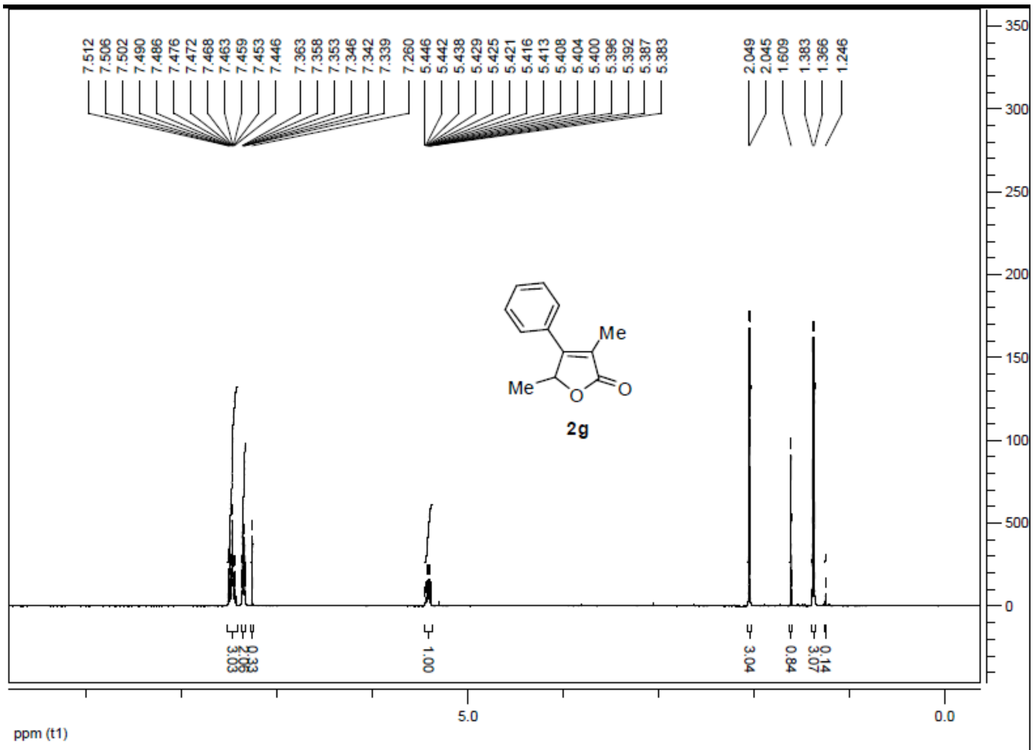
7.512 7.506 7.502 7.490 7.486 7.476 7.472 7.468 7.463 7.459 7.453 7.446 7.363 7.358 7.353 7.346 7.342 7.339 7.260 5.446 5.442 5.438 5.429 5.425 5.421 5.416 5.413 5.408 5.404 5.400 5.396 5.392 5.387 5.383
2.049 2.045 1.609 1.383 1.366 1.246
Me
Me
O
O
2g
3.03 2.06 0.33
1.00
3.04
0.84
3.07 0.14
5.0
0.0
ppm (t1)

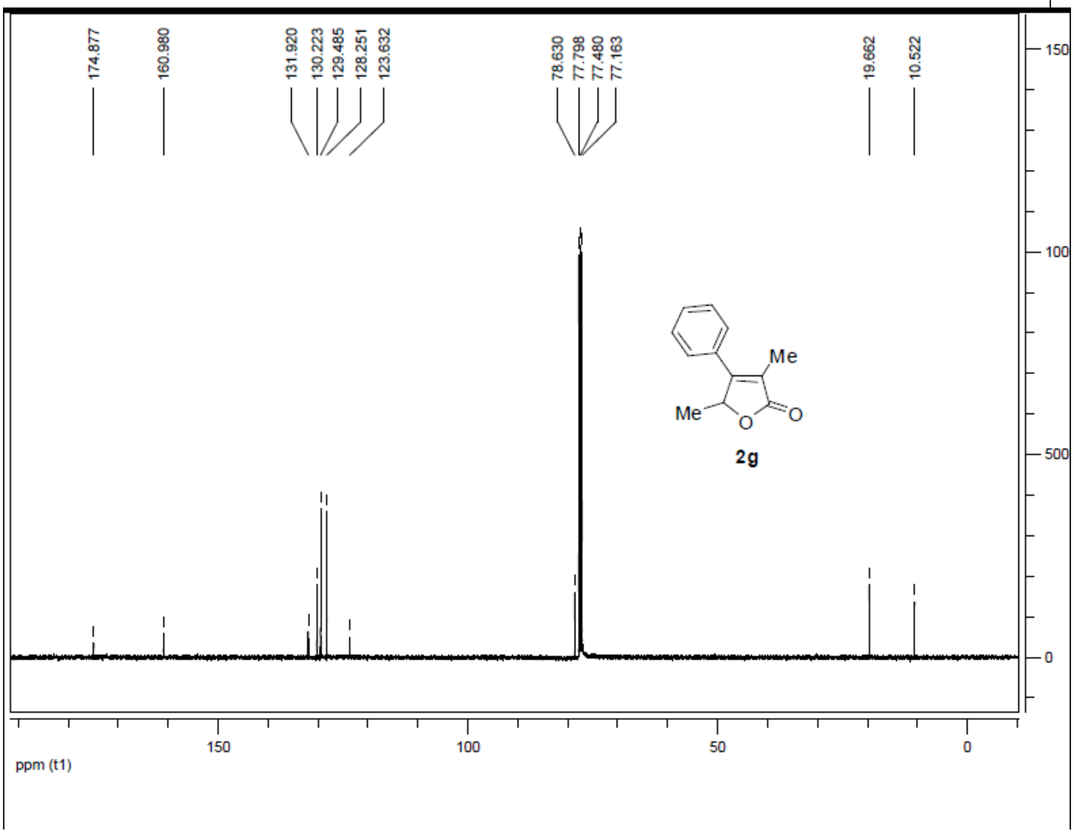
174.877
160.980
131.920 130.223 129.485 128.251 123.632
78.630 77.798 77.480 77.163
19.662
10.522
Me
Me
O
O
2g
150
100
50
0
ppm (t1)

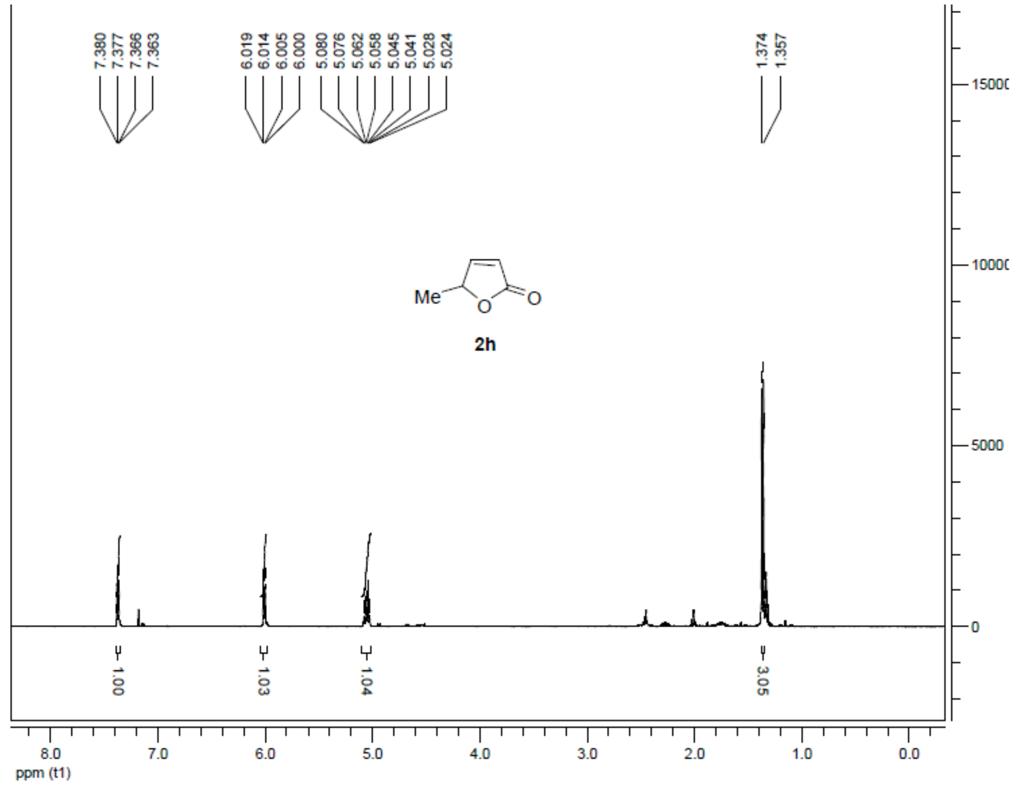
7.380
7.377
7.366
7.363
6.019
6.014
6.005
6.000
5.080
5.076
5.062
5.058
5.045
5.041
5.028
5.024
1.374
1.357
Me
O
O
2h
1.00
1.03
1.04
3.05
ppm (t1)

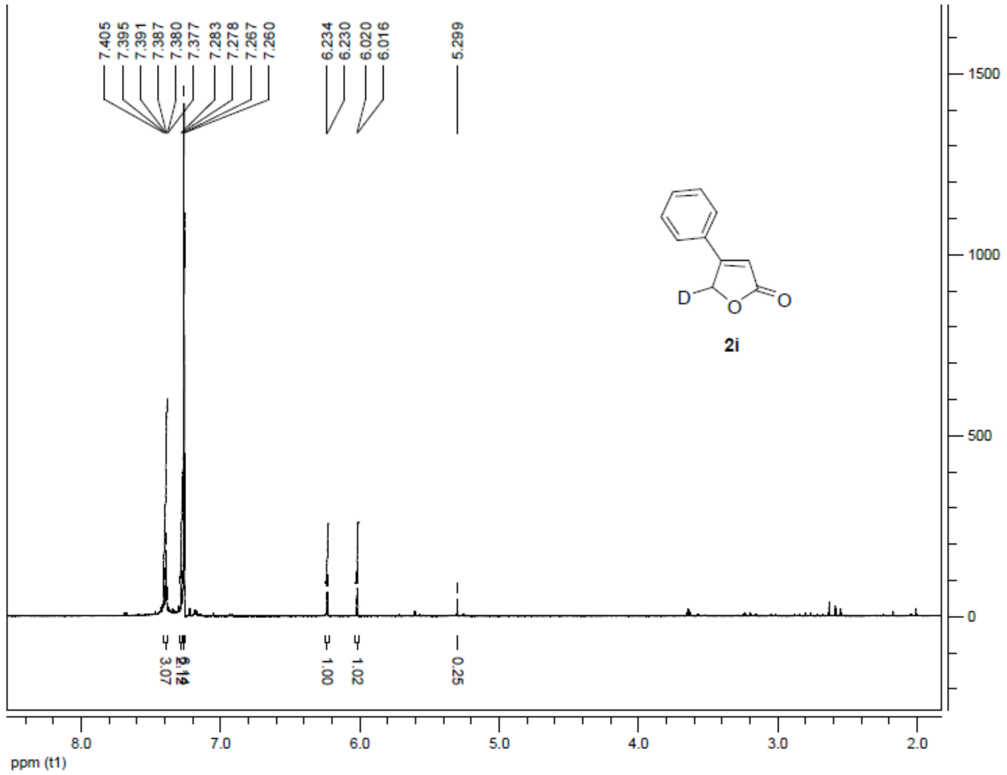
7.405
7.395
7.391
7.387
7.380
7.377
7.283
7.278
7.267
7.260
6.234
6.230
6.020
6.016
5.299
D
O
O
2i
3.07
1.00
1.02
0.25
ppm (t1)

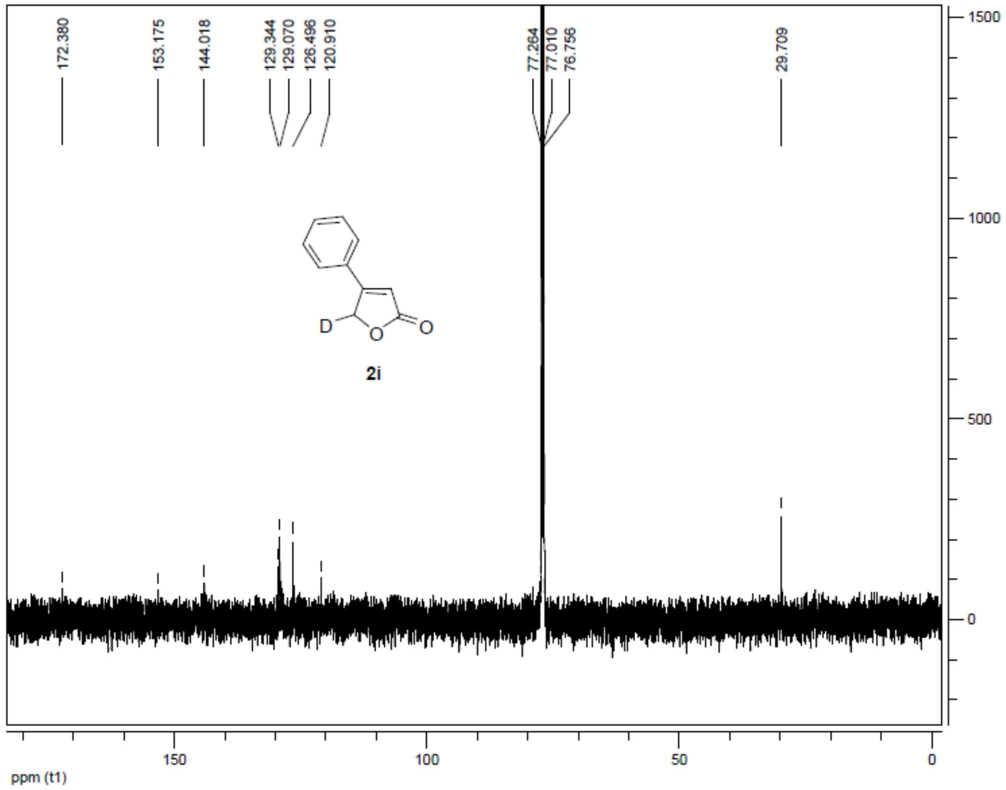
172.380
153.175
144.018
129.344
129.070
126.496
120.910
77.264
77.010
76.756
29.709
D
O
O
2i
1500
1000
500
0
150
100
50
0
ppm (t1)

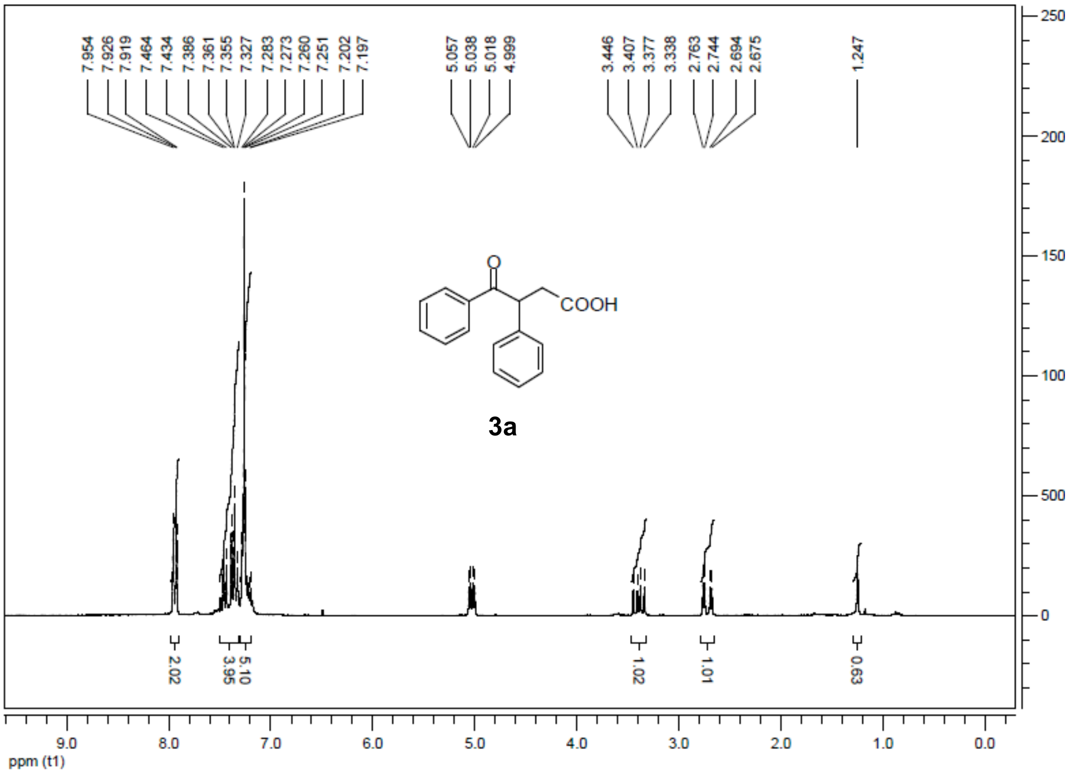
7.954
7.926
7.919
7.464
7.434
7.386
7.361
7.355
7.327
7.283
7.273
7.260
7.251
7.202
7.197
5.057
5.038
5.018
4.999
3.446
3.407
3.377
3.338
2.763
2.744
2.694
2.675
1.247
O
COOH
3a
2.02
3.95
5.10
1.02
1.01
0.63
250
200
150
100
500
0
9.0
8.0
7.0
6.0
5.0
4.0
3.0
2.0
1.0
0.0
ppm (t1)

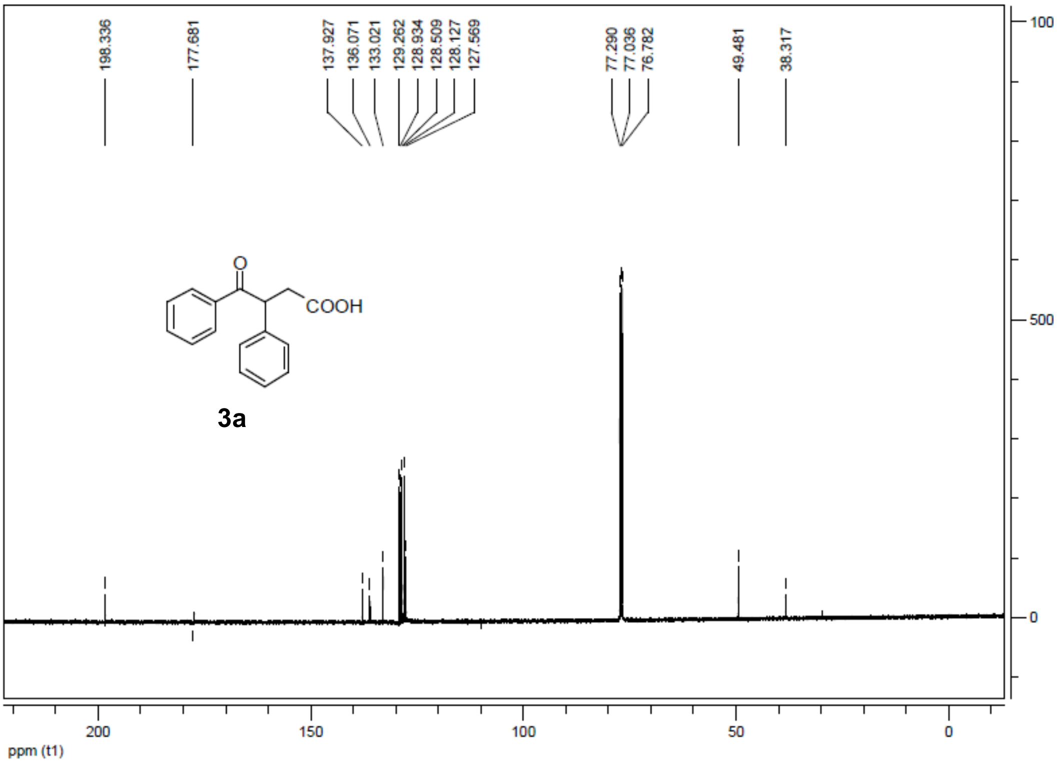
198.336
177.681
137.927
136.071
133.021
129.262
128.934
128.509
128.127
127.569
77.290
77.036
76.782
49.481
38.317
COOH
O
3a
100
500
0
200
150
100
50
0
ppm (t1)

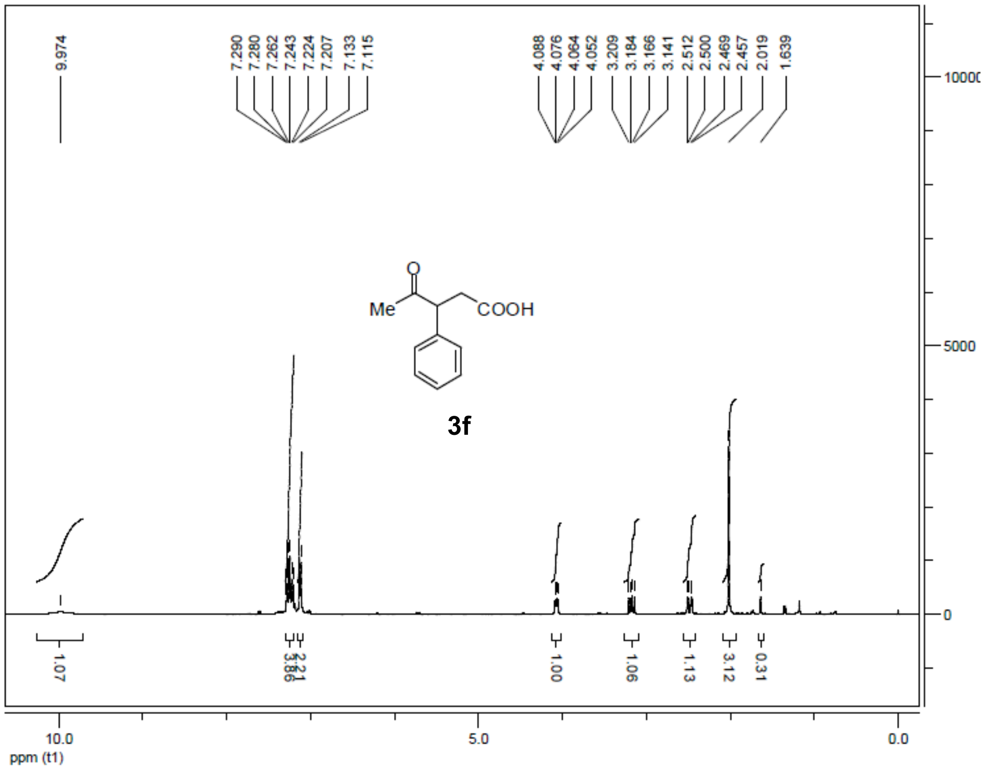
9.974
7.290
7.280
7.262
7.243
7.224
7.207
7.133
7.115
4.088
4.076
4.064
4.052
3.209
3.184
3.166
3.141
2.512
2.500
2.469
2.457
2.019
1.639
Me
COOH
O
3f
10000
5000
0
1.07
3.86
2.21
1.00
1.06
1.13
3.12
0.31
10.0
5.0
0.0
ppm (t1)

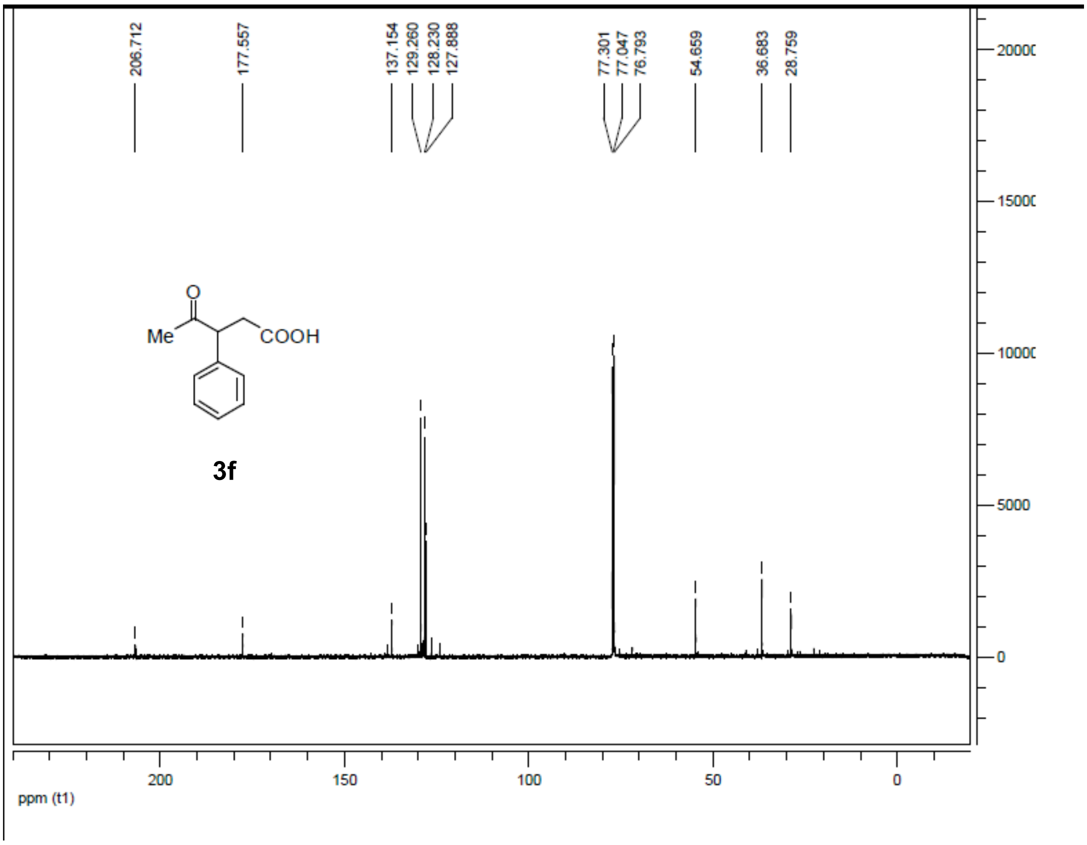
206.712
177.557
137.154
129.260
128.230
127.888
77.301
77.047
76.793
54.659
36.683
28.759
Me
O
COOH
3f
20000
15000
10000
5000
0
200
150
100
50
0
ppm (t1)

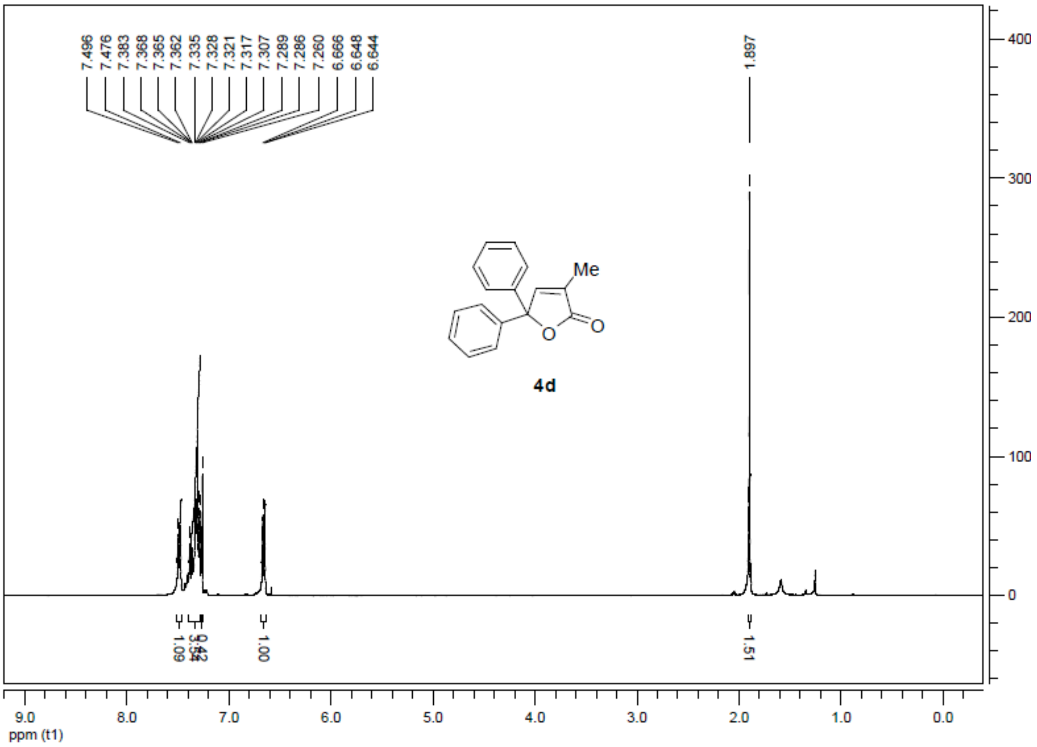
7.496
7.476
7.383
7.368
7.365
7.362
7.335
7.328
7.321
7.317
7.307
7.289
7.286
7.260
6.666
6.648
6.644
1.897
Me
O
O
4d
400
300
200
100
0
1.09
1.00
1.51
9.0
8.0
7.0
6.0
5.0
4.0
3.0
2.0
1.0
0.0
ppm (t1)

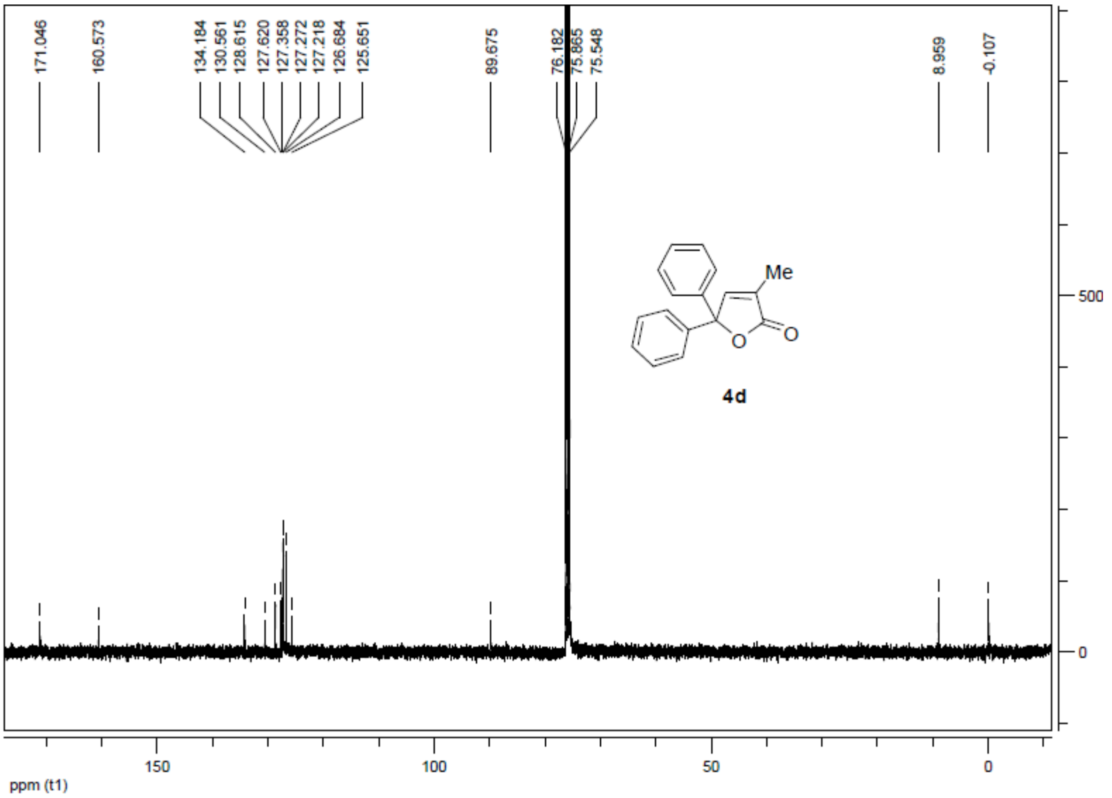
171.046
160.573
134.184
130.561
128.615
127.620
127.358
127.272
127.218
126.684
125.651
89.675
76.182
75.865
75.548
8.959
-0.107
Me
O
O
4d
500
0
150
100
50
0
ppm (t1)

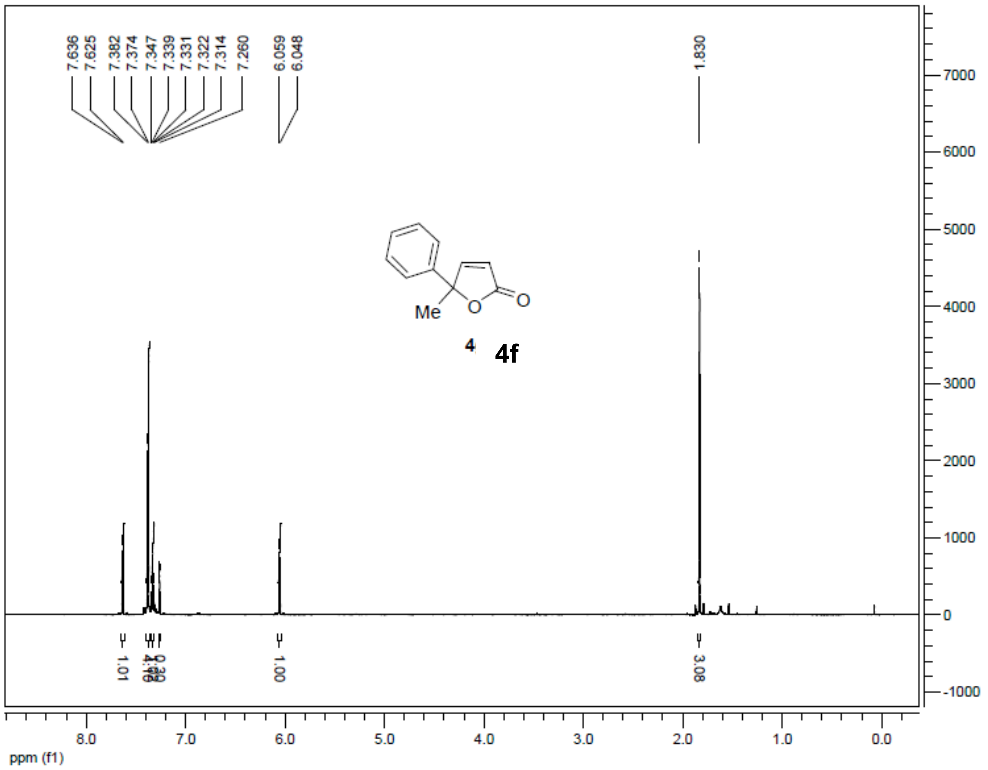
7.636
7.625
7.382
7.374
7.347
7.339
7.331
7.322
7.314
7.260
6.059
6.048
1.830
Me
O
O
4
4f
7000
6000
5000
4000
3000
2000
1000
0
-1000
1.01
1.00
3.08
8.0
7.0
6.0
5.0
4.0
3.0
2.0
1.0
0.0
ppm (f1)

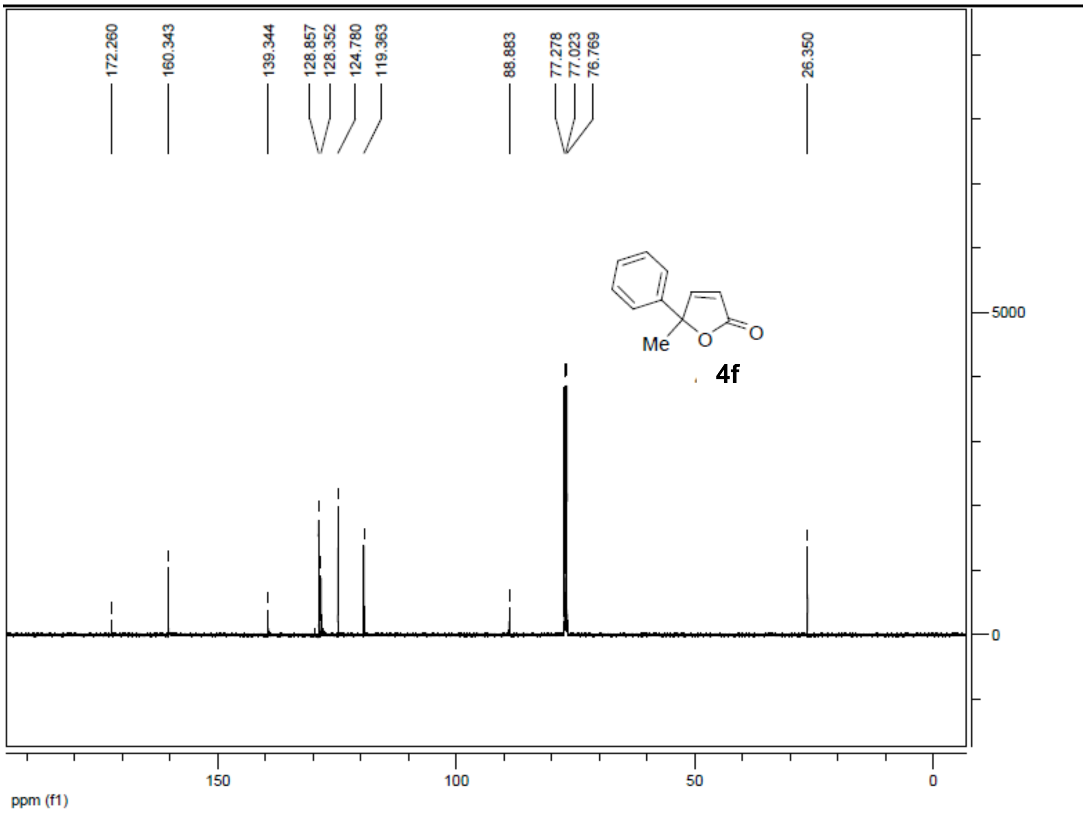
172.260
160.343
139.344
128.857
128.352
124.780
119.363
88.883
77.278
77.023
76.769
26.350
Me
O
O
4f
5000
0
150
100
50
0
ppm (f1)

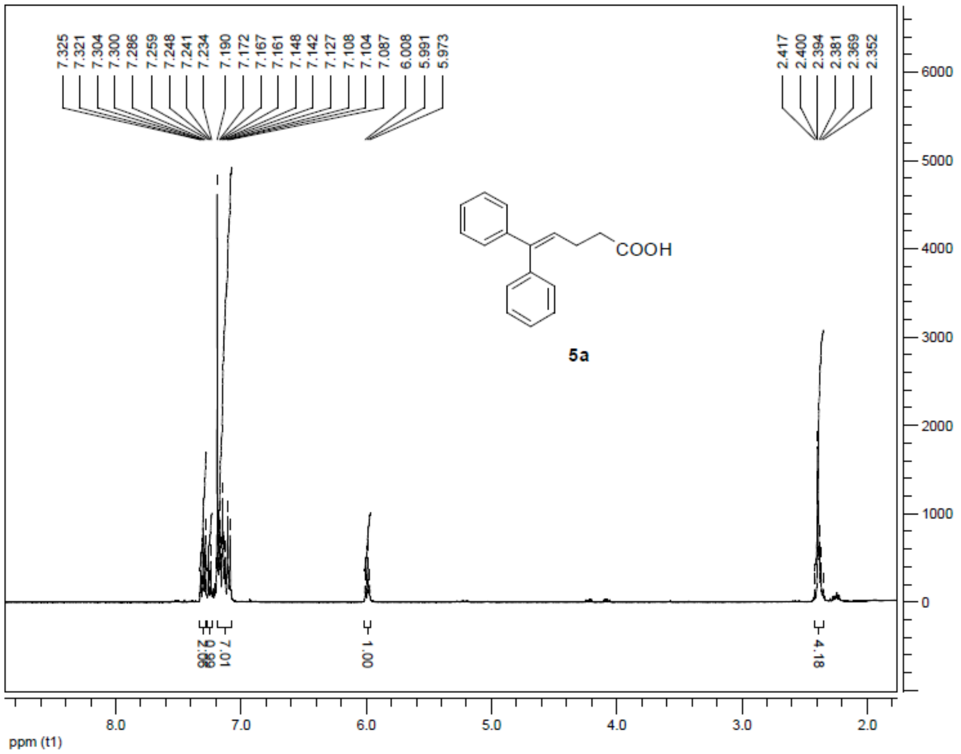
7.325
7.321
7.304
7.300
7.286
7.259
7.248
7.241
7.234
7.190
7.172
7.167
7.161
7.148
7.142
7.127
7.108
7.104
7.087
6.008
5.991
5.973
2.417
2.400
2.394
2.381
2.369
2.352
COOH
5a
7.01
1.00
4.18
6000
5000
4000
3000
2000
1000
0
8.0
7.0
6.0
5.0
4.0
3.0
2.0
ppm (t1)

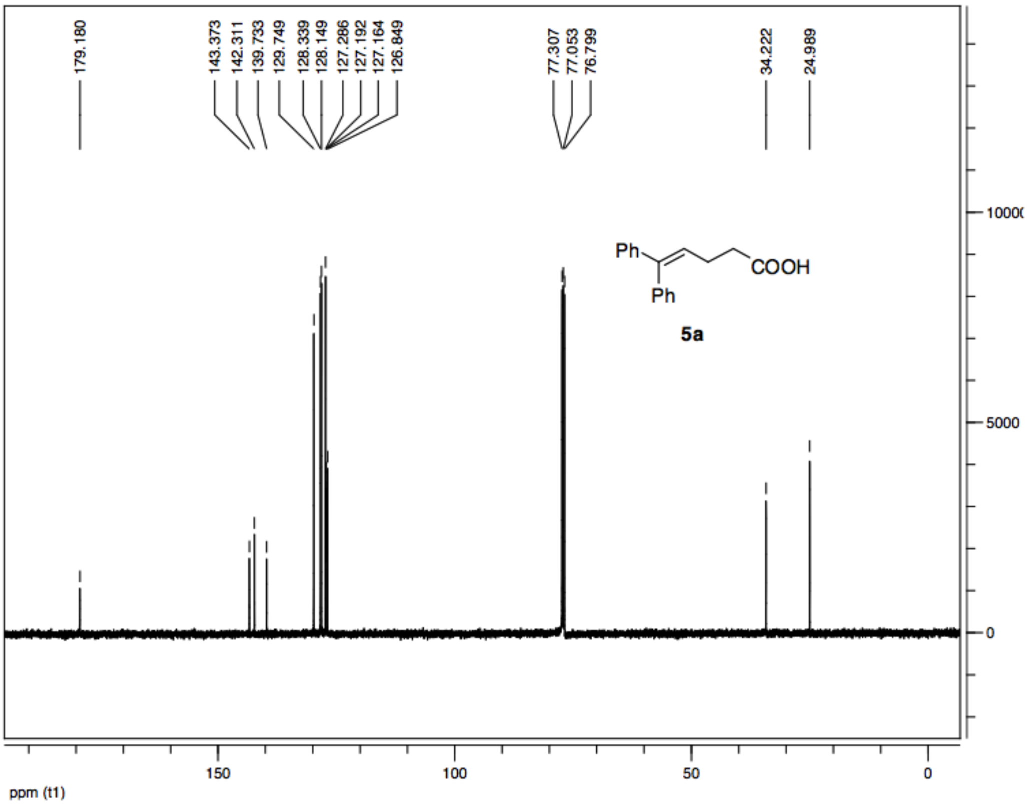
179.180
143.373
142.311
139.733
129.749
128.339
128.149
127.286
127.192
127.164
126.849
77.307
77.053
76.799
34.222
24.989
Ph
Ph
COOH
5a
10000
5000
0
150
100
50
0
ppm (t1)

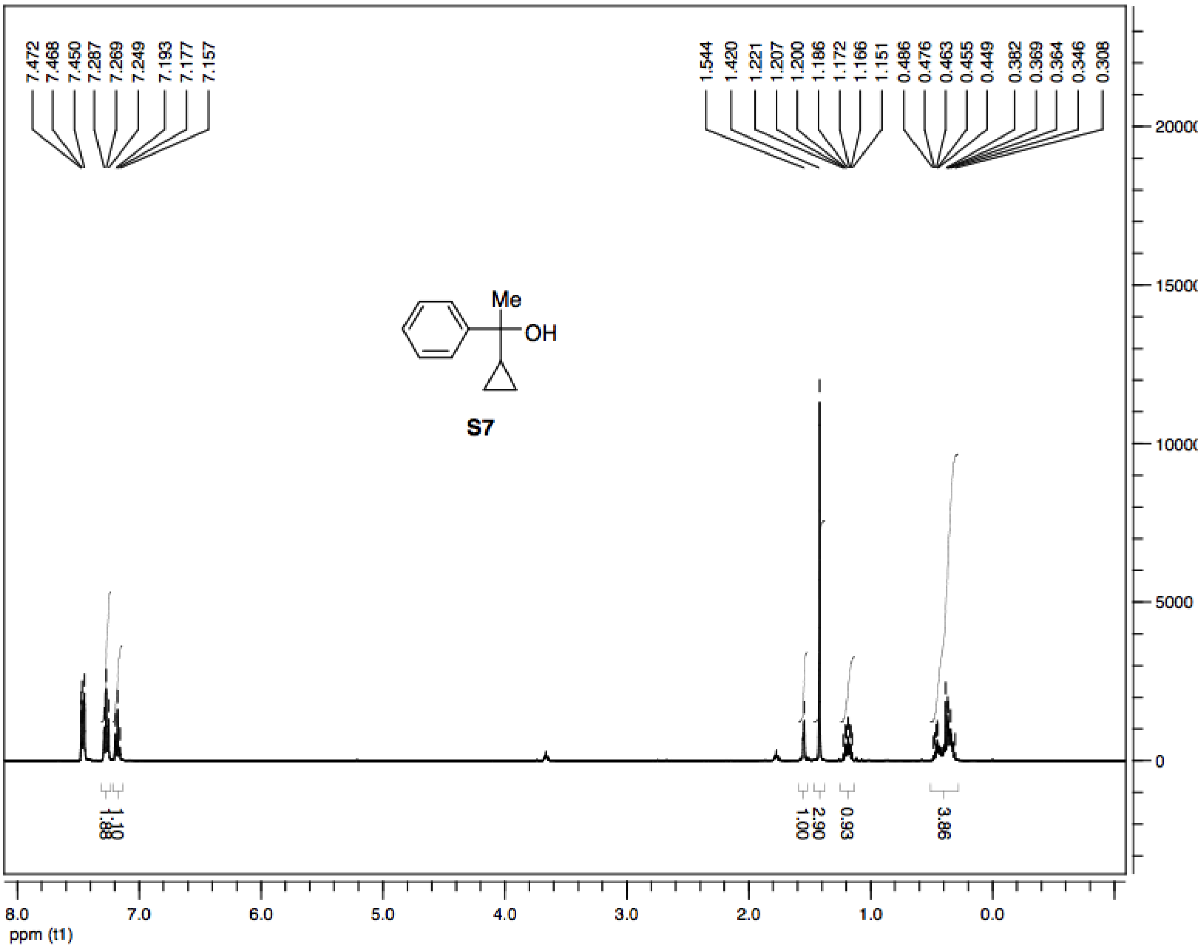
7.472
7.468
7.450
7.287
7.269
7.249
7.193
7.177
7.157
1.544
1.420
1.221
1.207
1.200
1.186
1.172
1.166
1.151
0.486
0.476
0.463
0.455
0.449
0.382
0.369
0.364
0.346
0.308
Me
OH
S7
20000
15000
10000
5000
0
1.88
1.10
1.00
2.90
0.93
3.86
8.0
7.0
6.0
5.0
4.0
3.0
2.0
1.0
0.0
ppm (t1)

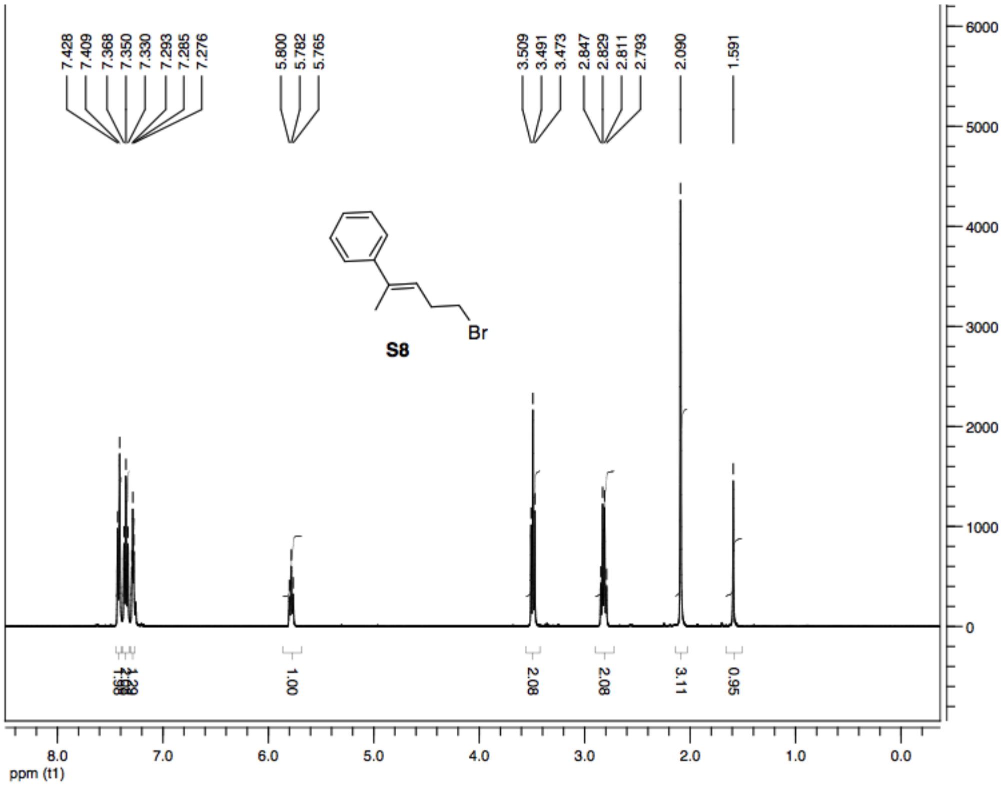
7.428
7.409
7.368
7.350
7.330
7.293
7.285
7.276
5.800
5.782
5.765
3.509
3.491
3.473
2.847
2.829
2.811
2.793
2.090
1.591
Br
S8
1.00
2.08
2.08
3.11
0.95
ppm (t1)

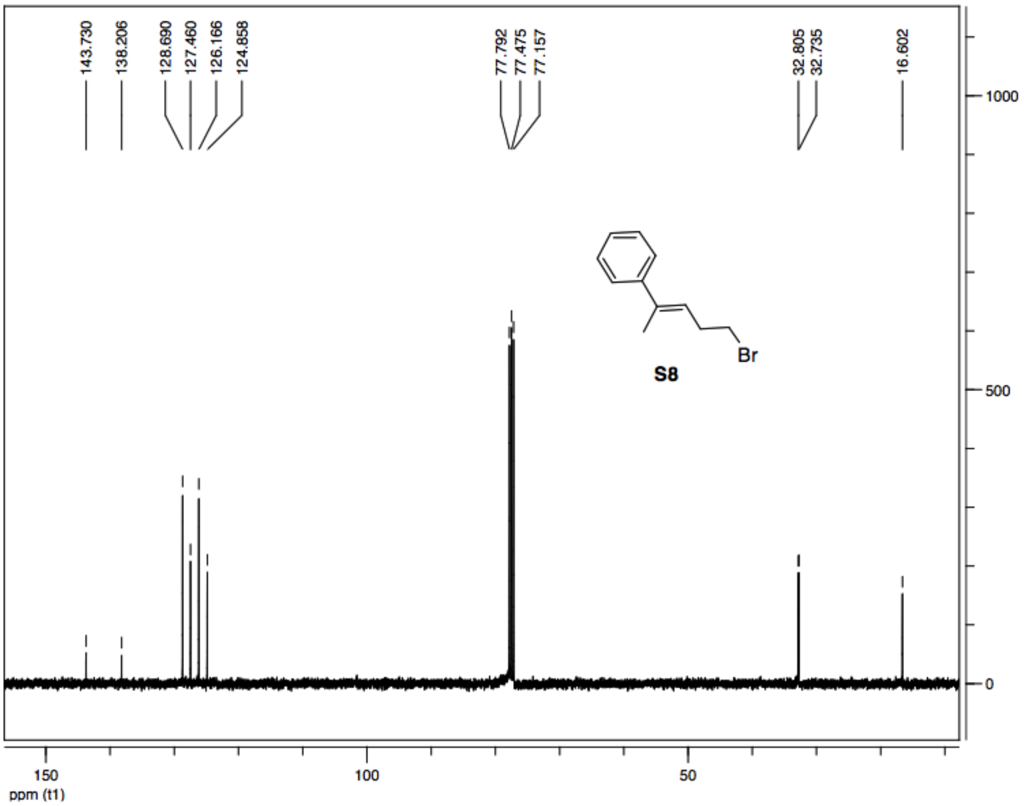
143.730
138.206
128.690
127.460
126.166
124.858
77.792
77.475
77.157
32.805
32.735
16.602
Br
S8
ppm (t1)

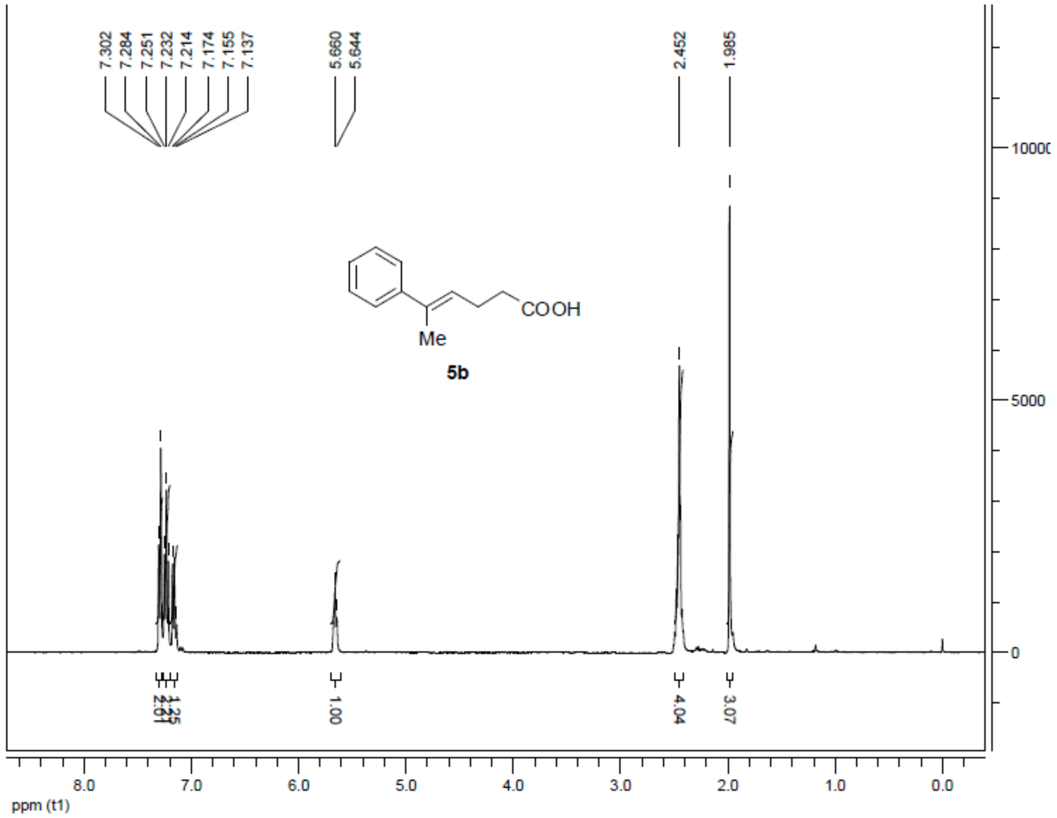

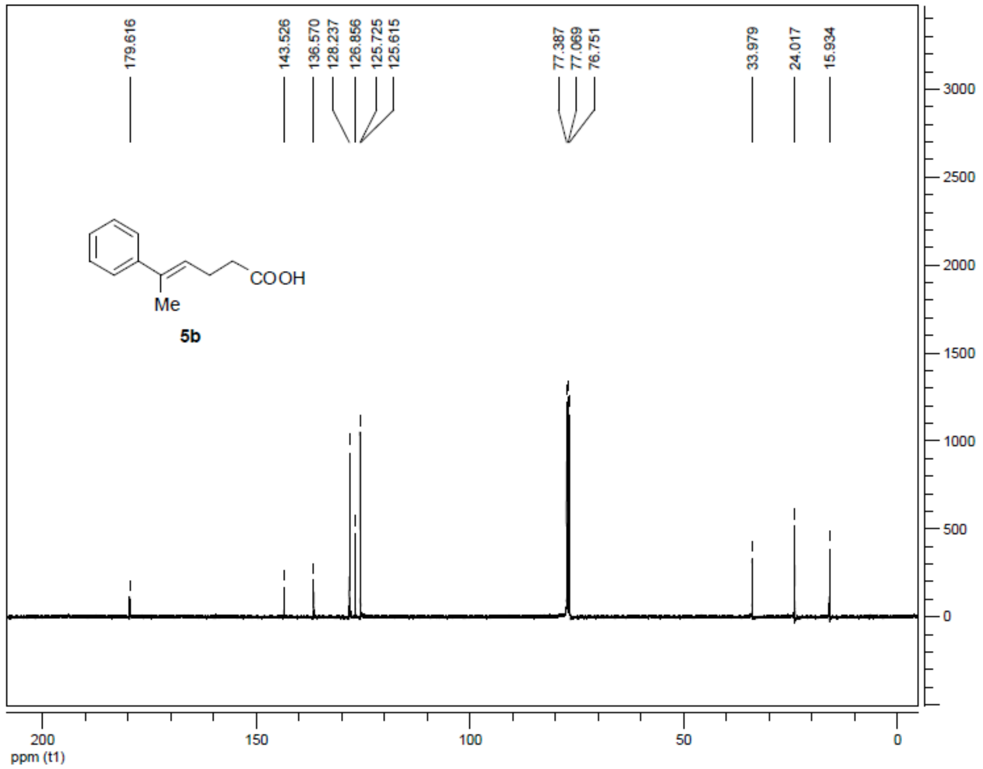

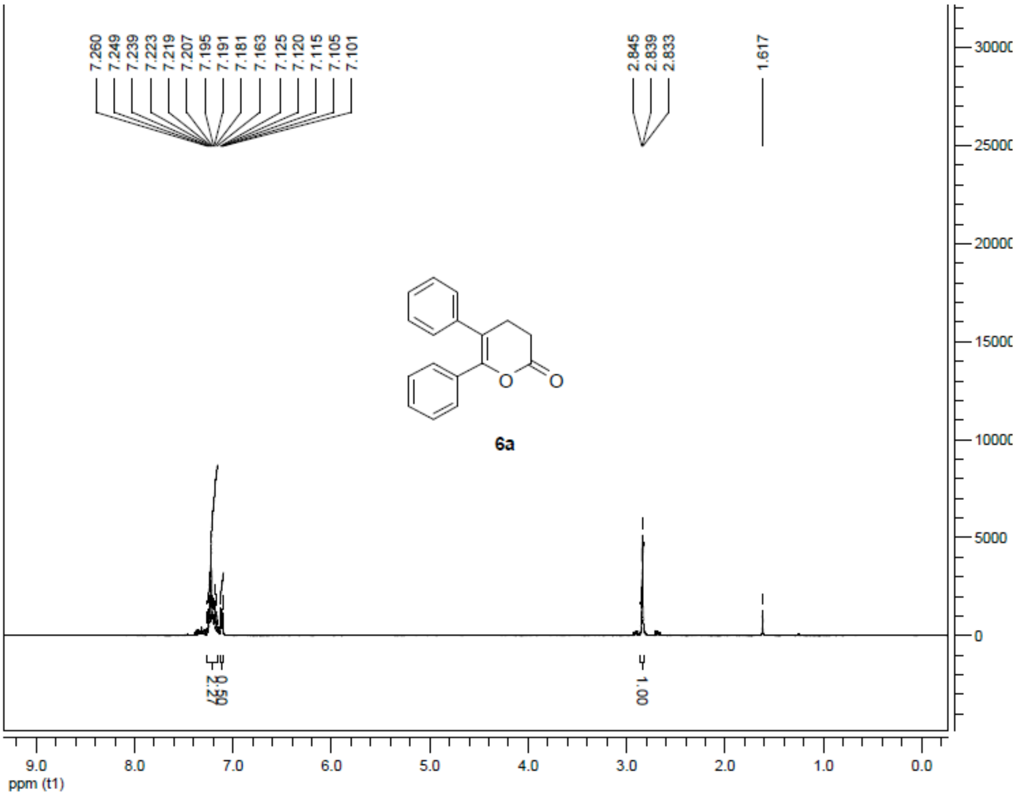
7.260
7.249
7.239
7.223
7.219
7.207
7.195
7.191
7.181
7.163
7.125
7.120
7.115
7.105
7.101
2.845
2.839
2.833
1.617
6a
1.00
ppm (t1)

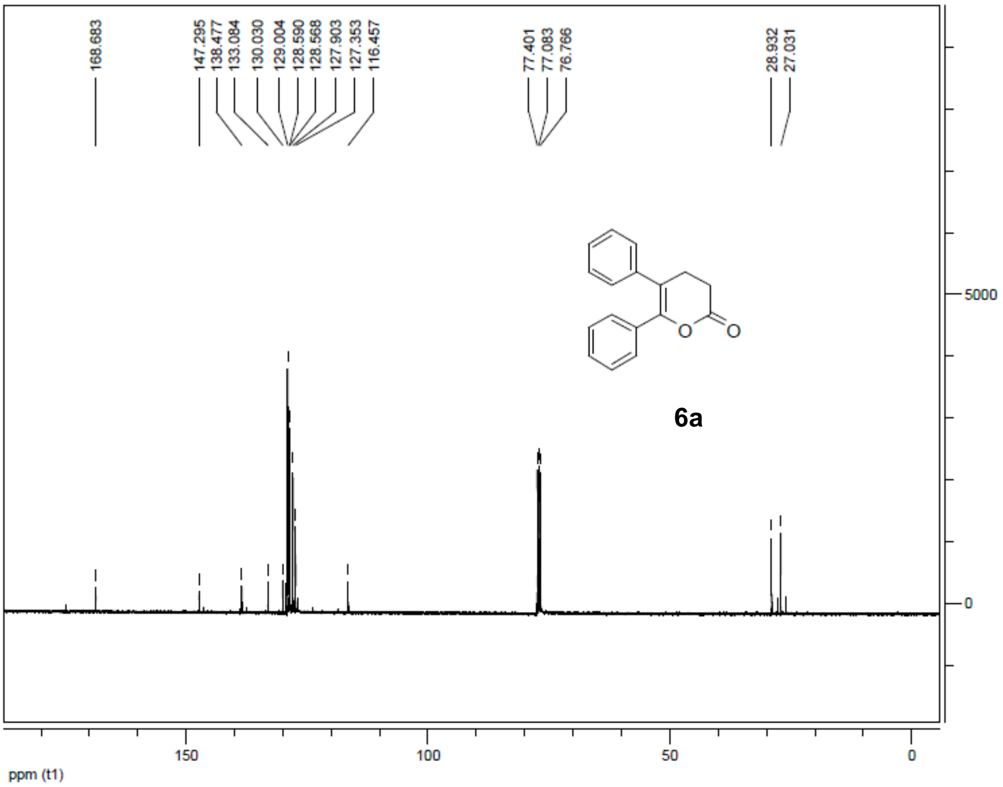
168.683
147.295
138.477
133.084
130.030
129.004
128.590
128.568
127.903
127.353
116.457
77.401
77.083
76.766
28.932
27.031
6a
ppm (t1)

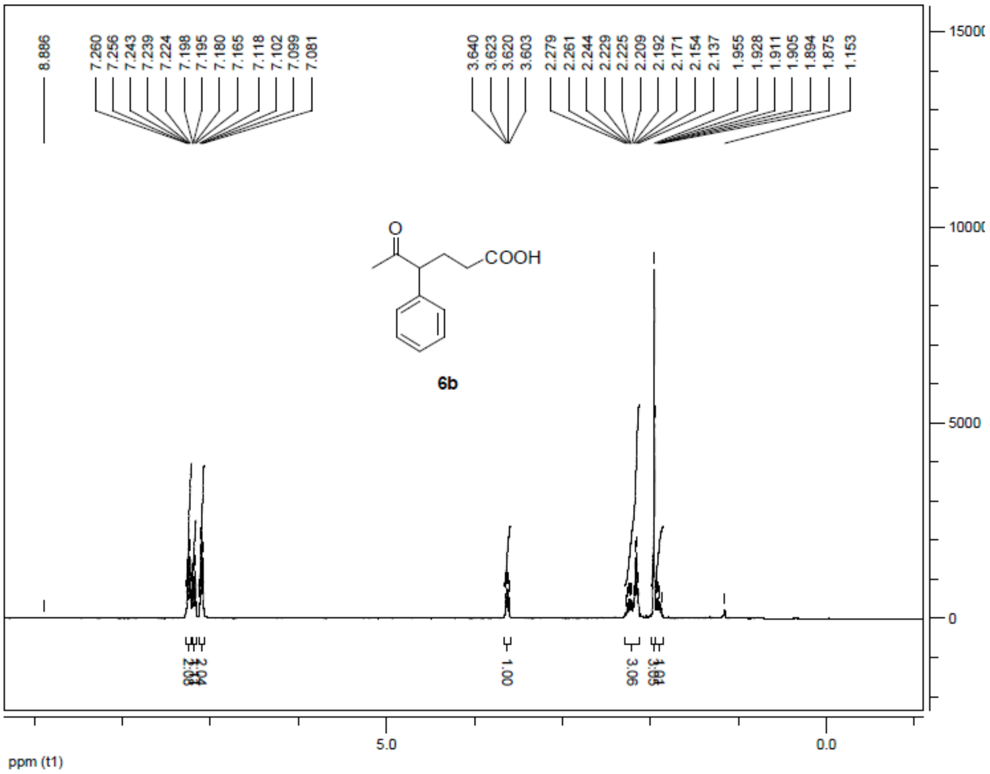
8.886
7.260
7.256
7.243
7.239
7.224
7.198
7.195
7.180
7.165
7.118
7.102
7.099
7.081
3.640
3.623
3.620
3.603
2.279
2.261
2.244
2.229
2.225
2.209
2.192
2.171
2.154
2.137
1.955
1.928
1.911
1.905
1.894
1.875
1.153
O
COOH
6b
15000
10000
5000
0
1.00
3.06
5.0
0.0
ppm (t1)

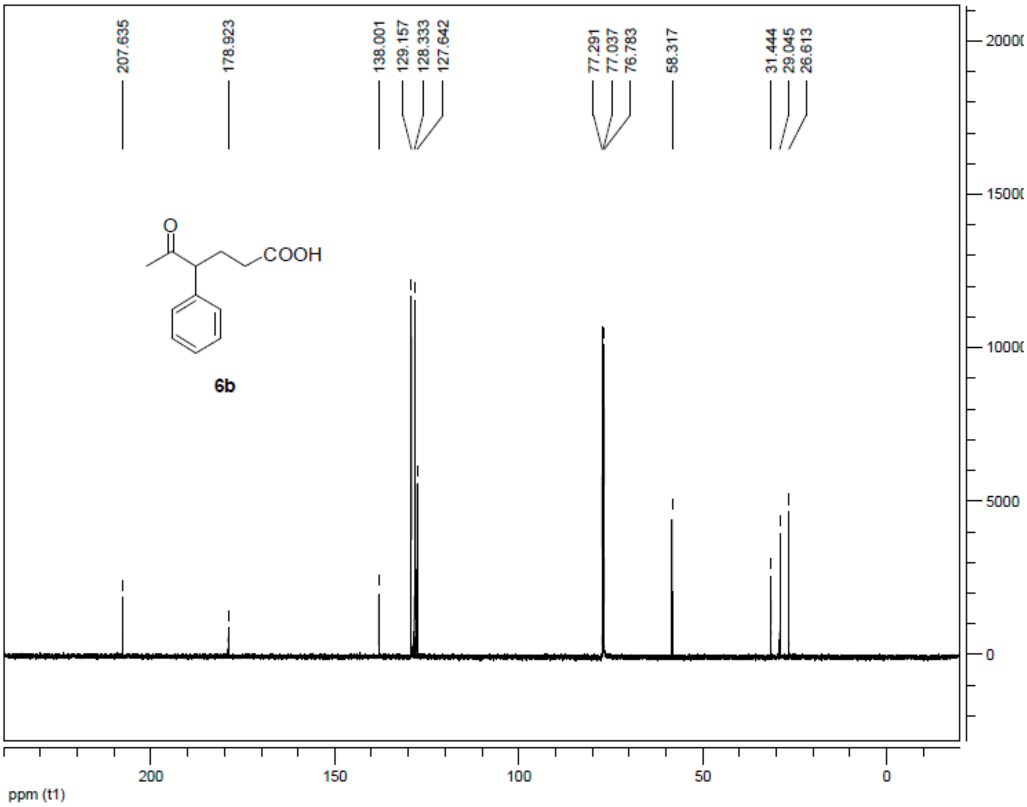
207.635
178.923
138.001
129.157
128.333
127.642
77.291
77.037
76.783
58.317
31.444
29.045
26.613
O
COOH
6b
20000
15000
10000
5000
0
200
150
100
50
0
ppm (t1)

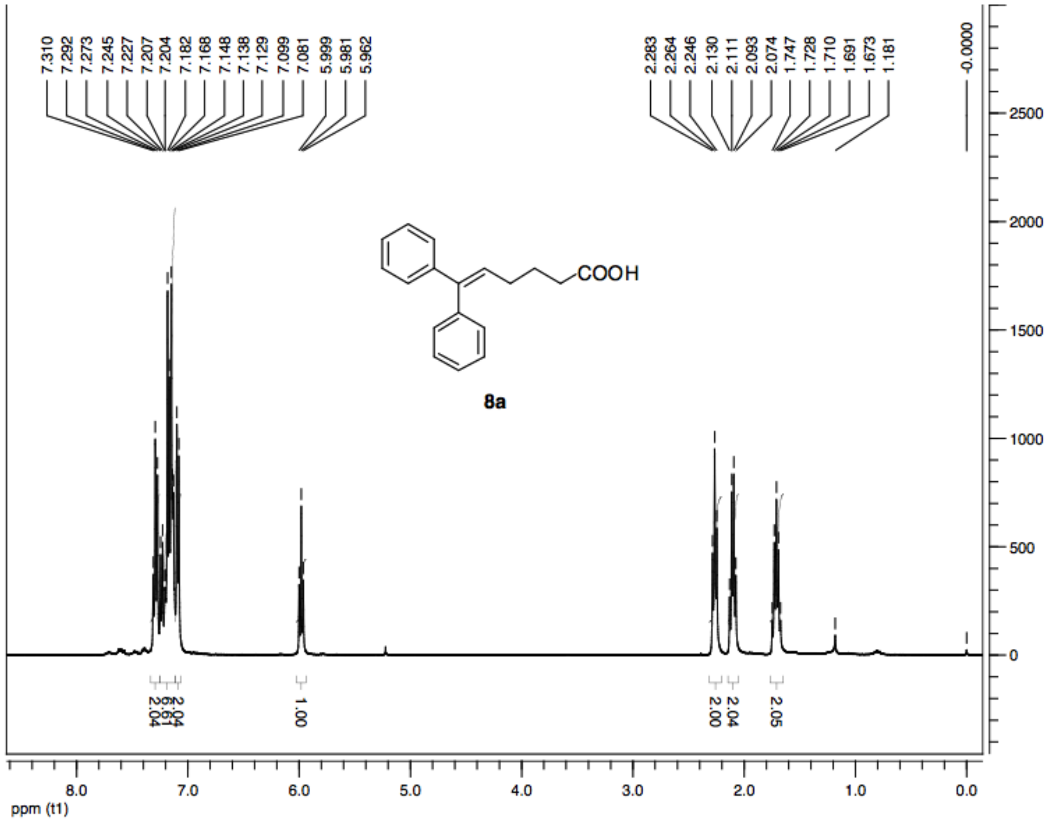
7.310
7.292
7.273
7.245
7.227
7.207
7.204
7.182
7.168
7.148
7.138
7.129
7.099
7.081
5.999
5.981
5.962
2.283
2.264
2.246
2.130
2.111
2.093
2.074
1.747
1.728
1.710
1.691
1.673
1.181
-0.0000
COOH
8a
2.04
6.61
2.04
1.00
2.00
2.04
2.05
ppm (t1)

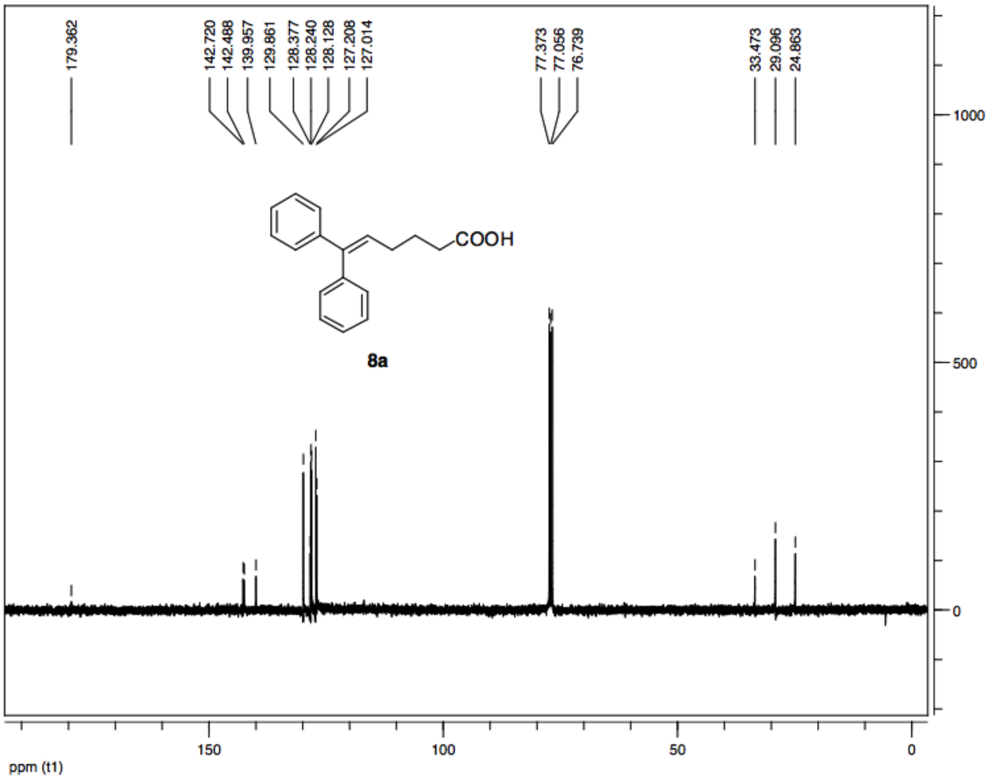
179.362
142.720
142.488
139.957
129.861
128.377
128.240
128.128
127.208
127.014
77.373
77.056
76.739
33.473
29.096
24.863
COOH
8a
ppm (t1)

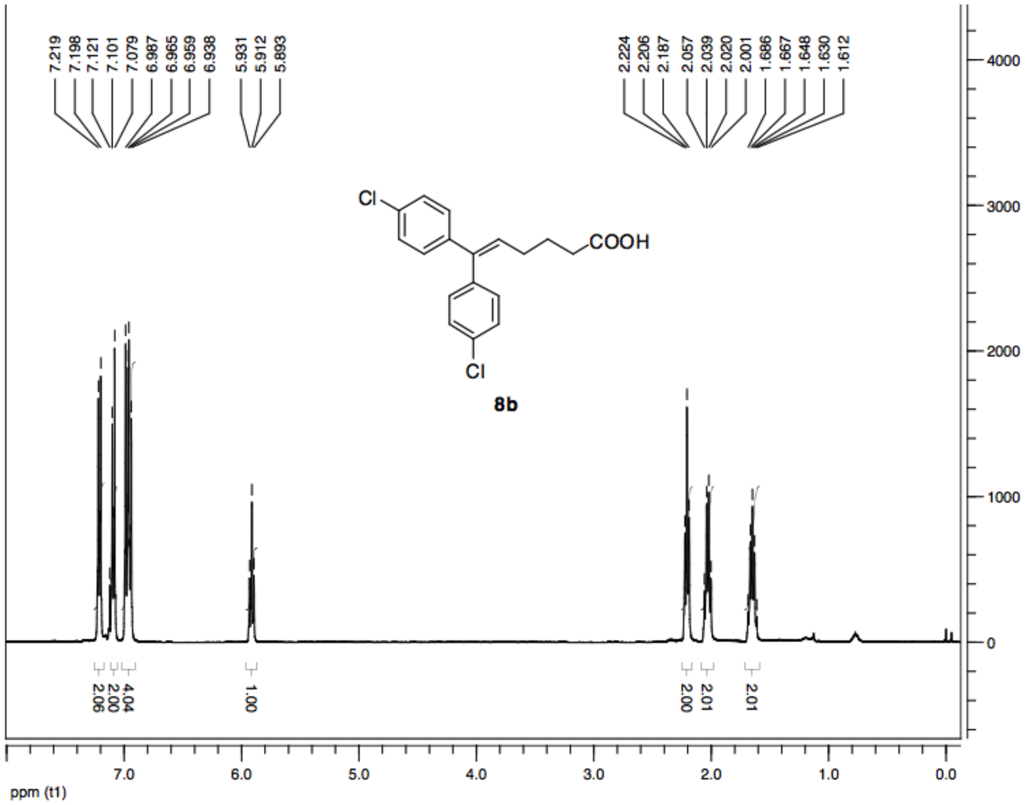
7.219
7.198
7.121
7.101
7.079
6.987
6.965
6.959
6.938
5.931
5.912
5.893
2.224
2.206
2.187
2.057
2.039
2.020
2.001
1.686
1.667
1.648
1.630
1.612
Cl
COOH
Cl
8b
2.06
2.00
4.04
1.00
2.00
2.01
2.01
ppm (t1)

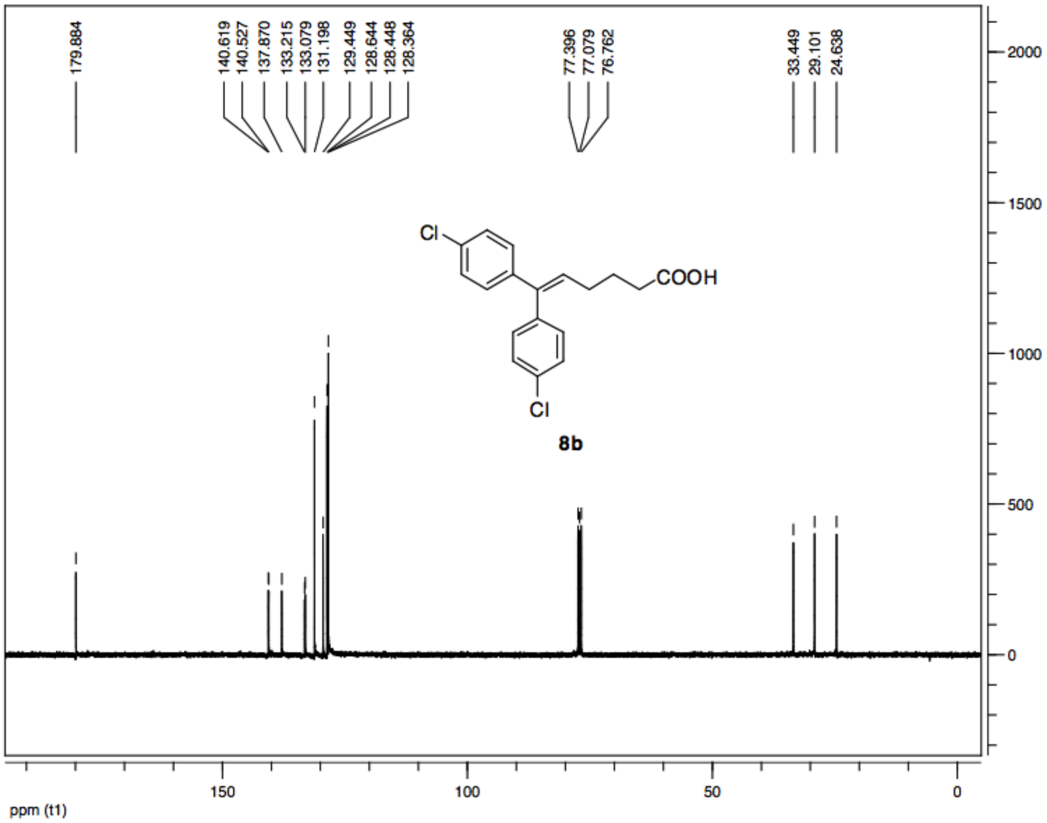
179.884
140.619
140.527
137.870
133.215
133.079
131.198
129.449
128.644
128.448
128.364
77.396
77.079
76.762
33.449
29.101
24.638
Cl
COOH
Cl
8b
ppm (t1)

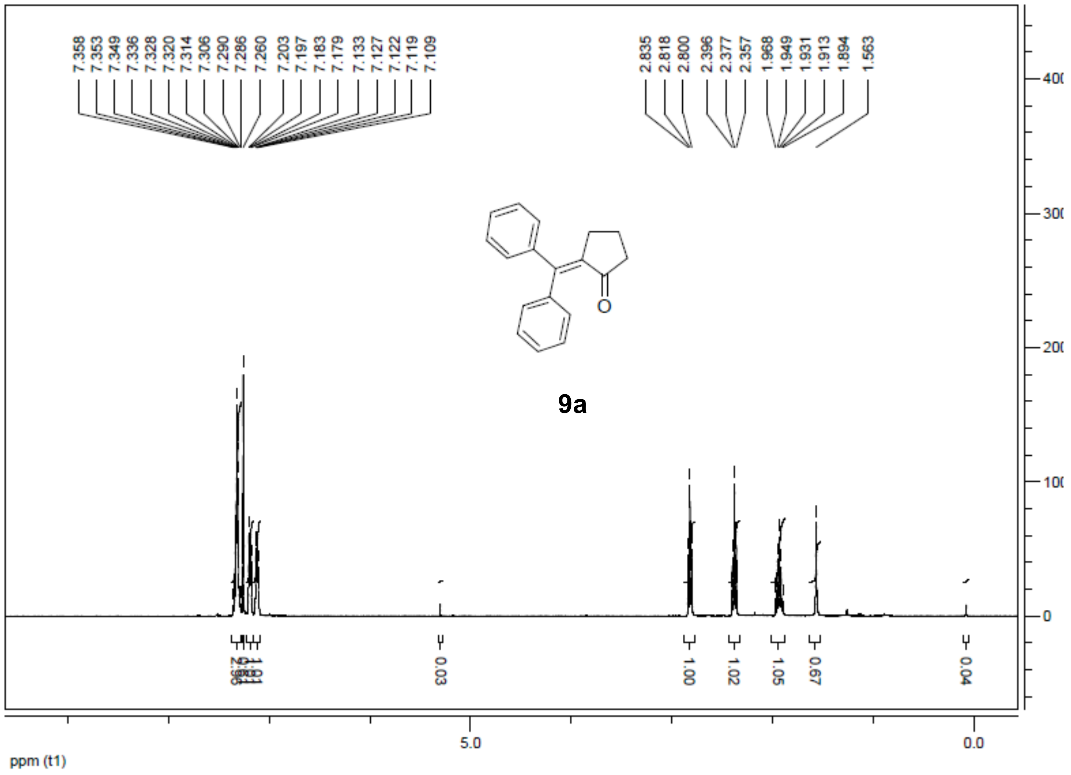
7.358 7.353 7.349 7.336 7.328 7.320 7.314 7.306 7.290 7.286 7.260 7.203 7.197 7.183 7.179 7.133 7.127 7.122 7.119 7.109
2.835 2.818 2.800 2.396 2.377 2.357 1.968 1.949 1.931 1.913 1.894 1.563
O
9a
0.03
1.00
1.02
1.05
0.67
0.04
5.0
0.0
ppm (t1)

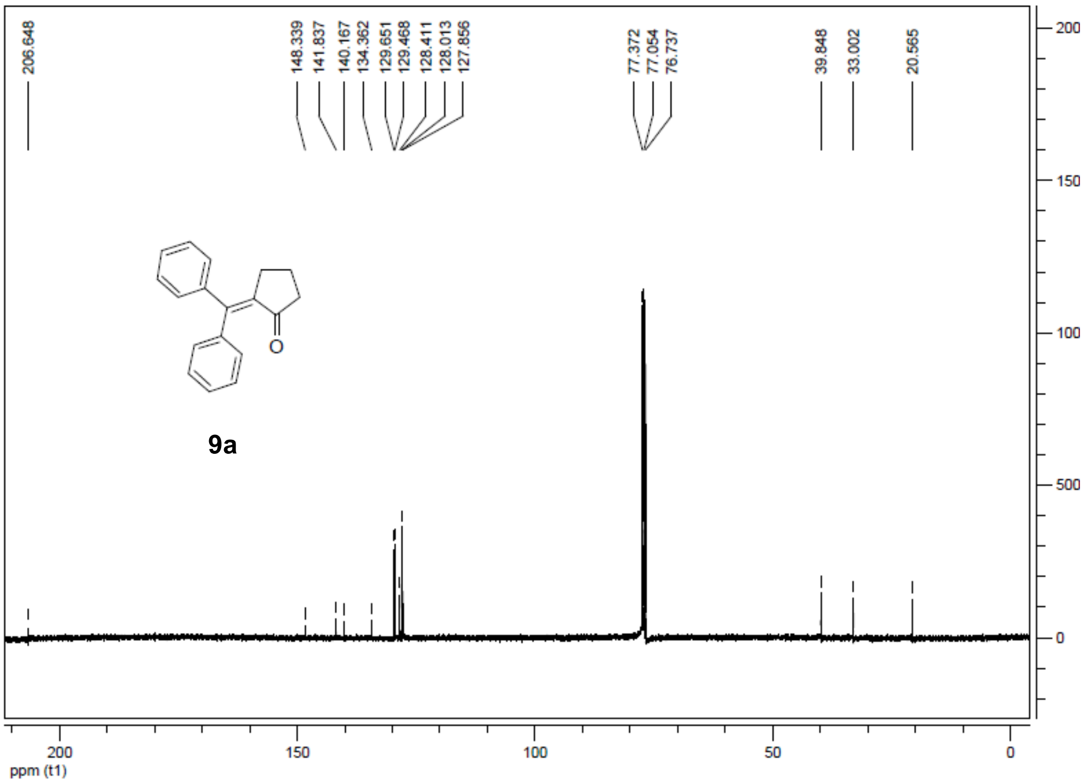
206.648
148.339 141.837 140.167 134.362 129.651 129.468 128.411 128.013 127.856
77.372 77.054 76.737
39.848 33.002 20.565
O
9a
200
150
100
50
0
ppm (t1)

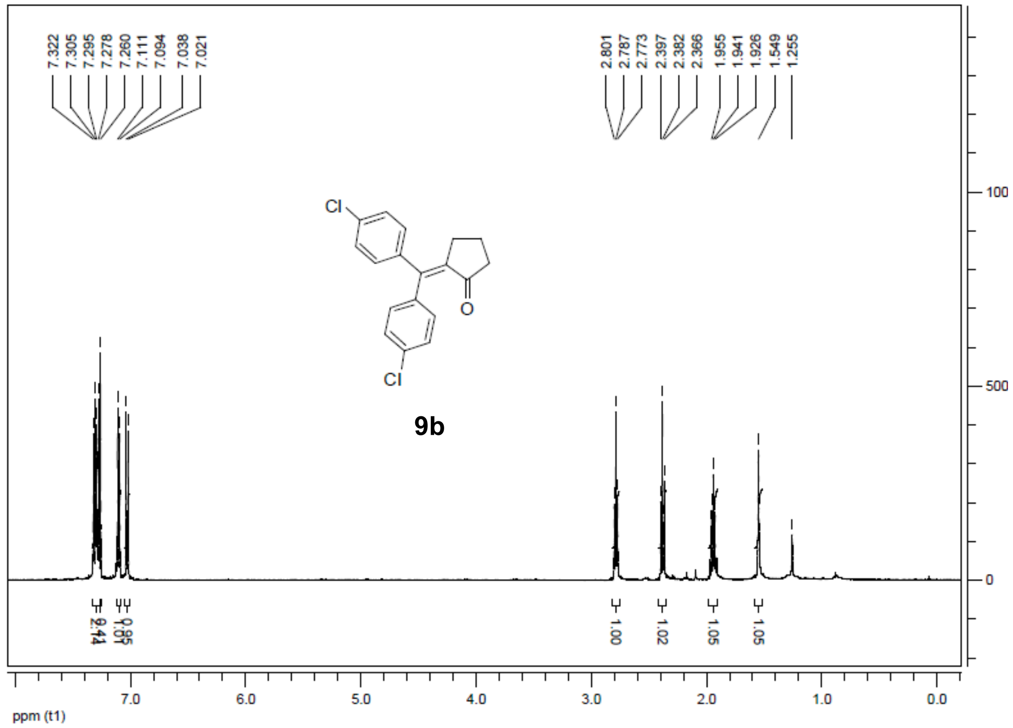
7.322
7.305
7.295
7.278
7.260
7.111
7.094
7.038
7.021
2.801
2.787
2.773
2.397
2.382
2.366
1.955
1.941
1.926
1.549
1.255
Cl
Cl
O
9b
ppm (t1)

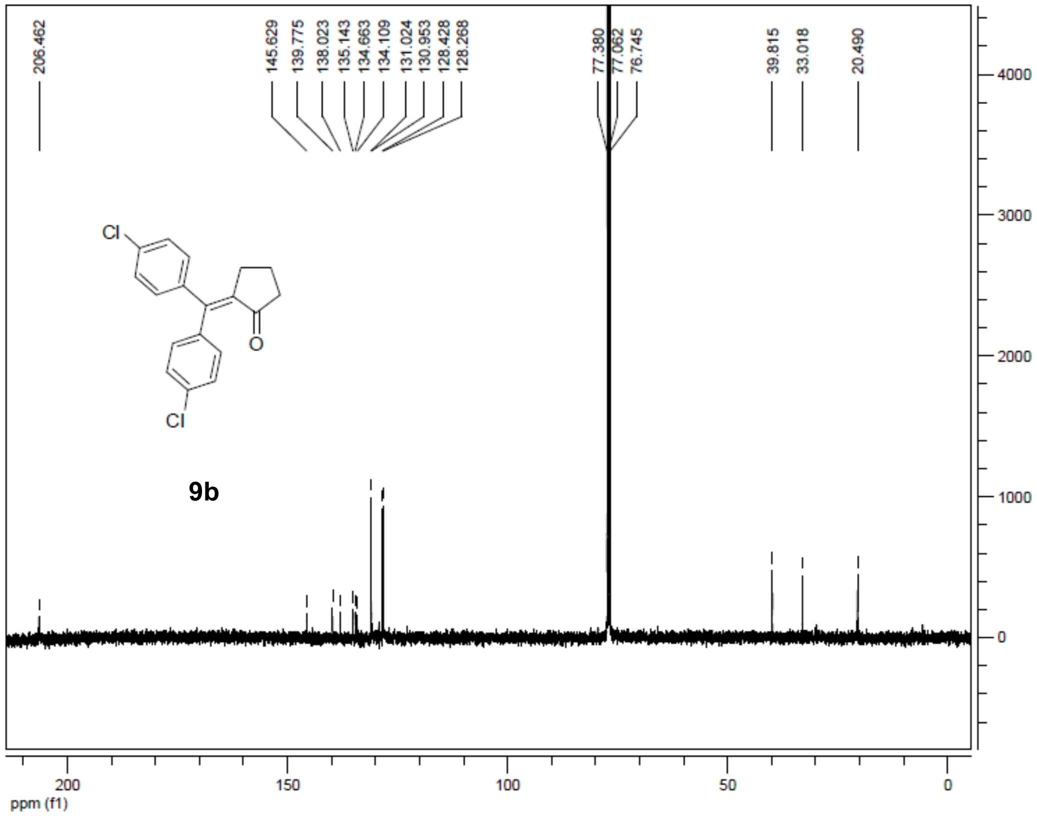
206.462
145.629
139.775
138.023
135.143
134.663
134.109
131.024
130.953
128.428
128.268
77.380
77.062
76.745
39.815
33.018
20.490
Cl
Cl
O
9b
ppm (f1)

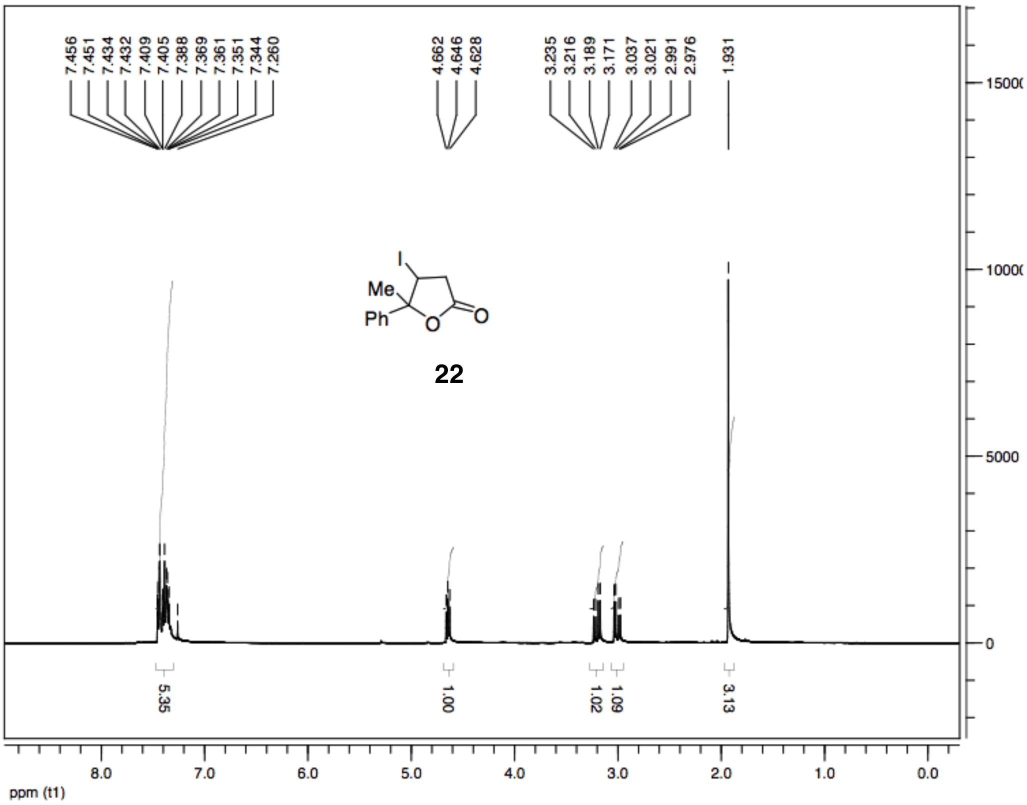
7.456
7.451
7.434
7.432
7.409
7.405
7.388
7.369
7.361
7.351
7.344
7.260
4.662
4.646
4.628
3.235
3.216
3.189
3.171
3.037
3.021
2.991
2.976
1.931
Me
Ph
I
O
O
22
5.35
1.00
1.02
1.09
3.13
15000
10000
5000
0
8.0
7.0
6.0
5.0
4.0
3.0
2.0
1.0
0.0
ppm (t1)

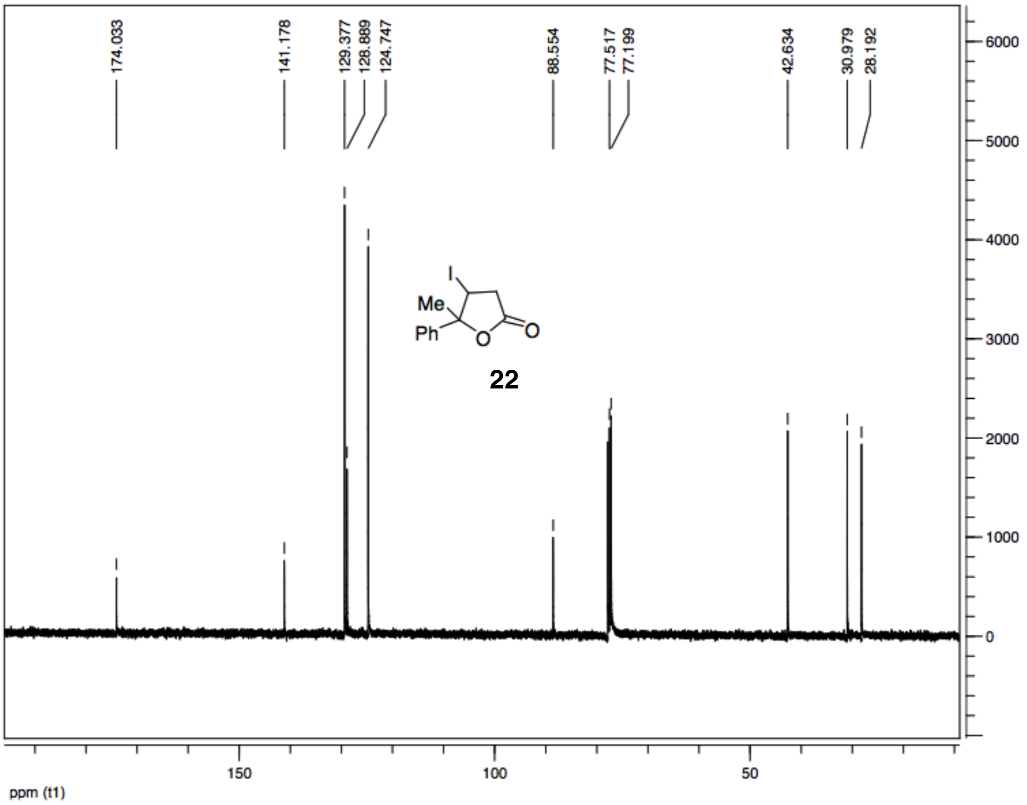
174.033
141.178
129.377
128.889
124.747
88.554
77.517
77.199
42.634
30.979
28.192
Me
Ph
I
O
O
22
6000
5000
4000
3000
2000
1000
0
150
100
50
ppm (t1)

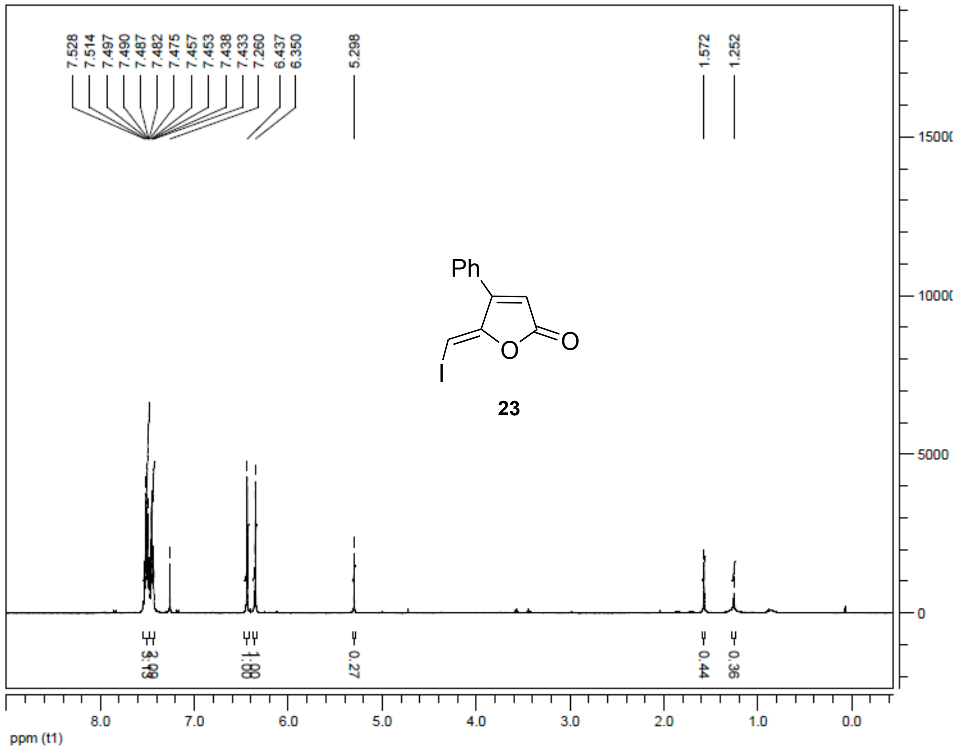

7.528
7.514
7.497
7.490
7.487
7.482
7.475
7.457
7.453
7.438
7.433
7.260
6.437
6.350
5.298
1.572
1.252
Ph
O
O
I
23
3.19
1.00
0.27
0.44
0.36
15000
10000
5000
0
8.0
7.0
6.0
5.0
4.0
3.0
2.0
1.0
0.0
ppm (t1)

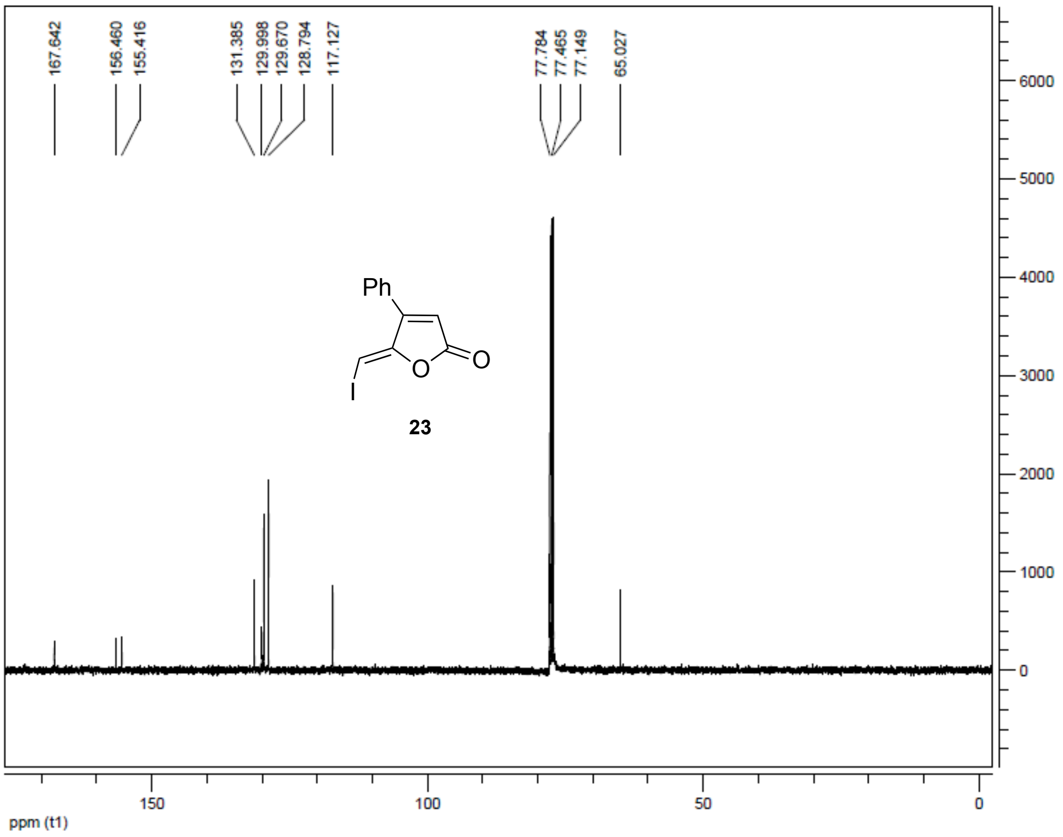

167.642
156.460
155.416
131.385
129.998
129.670
128.794
117.127
77.784
77.465
77.149
65.027
Ph
O
O
I
23
6000
5000
4000
3000
2000
1000
0
150
100
50
0
ppm (t1)

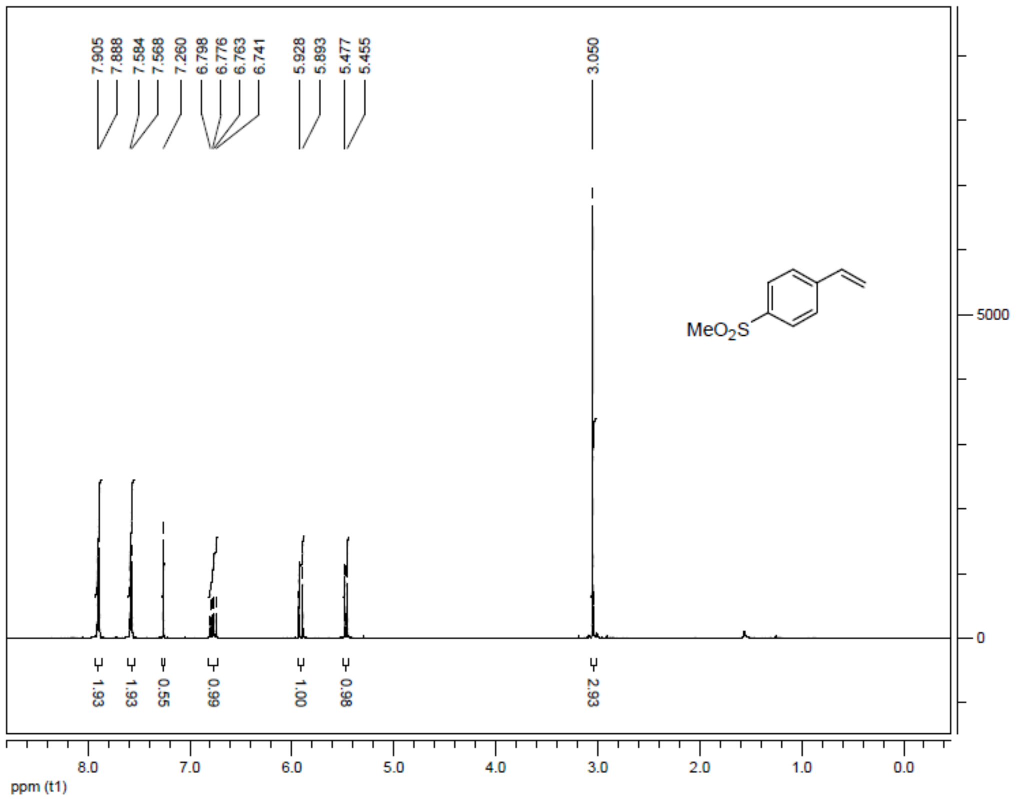
7.905
7.888
7.584
7.568
7.260
6.798
6.776
6.763
6.741
5.928
5.893
5.477
5.455
3.050
MeO_2S
1.93
1.93
0.55
0.99
1.00
0.98
2.93
5000
0
8.0
7.0
6.0
5.0
4.0
3.0
2.0
1.0
0.0
ppm (t1)

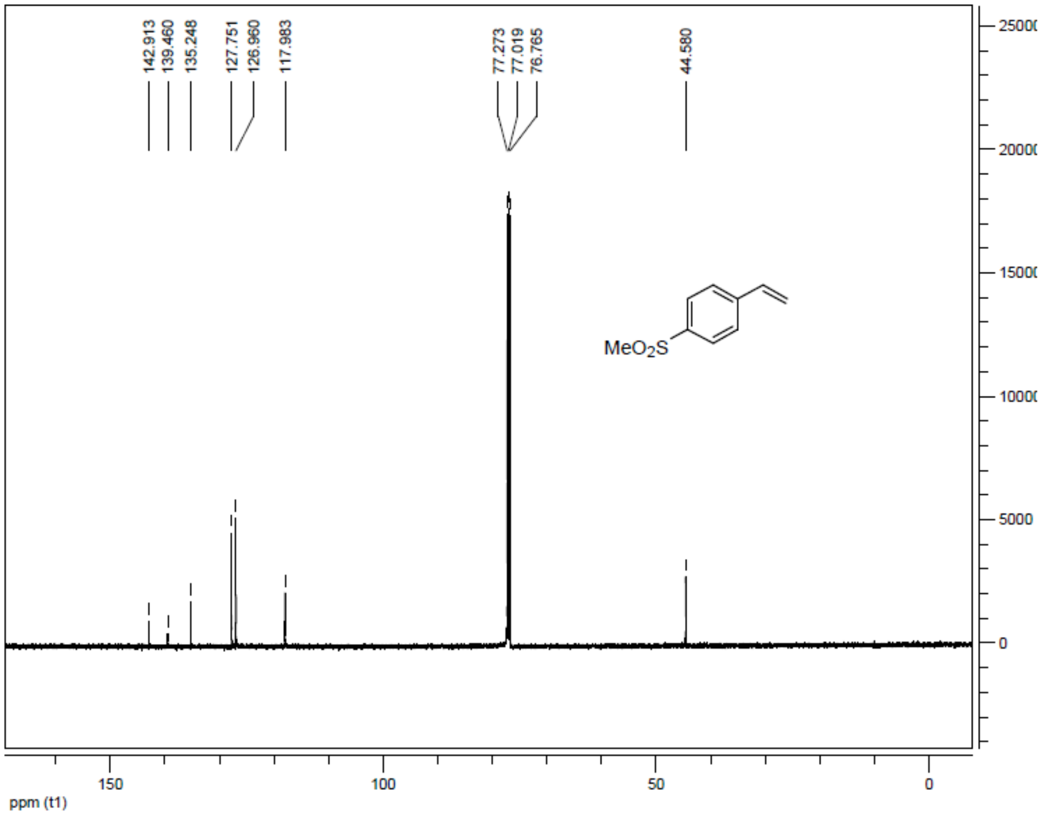
142.913
139.460
135.248
127.751
126.960
117.983
77.273
77.019
76.765
44.580
MeO_2S
150
100
50
0
ppm (t1)

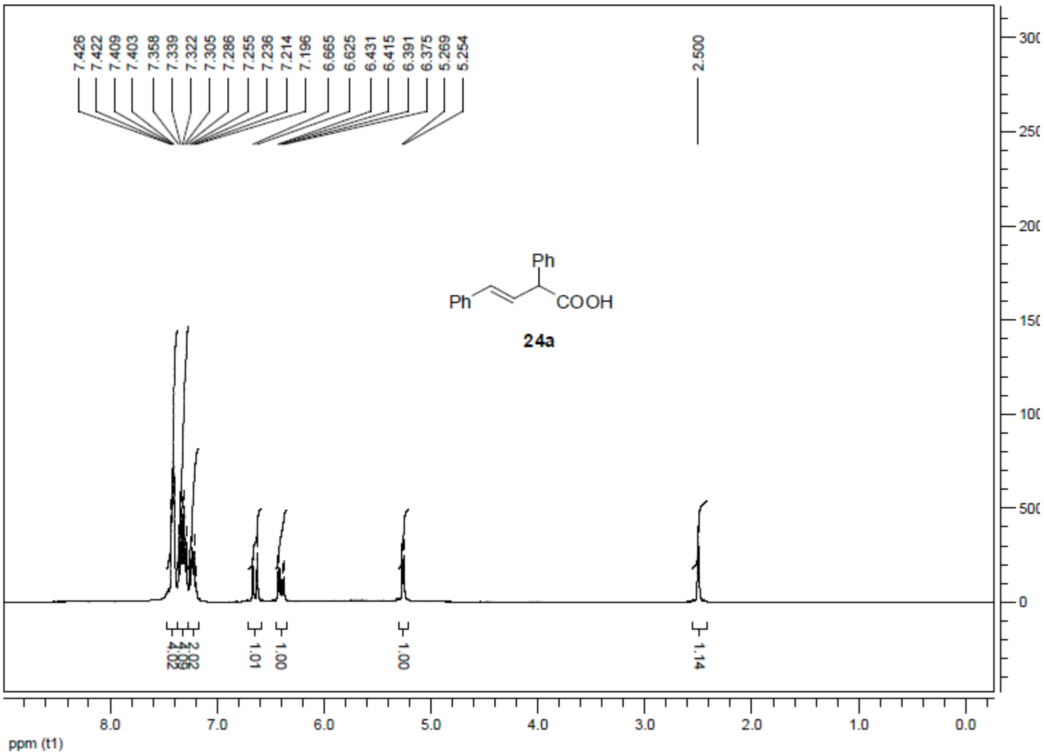
7.426
7.422
7.409
7.403
7.358
7.339
7.322
7.305
7.286
7.255
7.236
7.214
7.196
6.665
6.625
6.431
6.415
6.391
6.375
5.269
5.254
2.500
Ph
Ph
COOH
24a
4.02
4.09
2.02
1.01
1.00
1.00
1.14
8.0
7.0
6.0
5.0
4.0
3.0
2.0
1.0
0.0
ppm (t1)

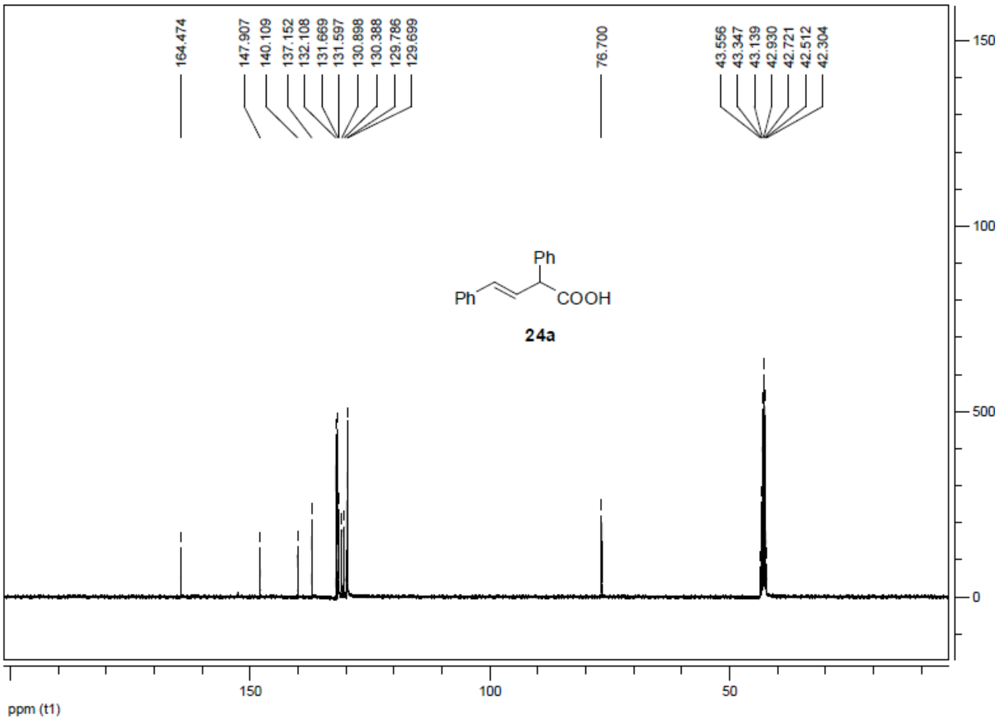
164.474
147.907
140.109
137.152
132.108
131.669
131.597
130.898
130.388
129.786
129.699
76.700
43.556
43.347
43.139
42.930
42.721
42.512
42.304
Ph
Ph
COOH
24a
150
100
50
ppm (t1)

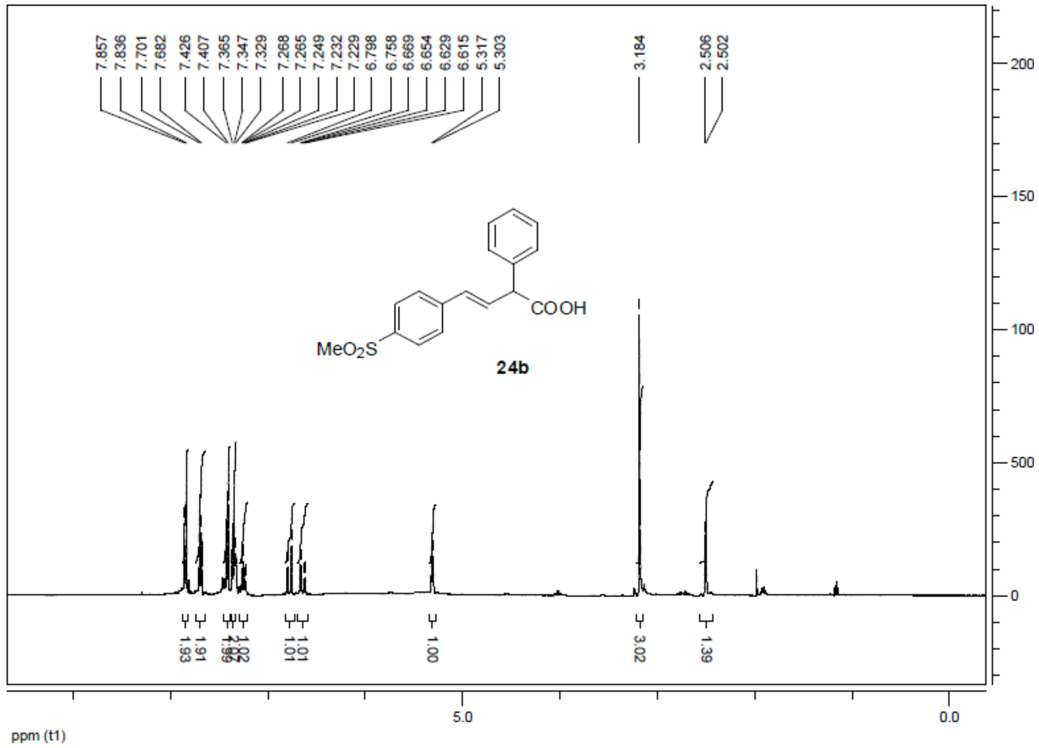
7.857
7.836
7.701
7.682
7.426
7.407
7.365
7.347
7.329
7.268
7.265
7.249
7.232
7.229
6.798
6.758
6.669
6.654
6.629
6.615
5.317
5.303
3.184
2.506
2.502
COOH
MeO2S
24b
1.93
1.91
1.99
1.02
1.01
1.01
1.00
3.02
1.39
5.0
0.0
ppm (t1)
200
150
100
500
0

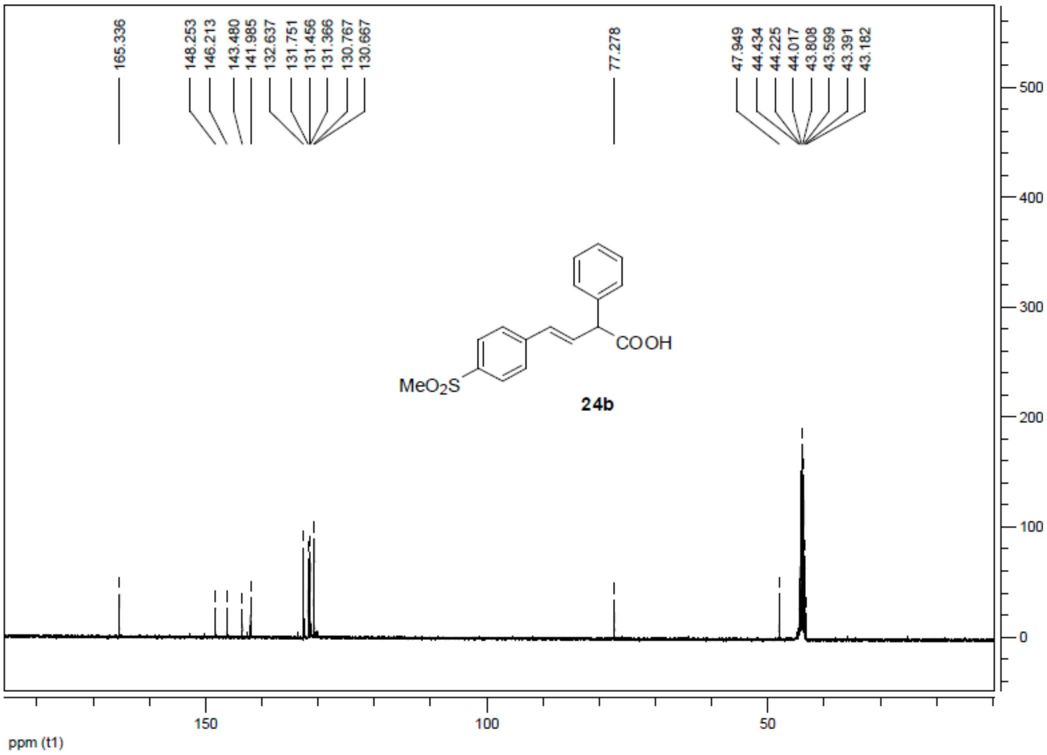
165.336
148.253
146.213
143.480
141.985
132.637
131.751
131.456
131.366
130.767
130.667
77.278
47.949
44.434
44.225
44.017
43.808
43.599
43.391
43.182
COOH
MeO2S
24b
150
100
50
ppm (t1)
500
400
300
200
100
0

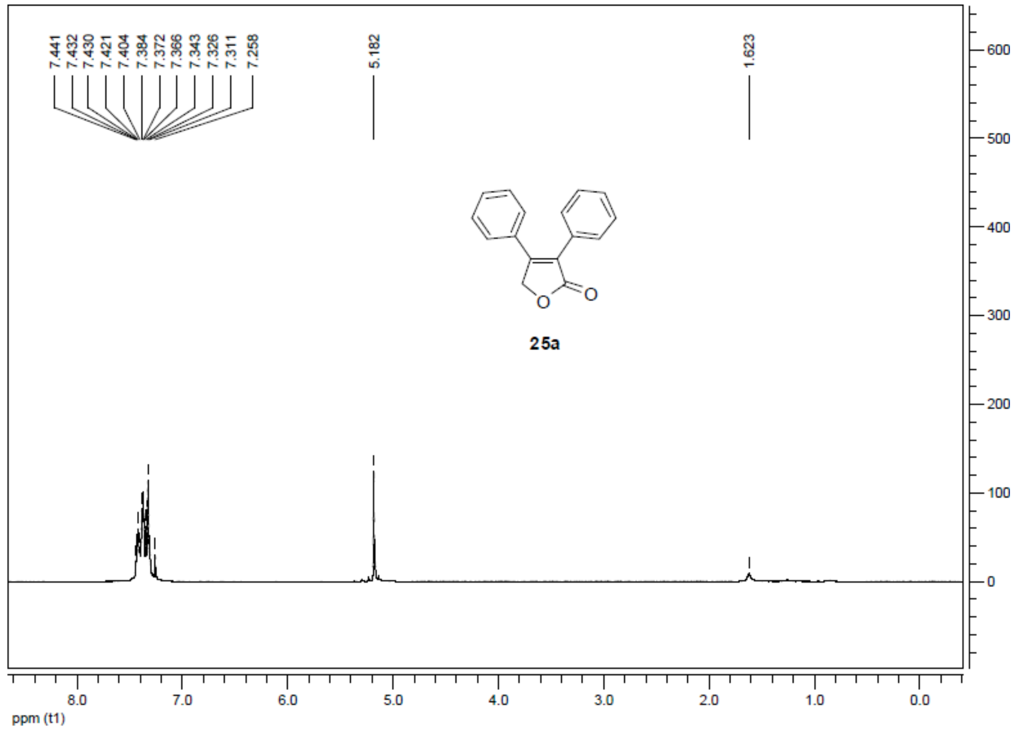
7.441
7.432
7.430
7.421
7.404
7.384
7.372
7.366
7.343
7.326
7.311
7.258
5.182
1.623
25a
ppm (t1)

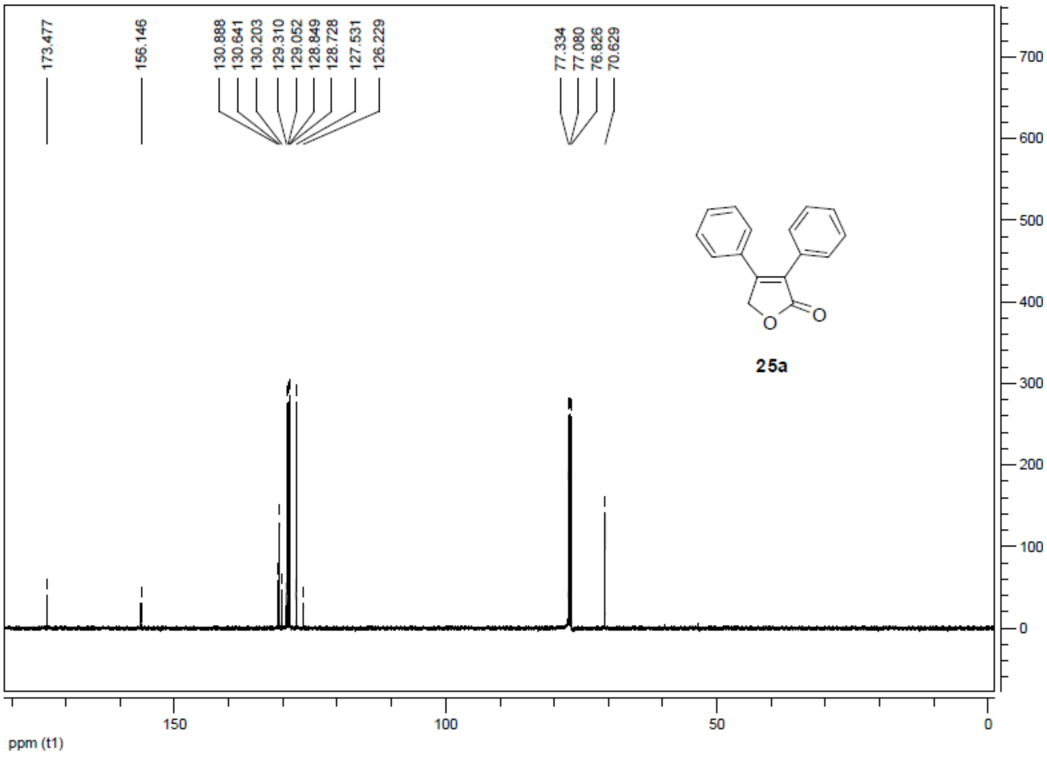
173.477
156.146
130.888
130.641
130.203
129.310
129.052
128.849
128.728
127.531
126.229
77.334
77.080
76.826
70.629
25a
ppm (t1)

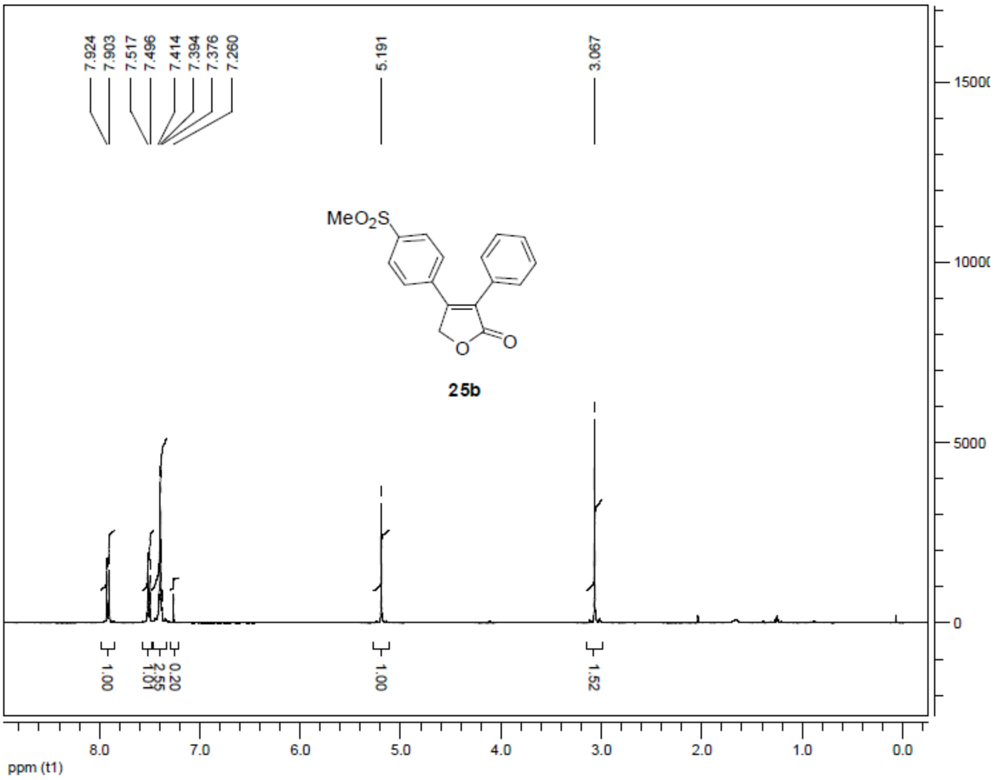
7.924
7.903
7.517
7.496
7.414
7.394
7.376
7.260
5.191
3.067
MeO2S
25b
1.00
1.01
2.55
0.20
1.00
1.52
15000
10000
5000
0
8.0
7.0
6.0
5.0
4.0
3.0
2.0
1.0
0.0
ppm (t1)

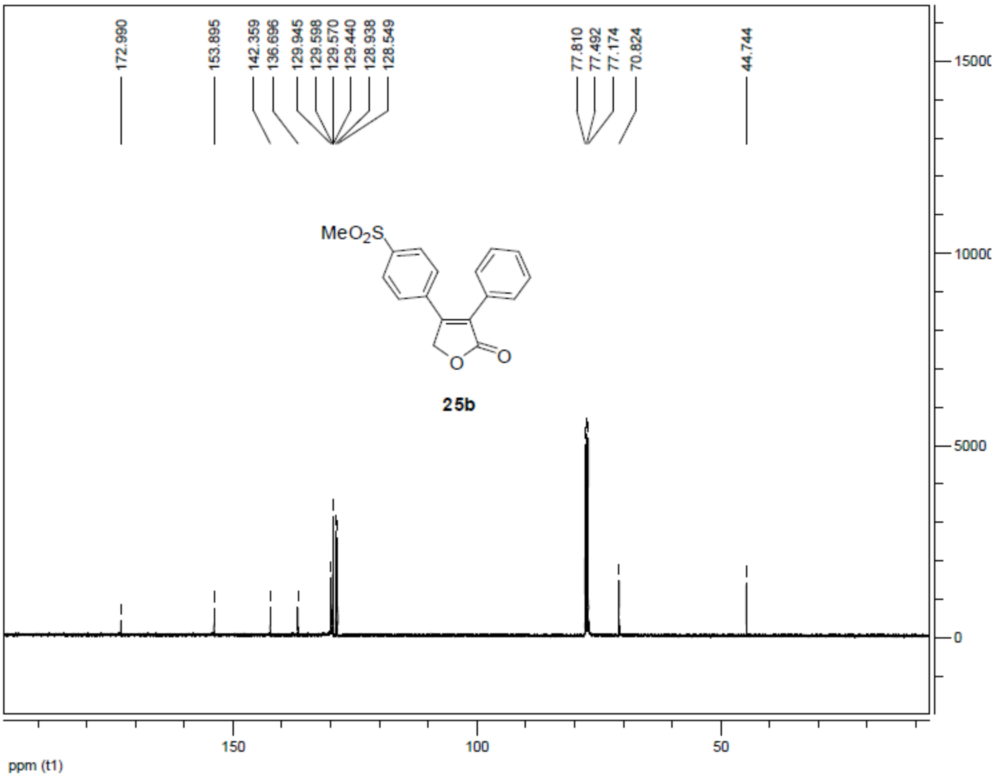
172.990
153.895
142.359
136.696
129.945
129.598
129.570
129.440
128.938
128.549
77.810
77.492
77.174
70.824
44.744
MeO2S
25b
15000
10000
5000
0
150
100
50
ppm (t1)
